# Supplementary material for: Bystanders’ attitudes towards drone delivered Automated External Defibrillators for out-of-hospital cardiac arrest: A qualitative interview study
Source: PLoS One. 2025 Dec 3;20(12):e0337082. doi: 10.1371/journal.pone.0337082 (PMC12674532; doi:10.1371/journal.pone.0337082)
Supplement: S1 Table — (DOCX) [file pone.0337082.s001.docx]

# **S1 Table. Possible barriers and facilitators to lay bystander retrieval and use of a drone-delivered defibrillator**

| **COM-B component** |  | **Factors affecting drone-delivered AED retrieval and use** |
| --- | --- | --- |
| Capability | Barriers | A lack of familiarity with drone technology. |
|  |  | A lack of familiarity with the drone-delivered defibrillation process. |
| Opportunity | Barrier | Low public acceptance of drone-delivered defibrillation. |
| Motivation | Barriers | Concerns about the drone not delivering the defibrillator close enough to the incident location, concerns about the bystander taking too long to reach the AED and/or concerns about the bystander not identifying the device (quickly). |
|  |  | Low bystander self-efficacy in relation to drone-delivered defibrillator retrieval and use. |
|  |  | Concerns about becoming overwhelmed and burnt out from retrieving and using the drone-delivered defibrillator. |
|  |  | Concerns about external factors impacting the drone-delivered defibrillation process (e.g. drone battery malfunctioning, drone safety and privacy concerns, extreme weather conditions, poor visibility). |
|  |  | Concerns about the lack of human intervention and expertise in drone technology. |
|  |  | Concerns that the call-handler might not know how to support the bystander through the drone-delivered defibrillation process or have enough time to do this. |
|  |  | Reduced motivation to leave the patient’s side to retrieve the drone-delivered defibrillator due to:   1. adhering to currently available protocols for Community First Responders, which do not recommend a lone bystander pauses CPR to get an AED; 2. fears that the patient might die or be seriously harmed if left alone; 3. concerns that the ambulance crew would arrive before the drone delivered the device; and, 4. concerns about personal safety while retrieving the AED. |
|  | Facilitators | Confidence in the drone to deliver a defibrillator quickly to the scene of a cardiac arrest. |
|  |  | High bystander self-efficacy in relation to drone-delivered defibrillator retrieval and use. |
|  |  | Increased motivation to leave the patient to retrieve the device if:   1. a second bystander is present at the scene; 2. the patient or defibrillator is within the bystander’s line of sight; and/or, 3. the bystander accepts that leaving the patient to get the device will maximise patient survival. |
|  |  | Belief that drone-delivered defibrillation can increase patient survival. |
|  |  | Belief in the importance of the call-handler to support the bystander retrieve and use the drone-delivered AED by providing (non)medical advice during the call. |
