# Supplementary material for: Bystanders’ attitudes towards drone delivered Automated External Defibrillators for out-of-hospital cardiac arrest: A qualitative interview study
Source: PLoS One. 2025 Dec 3;20(12):e0337082. doi: 10.1371/journal.pone.0337082 (PMC12674532; doi:10.1371/journal.pone.0337082)
Supplement: S2 Table — (DOCX) [file pone.0337082.s002.docx]

# **S2 Table. Potential interventions for drone-delivered defibrillator retrieval by an out-of-hospital cardiac arrest bystander**

| **What needs to change (behavioural determinants)** | **Potential intervention** | **Intervention function** | **Policy category** | **Behaviour change techniques** | **Mode of delivery** |
| --- | --- | --- | --- | --- | --- |
| **Physical capability**  The bystander can physically retrieve and operate the drone-delivered defibrillator despite feeling tired from delivering Cardiopulmonary Resuscitation (CPR). | The call-handler provides the bystander with clear instructions on how to change responders to manage bystander fatigue. | **Enablement** | **Guidelines**:  incorporating recommendations about when and how to change compressor quickly and efficiently into the call-handler protocol for cardiac arrests. | **Conserving mental resources.**  **Social support (practical).** | The call-handler delivers information over the phone if the bystander mentions they are feeling tired. |
| **Physical capability**  The bystander must be safe while using the defibrillator. | The call-handler emphasises defibrillator safety practices. | **Enablement** | **Guidelines**:  incorporating recommendations about how to operate the defibrillator safely into the call-handler protocol for cardiac arrests. | **Social support (practical).** | The call-handler delivers information over the phone at the point of defibrillation. |
| **Psychological capability**  Bystander knows how drone-delivered defibrillation works. | Incorporate information about drone-delivered defibrillation into existing training events and / or materials about how to manage a cardiac arrest. | **Training** | **Guidelines**:  adding recommendations about where drone-delivered defibrillation fits within the chain of survival and how this technology can be successfully implemented to existing training events and materials that inform the public about what to do during a cardiac arrest. | **Demonstration of the behaviour.**  **Behavioural practice / rehearsal.**  **Instruction on how to perform a behaviour.** | Print media e.g. leaflets and digital media e.g. webpages and / or face-to-face group training sessions are provided to the public. |
| **Psychological capability**  Bystander knows what will happen during the cardiac arrest response. | The call-handler prepares the bystander for what will happen during the management of a cardiac arrest by telling them that a drone will deliver a defibrillator to their location and providing information on what the drone and defibrillator will look like. | **Enablement**  **Education** | **Guidelines:**  incorporating recommendations about what will happen during the cardiac arrest response into the call-handler protocol for cardiac arrests. | **Social support (practical).**  **Prompts / cues.** | The call-handler delivers information over the phone when the bystander needs to reach the drone-delivered defibrillator during the incident. |
| **Psychological capability**  Bystander knows how to operate a drone-delivered defibrillator. | The call-handler provides clear and concise information to the bystander about how to operate the drone-delivered defibrillator. | **Enablement**  **Education** | **Guidelines**:  incorporating recommendations about how to operate the drone-delivered defibrillator or follow the defibrillator voice instructions into the call-handler protocol for cardiac arrests. | **Social support (practical).**  **Prompts / cues.**  **Conserving mental resources.** | The call-handler delivers information over the phone when required during the incident. |
| **Psychological capability**  Bystander executes multiple tasks quickly and effectively without becoming burnt out or completely overwhelmed during the incident. | Use an enhanced co-responder model (community responders, volunteer responders) to support the bystander to safely and efficiently retrieve the defibrillator from the drone. | **Enablement** | **Service provision**: establishing a co-responder model that is appropriate for drone-delivered defibrillation.  **Service provision:**  linking the drone activation system with the volunteer co-responder system (e.g. GoodSAM app) to ensure optimal use. | **Social support (practical).**  **Conserving mental resources.**  **Restructuring the physical environment.**  **Restructuring**  **the social environment.** | In-app alert that mentions drone-delivered defibrillation – e.g. ‘the defibrillator is en-route, it doesn’t need to be retrieved’ – to notify the co-responder that help is required.  Co-responder offers face-to-face support during the incident when required. |
| **Psychological capability**  Bystander executes multiple tasks quickly and effectively without becoming burnt out or completely overwhelmed during the incident. | Allow the call-hander to send brief pre-recorded video demonstrations about how to deliver CPR and / or how to access and operate the drone-delivered defibrillator to the bystander to better support them during the emergency. | **Enablement** | **Service provision**:  sending the bystander a link in a text to access the pre-recorded video demonstration(s).  **Guidelines**:  providing recommendations about how to access the pre-recorded video demonstration(s) during the incident. | **Restructuring the physical environment.**  **Social support (unspecified).**  **Conserving mental resources.** | The call-handler delivers an electronic link to the bystander when required during the incident.  The call-handler delivers information to the bystander’s mobile phone when required during the incident. |
| **Psychological capability**  Bystander executes multiple tasks quickly and effectively without becoming burnt out or completely overwhelmed during the incident. | Brief pre-recorded video demonstrations about how to deliver CPR and / or how to access and operate the drone-delivered defibrillator. | **Enablement**  **Education** | **Service provision**:  sending brief video clips about how to deliver CPR and / or how to access and operate the drone-delivered defibrillator. | **Restructuring the physical environment.**  **Prompts / cues.**  **Conserving mental resources.** | The call-handler delivers an electronic link to the bystander when required during the incident. |
| **Psychological capability**  Bystander executes multiple tasks quickly and effectively without becoming burnt out or completely overwhelmed during the incident. | The call-handler provides specific instructions about how to locate and retrieve the drone-delivered defibrillator. | **Enablement**  **Education** | **Guidelines**:  incorporating recommendations about how to locate and retrieve a drone-delivered defibrillator into the call-handler protocol for cardiac arrest. | **Social support (practical).**  **Conserving mental resources.**  **Prompts / cues.** | The call-handler delivers information over the phone when required during the incident. |
| **Psychological capability**  Bystander executes multiple tasks quickly and effectively without becoming burnt out or completely overwhelmed during the incident. | The call-handler provides specific instructions about how to operate the defibrillator that is being sent via drone. | **Enablement**  **Education** | **Guidelines**:  incorporating clear instructions about how to operate the drone-delivered defibrillator into the call-handler protocol for cardiac arrest. | **Social support (practical).**  **Conserving mental resources.**  **Prompts / cues.** | The call-handler delivers information over the phone when required during the incident. |
| **Psychological capability**  Bystander diagnoses cardiac arrest and provides necessary interventions competently during the incident. | Allow a video-enabled 999 call to facilitate cardiac arrest diagnosis and necessary interventions. | **Enablement** | **Service provision**:  sending the bystander a link in a text to access the live stream.  **Guidelines**:  providing recommendations about how to access the video live stream during the incident. | **Restructuring the physical environment.**  **Conserving mental resources.**  **Social support (unspecified).** | The call-handler delivers an electronic link when required during the incident.  The call-handler delivers information to the bystander’s mobile phone when required during the incident. |
| **Physical opportunity**  The defibrillator needs to be delivered close to the incident location. | Improve the accessibility of defibrillators by using a drone to deliver a defibrillator to the scene of an out-of-hospital cardiac arrest if a systems-level (automated) decision has deemed this action appropriate. | **Enablement**  **Environmental restructuring** | **Guidelines:**  Establishing parameters for initiating drone flight.  Introducing specific recommendations about how to locate and retrieve the drone-delivered defibrillator.  **Environmental/**  **social planning**:  planning the most appropriate place for the drone to deliver the defibrillator e.g. doorstep, garden, over the road.  **Regulation:**  Complying with Civil Aviation Authority regulations for UAVs (Unmanned Aerial Vehicles).  Complying with regulation governing the use of medical devices i.e. defibrillator transported by drone.  **Legislation:**  Complying with statutory requirements for UAV flight and carrying a payload i.e. defibrillator transported by drone. | **Social support (practical).**  **Adding objects to the**  **environment.**  **Restructuring the physical environment.** | The drone pilot and / or the drone system safely fly the drone to the correct location in accordance with regulation.  Once the drone has delivered the defibrillator, the call-handler verbally instructs the bystander over the phone to locate and retrieve it. |
| **Physical opportunity**  The box with the defibrillator inside needs to be easily identifiable. | Provision of instructions by the call-handler to explain how to locate and identify the box with the defibrillator inside. | **Enablement** | **Guidelines**:  incorporating recommendations about how to locate the box with the defibrillator inside into the call-handler protocol for cardiac arrests. | **Social support (practical).** | Once the defibrillator has arrived by drone to the incident location, the call-handler delivers information over the phone. |
| **Physical opportunity**  The bystander can easily access the defibrillator inside the box. | Ensure that the box with the defibrillator inside has a safe easy-open mechanism. | **Enablement**  **Environmental restructuring** | **Guidelines**:  incorporating recommendations about how to access the defibrillator inside the box into the call-handler protocol for cardiac arrests.  **Regulation**:  establishing voluntary agreements between manufacturers to ensure that the box containing the defibrillator is easy to access. | **Social support (practical).**  **Adding objects to the**  **environment.**  **Restructuring the physical environment.** | Once the bystander has located the box with the defibrillator inside, the call-handler verbally tells the bystander over the phone how to open it. |
| **Social opportunity**  The bystander receives sufficient and appropriate support from the emergency services throughout the process. | Support the bystander to perform life-saving actions by providing clear, relevant and concise instructions throughout the call. | **Enablement** | **Guidelines**:  introducing recommendations about how to retrieve and operate a drone-delivered defibrillator.  **Guidelines**:  developing and refining the call-handler protocol for cardiac arrests. | **Social support (practical).** | The call-handler delivers information over the phone when required during the incident. |
| **Social opportunity**  The bystander is supported by at least one other competent and cooperative bystander. | Use an enhanced co-responder model (community responders, volunteer responders) to support the bystander to safely, competently and efficiently retrieve the defibrillator from the drone.​ | **Enablement** | **Service provision**: establishing a co-responder model that is appropriate for drone-delivered defibrillation.  **Service provision**:  linking the drone activation system with the volunteer co-responder system (e.g. GoodSAM app) to ensure optimal use. | **Social support (practical).**  **Restructuring the social**  **environment.**  **Restructuring the physical environment.**  **Conserving mental resources.** | In-app alert that mentions drone-delivered defibrillation – e.g. ‘the defibrillator is en-route, it doesn’t need to be retrieved’ – to notify the co-responder that help is required.  Co-responder offers face-to-face practical support during the incident when required. |
| **Reflective motivation**  The bystander believes they can retrieve and operate a drone-delivered defibrillator safely and in a timely fashion. | The call-handler provides clear and concise information to the bystander about how to retrieve and operate the drone-delivered defibrillator.  The call-handler provides assurances to the bystander that their safety is important and has been considered. | **Enablement** | **Guidelines**:  incorporating recommendations about how to retrieve and operate the drone-delivered defibrillator into the call-handler protocol for cardiac arrests to bolster bystander confidence.  **Guidelines**:  incorporating safety recommendations into the call-handler protocol for cardiac arrests. | **Social support (practical).**  **Verbal persuasion about capability.** | The call-handler delivers information over the phone when required during the incident. |
| **Reflective motivation**  The bystander views the defibrillator as a potentially life-saving device. | The call-handler provides clear explanations for why it is important to retrieve a drone-delivered defibrillator and gives guidance on how to operate it. | **Enablement**  **Education** | **Guidelines**:  incorporating recommendations about the relevance of defibrillation and how to operate the drone-delivered defibrillator into the call-handler protocol for cardiac arrests. | **Social support (practical).**  **Verbal persuasion about capability. Framing / Reframing.**  **Prompts / cues.** | The call-handler delivers information over the phone when required during the incident. |
| **Reflective motivation**  The bystander understands the usefulness of a drone delivering a defibrillation to their location. | The call-handler provides clear explanations for why it is important to retrieve a drone-delivered defibrillator and gives guidance on how to operate it. | **Enablement**  **Education** | **Guidelines**:  incorporating recommendations about the relevance of defibrillation, where to find the device and how to operate the drone-delivered defibrillator into the call-handler protocol for cardiac arrests. | **Social support (practical).**  **Verbal persuasion about capability. Framing / Reframing.**  **Prompts / cues.** | The call-handler delivers information over the phone when required during the incident. |
| **Reflective motivation**  The bystander believes that they are permitted to retrieve and operate a drone-delivered defibrillator. | The call-handler provides clear and concise information about how to locate, retrieve and operate the drone-delivered defibrillator including informing them that they are permitted to use it. | **Enablement**  **Education** | **Guidelines**:  incorporating recommendations about how to locate, retrieve and operate the drone-delivered defibrillator into the call-handler protocol for cardiac arrests to bolster bystander confidence. | **Social support (practical).**  **Framing / Reframing.**  **Verbal persuasion**  **about capability.**  **Prompts / cues.** | The call-handler delivers information over the phone when the bystander needs to retrieve the defibrillator during the incident. |
| **Reflective motivation**  The bystander must be willing to leave the patient to retrieve the defibrillator from a drone. | Carefully worded call-handler protocol for cardiac arrests to encourage the bystander to leave the patient to retrieve the drone-delivered defibrillator if the bystander is reluctant to go. | **Persuasion** | **Guidelines**:  providing clear explanations for why it is important that the bystander leaves the patient to retrieve the drone-delivered defibrillator if they are reluctant to go. | **Verbal persuasion about capability.**  **Framing / Reframing.**  **Credible source.** | The call-handler delivers information over the phone if the bystander is reluctant to leave the patient’s side to retrieve the drone-delivered defibrillator during the incident. |
| **Reflective motivation**  The bystander wants to help the patient by getting the drone-delivered defibrillator. | Carefully worded call-handler protocol for cardiac arrests to encourage the bystander to leave the patient to retrieve the drone-delivered defibrillator if the bystander is reluctant to go. | **Persuasion** | **Guidelines**:  introducing a clear and concise set of explanations for why it is important for the bystander to leave the patient to retrieve the drone-delivered defibrillator if they are reluctant to go into the call-handler protocol for cardiac arrests. | **Verbal persuasion**  **about capability.**  **Framing / Reframing.**  **Credible source.** | The call-handler delivers information over the phone if the bystander is reluctant to leave the patient’s side to retrieve the drone-delivered defibrillator during the incident. |
| **Reflective motivation**  The bystander believes that the call-handler has the time and capacity to provide clear and appropriate advice until the ambulance service arrives. This advice needs to:   1. relate to the management of the cardiac arrest; and, 2. prompt the bystander to perform basic non-medical tasks e.g. take their phone with them, leave the door on the latch before going outside, put on a coat, take a torch, etc. | Carefully worded call-handler protocol that focuses on helping the bystander to navigate the management of the cardiac arrest process quickly and effectively, including providing practical advice to support them with the retrieval of the drone-delivered defibrillator. | **Enablement**  **Education** | **Guidelines**:  incorporating recommendations about how the bystander can be best supported when getting the defibrillator into the call-handler protocol for cardiac arrests. | **Social support (practical).**  **Prompts / cues.**  **Verbal persuasion about capability.** | The call-handler delivers information over the phone when the bystander needs to reach the defibrillator during the incident. |
| **Automatic motivation**  The bystander manages negative emotions (anxiety, fear, stress) surrounding the management of the cardiac arrest. | Inclusion of emotionally supportive statements in the call-handler protocol. | **Enablement** | **Guidelines**:  incorporating empathy and emotionally supportive advice into the call-handler protocol for cardiac arrests. | **Social support (emotional).**  **Reduce negative**  **emotions*.*** | The call-handler delivers information over the phone if the bystander becomes distressed during the incident. |
| **Automatic motivation**  The bystander is praised while they are managing the cardiac arrest. | Providing bystanders with positive reinforcement and feedback during the call. | **Incentivisation** | **Guidelines**:  incorporating positive reinforcement for the bystander’s involvement into the call-handler protocol for cardiac arrests. | **Social reward.**  **Feedback on behaviour.** | The call-handler delivers information over the phone at appropriate times during the incident. |
