# Supplementary material for: Bystanders’ attitudes towards drone delivered Automated External Defibrillators for out-of-hospital cardiac arrest: A qualitative interview study
Source: PLoS One. 2025 Dec 3;20(12):e0337082. doi: 10.1371/journal.pone.0337082 (PMC12674532; doi:10.1371/journal.pone.0337082)
Supplement: S1 File — (DOCX) [file pone.0337082.s003.docx]

# **S1 File. Topic guide.**

**Introduction / preamble**

*Initiate call. Recording has NOT started at this point.*

[Greeting]

“This is Dr Celia Bernstein, senior research fellow, from the University of Warwick. [pause, they may acknowledge]. Hopefully you’re expecting my call about the Drone-Delivered Defibrillators research study.” *Participant confirms.*

“Just to make sure I am talking to the right person, could I confirm your full name please?”

“Thank you. This interview is going to be audio-recorded, and I am going to start the recording now if that is okay with you?” *[Pause, allow time for response/objection/clarification]*

*Recording has started:*

“Okay, the audio-recording has begun.”

“This study forms part of a research study being completed by The University of Warwick and Welsh Ambulance Service NHS Trust.”

“Before we can proceed with the interview, I need to re-confirm your eligibility. Can you confirm that you are aged 18 or over *[pause for answer]*; that you are comfortable to proceed with the interview in English *[pause for answer]*; that you have provided assistance at a cardiac arrest at some point in the past *[pause for answer]*; that you agree to have an audio recording made of your interview” *[pause for answer]*.

“Thank you for confirming that.”

“The interview will last for around 45 minutes, and your participation is entirely voluntary. You can pause, reschedule or terminate the interview at any time, without providing a reason and without your rights being affected. *[For participants being interviewed via Teams]* You can also choose to have your camera on or off. There are no right or wrong answers; we are interested in your experiences of helping someone who had a cardiac arrest, and your attitudes around using drones to deliver defibrillators.”

“I will need to formally record your consent to participate in the interview by reading out each of the statements in the consent form that you received previously and asking you to answer ‘yes’ or ‘no’ to each question.

“Before I do that, do you have any further questions about the study or about the interview process.

*[Participant responds.]*

*[Questions answered.]*

*[Reads out the consent form statements verbatim**. Explains that the final question is optional, interview can proceed if the answer is ‘no’]*

“I will now stop this recording and start another recording of your answers to the main interview.”

*[End first recording.]*

*[Start second recording.]*

“Okay, the main interview audio-recording has begun, and it will continue until the interview questions end.”

**Main questioning**

*Opening question*

“Can you tell me as much as you can about when you provided assistance to the person who had a cardiac arrest?”

- Where did the cardiac arrest happen (public, at home etc.)?
- Who was the person who had a cardiac arrest (what is their relationship to you)?
- Did you realise what was happening at first? How did you realise it was a cardiac arrest? How soon afterwards did you realise it was a cardiac arrest?
- What was the outcome? (If on a stranger, be aware that the participant might not know).
- Did you perform cardiopulmonary resuscitation or CPR?
- Did you have immediate access to a defibrillator? (May need to probe other terms to gauge understanding: “AED” or “Automated External Defibrillator”).
- Were you by yourself or were there other people there? How did that make you feel?
- If other people were there, what did they do?
- If other people were there, how did they organise themselves?

*Areas of interest (with probing questions)*

**Interaction with call-handler**

“Can you tell me as much as you can about the 999 call?”

*It may be that someone else made the call, the questions below are likely still to be relevant in this case (can clarify whether the call was put on speakerphone so that the participant could hear the instructions directly, or if the instructions were relayed to them).*

- Did you (or somebody else) use a landline or mobile to speak to the 999 call-handler?
- Did you have to leave the patient to make a call?
- Can you remember what the 999 call-handler said / what help did the 999 call-handler offer?
- What questions did the 999 call-handler ask you (or the person making the call)? Did you ask the call-handler any questions? If so, what questions did you ask the 999 call-handler?
- What are your reflections on the interaction: what went well, what was difficult?
- Were there any technical issues while you (or somebody else) were speaking to the 999 call-handler on the phone? If so, what were these?

**Performing CPR**

“Can you tell me as much as you can about performing CPR?”

- Did you do chest compressions only?
- Did you do chest compressions with ventilations / rescue breaths?
- What are your reflections on how that went?
- Did the 999 call-handler offer or give you instructions?
- What are your reflections on the instructions / advice given?

**Availability of a defibrillator (AED)**

“Did you use a defibrillator?”

***If a defibrillator was used***

“Can you tell me as much as you can about using the defibrillator?”

- How easy was it to use and understand?
- How comfortable did you feel using it?
- How easy was it to follow the defibrillator voice instructions?
- Did the 999 call-handler offer any instructions over the phone about using the defibrillator? If so, how did you find following the defibrillator and 999 call-handler instructions at the same time?
- Was the defibrillator already there? If so, was it ‘on-site’ or had someone else brought it?
- Was the fact that there was a defibrillator nearby mentioned by the 999 call-handler?
- Did you go to get it yourself? How far did you have to go? How did you feel about this?

***If a defibrillator was not used***

“What are your thoughts on why a defibrillator was not used?”

- Was there a defibrillator there (or was one brought to or retrieved by you) that wasn’t used? What were the reasons for that?
- Was there a defibrillator that you were aware of (at the time) that you didn’t go and get? What were the reasons for that?
- Did you subsequently realise that there was a nearby defibrillator that you hadn’t remembered at the time?
- Had the 999 call-handler advised you of a nearby defibrillator?

“What are your thoughts about using a defibrillator in the future?”

“Is there something that would make it easier for you to use a defibrillator in the future?”

**Drones delivering defibrillators**

“Is drone delivery of defibrillators something that you’ve heard of?”

- Have you heard of drones?
- Have you seen drones used or used one yourself and in what situation?
- How do you imagine drone-delivered defibrillation works?
- How does the defibrillator get safely to the ground?
- How does one go about getting the defibrillator from the drone?

“How would you feel about a drone bringing a defibrillator to you whilst helping someone having a cardiac arrest?”

- Would it make it easier or more difficult for you to use a defibrillator? How?
- How do you feel about leaving the person if they are by themselves?
- How far would you be willing to go to get the defibrillator that had just been delivered?
- Would location (public vs home, indoor vs outdoor) affect your thinking on this?
- What do you think the role of the 999 call-handler is in all of this?
- What would you want to know from the 999 call-handler about drone-delivered defibrillator? That one was coming? Only when it was there? Information on identifying it?
- What concerns, if any, do you have about this?
- What problems or issues, if any, do you foresee?

**Wrapping up**

“Thank you. I have some final brief questions that may be useful for our research.”

“What is your age and gender?”

“Have you had training in CPR and defibrillator use?” It may be that this has come up during the interview but, in any case, clarify:

- When this was (If unsure, just ask whether or not it was in the last 5 years).
- Whether it was CPR only, or CPR and defibrillation.
- Whether it occurred before or after the incident being discussed.

“Is there something else you would like to add to your answers today?”

**At the end of the interview**

*Recording has finished.*

“Thank you for taking part in the interview today.”

“I have now switched off the audio recorder(s) and will briefly explain what will happen next. As mentioned in your Participant Information Sheet, a transcript will be made from your recording so that it can be analysed for any recurring themes and patterns.”

[*If participant agrees to receive a summary]: “*Earlier you agreed to receive a summary of the study. Could I check that this is the email address you would like us to send the summary to” [read out email address *to participant and wait for their response*]?

“Before we finish, do you have any further questions about the research or your participation within it?” [*Wait for participant to respond*].

“Thank you again for your time today.”

**Issues that may arise**

*Clinical Questions*

Do not offer clinical advice and do not suggest a course of action for next time. If pressed, be clear that it is not your role to do this. You can suggest that they access the webpages of Resuscitation Council UK (<https:///www.resus.org.uk>), who provide guidelines and information about a number of topics related to CPR and defibrillator use.

*Concerns for Welfare of Interview Participant*

If there are concerns about the participant and you provide assistance, you are acting in a Good Samaritan capacity.

If there are **immediate** concerns about the safety of the patient, see the Sensitive Call Action card, but:

- Confirm address if possible.
- Call 999 – it is likely you will be put through to the West Midlands Ambulance Service switchboard. If the participant is in a different region, you should specify that your call is related to someone out-of-area.
- Contact a senior member of staff from Warwick Clinical Trials Unit, according to existing University safety procedures. Notify the participant of this first.

If you do not have concerns about safety but think they may require support, the following sources may be useful (and you can remind the participant that they are in the Participant Information Sheet):

- Your own GP
- NHS 111
- Samaritans: 116 123 (from any phone)

<https://www.samaritans.org/how-we-can-help/>

- Mind: 0300 123 3393 (phone); 86463 (text)

<https://www.mind.org.uk/need-urgent-help/using-this-tool/>

- Sudden Cardiac Arrest UK

<https://www.suddencardiacarrestuk.org/get-support/>

- Bystander Support Network

<https://www.bystandernetwork.org/>

- Heartsight

<https://ourheartsight.com/>

**ITERATIVE INTERVIEW QUESTIONS (DEVELOPED DURING THE PROCESS OF DATA COLLECTION)**

Eligibility question (only eligible if recruited via WAST or cardiac arrest survivor charity): how did you hear about us?

1. *If bystander has attended multiple arrests:* Tell me about the time you assisted with a cardiac arrest in a lay or off-duty capacity.
2. *If relevant*: How did you feel about leaving the patient to let the ambulance crew in?
3. *If bystander has a medical background and/or has attended multiple cardiac arrests:* Please tell me about any other (off-duty) experiences of managing a cardiac arrest.
4. *If bystander has attended multiple arrests:* Why did you choose to discuss this / these particular incident(s) today?
5. Drones are small flying aircraft that are piloted remotely. They have camera capabilities so they can navigate and see where to go. They can carry defibrillators by landing and detaching the device or they can hover above the ground and winch the device down to the ground. Can you imagine interacting with a defibrillator in either of these ways?
6. Ask these questions after ‘*how far would you be willing to go to get the defibrillator’* topic guide question: How close would you want the defibrillator to be delivered to you? How would you feel about getting the defibrillator if it was delivered to you by drone? For example, if a cardiac arrest occurred at home, would you be prepared to leave the patient to get the defibrillator from the front door or would you be prepared to cross the road to get it?
7. Ask these questions after *‘what would you want to know from the call-handler’* topic guide question: Would you be willing to leave the patient’s side to retrieve the drone-delivered defibrillator if the call handler instructed you to do so? *Explore their thoughts on this*. *For example,* w*ould their thought processes around leaving or staying with the patient change?* *Do bystanders realise that the call-handler can tell them when to leave?*
8. Would you want the call-handler to give you advice about when to leave the patient? Would you find it useful? Why/why not?
9. How would you manage to get the defibrillator from the drone if others were around? For example, would the second bystander get the defibrillator while you performed CPR (or vice versa)?
