# Supplementary material for: Bystanders’ attitudes towards drone delivered Automated External Defibrillators for out-of-hospital cardiac arrest: A qualitative interview study
Source: PLoS One. 2025 Dec 3;20(12):e0337082. doi: 10.1371/journal.pone.0337082 (PMC12674532; doi:10.1371/journal.pone.0337082)
Supplement: S2 File — (DOCX) [file pone.0337082.s004.docx]

# **S2 File: Iterative interview questions (developed during the process of data collection)**

Eligibility question (only eligible if recruited via WAST or cardiac arrest survivor charity): how did you hear about us?

1. *If bystander has attended multiple arrests:* Tell me about the time you assisted with a cardiac arrest in a lay or off-duty capacity.
2. *If relevant*: How did you feel about leaving the patient to let the ambulance crew in?
3. *If bystander has a medical background and/or has attended multiple cardiac arrests:* Please tell me about any other (off-duty) experiences of managing a cardiac arrest.
4. *If bystander has attended multiple arrests:* Why did you choose to discuss this / these particular incident(s) today?
5. Drones are small flying aircraft that are piloted remotely. They have camera capabilities so they can navigate and see where to go. They can carry defibrillators by landing and detaching the device or they can hover above the ground and winch the device down to the ground. Can you imagine interacting with a defibrillator in either of these ways?
6. Ask these questions after ‘*how far would you be willing to go to get the defibrillator’* topic guide question: How close would you want the defibrillator to be delivered to you? How would you feel about getting the defibrillator if it was delivered to you by drone? For example, if a cardiac arrest occurred at home, would you be prepared to leave the patient to get the defibrillator from the front door or would you be prepared to cross the road to get it?
7. Ask these questions after *‘what would you want to know from the call-handler’* topic guide question: Would you be willing to leave the patient’s side to retrieve the drone-delivered defibrillator if the call handler instructed you to do so? *Explore their thoughts on this*. *For example,* w*ould their thought processes around leaving or staying with the patient change?* *Do bystanders realise that the call-handler can tell them when to leave?*
8. Would you want the call-handler to give you advice about when to leave the patient? Would you find it useful? Why/why not?
9. How would you manage to get the defibrillator from the drone if others were around? For example, would the second bystander get the defibrillator while you performed CPR (or vice versa)?
