# Supplementary material for: Bystanders’ attitudes towards drone delivered Automated External Defibrillators for out-of-hospital cardiac arrest: A qualitative interview study
Source: PLoS One. 2025 Dec 3;20(12):e0337082. doi: 10.1371/journal.pone.0337082 (PMC12674532; doi:10.1371/journal.pone.0337082)
Supplement: S3 File — (DOCX) [file pone.0337082.s005.docx]

# **S3 File. Anonymised interview transcripts**

| INTERVIEWEE: | R |  |  |
| --- | --- | --- | --- |
| INTERVIEWER: | I |  |  |

Transcripts have been anonymised, and other small redactions have been made to minimise risks of inadvertent reidentification. All such redactions are indicated in square brackets e.g. [name of location].

Participant 1

I:  Okay. So, the audio recording has begun, and it will continue until the interview questions end. Thank you for agreeing to participate in this interview, which forms part of a research study being completed by the University of Warwick and Welsh Ambulance Service NHS Trust. Before we can proceed with the interview, I need you to reconfirm your eligibility. So, can you confirm that you are aged 18 or over?

R: Yes, I confirm.

I: That you are comfortable to proceed with the interview in English?

R: Yes.

I: That you have provided assistance at a cardiac arrest, at some point in the past.

R: Yes, I have.

I: And that you agree to have an audio recording made of your interview.

00:01:04

R: Yes, sure.

I: Thank you. So, I've received a signed consent form from you. Has anything changed since you signed the consent form?

R: (Pause) Sorry? Can you come again?

I: Sorry. Has anything changed since you signed the consent form?

R: No.

I: Thank you for confirming that. So, the interview will last for around 45 minutes, and your participation is entirely voluntary. So, you can pause, reschedule, or terminate the interview at any time, without providing a reason, and without your rights being affected. And you can also choose to have your camera on or off. There are no right or wrong answers. We are interested in your experiences of helping someone who had a cardiac arrest, and your attitudes around using drones to deliver defibrillators. Do you have any further questions about the study, or about the interview process before we begin?

R: You can just continue, if any comes to my (?) mind, I'll let you know.

I: Excellent, that's absolutely fine. So, are you okay to start?

R: Yes, sure.

I: And like you say, if you do have any questions, please feel free to stop me. Or if you want to email me after the interview, please feel free to do that as well. Okay. So, can you tell me as much as you can about when you provided assistance to the person who had a cardiac arrest?

R: Yeah. It, it, it just happened so suddenly. And actually, I was bit, I was scared, because I, I don't know if I, I would be able to do it well right. It was in work, after work. So, a, a colleague of mine suddenly slumped. It, it, it was just frustrating, and, and the, actually, we have to call for 911, but we, we weren't, we don't know the time it might (inaudible) and the arrival of that. And it, it just, I, I decided to intercede, because I, I know about it being a lifesaving technique. So, so that was when, that was how I, I, I have to gather up courage, to be able to, to do that. But I, I wasn't too sure at first, but I can't just watch him die, or I, I, I, we can't just wait. We don't know how, how many minutes it might take for the ambulance to, for the emergency people to, to come over. So, so that was how I decided to do it proceedings.

I: Okay. So, you said it happened to a colleague. So, did this happen at work, then?

R: Yeah, it, it was just during the evening period, when we, we've dismissed already. So, for, for some reason, I, I don't know, but when I, when I have to do some research to (inaudible) I, I found out that he, he was, he was also smoking, and doing something that, that might cause that suddenly. So, so, so, that was how, how it, it was.

I: And how did you realise what was happening at first?

R: (Pause) Sorry?

I: How did you know that it was a cardiac arrest?

R: Yeah. Yeah. Yeah, it, it was the way, he just slumped over. And (inaudible) I have (?) a little experience of that, from, from, when, if, if my GP had just told me about more of those, so I, I have to just, even my GP had told me if, what I should do. So, he told me that if I can be confident enough to proceed with that, that I should give it a try, because even before he'll be able to come, it might be a lot more later. That was where I, I decided to cough up courage to do it.

00:05:16

I: Okay. And so, what did you think was happening at first, when you saw this person collapse? Or slumping, as you say. What, what, you know, what were, what was going through your mind?

R: Yeah, yeah. Actually, it, there were a, a lot of things were, were (inaudible) to mind (?) I was, like, "What might cause this?" At first, firstly, I have to pick up my phone to, to check on, on (inaudible) what might cause a person to slump, all, all of a sudden, with, without any, any much of a, of a sign. So, although we were, we were up to seven, or, or eight that was present at that time. So, I have to, may (inaudible) it, and come with... I have to let my GP know, now (?) tell me if I can push it further to, to do that, it might be, it might be helpful.

I: And what was the outcome for the person?

R: Yeah. Yeah. Absolutely (inaudible) extra. At least I was able to, to, to manage it before the ambulance would came, came over. But I, I, I am, I'm just scared I might not be able to give (inaudible)

I: Did you perform CPR?

R: Yeah. Yeah. I, I, I, I did.

I: Okay. And did you have immediate access to a defibrillator?

R: Sorry? What, what do you say?

I: Sorry. Did you have immediate access to a defibrillator? You might know this as an AED, or automated external defibrillator.

R: Yes.

I: You had access to a defibrillator, then? (Pause) Okay. And were you by yourself, or were there other people there?

R: Yeah. Yeah. There were, there were other people there. We were, a number of seven, if I'm not mistaken.

I: And how did that make you feel?

R: Yeah, yeah. At first, it was, to, to start, it was, "What, what if some, something goes wrong?" So, so, that was, that was what was going through my mind (?), or, or, or what are the cost of doing that (inaudible) the person, just, just go and I was like, actually, I don't want to involve myself in, in, in anything concerning life. So, that was what was, that's (?) going through my mind, but I can't just watch him die. I can't just watch someone die (inaudible) in front (?). And me having a little knowledge about this CPR lifesaving technique. So, I, actually, that was the first time of me performing that. So, I just have to cough up courage to, to, to do that. That's, that what, how I was feeling, and how scared I was.

I: And what did these other people do?

R: Yeah, yeah. It, it felt, also, there was present there, it was (?) "Are, are you sure you can do this? You're not a, a doctor, are you. (?) How, how, how sure are you? You shouldn't make the case a, a lot more complicated." But I was say, "Don't worry. I can, I can just push, and see how far. Because we're, we're, we're just running out of time. We just, something needs to be done." So, the, my, the other friend to say, "Okay, if, if he, if he knows something, he can perform this, he can just go straight away, before the ambulance would come, come in." Just instead of me just standing and, and, and watching. So, so, everyone would bring out their own different opinion about the case, before I decide to proceed.

00:09:02

I: And how did that make you feel, all these different opinions flying around?

R: Yeah, actually it, it, it, it was a (inaudible) order to bring down my, my morale to do it, but, but a, a, a, a friend also there, of mine too that was present, have to tell me to, that if I can, I, I, I (?) should help, (inaudible) try. Even if he, you know, might not make it, but I cannot give it a try that he, he blame it on me. So, I, I, I, I just can't stand, stand being idle, so, so that was how it is. But if, if I, I, I might have had a lot more of, of, of negative words coming through, I, I, I wouldn't have.

I: Okay. And can you tell me as much as you can about the 999 call?

R: Sorry?

I: Can you tell me as much as you can about the 999 call? So, did you make the 999 call, or did somebody else make the call?

R: Yeah, it was somebody else that, that, that placed that, that made the call.

I: Okay. And did they use a landline, or a mobile to speak to the 999 call handler?

R: It is, it is a mobile device that he used to make (inaudible) call.

I: Okay. And was the call put on speakerphone, so that you could hear the instructions? Or were these ...

R: Yeah.

I: ... Yeah.

R: There were, they, he have to put it on, on loudspeaker, just for me to be able to hear how, the direction or how to, to go about it.

I: Okay. And did you have to leave the patient to make the call?

R: Sorry?

I: Did you, or the person making the call, have to leave the patient?

R: No, no, no. It, it (inaudible) me.

I: You stayed with the patient.

R: Yes.

I: Yeah. Okay. And can you remember what the 999 call handler said, or what help they offered?

R: Yeah. They, they directed me about some, some direction on, on how to, to, to, to perform the CPR.

I: Right. Okay. So, you got guidance on, on how to perform the chest compressions. And did they say ...

R: Yes.

I: ... anything else to you?

R: They, they just have, they just have to encourage me at first, because they, they (inaudible) that they know I was, I'm scared to do that, but told me I, I, I, I can do it. So, I, I had to just manage myself, put myself together to, to do it. So, those, those, those more encouraging words were coming from them that, that, that helped give me the, the, the strong faith that I should proceed, and that, and that I would be successful. So, so, those words too that, that came, also motivated me to proceed.

00:12:08

I: And what questions did the 999 call handler ask you, or the person making the call?

R: Sorry, I, I don't get it.

I: Can you remember if the call handler asked you any questions? Or asked the person who had phoned the call handler any questions?

R: Yeah. Yeah, yeah. (inaudible) the, the, the question that (inaudible) were just coming to at the time. What, what, where, where, where our position was, and who is the person. If, if anybody there's close to the person, or a family member, close, just have, know the, the history of, or what he had been doing before then, that might cause that sudden, or, or activity that he was doing before, what (inaudible) that, that was more the question that was coming in, and, and the, the one (inaudible) just giving them feedback and answer to those questions.

I: Okay. And did you ask the call handler any questions?

R: Actually, I, I, I, I didn't (inaudible) ask. I just (inaudible) stay by me, and, and, and just let, let me go through, through the tests, actually.

I: Okay. And what are your reflections on the interaction with the call handler? So, how do you think ...

R: Yeah. Yeah.

I: ... it went?

R: Yeah, yeah. They, they, they, they better (inaudible) what, what someone's (inaudible) in, in mind, or tell (?) that we wouldn't want to (inaudible) ourselves. So, so, that's how (?) the person was, so yeah, the person wanted to give me this, this vibe of stopping, he would just want me to do (inaudible) everywhere at the person, or being scared, because I was not getting the, the, the feedback which, which, which was to have emerged. So, so, the person would say I should just go on to, go on, and that's how it, it, it was.

I: And what went well, would you say?

R: (Pause) Yeah, yeah, at, at, what went well the person was, was unresponsive, and, and, and not breathing even after the airway tip, tipping, tipping (inaudible) so it was, how scared I, I was. So, when the person wasn't responding, so I was, I wanted to just stop everything, all the process, but the, the person was, he gave me some, some more courage to, to, to keep practising it, and look if there would be some (inaudible)

I: And what did you think was difficult about the interaction? Was anything difficult?

R: Actually, it, there, there wasn't any, lot (inaudible) difficult, in terms of the interaction.

I: Okay. And were there any technical issues while you or the other person was speaking to the call handler on the phone?

R: Sorry?

I: Did you experience any technical issues with talking to the call handler on the phone?

R: Yeah, (inaudible) did, there were, there were some times (inaudible) was being (inaudible) I was being blanked about, because that's, that's not in my field (?). So, I was like, "Okay, can you just break that down, so, so I can be able to get." So, that was how it went.

00:15:36

I: Right. So, you had to put the phone down, is that what you mean?

R: No, there, there were, there were some times that (inaudible) was being (inaudible), some, sometimes that in the medical field, I was, it was (inaudible), that I wanted a lot more idea about. So, I have to let him, explain that down, so I can, able to get the point.

I: Okay. And can you tell me as much as you can about performing CPR?

R: Sorry?

I: Can you tell me as much as you can about performing CPR, or cardiopulmonary ...

R: Yeah, yeah ...

I: ... resuscitation? Yeah.

R: Yeah. Yeah. Cardiopulmonary resuscitation, it just mean like, I know it about, it's being a lifesaving technique that, that help maintain blood circulation, and oxygenation to the brain, and, and maybe other vital organs, where someone heart just suddenly stopped beating, or it might not be beating effectively.

I: Okay. And did you do chest compressions only?

R: Yeah, there was, there was some, some, some, the way I was, been taught, to place on my hands, and, and tip, tip them, so I, I have to do that, that was what, actual I did.

I: Okay. And did you also do chest compressions with ventilations, or rescue breaths?

R: Sorry, can you come again?

I: Yes. Did you do the chest compressions with ventilations, or rescue breaths?

R: Yeah. Doing, when, before we proceed with that, the, the, 911 (inaudible) was, wasn’t around (?) so they have to do that (inaudible) instead (inaudible)

I: Okay. So, so, you did do the ventilations as well as the chest compressions?

R: Yeah. I was, I, I was (inaudible)

I: Okay. And what are your reflections on, on how you performed the CPR, on how it went?

R: Sorry?

I: Sorry. What are your reflections on how the CPR went? On how you performed the CPR? How would you say ...

R: Yeah, I ...

I: ... it went?

R: I, I, I, I wouldn't say it, it, it went too smoothly, because I, I, I was bit scared of the (inaudible) but I, I (inaudible) think I, I did to the best of, of which I know I can.

I: And did the call handler give you instructions? I, I think you said they were reassuring you. Did they give you any instructions about how to do the CPR? I think you said they did. Is that correct?

R: Yes.

I: Yeah, okay. So, can you just go into a little bit more detail about what instructions they gave you?

00:18:47

R: Yeah, the, the, the first thing was, was to take the person, airway, put him (inaudible). So, they told me about the positioning, how to position the person on, on their back on a firm flat surface. So, that one thing. And, and also, they told me about tipping, tipping their head back, and lifting their chin to open the airway. They told me about how I, I, I should, I should listen to, to, to the breath sound, and, and feel, feeling for air, and, and, and the check (?)... They told me about also, like, checking, yeah, checking for the pulse, for, for the pulse. And (inaudible) they told me to check if the person responding or, or not. Yeah. So, those things, (inaudible) them to check person airway, positioning, the tipping, the listening, and taking of the pulse.

I: Excellent. And what did you think about the instructions and advice that you were given?

R: Yeah, yeah, yeah. Actually, it, it, it, it, it guided me in the, the whole, whole lot of this process, because I, I might be (inaudible) enough, but if, if I, I wouldn't have got some additional things (?), I, I, I wouldn't have, have done that the, the, the way I did it.

I: Yeah. So, they helped you to perform the chest compressions in the way that you did?

R: Yes.

I: Yeah. You, you wouldn't have known otherwise, is that correct?

R: Yeah.

I: Okay. So, you said that there was a defibrillator around. Did you use the defibrillator? (Pause) The defibrillator, did you use it at all?

R: (Pause) I, I don't, I didn't actually use the defibrillator (?) but I would just be told about, about what it was meant for, and the, the automatic (inaudible) defibrillator.

I: Okay. So, did you ... Sorry, so, did you use one? Or ...

R: I was, I was just bit scared to, to, to, to make use of that, but I didn't actually use that.

I: Okay. So, you didn't use one. Okay, that's fine. So, what are your thoughts on why a defibrillator was not used?

R: (Pause) Okay, actually, I, I, I might not be too, too, too knowledgeable about that. But what has been (inaudible) is (inaudible) electronic device that automatically (inaudible) and, and more of those. So, I was just scared to, the whole process is, the pressure was much on me, so I was just (inaudible) to proceed and how to go about it. So, that, that was how, how it was on me. It would be a lot more good if, if there were maybe some, someone in the medical field that was present, that maybe I’m just assisting, and, and I would be a lot more better. But now, I'm just on my own, so doing all of that, something that I'm doing for the first time with a, a lot more people looking, looking at me. So, that was how scared I, I was.

I: Yeah.

R: What (?) my, my, my thought was, "Well, what if something go wrong?"

I: Yeah.

R: So, I, I, that's why I was just, even during the time I was, I, I was doing the tipping, and checking the pulse, but no responding, I was a lot more scared that maybe I just wasted a whole lot of my time doing, doing nothing, because (inaudible) I was seeing no response. So, so, that, that was how it was.

00:22:34

I: Yeah. Yeah. You were concerned that if you used a defibrillator, that would take time away from performing chest compressions on the patient, and helping the patient. Is that correct?

R: Yes.

I: Yeah. Okay. So, there was a, so, there was a defibrillator nearby. Was that, was one brought to you, or retrieved by you? I know you didn't use one, but was one brought to you, or retrieved by you?

R: (Pause). Actually, I was just been to that (?) that there's, there's one nearby, but I, I didn't, didn't let them bring it over to me. So, I was just knowledge that, yeah, there's a defibrillator automatic external defibrillator nearby. So, I was like, "So, what might that be used for?" Even during, when I was doing the calling, there was the, they talk about that too. But I, I say, "I don't know how to make use of that." So, they, they say, "Okay, fine." I should just go with the, with the chest compression that I'm, I'm, I'm doing. So, that was how it was. It wasn't actually present in the scene, but it was nearby, according to what I was been told.

I: Did the call handler advise you of a nearby defibrillator?

R: Mmm hmm.

I: Did they, they, they told you where one was?

R: Yeah.

I: Yeah. Okay. And then, sorry, did you say one was brought to you, or retrieved by you, or not?

R: No, (inaudible) but I, they didn't actually bring it (emphasis) to the scene.

I: And what are your thoughts about using a defibrillator in the future?

R: Maybe, that, that would be, if, if, if I become more knowledgeable, then I can, I can give it a try. But for, for, for, for, for now, I, I, I wouldn't make use of that. Mostly if, if it's an, an urgent case, with time not on your side.

I: Is there something that would make it easier for you to use a defibrillator in the future?

R: Actually, if, if I know about how I can use it, without being time consuming, so someone can just, just let us know, since it's available, there are some things that I should be available, that knowledgeable about in case of urgency. Even making (inaudible) so we went to the bar to make sure that, so, that, that, that's for urgent people. So, if you have been given some light (inaudible) about those things, then I think it will help us (inaudible) it (inaudible)

I: Okay. And just moving on to your attitudes around drone delivered defibrillators. Firstly, is drone delivery of defibrillators something that you've heard of?

R: (Pause). Sorry, can, can, can you come again?

I: Yes. So, have you heard of defibrillators being delivered by drone?

R: Yes. Yes. I've just heard about this.

I: What, what, what have you heard then, in particular?

R: I'm having difficulty hearing you. Can, sorry, can, can you just come again?

I: So, what have you heard about defibrillators being delivered by drones? Where have you heard about it?

00:26:27

R: Yeah. Yeah. I, I, I've heard about that, delivering defibrillator where journey is a, a (inaudible) But it's questions consider, the, the, the (inaudible) and, and the, and the disadvantages too. Like, the, like, the weather conditions, and, and more. More like navigating, and location accuracy. So, we have been (inaudible) about some response time, and, and also it might need some training and instruction, just, just, just to enable the recipient know how to make use of that correctly. And we have also know about the, the public acceptance to drones, concerning privacy, noise, and safety. So, so, we, we (inaudible) just how the (inaudible) about the merits of it, and how it might be used, and how it, it should be kept safe.

I: Okay. And have you heard of drones? (Pause) Have you heard of drones?

R: I've, I've not heard about it alone, but I've just heard about the delivering of defibrillator via drones, but to, to hear about a singular drone, I've, I've not really do.

I: And how do you imagine it would work? How would you imagine defibrillators being delivered by drones would work?

R: (Pause). Sorry. Sorry, can you come again?

I: How, how do you think a drone might deliver a defibrillator?

R: Actually, I, I would (inaudible) about more, of the, the amount that you, I haven't read that into to know how, how it might be delivered. But I just know (inaudible) but signals could delay, or, or prevent delivery. And malfunctioning, and the battery failure, the loss of signal could, could delay, or prevent delivery. But I, I, I don't really know if, if there are a lot more process to deliver the drone.

I: Okay, and how do you think the defibrillator would get safely to the ground?

R: Maybe if the, the, we be more, more, more, like, how should I say? Yeah (?), how it can be kept (inaudible) just to keep it safely (inaudible)

I: And how would you go about getting the defibrillator from the drone? (Pause) How do you think one would go about getting the defibrillator from the drone? (Pause) It's okay if you're not sure.

R: Yeah, I'm, I'm, I'm just thinking. I'm just thinking. If something comes to, to, to my mind. (Pause). Yeah, I can't really think of something, I, I haven't come across that, that, that, that instances. But if it comes to mind, I'll let you know.

I: Yeah, sure. That's fine. And how would you feel about a drone bringing you a defibrillator while you were helping someone who was having a cardiac arrest?

R: Yeah, yeah. I think if, if the, if drone come with, with more, more like someone for (?), that, that can direct me, or (inaudible) maybe if there's a manual, although there might not be a lot of time to be able to dive into that, to check about it so quickly. But maybe, there might be some instructor that can just direct on, on the uses (?) of that. Just real quick, just proceed I, I think it, it might help.

I: Do you think it would make it easier or more difficult for you to use a defibrillator if it was delivered by drone?

R: I do (?), I think it, it would be more easier if, if maybe an assistant also would be present.

I: Why do you think it would be easier if there was someone there?

R: (Pause). Yeah, yeah, that's ... For me, if, if there's someone close to me, that also have idea about that, so I, I think it, it, it would help, so, rather than just me alone, and not being, a lot more, have a lot more idea about the (inaudible), and a lot more of those.

I: Okay. And how would you feel about leaving the person, if they are by themselves?

00:31:36

R: Sorry?

I: So, imagine that you're with the person, the person who suffered a cardiac arrest, and you have to leave them to get the defibrillator from the drone. How would you feel about leaving the person to do that?

R: Actually, I might, I might not want to leave the person to, to get this drone if, if it might get some assistance from somewhere, but I dunno my, my concern is just to, to make the person, to ... I would be scared to leave the person, so I, I, I wouldn't want to leave.

I: Okay. And how far would you be willing to go, to get the defibrillator that had been delivered? I know you said that you don't want to, you wouldn't want to leave them, but how far do you think you'd be willing to go to get the defibrillator?

R: Just, just, just maybe within, within the scene. Not, not, yeah, I don't have to go, that I have to lose sight of the person with, with this cardiac arrest.

I: Yeah. Yeah, you wouldn't wanna lose sight of them, that makes sense. Okay. Would the location of where the cardiac arrest had happened, would that affect your thinking on how far you'd be willing to go to get the defibrillator? So, would your thinking around this change if, say, it happened in your house, compared to if it happened outside, in a public domain?

R: I, I, I can't really say much on that, but if it's, if it's, if it's in the house then, maybe in the compound, the house, I (inaudible), no I can't give you straight answer with that.

I: No, that's fine. And what do you think the role of the call handler is in all of this?

R: Sorry?

I: What role do you think the call handler has, when, in, when we're thinking about drones, defibrillators being delivered by a drone? What role does the call handler have in facilitating that process?

R: I think that the person is the one in, in the, in the, in the right, in the, in the right space to, to maybe check the drone to help (inaudible) down also. Then, maybe, maybe for, for me. I should just be able to, just be with the person right there at the scene. So, so, yeah, I, like, I'm just thinking.

I: (Pause). Did you have anything else you wanted to add to that? Or have, have you said what you wanted to say? Because I was also wondering what you would want to know from the call handler about a defibrillator that was delivered by drone. Is there anything you'd want the call handler to tell you?

R: Yeah, definitely. He, he, he should tell me about, about the, how, how you might navigate it, and all of the, of, give, give me an update about maybe how, how to, yeah, careful (?) (inaudible) that. But just, just my, my, my issue is just that time might not be, at (inaudible) on our side to be able to do those things in depthly [sic], to be able to explain all that depthly.

I: Would you want the call handler to tell you that the drone was coming, or only when the drone was there? What, what do you think?

R: Yeah, for, for, for me, I would, I would love to know if, if it would take 10 minutes for the, for the drone to arrive, or the (inaudible) I should just be informed, I would just be informed. Maybe before the arrival of the drone, I, I, I should be told about, about it, and about how to let it down, and how to proceed, so, before that, before it arrives. So, if it arrives, I won't have to waste a lot of time to, to, you know, start that knowing about all of those. I'm (inaudible) already. So, it just (inaudible).

00:36:08

I: Okay. And what concerns, if any, do you have about this?

R: Sorry?

I: So, what concerns, if any, do you have about defibrillators being delivered by drones?

R: It's just (inaudible) response time, so, this drone may, may not always be (inaudible) available, or deploy quickly enough in emergency situations. So, it just (inaudible) response time, there might be more, the issues might have with this drone. So, (inaudible) also, before we talked about the instructions, so, yeah, to, to ensure we make use of it correctly. So, that's, that's something to, that might be on my mind.

I: And what problems or issues, if any, do you foresee?

R: Sorry, can, can, can you come again?

I: Yeah. So, what problems or issues, if any, do you foresee with using this technology, with using drones to deliver defibrillators? Can you see any problems, or issues with it?

R: (Pause). Yeah, yeah. One, one of the (inaudible) is, is just, probably, sometimes (inaudible) make noise, and more of, like, safety. So, those are the things that, that would just be in my mind.

I: The noise of it, you say?

R: Yeah.

I: Yeah. Yeah. Okay. For yourself, or the neighbours, or the, or ... Who might ...

R: It just depends on, on where it take place. It might be in, in the, in the, in the space where might, they might not allow that. So, that's why I, I would just say public acceptance. Maybe it might not, most of some place that is a bit, place that is lots more quiet. So, you're having to make use of that, yeah, and you don't know if it's been accepted in, in the area, because it just happened suddenly. So ...

I: Yeah.

R: ... no one can tell about the, so it's not so a circumstance that, if we're seeing that we, we have to try to transfer this thing to somewhere, and just to, that it's been, that this drone has been accepted. So, (inaudible) this happens suddenly, so you just have to bring it over to the scene at once, without having to, to, to really know in any depth about if it's been accepted in that area. So, the, (inaudible) just say the noise, and just safety. And also, privacy, and lot more of those.

I: Privacy as well, you think could be an issue.

R: Yeah.

I: Okay. Could you just go into a bit of detail about what you mean by that? Privacy for who? Like, why might that be an issue? That's very interesting. Why might that be an issue, do you think?

R: I, yeah, I just think that address concern privacy, you don't know if it, it might be something (inaudible). I can just that more in, in depthly, but I just think privacy, too, might come in place, couple together with safety.

I: Okay, thank you. So, I just have some final, brief questions that may be useful for our research. So, if I may, what is your age and gender, please?

R: Yeah. I'm, I'm 29. Yeah. I'm, I'm a male.

00:40:15

I: Thank you. And have you had training in CPR and defibrillator use?

R: Actually, I haven't had more of a precise training, but I, I, I've been told about this by my GP.

I: You've been for, for what, by your GP, sorry?

R: Yeah, I've, I've been, I've been told about it by my GP.

I: Okay, when was this?

R: Yeah, that, that, that was quite some time, so, it, maybe early last year, so ...

I: Okay. So, was it CPR training only, or CPR and defibrillation?

R: Yeah, it was, it was more of a, like, a general training, but with it, the CPR training to was involved. So, that, that, that was the first time I heard about it, heard about it, (inaudible) all the processes.

I: And did this training occur before or after the incident that you've discussed today?

R: (Pause). Sorry?

I: So, so, you know the training that you received from your GP. Did this occur before you assisted with a cardiac arrest, or did it, did the training happen after you assisted with the cardiac arrest?

R: It, it happened before the cardiac arrest, but even after the arrest too, after that time, I, I still have to go in depthly to know more about it, just, just in, in, in case something (inaudible) happen, I, I might be bold enough, because now I, I'm a lot more knowledgeable. So, so that's how (inaudible). But the first time I heard about it, I haven't encountered any.

I: You haven't encountered any ...?

R: The, the, the cardiac arrest, I haven't encountered any, so, yeah.

I: Okay. You haven't encountered any more cardiac arrests since the last time you assisted with one, is that what you mean?

R: Yeah.

I: Yeah. Okay. Is there something else you would like to add to what you've said today?

R: No, I, no, I don't have anything else to add.

I: Okay. So, I'm just gonna turn this, the recorders off, and go through what will happen next.

END OF INTERVIEW

Participant 2

I: Okay. So the audio recording has begun and it will continue until the interview questions end. So thank you for agreeing to participate in this interview, which forms part of a research study being completed by the University of Warwick and Welsh Ambulance Service NHS Trust. Before we can proceed with the interview, I need to reconfirm your eligibility. So can you confirm that you are aged 18 or over?

R: I confirm.

I: That you are comfortable to proceed with the interview in English?

R: Yeah, I confirm.

I: That you have provided assistance at a cardiac arrest at some point in the past?

R: Yes, I confirm.

I: And that you agree to have an audio recording made of your interview?

R: Yeah, I confirm.

I: I've received a signed consent form from you. Has anything changed since you signed the consent form?

00:01:17

R: No.

I: Thank you for confirming that. So the interview will last for around 45 minutes and your participation is entirely voluntary. So you can pause, reschedule or terminate the interview at any time without providing a reason and without your rights being affected. And you can choose to have your camera on and off. That's absolutely fine. So there are no right or wrong answers. We are interested in your experiences of helping someone who had a cardiac arrest and your attitudes around using drones to deliver defibrillators. Now, do you have any further questions about the study or about the interview process before we begin?

R: For now, no question. I can begin.

I: Excellent. Thank you very much. So can you tell me as much as you can about when you provided assistance to the person who had a cardiac arrest?

R: Can you rephrase it?

I: Oh rephrase the question. Okay. So what happened when you provided assistance to somebody who suffered a cardiac arrest? What did you do?

R: (Pause) At the first instance, I was scared. I was, I was worried. I, at first I thought the person was no longer alive because he had stopped breathing. The first thing I did was to, I give a call, of course. I called 911. And then I wasn't able to get fast help, so someone close by had to call a local emergency number. And after that I, I laid the person on the floor. Sorry, please, the question you're asking me, is it how I actually helped person, is it the actual incident or the experience behind the incident?

I: So it's, "How did you provide assistance to the person?" So, so if it would help, where did the cardiac arrest happen, first of all?

R: Okay. It was at a bar.

I: At a bar?

R: Yeah.

I: Yeah. Okay. And who was the person who had a cardiac arrest? What was their relationship to you?

R: A stranger.

I: Okay.

R: I don't actually know the person.

I: Okay. Okay.

R: I feel his cardiac arrest was, he was very high on drugs.

00:04:28

I: Right. Yeah. Okay. So did you realise what was happening at first? You said that the person had stopped breathing. Did you …

R: Yeah. At first … that was my very first experience. So at first I was confused. Like, people around didn't even know that, oh, he had stopped breathing. He stood up, he staggered, and the next thing he dropped on the floor. So initially we all thought that, "Oh, maybe he's just high." And then I walked closer to him because I, I get easily attracted to those kind of incidents. I walked over to where he was lying down. I tapped on him, if he was okay. He no response. I had to squat. I got closer, I carried his hands. His hand swell up. I noticed he wasn't breathing. I, I called (inaudible) to come over, to reach out. I tried calling 911, but I wasn't able to connect. Someone called for help because the bar was close to a healthcare centre. At first, I laid him down on the floor. If what you're asking me is the initial process or how I conducted the, the help I rendered for him, that's the CPR.

I: Yes. So you performed CPR?

R: Yeah.

I: Yeah. Okay. And did you have immediate access to a defibrillator?

R: No. But then another instance I have watched someone use it, like, on a different occasion, a different day, like, a different year.

I: Okay. But not for that specific incident. You didn't have access to one.

R: No.

I: No.

R: No. No. No.

I: Okay.

R: It wasn't close by.

I: There wasn’t one close by. Right. Okay. Yeah. Yeah. And you say that you were in a bar, so presumably other people were there with you?

R: Yeah.

I: Yeah. And, and how did that make you feel, that there were other people there?

R: Generally speaking, that very instance, I, I don't know. I was confused. I was lost. I didn't, I, I don't know. But I would say that I, I get easily, I don't like crowd. But then there was a crowd there, and it seems like the people there were either high, they were, like, they were not okay, I, they were not sound enough to perform the CPR. So one of the person who said he, he can do it was already drinking, so it wasn't safe, or we felt it wasn't safe for us to, for him to do it. So I had to take it upon myself.

I: Right. Okay. You had to take it upon yourself to perform CPR, is that what you mean?

R: Yeah.

I: Yeah.

R: But at first I felt really, I felt confused. I felt, I was tense. That was my very first time attempting it. So I was worried about the man's life. I was worried about, "What if something happens? What if in the process of doing this, this person maybe ends up losing his life? Would I be held accountable?"

I: Yeah.

00:08:35

R: I, I, I, I didn't want any implications. I didn't want to be involved in making any entry to report an incident or to tell a story. But then I, the urge to help the, the young man was more superior, so I just had to do it.

I: Sure. Yeah. And do you know what the outcome was?

R: Yeah, I was able to (pause) help him till … I, like I said earlier on, there was a primary healthcare centre close by. (inaudible) the nurses, the ambulance arrived and he was still breathing. I went with them to the hospital and I kept on checking up on him till he was okay.

I: Yeah. Yes. Yes.

R: From, from what I, from what I, I gathered, the substances he consumed blocked his, I, I don't know, his ability to breathe and made him (loss of audio).

I: And made him what, sorry?

R: I said the high dose of substances he consumed …

R: Yeah. Yes.

I: … was what made him blackout.

I: Yeah. Yeah. Okay. Okay. And at the bar, just going back to when the gentleman collapsed at the bar and there were other people there, can you remember how they organised themselves and what they did?

R: If I can recall, there were about seven to eight people there. And the people who walked closer to the man, there were (inaudible) up to five, if I am to include myself.

I: Yeah.

R: So about two or three people didn't really care about what was going on. So, I had about three or four people around me who were also there to assist the man.

I: Yeah. And, and what did they do? How did they assist the man? Did they do something similar to what you were trying to do, or did they do anything differently, or …?

R: No. No. Most of them were just standing and staring.

I: Right.

R: Why? I, I believe they were staring just because they didn't know what to do.

I: They didn't know.

R: They knew very well that they were not in the best place to offer any medical assistance to the person. But then I, like I said, one of them reached out to give a call across for help. But the rest were just standing and watching. Someone had also … because where he fell down wasn't a stable ground. Like, it was a bit slopey. Like, it had a stairs. So we had to carry him up, like, back into the bar and then kept him on the bare floor that's flat.

I: Yeah. Yeah. Must have been difficult then. So could you tell me as much as you can about the 999 call?

R: Like I said, I wasn't able to connect. I don't know. I had a technical issue there. So some other person called the clinic, the healthcare centre that was close by.

I: Okay. And do you know if that person used, well, I, presumably a mobile, or did they use the phone at the bar? How did they make that call?

00:12:48

R: They used a personal phone …

I: Yeah.

R: … because I think the person was a worker there.

I: Yeah. Okay. And did anybody have to leave the patient to make, to make a call?

R: The call was made there, like, right there. The person didn't have to leave. Like, well, I don't know how to put it, but the person was, like, still in the bar or know where the person was lying down.

I: Yes. So, so the person, just to clarify, sorry, the person who was making the call was with the person who had collapsed. Is that correct?

R: Yeah.  Yeah.

I: Yeah. So you were all with the person who had collapsed, you didn't leave them.

R: (Pause) No, I didn’t.

I: No. Okay. And do you know what the 999 call handler said or what help they offered? Can you remember anything about that?

R: I believe the person called the healthcare centre's emergency number, like, not directly 911.

I: And, and do you know what was said? Do you know what the 999 call handler said?

R: He, I, I don't really know the exact words, but the person called and reported an incident that someone has just collapsed, that the person is no longer breathing. And they asked for the location. Person gave the location of the bar. And then that's when we had to wait. After the call, we had to wait for the ambulance to arrive.

I: Yes. Yeah. And, and while you were waiting, did the call handlers say anything to you? Did they offer any help? Did they ask you any questions?

R: No. Well, I, they asked questions, who the person was …

I: Yeah.

R: … what was wrong with him, what was the person's physical condition at that moment, what, what caused the person to fall, falling. Because there was a bit of confusion there. Has he been … we didn't found, we didn’t find some substances on him. Maybe would just think that maybe he slipped, because he was about going down the stairs. But when he fell, he was about leaving the bar, and then he slipped and fell. So had he been … we didn't find substances in his pocket. And the alcohol smell around him, maybe I would have been confused. Maybe the information we would have given out was that he slipped and fell and lost consciousness, or realising that he was already high even before walking close to the stairs. And we concluded on our own that could be the cause, and that's what we told the emergency centre.

I: Right. Okay. So at this point did you think that he had a cardiac arrest or did you just think he was asleep from the drugs?

R: (no audible response)

I: Or did you think (inaudible) …

R: Sorry, I heard you correctly, if I heard you correctly, you're asking me if at that point I thought he was sleeping or I thought he was affected by the drugs?

00:16:55

I: Or the cardiac…

R: Is that what you say?

I: Yeah. So how did you know that he was in cardiac arrest then. Because you said that it just looked like he was sleeping and high on drugs. So how did you know that it was a cardiac arrest?

R: Okay. I think you heard me wrongly, you didn't hear me, hear what …

I: Sorry.

R: … I said.

I: Apologies.

R: I didn’t say sleeping. I said he slipped, like, he …

I: Yeah. Oh, he slipped, yes. Yeah. Yeah.

R: … fell, or yeah.

I: Yeah.

R: Now, how I knew that he had a cardiac, cardiac arrest …

I: Yes.

R: … he wasn't breathing.

I: Yes. He wasn't … yeah. So …

R: Like I said …

I: Yeah. Yeah.

R: … he fell …

I: Yeah.

R: … from the way he was walking when he stood up from his seat ...

I: Yeah.

R: … showed he was, he wasn't walking well. He was staggering …

I: Yeah.

R: … and trying to hold the pillars there. So I, I was still observing him. When he fell on the floor I thought he would maybe try standing up immediately. Or I, I feel like as soon, as he fell on the floor or I feel like the, reason why he fell was because he blacked out …

I: Yeah.

R: … and he wasn't in control of his body …

I: Yeah.

R: … anymore.

I: Yeah.

R: Because even when I walked close, I knelt by his side. I carried his hands and it's, I didn't feel any flow.

00:18:25

I: Yeah. Yeah. Sure. Brilliant. Thank you for clarifying that. And then just going back to the call handler, did you ask the call handler any questions? Or the person who had made the call, did they ask the call handler any questions?

R: Yeah, the person asked what we could do. And that's when they suggested CPR that I was already doing. And they also asked if the building, that’s, if the bar had (pause) if the bar had AED. And we said, "No."

I: Right. Okay. And I'm going to ask you about the CPR and the AED in a moment. But just for now, what are your reflections on the interaction with the person who made the call and the call handler? So what do you think went well and what, if anything, was difficult?

R: I feel like the receivers of the call, they, they acted more worried, even more than the caller. They, they showed a high level of interest and they, they, they showed a high level of care. The, like I said, the person who made the call wasn't really that, wasn't sounding that worried. Like, like, it was really an emergency. But the person telling the emergency centre that this person wasn't breathing, I feel like they showed this high level of care, and they gave reassurances that they would be there in a couple of minutes, that the ambulance team would be there to take care of the situation. When they first arrived, they continued, they carried him into their vehicle and they continued with the CPR.

I: Okay. So it sounds like there was a, a positive interaction between the, the person who made the call and the, and their interaction with the call handler. Were there any technical issues while the person was speaking to the call handler on the phone?

R: No. No.

I: No. Okay. So it went fairly smoothly then, you would say.

R: Mmm.

I: Yeah? Okay. So …

R: Yeah.

I: Yeah, sorry, was there something else you wanted to add to that?

R: No, not (inaudible).

I: Okay. So you mentioned before that you performed CPR. Can you tell me as much as you can about your experiences of performing CPR, please?

R: That was the first and only time I have attempted it. I, like I said earlier on, I was tensed.

I: Yeah.

R: But then I, I felt, I had this drive that I, I have to do it right, I have to strive, that I, I have to do my best. I, I really didn't want the person to have, to lose his life, or I didn’t want … I didn't anticipate any negative (pause) what did I just say? Any negative thing to happen at that very moment. So I would say my experience, at first I was tense, but later on I gained courage because people around me encouraged me and I … I don't know. But I felt like I gained confidence when people said I was doing it right.

I: Yeah. And did you do chest compressions only?

R: Not just that. I, I, I breathed into his mouth too.

I: And what are your reflections on how that went?

00:22:59

R: It was strange because I, I have never gone that close to male gender before. So it was very strange. I felt really strange, but I just had to do it. It would have been better if it was maybe female. But it felt odd at first, but I, I had to do it. I didn't mind. I didn't even realise I was doing it until I, I was done.

I: And did the 999 call handler offer or give you instructions on how to perform CPR?

R: No, they didn't really. They didn't, they didn't give. But the caller told them that someone was there who could do it, and I feel that's why they didn't offer that.

I: Right. Okay.

R: Because …

I: Yeah.

R: … at the time of the call I was already doing it.

I: Yeah. Yeah. Okay. So you weren't being told what to do. You were doing it, you were doing what you thought was right. Is that correct?

R: Yeah.

I: Yeah. No one was telling you what to do. You were just …

R: No.

I: No. How did that make you feel, doing it on your own and not being given any advice? (Pause) Did you really think about that or were you just focused on doing the CPR?

R: (Pause) I generally, I didn't think about that.

I: No. No, no. Okay. That's fine. So you, can I just clarify that you didn't use a, a defibrillator, a, an AED?

R: No.

I: No. No. Okay.

R: No.

I: And I, I think you've said already, what are your thoughts on why a defibrillator wasn't used?

R: (no audible response)

I: Do, do you know why one wasn't …

R: (inaudible)

I: … used?

R: It wasn't used because it was not available there.

I: Yeah. Yeah. Okay. So was there one that you were aware of?  There, or there just wasn't one at all anywhere within the vicinity that you were in?

R: No.

I: No. Yeah, okay. Did you perhaps realise that there was a nearby defibrillator that you hadn't remembered at the time?

R: Can you repeat what you just …

I: Yeah, sure. So did you subsequently realise that there was actually a nearby defibrillator you just perhaps hadn't remembered at the time because of the crisis that you were dealing with, you'd forgotten? Or was there really no defibrillator nearby?

00:26:07

R: There was none …

I: No.

R: … at the bar there. None. It wasn't available. Because someone asked for it.

I: Yeah. Yeah. And what about outside the bar? You know, do you think that there was one, like, outside, like, maybe over the road or in the adjacent street? Or, or was there just no defibrillator anywhere?

R: Actually, no one went out to check for it.

I: Yes.

R: So we left that. Since the bar said they don't have it, no one really went to check for it.

I: No.

R: But I feel like the next building should have it, other places around might have it.

I: Yes. Yeah. Did the call handler advise you of a nearby defibrillator?

R: No.

I: No. No, no. So you asked at the bar, the bar said they didn't have it, and then nobody mentioned anything more about it. Nobody said, "Oh, there's one over the road," or, yeah, nothing like that.

R: (no audible response)

I: Okay.

R: Nothing.

I: Okay. Thank you. What are your thoughts about using a defibrillator in the future?

R: (inaudible) I would like to use because I feel like it's very, very more effective when properly used.

I: Yeah. Why do you think that?

R: I think that way because I have watched someone being revived from cardiac arrest be, like, much more easily.

I: Oh, okay. You've seen someone, you've seen a defibrillator being used on somebody and they recover much quicker or revive much quicker. Is that what you, is that what you're …

R: Yeah.

I: Yeah. Yeah. Is there something that would make it easier for you to use a defibrillator in the future?

R: (Pause) Yeah. Having, for instance, when it's actually happening, having quicker access to it …

I: Yeah.

R: … would enhance my willingness to use it.

I: Yeah. Of course. Yeah. So just moving on now to drones delivering defibrillators, is drone delivery of defibrillators something that you've heard of?

R: (Pause) No.

00:29:06

I: Have you heard of drones?

R: Yeah, I, I know a little about drones.

I: Have you seen drones used or used one yourself? Perhaps have you seen it on the TV?

R: I have maybe (?), yeah, I've seen it on TV.

I: Yeah.

R: I've seen it being used, but I've never used one myself.

I: Okay. In what context, apart from the TV, in what context have you seen drones being used? Is this something in real life or is this on the TV?

R: No, I've only seen it on the (inaudible) TV, movies.

I: Yeah. Okay. So how do you imagine, (coughs) excuse me, how do you imagine drone-delivered defibrillation works?

R: (Pause) I don't know. Like, actually it's something that would be good. Because from the little I know about drones, I feel like they are faster. If that kind of services is being required and a drone is sent, I, I believe it will yield a positive effect, even though it should have its own disadvantages.

I: Okay. What might be some of the disadvantages of using a drone to deliver defibrillators?

R: For instance now, someone who, someone who don't really have knowledge on how to use it …

I: Yeah.

R: … might have difficulties. That's the recipient. Might have difficulties using it properly.

I: Yeah. Yeah. You, you said that drones might be able to be faster. Do you mean in …

R: Yeah.

I: … terms of getting the defibrillator to the person, or do you mean something else?

R: Yeah, getting it to the person.

I: Yeah. Yeah. Okay.

R: And (inaudible).

I: (Pause) And also what, sorry?

R: I think, I also just feel that there are some parts that you could find dissolving (?), and it might be difficult for drones to locate the exact spots where the emergency is happening.

I: So that might be difficult for a drone-delivered defibrillator. It needs to be quite exact in terms of where it's delivering the drone to.

R: Yeah. And also the distance also. If it goes to a place where there's really no signal for it, it could prevent delay and further extend the time it's supposed to deliver.

I: Interesting. So there could be, so it could be faster but there could also be delays as well.

R:  Yeah.

I:            How do you think the defibrillator can get safely to the ground?

R: (Pause) I don't really know. But I feel like if a drone is being sent, it should be controlled by someone. And the drone itself should have, like, a camera on it, like, a video. It should, it should be tracked in a way that the visuals of the drone can be viewed on a screen. So that way, if the drone has control, I feel it can easily be dropped.

I: And then how would one go about getting the defibrillator from the drone?

R: (Pause).

00:33:38

I: Or are you saying that it would just need to, it would just drop on the floor and then the person could just use it as …

R: I, I don't really know if drone could come down, like, very low to the ground. But if it can, I feel like whoever is controlling it should be able to bring it down so the recipient could retrieve it.

I: Yeah.

R: But if it cannot come down to the ground, I don't know how that's possible. Maybe it should have something like a rope, I don't know, a wire, or something to be able to drop it off. I'm interested now (?). I haven't directly received something from a drone, so I have little knowledge of how it works. But I, I believe, in terms of speed, we could partly rely on drones. But in terms of delivering that to someone who don't have knowledge about how to use the AED, I, I feel like if it's delivered by a human, the person can literally explain and help facilitate the process of helping that person in an emergency situation. But if it's a drone, a drone cannot talk, so there, there would be a delay in this person coming to learn how to use it within that short space. Don't know if you get my point.

I: Yes. Yeah. So the person interacting with the drone would need to be fairly skilled in terms of getting the defibrillator from the drone. And perhaps flying the drone as well? Is that, are you thinking in those terms? Or is it …

R: Yeah.

I: … or are you thinking of just it being delivered to the person, the person would need to be skilled in getting the drone from the, getting the defibrillator from the drone?

R: Yeah.

I: Yeah. Okay. So how would you feel about a drone bringing a defibrillator to you whilst helping someone having a cardiac arrest?

R: (Pause) I'd be excited. I would just wonder at the advancement of modern technologies.

I: Yeah.

R: I would, I would like it. That is if I have prior knowledge of how to use it.

I: Yeah.

R: I'll be happy. But I would prefer a human bringing it. That is if I have never used it for the first time and I want to make use of it in an emergency situation.

I: Yes, yes, (inaudible) knowledge and skills of how to interact with the drone and get the defibrillator from the drone is, is very important. So do you think it would make it easier or more difficult for you to use a defibrillator if one is being delivered to you by drone?

R: (Pause) May you repeat yourself?

I: Yeah. So, so imagine, if you want, that situation where you were helping the person in cardiac arrest. Would it have made it more easier or more difficult for you if a drone had delivered a defibrillator?

R: Can you rephrase it?

00:37:34

I: (Clears throat) So do you think it would be easier to use a, a defibrillator, an AED that has come from a drone? Do you think …

R: Yeah.

I: … you'd find it easier to use a defibrillator in that situation? Or do you think it'd be more difficult to use a defibrillator that has come from a drone?

R: It’s probably, it's, I, I, like, I, I think I said personally I actually need some knowledge now of how it can be used. So …

I: Yeah.

R: … I prefer it being delivered by a drone, because the speed matters.

I: Yeah. Yeah. Yeah. Okay. And how would you feel about leaving the person if they are by themselves? Yeah.

R: (Pause) How will I feel about doing what?

I: About leaving the person. So if you have to leave the person to get the defibrillator from the drone, how would you feel about that, if the person's not …

R: No, no …

I: … with anybody?

R: … I wouldn’t like that. I won't like.

I: You wouldn't like it. Okay. Why wouldn't you like that?

R: I wouldn't, I wouldn't like it, because at that very point I might be tense, might be worried. So leaving the person in such a situation isn't something I would want to do.

I: Yeah. Okay. So how far would you be willing to go to get the defibrillator that had just been delivered?

R: It should be, it should be on that very thing. The drone should be able to come to the very spot where the incident has happened.

I: Yeah. Yeah. Okay. And would, like, location, so if the cardiac arrest had happened in the public domain or in your house or indoors or outdoors, would the location affect your thinking on this?

R: (no audible response)

I: So, I don't know, imagine if a cardiac arrest had happened in your house and you had to leave the person to get the defibrillator at your front door, would you be more willing to do that than if you were outside and you had to walk a little way down a street to get the defibrillator? Do you think …

R: No …

I: … it would matter?

R: … I would not. It would matter.

I: Yes.

R: I wouldn't like it.

I: No. No. You just wouldn't like to leave the person at all.

00:40:22

R: Mmm.

I: Okay. And what do you think the role of the 999 call handler is in all this?

R: What do I think, what…

I: What do you think the role of the 999 call handler is in all this?

R: (no audible response)/pause

I: So what role do they have …

R: I think, like, in a situation whereby they get a call from someone that someone have a cardiac arrest and then there's no one there that knows what to do, I feel like they're in the best position to guide on a willing person that's ready to attempt saving a life on the necessary steps, what to do, how to do it, and the whole process involved, while send a team to come pick up the person.

I: Yeah. Yeah. What, what would you want to know from the call handler about drone-delivered defibrillators?

R: If, if I have no knowledge, knowledge about it …

I: Yeah.

R: … the first thing I would want to know is how it's being used, does it have side effects, (inaudible) how I can use it and it has the negative effect on the person. I, I would ask clarifying questions and I would ask questions, I would ask for instructions to be repeated severally so as not to make mistakes.

I: Yes. Yes. Yeah. And do you think you'd want to know whether the drone was coming? Or would you prefer to only know when it was there?

R: (no audible response)/pause

I: Would you prefer the call handler to say, "Okay, one's coming now. It's going to take ten minutes," for example, or …

R: Yeah.

I: … would you …

R: I'd prefer that.

I: You'd prefer them to give you updates as it was happening.

R: Yeah.

I: Yeah. Okay. And I know you touched upon this earlier. Perhaps you can think of any other concerns or problems that you might have about drone-delivered defibrillators?

R: No.

I: No.

R: I don't think of any now.

I: No. Okay. So thank you for that. I just have some final, brief questions that may be (coughs) useful, sorry, for our research. So if you don't mind me asking, what is your age and gender?

R: (Pause) Okay. I'm transgender. I, can I give, like, an age range?

I: Yeah, sure, whatever you feel comfortable with.

R: I'm between 25 and 27.

I: Excellent. Thank you. And have you had training in CPR and defibrillator use?

R: No special training.

I: No. Okay. That's brilliant. Now, just before we finish, is there something else that you would like to add to your answers today?

R: No for now, no.

I: Okay. Thank you. That's great. I'm just going to stop the recording.

**END OF INTERVIEW**

Participant 3

I:  Okay. So the main interview audio recording has begun and it will continue until the interview questions end. So …

R: Okay.

I: Thank you. So to start, can you tell me as much as you can about when you provided assistance to the person who had a cardiac arrest?

R: Okay. That was in [year], [year]. I went to visit my father-in-law. And I, prior to that time he, he called, he called my husband, his son, and he, he said he needed to go for a check-up. And my husband wasn’t free to take him for the check-up, so I decided to go get him to the hospital. And when I got there, he was just lying on the floor, lifeless. And when I checked his pulse, he wasn’t responding, he wasn’t breathing. And I, I *shook* him (makes a shaking sound with hands) so hard. I shook him so hard. He was not responding. I was calling his name. No response. And then it occurred to me that this could be a cardiac arrest, and I decided to do a CPR on him. And before that I, when I saw him lying I immediately called 999. I called the emergency line to come help me salvage the situation. And then I did a CPR on him. We were (breathes out) oh my God, I really tried my best that day. I, I did. I was scared. I was, I was so scared. I, it was this *fear*. I had this fear, 'cause I've never experienced such a thing. But I do see it in movies and I see, I see things like that in movies, but I have never experienced it. And seeing a lifeless body on the floor without knowing what to do … and then it just occurred to me that, "This, this could be a cardiac arrest. Why not give him a CPR to see if he could respond to it?" And that was how I just started …

00:02:52

I: That sounds …

R: … CPR on him. So pressing his chest and … I think it took about 15 to 20 minutes for him to respond. And when the ambulance came, the man who came on him pressed him a bit harder. And that was when he immediately responded. He shook his hands. And he wheeled him to the ambulance and we went straight to the hospital. So …

I: Okay.

R: … that was it. But they were able to salvage the situation at the hospital, but ever since then he hasn’t fully recovered. He hasn’t fully … think it affected him to some extent.

I: Sorry.

R: 'Cause the hospital said he, they think he was there for, like, ten to 15 minutes before I got him. So, and it means I got in late to help resuscitate him on time, and that was why it really affected him.

I: Oh, okay. Are you okay to continue with this interview? I understand that it can be quite difficult recounting these experiences. Do, would you like to continue? I just wanna check.

R: (Pause and breathes outs) Yeah, let's go on. Let's go on.

I: Okay. If you do wanna pause or stop, just, just let me know. Okay?

R: Thank you (?).

I: So can I just clarify, if I may, where the cardiac arrest happened? Did it happen in a hospital or were you going to the hospital at the time? Could you just clarify that, please?

R: No, it happened at the house.

I: Ah.

R: The house.

I: Okay.

R: At his house. Yeah, at his house. I went to get him to the hospital. He said he had a check-up. So I went to his house to get him, and I found him lying lifeless on the floor in his house, not a hospital.

I: Okay. Thank you for clarifying that. So how soon afterwards did you realise it was a, a cardiac arrest? So as soon as you came into the house, or a few minutes after? Or, or how did you know?

00:05:30

R: Okay. It was when I touched him and he was unresponding. I shook him. He wasn’t responding. He wasn’t responding. And then it just occurred to me that this could be a cardiac arrest.

I: Yes. So pretty soon.

R: He's quite aged though.

I: Sorry?

R: I said, "He's quite aged though." So I just knew it could be a cardiac arrest.

I: Sure. And did you have immediate access to a defibrillator?

R: No. No.

I: Okay.

R: No.

I: And what, were you by yourself?

R: Yes, I was, with my son.

I: Okay. Okay. So how did that make you feel?

R: Like I said, I was really terrified. My son was crying. I was confused. I was scared. I (pause and breathes out) well I was terrified. I was terrified.

I: Yeah. Understandable. And I'm sorry that you had to experience that. So you said that you made the 999 call. Can you tell me as much as you can about that?

R: Okay. When I made the call, they asked what … I said, "I just met him lying on the floor lifeless. He isn't responding." And they said, "It's alright. They're coming. They're on their way down." They said they were going to get to me as soon as possible and (sniffs) then the ambulance came, I think, about 15 or 20 minutes later (wavering voice).

I: Yeah. Are you, are you sure you're okay to continue? Or would you prefer to stop?

R: It's alright. Let's go on.

I: I'm just sensing the emotion in your voice and … are you sure?

R: Let's go on, please.

I: Okay. So did you use a landline or a mobile to speak to the call handler?

R: Okay, I used my mobile.

I: Okay.

R: (inaudible)

I: Sorry?

R: I used my mobile phone, my phone to call.

I: And did you put your mobile on speakerphone?

R: No, no, I … I can't remember. I, no, I, I don't think so. I don't think so.

I: And did you leave your father-in-law to make the call, or were you with him?

R: Did I leave my …

I: Did you leave his side? Did you leave your father-in-law's side to make the call? Or were you by …

R: No.

I: … his side?

00:08:32

R: No, I did not. I was, I was right there with him. I was on the floor with my bag (voice breaks/wavering voice). And I brought out (?) my phone and called. I couldn't leave his side (breathes out).

I: Okay. And can you remember what the call handler said?

R: Okay. It was a woman, and she asked my name. I told her. She asked what was wrong with him. I said, "I do not know. I just came in and met him lifeless. I've been calling and shaking him. He's not responding." And she said, "Okay, do not be afraid. Do not panic. We'll be there shortly. Just," she said, "Just administer CPR. Try your best. Try what you can do. We're on our way. We'll be there as soon as possible."

I: Did she give …

R: And I think she heard my son crying at the background. So, so I, I, I think she felt the tension in the house. She just gave some words of consolation and comfort.

I: Did she give you any advice as well or …?

R: Yeah. She said I should do a CPR on him, I should try my best to make sure he responds, even if it is a physical movement. So, and I just started doing CPR on him, pressing to see if …

I: Yeah. Sure. And what questions did the call handler ask you?

R: Okay. She said, "Is, how is he lying?" She asked, "How is he lying?" I said, "He's just lying there on the floor." She said … what did she say again? I think … okay. She asked, "Is he bleeding in any part of his body?" She asked that, "Is he bleeding from the nose?" I said, "*No*." She said, "Are his," she said, "Open his eyes." I said, (louder) "His eyes are *shut*. I can open them." And I think she said, "Check if he has an injury on the head." And I said, "No, he doesn’t have an injury." She said, "Okay," I should try to do CPR on him.

I: Okay. And did you ask the call handler any questions?

R: Okay. I did not. I only told them I needed an ambulance to come get him, that he's not responding, he's lifeless and he's not responding. And that was when she told me not to panic, that I should try to do CPR on him, I shouldn't panic …

I: Sure.

R: … and they'll be on the way.

I: And what are your reflections on the interaction? So what, what went well and what was difficult?

R: (no audible response)

I: Hello?

R: (inaudible) I wouldn't say.

I: You, you wouldn't say? Did, is that what you said?

R: Can you hear me?

I: Yeah, sorry, did you hear my question?

R: Yeah.

I: Yeah. Okay. So, sorry, could you just repeat what you said the first time? I apologise for not hearing properly.

R: I said at the time I was tensed and I wouldn't say I, I noticed anything. But I think the, the response I got was quite helpful. It was quite helpful and she sounded really professional. She sounded really professional and (pause) and, and very ready to help. I think she sounded really ready to help. She was really professional.

00:12:49

I: Were there any …

R: (inaudible)

I: Yeah. Were there any technical issues or difficulties?

R: While speaking with her?

I: Mmm hmm. Yeah.

R: No. No, there wasn’t. There wasn’t. It was a free call. The line was free and … the lines were free.

I: Okay.

R: There weren't any technical issues.

I: And you appreciated her professionalism towards you at the time.

R: I really did. I really did.

I: Okay. So you said earlier that you performed CPR on your father-in-law. Could you tell me …

R: Yeah.

I: … a little bit more about, about this, if you, if you could?

R: About the CPR?

I: Mmm hmm. About your experiences of performing it and what you did.

R: Okay. (inaudible) that was my first time. And I just had to place my … first I shook to see if there were going to be any response. I shook his shoulders. I called his name. I tried checking for any responsiveness. None. And then I had to push hard. I used my hand to push hard on his chest, hard and fast. And I was just pushing in the middle of his chest. I was pushing. And I think I did that for, two to three times I'll give a break to see if he would respond. And when he didn’t, I did again. I, I was just doing that 'til they got there.

I: So did you do chest compressions only?

R: Yes. Yes.

I: Okay. Yeah.

R: Okay. I remember, I remember, I remember the lady telling me to place his head backward. Yeah, I remember telling me to place, I remember her telling me to place his head backward and also to, I think, lift his nose or something like that. Yeah. She, I remember that.

I: And did you …

R: I also did.

I: You, ah, so you did that. You tilted his head back.

R: Yeah. Yes, I did. I tilted his head backward and … I did that.

00:15:33

I: And did you deliver any rescue breaths with the chest compressions?

R: Sorry?

I: Did you, when you tilted his head back, did you deliver any rescue breaths?

R: Yes. Yes, I did. She asked me to do that. I did that twice. I did that twice.

I: Okay. And what are your reflections on how that went?

R: (Sighs heavily) Well, I, I would say at a point where you just needed one to be alive, you would do anything, go to any lengths, go to any lengths to see the person recover from whatever it is the person is fighting from. So (breathes out) oh. (Sniffs and breathes out)

I: Would you like to, to pause and, or stop this? You know, we, even if you terminate the interview, we, you'll still be entitled to your £25 e-voucher compensation. So it's, you know, if, if that’s, I'm not saying that that is an issue, but if, if that, if that was a concern … it's entirely up to you. You are, you do not have to continue with this interview. So please, yeah, if you …

R: Alright. Alright.

I: … tell me what you would prefer to do.

R: It's alright. It's alright. Reflecting back on what happened, I would, I, I would say I, at the point I just, I just needed to keep the man alive. I just, I just needed to do whatever it is in my capacity to do to resuscitate him, to bring him back to life. 'Cause he's the only family left and (breathes out) it would be so, so, so traumatising, seeing him leave. That, that man, without getting to talk with him one last time, see him or … you know what I mean.

I: Yeah. Sure.

R: So …

I: Yeah.

R: … so I think …

I: Sure.

R: … so that was …

I: Okay. And what are your reflections on the instructions and advice that you were given from the call handler?

R: (Pause) They were very helpful. Very helpful. Reflecting back on the call I made with the woman that day, I, I just, I realised how fickle humans can be and how at some point you just get answers to everything. You get answers to almost everything one needs. And at that point, while I was searching for solutions, searching for an answer, I think I quite got it from the lady. She was really responsive. She was really responsive.

I: Oh, okay. So you felt supported.

R: Yeah, I did. I did feel supported.

I: Okay. And did you use a defibrillator?

R: Sorry?

I: Did you use a defibrillator?

00:19:38

R: No, I didn’t. They did. When they came in, they did.

I: Okay.

R: They did.

I: Yeah.

R: They did.

I: So what are your thoughts on why a defibrillator was not used prior to the paramedics using one?

R: I, I didn’t get that.

I: Sorry. I'm just wondering what your thoughts are on why a defibrillator was not used prior to the paramedics coming.

R: Okay. So for starters, I wouldn't say I know how to use it. I didn’t know, I do not know how to. And frankly speaking, that was my first time of seeing that. That was my first time of seeing that. So when I asked and they said it was defibrillator and it was used for cardiac arrest response, and I said, "Okay. Wow." I said, "Wow. It's the first time I'm learning of this."

I: Okay. So were you aware then of a defibrillator? Were your …

R: Sorry?

I: Had you heard of defibrillators before the paramedics turned up?

R: No, I … no. No. That’s what I'm saying. I said, "No, I hadn't, I hadn't." It was, when I saw it, I asked and they said, "Okay, this is a defibrillator and this is what it is used for." And I think it was in the hospital, while we were at the hospital I had to use my phone to google about a defibrillator to know what it was really all, all about.

I: Did you … I, I know at the time you weren't aware of any defibrillators, you hadn't heard of defibrillators. But after the event, some time after the event, did you realise that there was a nearby defibrillator that …

R: Yeah. Yeah. Yeah, I did. I did.

I: Yeah. And did the call handler advise you that there was this nearby defibrillator, can you remember?

R: Yeah, she (louder) *asked* me, she asked me if there was one there. And I told her I do not even know what she was talking about. And she said I shouldn't panic, that they will be there in a jiffy. She asked me, but I didn’t know, I (nervous laughter) didn’t know what a defibrillator was that she was talking about.

I: Okay.

R: And …

I: Yeah. So she didn’t go into detail with you about what it was. She just said, "It's fine. An ambulance is coming." Is that correct?

R: Yes, she just told me what to do at the meantime before they got there.

I: Yeah. Okay. And that was regarding the CPR. She was telling you how to perform the CPR.

R: Yeah.

00:22:50

I: Okay. So what are your thoughts about using a defibrillator in the future?

R: I would say it's, it's, it's a very good one. It was really responsive and very active. It was really active. I think it's, it's something every home should have. Something every home should have. Yeah.

I: Yeah. And why is that?

R: Just in case of any emergency, just like the one I faced.

I: Yeah.

R: 'Cause if I knew about that and I got there, there was a defibrillator, I would have used it. I would have administered that on him. But I didn’t know. And, and I do not think he had it in his house, 'cause they brought one with them.

I: Yeah.

R: Yeah.

I: Is there something, then, that would make it easier for you to use a defibrillator in the future? I think you've just said, "Have one in, in your house." Anything else?

R: Sorry?

I: So is there something that would make it easier for you to use a defibrillator in the future?

R: Okay. Right. (pause) Like a topic, topic of drone delivering defibrillators, I think that will be a lot easier. Think that would be a lot easier to people with such situations to help them out of that before the ambulance gets there.

I: Okay.

R: I think a drone delivering it, it, it will really help to salvage the situation at the time.

I: Okay. Excellent. 'Cause we're going to move on to that next. So why do you think that drones would be useful? What is it about them, do you think?

R: Sorry?

I: So you're saying that it would help to salvage the, the situation, when someone's having a cardiac arrest. Why do you think that? How can they do that?

R: Okay. 'Cause I think drone is faster. It has a faster response time. Yeah. I think it's faster. And it also has the ability to access any location, any given location. So I think so.

I: How do you imagine it would work, drone-delivered defibrillation? Do you have any ideas, any thoughts on the logistics of it?

R: No.

I: No. That’s fine. It is new technology. So have you heard … you've heard of drones if you're talking about drone-delivered …

R: Yeah.

I: Yeah.

R: I have.

00:26:14

I: Have you used them yourself? Or have you seen others use them?

R: I haven't used one myself, but I've seen people use them.

I: Personally or, like, on the TV? Or both?

R: No, I've seen on the TV, I've seen personally. I've seen personally.

I: Okay. So you're probably aware that drones, like, are small, flying aircraft, they're piloted remotely …

R: Yeah.

I: … and they have camera …

R: Yeah.

I: … capabilities so they can navigate and see where to go. So they can carry defibrillators by landing on the ground and then detaching the defibrillator, or they can hover just above the ground and winch …

R: Okay.

I: … the device down to the ground.

R: Okay.

I: So, kind of, like, the winch is a kind of rope, object, as it were. So, yeah, so they can land and detach the defibrillator or they can hover above the ground and winch the device down. So can you imagine interacting with a defibrillator in the ways that I've just mentioned? Or what are your thoughts?

R: (Pause) Can you go over again?

I: Yeah, sure. So I'm just wondering what you think about a drone carrying a defibrillator and then landing on the ground and detaching …

R: Okay.

I: … the device. So can you imagine interacting with the drone in that way, picking up the defibrillator from the ground after the drone has detached it from itself? Or perhaps can you imagine interacting with the drone by, and getting the defibrillator if the drone hovers above the ground, so if you can imagine that, and then it winches the defibrillator down to the ground by a, a rope?

R: I'd say I would prefer the drone winching and bringing it down, bringing the defibrillator down by a rope, 'cause that would be a bit safer than it landing with a defibrillator down. 'Cause it could land on a hard surface, could land on a hard surface, causing a damage to the defibrillator. So I would prefer it winching with a rope slowly. With that way you know, with that way you know it's a bit, should I say, careful with the defibrillator.

I: Yeah. Oh, thank you for those insights. That was really interesting. So I think you've touched on this already, but perhaps you want to say a bit more about it. How would you feel about a drone bringing a defibrillator to you whilst helping someone having a cardiac arrest?

R: Well (sniffs and sighs heavily) I would feel elated, elated. Use the word 'elated' 'cause that is a very fast response and something coming to your rescue at a very fast pace. So I would feel elated.

00:29:59

I: So do you think it would make it easier for you to use a …

R: Yeah.

I: … defib? Yeah.

R: Yeah, it will, definitely.

I: And is that because of how quickly it would get the defibrillator to you?

R: Yes.

I: Is there anything else maybe that you can think of that would make it easier for you?

R: (Pause). Not that I can think of.

I: Okay. I just wanted to clarify that it's the, the quick response time that would make it easier for you and help you in that, in that challenging situation. Okay. So how do you feel about leaving the person, if they are by themselves, to get the defibrillator from the drone?

R: (Breathes out) Okay. That’s a very sensitive question.

I: You don't have to answer it if you don’t want to. We can move on. It's up to you.

R: It's okay. It's okay. I would feel not so good, to be fair, 'cause at any point you leaving the person could be the person's last moment. And, and again, you leaving the person, you're not just leaving the person but you're going to get (louder) *what* will help the person come out of that situation. So it's a two-way thing. It's just a two-way thing. You do not know if, if you leave this person, that could be the person's last moment. And you leaving this person, you're not just leaving the, the person but you're going to get what will help resuscitate this person. So it's really a sensitive question.

I: It's difficult. You can't really say. There's pros and cons. Is that what …

R: If there's …

I: … you mean?

R: …  a way that, if there's a way the drone could come into the house (laughs), if there's a way … if, if it, if it means keeping the door open for the door, the drone to come into the house, I, that would be really great. That would be really great.

I: If that wasn’t possible, would you be prepared to leave the patient's side to get it from the doorstep?

R: Yes. I would say yes, hurriedly. I will do this hurriedly.

I: What about over the road? Or is that, would that be too far for you?

R: Over the road, over the road, over the road, it's (breathes out) quite difficult. It's quite difficult.

I: And would location affect your thinking on this? So if the, if a cardiac happened outdoors, would you change your mind or would you still be reluctant to leave the person's side?

R: Sorry, I didn’t get that.

I: I'm just wondering if location would affect your thinking on how close you would want the drone to deliver the defibrillator to you.

R: If my location …

I: Was, so say you, you had to assist with a cardiac arrest that happened outdoors …

R: Yeah.

00:33:48

I: … in a public space …

R: Okay.

I: … would your thinking change on how close you would want the defibrillator to, on how close you would want the *drone* to deliver the defibrillator to you?

R: Okay. If it was in an outdoor space?

I: Mmm hmm. Yes.

R: Since …

I: Sorry? Are, are you …

R: It's an (loss of audio) possibility of (loss of audio).

I: Sorry, the connection's …

R: (loss of audio)

I: Sorry. I'm really sorry. The …

R: Sorry about that.

I: … connection's dropping out. No, it's not your fault. It's, I, I didn’t get your response. Would you mind starting again, if that’s okay? I, I didn’t catch it.

R: Okay. You talked about … sorry. You talked about outdoor space and if my decisions would change if a, a drone was delivered to me in a situation where it's an outdoor space.

I: Yes. How close would you …

R: Right?

I: … want it, yes, to be delivered to you in that situation?

R: Okay. I'll say very close. If it's an outdoor space, I would, I would, I would prefer the drone delivering it very close, very close to me.

I: And may I ask how you felt about letting the ambulance crew in when you were assisting with your father-in-law? Did you have to leave his side to let them in?

R: Sorry?

I: With your, when you were assisting your father-in-law, did you have to leave his side to let the ambulance crew in?

R: Did I have to leave his side when the ambulance came in?

I: Yeah, did you have to leave his side to open the door to let them in?

R: Okay. Okay. The door was open.

I: So …

R: I left it open.

I: Yeah. So you didn't need …

R: I left …

I: … to leave.

00:36:24

R: I left it open.

I: Yeah. Okay.

R: 'Cause I knew they were coming, so I left it open.

I: Yeah. Sure. Okay. So I just wondered what you thought the role of the call handler was in drone-delivered defibrillation.

R: (Pause) You just wondered what the call handler was?

I: Yeah, the role. What, what is their role? When we're talking about drone-delivered defibrillation, what is their role …

R: Yeah.

I: … in assisting and facilitating with this.

R: Okay. In that I think it's, so if we educate the person on how to use a defibrillator when it's been delivered, that is if the person do not know how to use it, think your role is just to tell the person, give directives on how to use it.

I: Okay. So you'd want to know how to use a defibrillator. Would you want to know anything about the, the, the drone? So did you, would you want to know that one was coming or only when it was there? Or how to identify the drone that was carrying the …

R: Yeah.

I: … defibrillator?

R: Yeah, all that.

I: All of that.

R: Yeah.

I: And I know you said that you'd be very reluctant to leave the patient's side if you were by yourself with them. If there was, say, another person there, how would you manage to get the defibrillator from the drone if there was another person with you?

R: If there was another person, then I would go get it. I would go out to get it myself. 'Cause I know there's someone there with him, because I know there's someone there with him. So I would go out to get it.

I: Yeah. Sure. Okay. And what concerns, if any, do you have about drone-delivered defibrillation?

R: What concerns?

I: If any, do you have about this technology?

00:39:01

R: Okay. I wouldn't say I have much concern, except for maybe bad weather, 'cause I (loss of audio). Yeah, (inaudible) bad weather.

I: Yeah.

R: The weather will definitely affect the, a drone delivering a defibrillator.

I: Okay. And any other … sorry?

R: Yeah, I said the weather condition can significantly impact the effectiveness of a drone, of a drone delivery.

I: Yeah. Sure. And when you say that, do you mean that it would be difficult to get, to fly? Do you mean it'd be difficult to fly, or do you mean it would be difficult to get the defibrillator to the ground? Or none of those? Or all of those? How might the weather affect that?

R: None of the, none of those. And I do not know how the drone works. I did not manufacture it. But I think when it's raining it might affect it, or wouldn't it? And I also know when it's night, it will be quite difficult to, it would be quite difficult for a drone to deliver something at night 'cause everywhere will be dark. And also a poor temperature, it, a very hot temperature could also affect it …

I: Yeah.

R: … be overheating or …

I: Yeah. Sure. Any other problems or issues?

R: Maybe snow too. Snow could also affect it, wouldn't it? Snow could also affect the drone.

I: Yeah. So these are things, these are issues or potential problems to consider. Yeah.

R: Yeah.

I: Okay. Excellent. Thank you for those insights. So I just have some final, brief questions that may be useful for our research. So if I may ask, what is your age and gender?

R: Okay. I am 29 years old. Okay, I will be 29. I will be 29 by (date). I'll be 29 by (date). I am a female …

I: Okay.

R: … a woman.

I: Thank you. And have you had training in CPR and defibrillator use?

R: Sorry?

I: Have you had training in CPR and defibrillator use?

R: Yeah, I have. I had that after the incident with my father-in-law, 'cause it seemed I wasn't doing it properly. That's why he didn't respond until they got there. So I had to go for that.

I: Okay. So when, when was this? When did you have the training?

R: Okay. That was in [year].

I: And was it CPR only or CPR and defibrillation?

R: Just CPR.

I: Okay. And it happened after the event, the incident.

R: Yes, after the event.

I: Okay. Is there something else you would like to add to your answers today?

R: (Pause) None I can think of.

I: Okay. Well, thank you. I'm just going to turn off the, the audio recorders and just go through what will happen next.

**END OF INTERVIEW**

Participant 4

I:  ... on. Excellent. Okay. So, the audio recording has begun, and it will continue until the interview questions end. So, thank you for agreeing to participate in this interview, which forms part of a research study being completed by the University of Warwick and Welsh Ambulance Service NHS Trust. Before we can proceed with the interview, I need you to reconfirm your eligibility, please. So, can you confirm that you are aged 18 or over?

R: Oh, definitely. I'm 25.

I: Thank you. That you are comfortable to proceed with the interview in English?

R: Yes, I am.

I: That you have provided assistance at a cardiac arrest at some point in the past?

R: Yes, I have. My uncle, actually.

I: Okay. And we'll explore that in a little bit. And that you agree to have an audio recording made of your interview?

00:01:11

R: I agree to that.

I: Thank you. So, I've received a signed consent form from you. Has anything changed since you signed the consent form?

R: No, not at all.

I: Thank you again for confirming that. So, the interview will last for around 45 minutes, and your participation is entirely voluntary. You can pause, reschedule, or terminate the interview at any time, without providing a reason, and without your rights being affected. And you can choose to have your camera on or off, it's your decision. So, there are no right or wrong answers. We are interested in your experiences of helping someone who had a cardiac arrest, and your attitudes around using drones to deliver defibrillators. Do you have any further questions about the study or about the interview process before we begin?

R: Well, no, nothing really comes to mind right now.

I: Okay, that's fine. If any do, just let me know during the interview, or feel free to email me afterwards. So, are you ...

R: Okay.

I: ... are you okay to proceed?

R: Yes, I'm okay.

I: Excellent. So, can you please tell me as much as you can about when you provided assistance to the person, which I'm assuming is your uncle, who had the cardiac arrest?

R: Okay. So, I'm very close with my uncle, and he is into [hobby], and it was something I was really interested in. So, he and I really, you know, bonded a lot over that. I, I'm trying to give context to the story, so, sorry. So, we live in [Name of area], and we were going to the [Name of transport system] 'cause we want to get to somewhere else. And, all of a sudden, it's like he just seizes, freezes, and falls. And it, it was really scary. The only thing I really think that helped me, was that in school, one of the things ... I went to private school, and I think one of the reasons, they were very particular about, every student must have, must be trained in basic first aid. So, that's everything from CPR, to the Heimlich manoeuvre, to how to respond when someone is having a seizure, you know. The basics. So, I, I didn't know what was going on, really, but I knew it was heart related. So, I just immediately started administering CPR. And then I called emergency services, and I told them what I saw, and what was going on. I was still performing compressions. Someone who saw what happened had actually, like, called, and they were like, "Okay, let me talk about what's going on."  And then they were like, "He's probably having a cardiac arrest." And that since we're probably in, like, already in the [Name of transport system], there's what they called a public access defibrillator. And they, like, gave me the instructions. Although the instructions, kind of, were on there, but what to do, how to use it, and, like, I gave them the address of where I was. I performed compressions, compressions, defibrillated. Just kept on performing compressions again, then defibrillated. And I think, luckily, like, by the time I had done the second defibrillation, and I was doing compressions, they got there, and they, they took over.

I: You mean the, the ambulance, the emergency service?

R: Yeah, the, yeah, the emergency services. It was really scary.

00:05:10

I: Yeah, I can ... Oh, I'm sorry to hear that. So, just to confirm, it was your uncle who suffered the cardiac arrest.

R: Yes, it was.

I: Yeah. Okay. And it happened, was it on the [Name of transport system], or was it at the station? Or just outside?

R: It was at the, it was in the station. We were about to board.

I: Oh. Yes. Yeah. Okay. So, you said that you knew it was something heart related. Did you realise it was a cardiac arrest, when that other person said that it was a cardiac arrest? Or had you realised that before they'd said that to you?

R: I knew it wasn't a heart attack. So, I, I, I just knew that there was something else wrong with the heart. Only 'cause one of the things we learned was, like, the difference between, like, a heart attack, and another heart related event. You don't necessarily have to be unconscious to have a heart attack, and, like, you know, numbness in your ... Is it left or right arm? I really, I always mix it up. I really do need to learn to remember that. But he was unconscious. He, he wasn't conscious, so I, I, I think it was just a gut feeling, really.

I: That you, that you thought it has to be a cardiac arrest?

R: Yeah, because he's, kind of, he's, like, in his [age range]. So, I, I, I thought it was a healthy assumption ...

I: Yes.

R: ... 'cause, you know, by that age, it starts being stuff about, "Oh, be, be careful with your heart, cholesterol, la de dah." And I did check his pulse. I, I, I checked his pulse. So, I, I also think that was one of the major signs that, for me, it was heart related.

I: Right. Okay. Okay. So, what was the outcome?

R: Oh, luckily, he survived. They said that, but we were also told that without the emergency reactions that I did, it may not have been such a positive outcome. I never actually knew, until that incident, how fatal cardiac arrests could be.

I: Yeah. So, it's ...

R: I, I never actually knew how critical that situation was, until he got better.

I: You said that you monitored his pulse. What had happened to that, then? Had…?

R: It, there, there, it was, like, there was no pulse. Like, it was, like, nothing, you know. I checked his wrist. I checked his neck. It was, like, nothing.

I: Okay. So, you said that you, there were other people there. So, were they strangers, or were they family members, or friends?

R: Total strangers, actually.

I: Oh, okay. So, they came when they'd seen that your uncle had unfortunately ...

R: Yeah.

I: ... collapsed, so they came to assist. How did that make you feel, them being around?

R: It was a mixed feeling, actually. I, I have social anxiety, so I, I don't do well with a lot of people. But at that point, I also knew I needed all the people I can get. Because I can't, I can't do the chest compressions while talking to emergency services, while they were also asking me to get access to a defibrillator. I knew I couldn't do that all by myself. So, I was very grateful at the same time, but it was, it was a, an unconventional, and a bit uncomfortable situation for me.

00:09:14

I: And that ...

R: But, but not, but not because of the assistance, just more of a personal quirk.

I: Because of the anxiety that you experience, yeah. Okay. So, the other people, the, the strangers that came to assist, what did they do?

R: One called emergency services. And, like, he was with his wife. She went to ask someone around where the closest defibrillator was, and brought that over. And she just really stayed by my side, and kept on soothing me, like, "You can do it. You can do it. You can do it." Just giving me, like, motivational support.

I: How many ...

R: Because ...

I: Sorry.

R: Well, people had gathered, but, like, direct assistance, about three.

I: Yeah. And what were you going to say, sorry?

R: I, I know this is an aside. I was very afraid that I was doing the chest compressions wrong.

I: Yes. Yeah. And we'll look at your experiences of performing CPR in a moment, if that's okay.

R: Okay.

I: I just wondered ... So, the, the strangers who were there, how, how did they organise themselves? And I think you've, you've answered this, but just in case there's something more to add.

R: (Pause) One thing I noticed is that, like, they tried not to crowd me, 'cause I, I think, I think maybe they have experienced this type of thing before. But their response to it, it's like they were more familiar with this type of scenario than I was.

I: Interesting.

R: So, it's, like, they, they, they, they gave me space. The woman kept on assuring me that I was doing okay, that I should continue with the chest compressions, while the husband called emergency services. She already, kind of, knew they would probably ask for a defibrillator, so she had already asked. So, then they were like, "Oh, there should be a public access defibrillator around." So, but she had already run off to get that. (Chuckles)

I: Oh, thinking ahead. Yes, yeah.

R: And I think it was that thinking ahead that really made the difference, to be honest.

I: So, there were three people, the husband, and the wife, and then the third stranger. What did they do? How did they organise themselves?

R: Funny, they, they were just there to support in whatever capacity. I really can't remember doing the, them doing something specific. I think they just, kind of, saw how panicky the situation was, and they were like, "Okay," whatever they can do to help, they can, you know, help.

00:12:28

I: Yeah. Yeah. So, offering you reassurance while you were performing the CPR. Okay. So, can you tell me as much as you can about the 999 call?

R: (Pause) Oh (pause) I had to, like, really compose myself. 'Cause I was really stuttering, and just saying, I was just really all over the place. And then, I was like, "Okay, I should take a deep breath, and tell them what, what, what's going on." And I really couldn't compose myself that well at first, but then I was able to tell them what had happened. And then they were like, he had fallen down, I'd checked his pulse. Nothing was there. By that time, I had started chest compressions, and the lady's husband was holding, you know, the phone. So, he, kind of, took over the calls, as I had said, like, what had led up to that specific incident. So that I could just majorly focus on, like, the chest compressions.

I: So, who'd phoned the, the call handler to start with?

R: No, no, I had.

I: You had, okay.

R: I had. I, I had ... I, I called 999, I put the phone on. I had started chest compressions, someone saw I was talking, they helped pick up the call. It was really such a messy, yet organised situation.

I: Oh, not at all, you know, everyone was doing their best in what was a very difficult situation. So, presumably the phone you used to speak to the call handler was your mobile?

R: Yeah ...

I: Yeah.

R: ... it was my, it was my phone.

I: Okay. And when the husband was talking to the call handler, did he put it on speakerphone? Had it already been on speakerphone?

R: Oh, it was already on speaker, and he left it on speaker. He was able to help provide more information, like where exactly we were. And that's one of the things I remember that he was able to provide. Like, where exactly we were, and what he observed. To be honest, I really don't remember much of what he said. I, I think by that time, I was really just focused on my uncle.

I: On your uncle. Yes, yeah. Understandable. So, did you leave the patient to make the call, or did the husband leave the patient to make the call?

R: No, he was still there, kind of, like, I'm sitting here, and maybe, like, two feet away, or a foot and a half. (Demonstrates on camera)

I: The, the husband. Yeah. Was ...

R: Yeah.

I: Yeah. Yeah. Okay. And I know you say that it was very chaotic, and busy. And, you know, if you can't remember, that's absolutely fine. I'm just wondering if you can recall anything that the call handler said, or what help the call handler offered?

R: (Pause) By then, I had already just started the chest compressions. To be honest, I didn't know if the chest compressions would help. I just knew that, it just seemed like the default thing to do. So, they asked, "Oh, are, are you performing chest ..." That I should perform chest compressions, and if I can find a public access defibrillator, I should. But since I'm in a [Name of transport system], actually, there should probably be one around. I had already started, so they were like, "Oh yeah, you're doing great. You're doing great." Then they told me to do compressions for at least 30 to 60 seconds, then pre-set the defibrillator. And, like, when (?) it's in place, they, they gave the instruction, and that ... Then, if that still doesn't help. I should, I should repeat that step, and keep on repeating it, till, like, emergency services show.

00:16:44

I: Okay. So, quite detailed help, would you say? Or, or how do you …

R: I'd, I'd say as detailed, I'd say as detailed as, as possible. But, but, but to be fair, I, I think I felt a bit clumsy with the defibrillator. Because I was like, "Oh, oh, sorry ..." I just kept dropping it. (Chuckles/slight laughter)

I: Okay. Well, as you say, the outcome was positive, and your, you know, quick intervention, you know, was useful, you say. What are your reflections on how the interaction with the call handler went? Did you find, did it, what went well? What was difficult? Were there any issues?

R: (Pause) Well, I, I think it went well. But to be honest, I, I don't … Okay, this is the thing. This is a really sensitive situation. And I, I think at the point, without their help, the people that were there, I felt, I felt a bit stretched thin. It's like trying to talk to someone, and try to do the lifesaving act at the same time. It just felt a bit clumsy, but I don't think that is necessarily, you know, their fault. I think it's just the reality of the situation. It's like, I felt like I had to be in two places, listen to the instructions, and get them right. Then apply them, so my uncle doesn't die.

I: So, are you implying that the call handler wasn't aware of those difficulties, or didn't help you try and manage them? Or are there any, or there, there's no implications, you're just describing what happened?

R: I, I think I feel, like (pause) may, maybe some, some type of (pause) I'm, I'm thinking of the right way to phrase it (pause) they provided the information, and they provided it well. I, I just think that maybe it could have been provided faster, with the gravity of the situation. I, I, I, I feel like, okay, yeah, okay, she was trying to provide that information in a way I could grasp. But I'm, like, at the same time, I think, my uncle here doesn't, apparently, he didn't have the luxury of time. Apparently, quicker responses save better. And frankly, even with my knowledge, it, it was the couple that really still made the difference for me.

I: Okay.

R: The, like, the woman, like, just know, knowing that a defibrillator may be needed, or, or the husband taking the phone from me so I could focus on what I was doing.

I: Okay. So, the practical support from the couple ...

R: Yeah.

I: ... you found beneficial, but the call handler, the way that they were giving the instructions, it was quite slow. Is that, is that what you mean? That they ...

R: A bit, a bit, yeah. A bit, yeah.

I: Okay. So, would you have wanted them to give the instructions in a quicker way? Or not so detailed way, or ...?

R: Quick and, I think more, like, precise. Like, this is what you need to do. Do this, do this, do this. Especially with something so sensitive.

I: Okay. Whereas, the call handler was being more ... How would you describe it?

R: (Pause) Should I say conversational?

I: Okay. If that's how you feel it went, then absolutely.

00:21:16

R: Yeah. I, I, I didn't need a conversation, I needed instruction. I needed direction. And she did give the direction, but I needed ... It's, like, it was later I realised how time sensitive that situation could have been, without, like, immediate response.

I: So, when you say conversational, do you mean, like, she was asking how you were? Had you completed a step?

R: Yeah.

I: If you were alright?

R: Yeah. Just give me the instructions. Yeah, and I'm, and I'm not saying that it's, it's bad. Maybe it's my anxiety, that maybe made me feel that way.

I: Okay.

R: I don't, I don't, I don't know.

I: If that's how you feel. And that's how you experienced it. Then that's fine. That's what we're here to do, to see how you experience that. Did you, what, did the call handle ask you any questions? Or the husband?

R: (Loss of audio) Question (inaudible) she asked how long it had been since the, like, the difference in time between him falling, and me calling. She was like, "What's the time difference?" I remember her asking that. If I had checked his, that I should check his pulse if I hadn't. I think she probably asked the husband more questions. Well, those are the two major questions I remember asking. I remember being asked, sorry.

I: Did you ask the call handler any questions, or did the husband ask any questions?

R: (Pause) The only thing I remember asking is, "What do I do? What do I do? What do I do?" (Chuckles)

I: Yeah. Yeah, understandable. Were, were there any technical issues, while you or the husband were speaking into the call handler on the phone?

R: Oh, no.

I: No.

R: Not at all.

I: No. Okay. So, you said before that you performed CPR. Can you tell me as much as you can about this, please?

R: (Pause) Well, when I started CPR, I, I was literally playing in my head the, the instructions from when I was in school, about, like, how to position your hands, where to position your hands. How to perform the chest compressions. I did try mouth to mouth, but I, I usually thought that's for, like, drowning, but I still did it. But it was majorly the chest compressions for 30 to 45 seconds, then mouth to mouth. Then by the time they got the defibrillator, it was chest compressions, then the defibrillator.

I: Okay. Okay. So, what ...

R: Well, they were, like, they were, like, "Under ..." They were like, under no circumstance should I, like, break the flow. I should just keep going, like, there shouldn't be a pause, you know. It was, kind of, like arm exercise, looking back. (Laughs) My arms were sore (laughs) after the whole, after the whole situation, 'cause it did go on for a couple of minutes, so I just had to keep on.

00:25:09

I: Yeah. Yes, yes. So, what are your reflections on how performing CPR went?

R: (Pause) Considering my, my uncle is alive, I'd say I did okay.

I: Yeah (laughs slightly) Yeah. But you found it tiring, you say. Yeah.

R: Yeah.

I: Yeah. Okay.

R: I've never had to do it for that long. The, the last time I performed CPR was, like, five or six years ago, and that's because my cousin had, cousin had a drowning incident in a pool, and I performed CPR. That, that was literally the last time, and I, I didn't have to perform chest compressions for that one.

I: Yeah. Okay. And you say that the call handler gave you instructions? Yeah.

R: Yes, they did.

I: Yes. Did you want to reflect any more on the instructions or advice that they gave you?

R: No, no. No.

I: No. Just that it, it was a bit too conversational, you felt. Yeah. Okay. So, you say that you used a defibrillator?

R: Yes.

I: Can you tell me as much as you can about using the defibrillator?

R: (Pause) I think that's actually where my greatest fear was. I think my fear was that I would mistakenly set it wrong, and end up doing more harm. So, even as I, as I set the (pause) I'm gonna use the word parameters, as I set it to what they asked me to set it to, and I, I kept rubbing the panels together, to charge. I was like, "Lord, let me get this right. Lord, let me get this right. Lord, let me get this right." But I was, like, saying that in my head, and I kept on checking to make sure I had set it to the right setting. I remember I kept on checking the numbers, and I kept on repeating them in my head to make sure, And then I'll check again that the (inaudible) are right. Did I get it right? Okay. Yeah, I got it right. Then, but you know, when you watch all the movies, you just see them using the defibrillator, I mean, like, "Clear."

I: Yeah.

R: (Louder) And they make it look so easy. They, you don't realise how much of a jump it takes out of you, to even just, clear.

I: Yeah. Yeah. It's, it's difficult, isn't it? But the, like you say, in the media, it's portrayed as a relatively simple, uncomplicated process.

R: Yeah, you just see the paramedics, you just see the doctors, and they're like, (dramatic) "Oh, set it to 150 over this ..." Setting it to 150, and you’re whacking it together, and like you hear, "Clear." Okay, and they're checking, and I'm like, "The movies were wrong." (Laughter)

I: Yeah. That's not what happens in real life.

R: The moment, you, you don't realise how much physical exertion comes into the whole thing.

I: That's interesting.

R: Like, you literally almost feel, you, you literally, kind of, feel it. Like, like, the jerk back.

00:28:29

I: Yeah.

R: Kind of, like a weird, kind of, like a weird whiplash, of a sort.

I: Interesting. That's, that's, that's how you experienced it. That's interesting. Yeah.

R: It was kinda like a ‘ooh!’ I think I'd, I, I was, I think ... Well, it's not necessarily a bad thing. I think it was based on movie portrayals, thinking it was just gonna be something so simple. And I'm like, "Oh, I did not expect that to happen." So, by the time I did it again, I didn't really feel it. I think it was just unexpected.

I: Yes.

R: The first time was where the, like, the jerk back really was. And I'm like, "Okay, this is not the way it is in the movies. Okay. Okay. So, this, this is how we know it's going to be. If we need to do it again, well, we're set, we're prepared. We're sturdy. We're gonna do it again." (Chuckles)

I: Yes. Yeah. And you're having to learn that, as you were doing it.

R: Learning on the go.

I: Yes. Yeah. So, how easy would you say it was, was to use the defibrillator, and understand how to use it?

R: (Pause) The, the only part I, I, I think I found a bit tricky, and I think that's basically me being panicky, was, like, the settings. Like, what I'm supposed to set it to. Apparently, they are different, like, defibrillator settings, like, the numbers. Like, 150 over this ... I can't remember which number they asked me to set it to, but I, I kept on, like, being a bit clumsy with it. But I think that was more of panic. I think that was a bit more, more the panic, than maybe ... There should be, like, an easier keypad, where you just press the number. Press the number you want, press it. Press the, like, kind of, like a Nokia keypad, where you just press it in. (Laughter)

I: Yes. That would have made it easier for you to use. Yeah.

R: Probably, yes. I think that, that's the only major hiccough. Because I feel like you shouldn't have to worry about learning new technology in that, of that gravity, because then, what if you're not able to get it right. What happens then? I, I get that, I feel like the defibrillators should be as much as they're public access, they should actually be *public access*. Something that any random person can easily see, and, like, even if they don't have as much direction, they can, like, try and figure it out on the go.

I: Yes. Yeah. Yeah. So, you're saying that they're not very accessible, but when you do have one, they're not very easy to use. Is that what you mean?

R: Yeah. Yeah. Yeah. They're accessible physically, but not accessible in, necessarily, in the application of, like, using it, sometimes.

I: Yes. Yeah. Okay. So, given those issues, how comfortable did you feel using the defibrillator, if at all? (Chuckles)

R: I knew it was something I had to do. I just kept on praying to the heavens that I was doing it right.

I: Yeah. Yeah. How easy was it to follow the defibrillator voice instructions?

R: Funny, I think that was the part that actually made it a bit easier for me. I'm, I'm a, I'm, I, I learn by listening. I love reading, but I assimilate better, like, listening to stuff. So, the voice part actually helped me a bit more, especially when it was, like, in line with what emergency services had said. So, it's, kind of, like, "Okay. I've heard it twice. Okay. We, we can figure this out."

00:32:26

I: Were there any times when the instructions from the defibrillator were not in sync with the instructions from the call handler?

R: Oh, no. Well, not for me, anyway, no.

I: No.

R: Quite in line, actually.

I: So, had did ...

R: Has that happened to someone before?

I: I'm, I'm just exploring whether it happened to you.

R: Oh, I, I, I would panic if that happened.

I: Yeah. Yeah.

R: That would be very freaky. 'Cause I'm like, "Okay, who do I listen to?" (Laughter)

I: Yes. Yeah. Yeah. I think it's been reported in the literature, sometimes people might be, you know, might struggle a little bit. But some have other experiences, so I'm just seeing what experience you had. So, you found following the defibrillator and the call handler at the same time relatively easy? Is that correct?

R: Well, well, relatively easy, yeah.

I: Given the situation.

R: Yeah.

I: Yeah.

R: Given the situation, I think everything should always be in brackets of, "Given the situation."

I: Yes. Yeah. Relatively speaking. So, how did you find the call handler’s instructions, regarding how to use the defibrillator?

R: They were (pause) they were very explanatory, but unlike the defibrillator, she would want you to, like, she would give you one instruction, then you do it. But the defibrillator, kind of, just, like, kind of, I think the, the voice instructions were faster than her instructions. She was like, "Put it on. Have you put it on?" "Yes." "Have you done this?" "Yes." So it was, kind of, like, a guided procedure?

I: Whereas the defibrillator just went through the motions, it wasn't checking whether you'd put it on the, on your uncle, and ...

R: Yeah.

I: Okay. Okay.

R: I, I do have a question, though. It's something that just popped up in my head. Is there an option where, with these machines, you can repeat the instructions if you don't, like, hear them right?

I: Repeat, ask the defibrillator to repeat the instructions? I'm not entirely sure about that, actually. But I can get back to you, afterwards, if ...

00:35:00

R: I mean I'm just thinking that maybe that should be an option, if it's not an option already. Because I just realised that I, I may have gotten it right at first, but not everyone would.

I: So, you'd want a button on the defibrillator that you could press, so it could repeat, go through it again?

R: Yes. Like, what, it, it doesn't necessarily have to be, like, all the instructions. Like, let's say there are five instructions. You can press, like, you know, the way I said, like, the Nokia buttons? You press button two if it's just the second instruction you missed. You press the third one, if it's just the third one. Or if it's just the last instruction you didn't get one, you just press the, like, the fifth button, since that's, like, five instructions.

I: Okay. So ...

R: It's just an idea, really.

I: Yes, absolutely. That's, to make it more accessible for people. Well, that's what this project's about, is looking at the barriers and facilitators.

R: Yeah. Instead of it, like, go, starting from the beginning, and taking all that time, like, whatever specific instruction you didn't hear, you're just able to press a button. Like, maybe the instructions are already numbered in the software. So, if you press button two while it's listing the instructions, you know it's the second one that you wanna hear again.

I: That's very interesting. Yes, some food for thought, at least. So, the defibrillator was on site, it was in the train station. And had the husband brought it to the scene, did you ... Or the wife, did you ...?

R: It was, it was the wife, it was the wife. Very sweet lady.

I: Where was it in the train station? Was it, did she have to go up escalators, or was it behind you? Or did you have, did she have to ask the, one of the train guards?

R: She asked one of the train guards. I think it was hung up a bit down the line. I, I really never actually saw where the defibrillator was hung up, I wasn't the one who picked it up. But I do remember her like running down, like, straight down, and then, kind of, running, and then she was just holding this like box.

I: Yeah. Yeah. So, she ran down the platform and then ran back, but you stayed with the patient, with your uncle at all times? Yeah. Okay. So, she didn't have to go too far, presumably?

R: No.

I: No.

R: She didn't have to go too far. She was back in about 90 seconds to two minutes.

I: Okay. And what are your thoughts about ... Sorry, did you have something else that you'd like to add?

R: Oh no, oh no. No.

I: Okay. What are your thoughts about using a defibrillator in the future?

R: I do like, I may not like how I got the experience of using it. But I am happy I have used it before. And I feel like my response time in the situation will definitely be better. It may be unfortunate how I got the experience, but I think this is one of the places where experience is a good teacher. It sounds a bit morbid, but in, at least I know what to do, if this ever happens again. I mean, I would still probably call emergency services. I would still probably want people to help me. But I know what to do. I feel like I probably need less guidance. I'll probably panic less, and probably be less anxious, and be less fidgety, because this is not my first rodeo. But I immediately know what to do in this incident.

00:38:57

I: Yeah. And you touched upon it before, when you said you'd like the ... Can ... Sorry, can you hear me? 'Cause you've frozen. (pause) Hello? Can you hear me?

00:39:38

(Recording paused and resumed)

I: Okay. So, I've just turned the audio recorders back on, and we are recording again. And you're sure you're okay to proceed?

R: Oh, yes, I am.

I: As long as the technology holds out this time. (Laughter) So, I think I was just in the process of asking you whether there would be anything else to make it easy, or easier for you to use a defibrillator. I know that you mentioned about the, have it, you know, the defibrillator being able to repeat instructions. Was there anything else that can make it easier for you to use an AED?

R: Well, frankly, no. I think that was the major hold up for me.

I: Oh, what, sorry? I didn't quite catch that.

R: I said I don't think so. I think that was just a major hold up for me.

I: Yeah. Okay. That's fine. I, I just wanted to check whether there was something else to add. But your answers have been very comprehensive, so thank you. So, the next part of the interview, I know we're running over time now, so, but are you okay, you're sure, to continue with the next bit of the interview?

R: Well, I still have about 10 minutes.

I: Okay.

R: So, go ahead.

I: Okay. Thank you. So, I'd want to look at, now, your attitudes around defibrillators being delivered by drones. So, firstly is this something that you've heard of?

R: No, this, that, this is relatively new to me.

I: Yeah. Okay. That's understandable. So, have you heard of drones, first of all?

R: Oh, yes, I, I have a younger brother who's obsessed with them.

I: Okay. So, have you, so have you seen drones used, or have you used one yourself, or, and in what situation?

R: I have toyed around with one, but mostly I see them used for, like, aerial shots, like, photography.

I: Can you say anything else about drones? How, how you understand them?

R: I, I, I just see them as, like, small mechanical devices that can fly and, but the, you need to direct them to where, specifically, you want them to go.

00:42:13

I: Okay. So, how do you imagine a defibrillator that has been delivered by a drone works? So, how might it get it to the person, the bystander who needs it?

R: (inaudible) Can I tell you the visual I have in my head?

I: Please.

R: It kind of reminds me (loss of audio) ...

I: Sorry, you're dropping out again.

R: ... of, like, you know, when (?) the ones that deliver babies. I think it's weird, because I'm using ... Also that I, kind of, have this visual where it's, like they're, stork deliver babies. Like, in, like, the white sheets, and they just, kind of, drop them off at the new parent’s house.

I: Yeah.

R: (Laughs/giggles) So, I, kind of, imagine, like, the, the drone holding the defibrillator, kind of, like this way the stork is holding the baby. Just, kind of, drops it off where it's needed. (Chuckles)

I: Okay. So, you can imagine it as landing, and then detaching the defibrillator? Or hovering above the ground and winching it down? Can you imagine a defibrillator being delivered to you in one of those ways?

R: (Pause) To be honest, I think it's a bit complicated. Because I feel like people today have this weird thing about drones. They're either trying to lock them off, or, like, just follow them, or ... This unhealthy fascination. So, I do not think that a lot of people have enough sensitisation to know that this is a device that's delivering a medical, that delivering medical equipment. And I feel like it creates a lot of room for inaccuracy. If people could understand that it's, like, and this is, like, a medical emergency, that this is delivering a medical device that's needed for a critical situation, I feel like it would be very effective. Especially with places that don't always have, like, public access defibrillators. It will probably get there faster than emergency services. But people can be weird about drones, and I feel like, to a large extent ... (loss of audio)

I: Sorry, you've dropped out again.

R: ... (loss of audio) that impacts the person that need ... Oh, sorry, can you hear me?

I: No, it dropped out when you said ...

R: Can you hear me?

I: ... about other people might not like defibrillators being delivered by drones, or ... Could you go through why you think that?

R: I was saying that people can be weird about drones, generally. And I think that's because people have misused ... So, when people would see a drone, they wouldn't necessarily see it as a drone carrying a medical device. They will probably just see it as, someone is, like, trying to be cheeky with a drone. And they, and they just try, like, to disrupt it.

I: Okay. So, you think there'd be misperceptions about drones, and what they can do. And people wouldn't really trust that a drone could deliver a medical equipment?

R: Yeah. I feel like there would need to be, like, public re-information concerning how to identify a medical drone, drones delivering medical equipment. So, they know that this isn't someone being ... Like, people, like, would be, we would need to know about, like, medical, drones delivering medical devices, how to identify them. The same way they know to call 999, or 112. It needs to be that identifiable.

00:46:05

I: Interesting. Really interesting. And can you say anything about how you would get the defibrillator from the drone?

R: Well, I, kind of, imagine, like, the drone dropping it off. And then, like, maybe, like, it's in a package, and then you just remove the drone from the package, and then you bring up the defibrillator.

I: And how would you feel about using a drone delivered defibrillator whilst you were helping with a cardiac arrest?

R: It could be delivered by the Archangel Michael, for all I care. I just needed the device, you work on the transportation and delivering (chuckling) I really don't think I'd have an issue with how it's delivered. I think I just really need it delivered.

I: Do you think it would make ...

R: I, I think (loss of audio) but if you do eventually use drones, there should be a way for, like, the person that wants to access it to track how far that, the drone has gotten. And how close the drone is to their location.

I: Would you want to be told this information from the call handler, then?

R: I think that would be wise. Because this is not information like you see, you teach in schools where everyone is aware of. So, I do think it should be the call handler’s responsibility.

I: To tell you when one's on its way, and that it was coming, and ...?

R: Yeah. Well, there should, kind of, be like an app where you like type in something, so you can see how far it is.

I: Would you prefer an app over the call handler then, or would you have both, or ...?

R: I think I'd rather have both. I think, 'cause the thing with (inaudible), thinking of mine, like, you need to see it coming, not just believing that the call handler is telling you it's coming.

I: Yes. Yeah. Okay. Do you think it would make it easier or more difficult for you to use a defibrillator if one was delivered by a drone?

R: To be honest, I don't, I don't know. I really don't think transportation affects the ability for you to learn how to deliver the device. 'Cause whether it's delivered by drone, or whether it's already there, you're still gonna have to figure out how to use it. But I do think, like, in the time that maybe the drone gets to you, the call handler could already, like, give you, like, instructions. So, it's not, like, as the device gets there you start having to learn. Like, before the device even gets there, you could have already started (loss of audio) to use it.

I: Could you just say that last bit again? 'Cause of, the connection dropped out, sorry.

R: I said that it would, it would be nice that, like, as the device is coming to you, you're already being informed on how to use it.

I: Okay. And how would you feel about leaving the person if they're by themselves to get the defibrillator from the drone?

R: Well, to be honest, that, that does make me a bit uncomfortable. I, I would prefer if there's someone else that can get the defibrillator, instead of me having to leave my uncle. I, I don't think I would have been able to do that. I think that part's a bit tricky.

00:49:39

I: And why would you feel uncomfortable then?

R: (Pause) I feel like leaving them could potentially impact with their care. Even though I'm bringing a defibrillator, I think I'd still just be too worried of leaving, about leaving their side.

I: How far, if at all, would you be willing to go to get the defibrillator that had been delivered?

R: (Pause) I feel like it shouldn't take me more, like, going and coming, it shouldn't take me up to two minutes. Like if I'm brisk walking, shouldn't take me up to two minutes.

I: You wouldn't wanna go beyond that time frame.

R: Yeah.

I: Would location affect your thinking on this? So, imagine a cardiac arrest happened at home, as opposed to in a public space. Would that affect your thinking on how far you'd be willing to go to get the defibrillator, or would the same, kind of, rule for you apply?

R: I think the same would apply.

I: Yeah. Yeah. So, the, the, the quicker you can get it, the better, in either situation.

R: Yeah.

I: And two minutes ...

R: Yeah.

I: ... is your maximum. Okay. And what do you think the role of the 999 call handler is in all this? I know we touched upon that just before. Is there anything else you would want from them, or ...? What's their role, do you think, in this?

R: They, to a very large extent, they perform their responsibility well (?) to guide you is providing emergency help until professionals show up.

I: Yeah. And, I know you said the public might be sceptical about seeing drones delivering … Hello?

R: No, still here, sorry ...

I: Sorry ...

R: ... I, I think it was ...

I: ... your picture went. Sorry. That's fine. So, I know you said the public might have misperceptions about the drone and that might make them less likely to interact with the drone. Can you, do you have any other concerns, or can you foresee any other issues with using a drone delivered defibrillator?

R: Well, electrical devices, they all have bad days. What happens, like, if the drone malfunctions along the way? Like, what, what protocols are in place to, you know, help with that? I think that's the greatest issue I have ...

I: Yeah.

R: ... (inaudible)

I: Yeah. Okay. Excellent. Thank you. So, I have some final brief questions that may be useful for our research. So, I think you mentioned it right at the start, but just to clarify. If I may ask, please, what is your age and gender?

00:52:43

R: I'm female, and I'm 25.

I: Thank you. And have you had training in CPR and defibrillator use?

R: No, only on CPR. Not with defibrillator use.

I: Okay. And when was this?

R: CPR was about 10 years ago. It's, it's been a minute.

I: It's been minutes, did you say? Sorry.

R: I said that it, it was about 10 years ago.

I: 10 years ago. Yeah. And it was only CPR.

R: Yes.

I: Yeah. And that was at the school, wasn't it? I think you said earlier.

R:  (inaudible)

I: And I presume it, the CPR training happened before the incident that we discussed today?

R: Oh, yes. (Chuckles)

I: Okay. Excellent. Thank you for that. So, before we finish is there something else you would like to add to your answers today?

R: Nothing off the top of my head.

I: Okay. Thank you. So, I'm just going to turn off the audio recorders, and just go through briefly what will happen next.

**END OF INTERVIEW**

Participant 5

I:  Excellent. Okay. So the main interview audio recording has begun, and it will continue until the interview questions end. So to start, then, could you tell me as much as you can about when you provided assistance to the person who had a cardiac arrest, please?

R: It was [time] in the morning on the [date of incident] (clears throat) [year]. It was in the house. It was my partner. He'd just put a teabag in a cup for me and put the kettle on to boil, he went in the room to sit down, and before I’d finished making (inaudible) and making my cup of tea, I could hear him making, like, big, massive breaths. And so I went in to see what was wrong, and you could tell by looking at him that he wasn’t in … his eyes were not, you know, focused or they were, like, slightly rolled up. And so, I give him a little tap on the face and, you know, asked him if he was alright and tried to (inaudible) so I dialled 999, 'cause I thought he was having a stroke, 'cause his brother (?) had had one. And while I was on the phone, he just, his lips went blue and you could tell he was basically dead. I then ran out the street to shout for somebody to help me get him on the floor because I couldn't do it myself 'cause he's too heavy. And then my neighbour come in and helped me get him on the floor. And I did CPR until the [first responder] … do you know what [that] is?

I: No, sorry, could you explain, please?

00:02:21

R: They're, like, the emergency people who only come out to things like cardiac arrests …

I: Yeah.

R: … you know, in a car. She came out on her own. She was here first. And she was here, I think it was nine or ten minutes and she was here. And then I stopped and handed him over to her. Never been so glad to see someone.

I: Yes. Sure. Yeah. Are you okay to continue with this? I …

R: Yeah. Yeah, I'm fine.

I: … I understand it's quite difficult. And, as I say, you can pause or, or reschedule, terminate the interview. It's up to you if you're okay to continue.

R: Yeah, I'm fine.

I: Okay. Yeah, I'm sorry to hear that. It sounds really difficult. So if I may ask, you, you say that you thought that your partner, your husband was suffering from a stroke.

R: It wasn’t a stroke though. It was, it was, he'd just, it was a cardiac arrest, not a stroke.

I: Yes. Yeah. So how did you realise it was a cardiac arrest?

R: Well, 'cause he wasn’t breathing at all. You know, his lips went blue. I'm just listening then to what the, the call handler said to me. She just said, "You need to do CPR."

I: Yeah. Yeah. So, so how soon afterwards, him becoming ill, did you realise it was a cardiac arrest?

R: Well, I, I knew his heart had stopped (inaudible). I dunno if I actually knew the difference between a cardiac arrest and a heart attack, but I knew that I had to do CPR to keep the oxygen to his brain (inaudible).

I: Okay. And if I may, what was the outcome?

R: He's really good. He (loss of audio) memory issues and he's, you know, he's still just tired and, you know (inaudible) brilliant.

I: So, so they, they helped, the emergency services?

R: Yeah, he was, he, when they came, they had to work on him for a long, long time. They couldn't get (inaudible) his heart. They, they (inaudible) altogether. And when he came round, he was so, he wasn’t awake, but his arms and legs were feeling and they (inaudible) by the time he was lying on his legs. And they said they had to come in a helicopter, get the helicopter, with the speciality (?) put him, put him in the house 'cause they said he wouldn't have made it otherwise.

I: Okay. Okay. So you said that you performed CPR.

R: Yeah.

I: And I will ask you about your experiences of that in a moment. Did you have immediate access to a defibrillator?

R: No.

00:05:48

I: No. Okay. And …

R: There is one (inaudible) in the square in the town, but I never had time to go down and get it.

I: Okay.

R: So …

I: Okay. And we'll go through that in a little bit more detail as well in a moment. So initially you were by yourself in your house.

R: Yeah.

I: And then you went out in the street, and then did other people come to you at that point?

R: The neighbour came in, helped me get him on the floor.

I: Yes.

R: And she, I know (inaudible) used to work in a school, and she worked in the same school. And, and I did say to her at one point, "Oh, can you do this a minute while I put my trousers on?" 'Cause I was (inaudible) and my top. And I just didn’t, I dunno, I just wanted to, I thought as soon as the ambulance came I would be going with him straightaway. I just wanted a couple of seconds to put my trousers on. And then she said she had to go 'cause she was taking another class. So, so she went then.

I: Okay. So how did it make you feel when you were initially on your own and then when your neighbour came to help? How did that all make you feel?

R: Well, I was very glad she came to help get him on the floor for me, 'cause I couldn't do it.

I: Yeah.

R: So I was really grateful to her.

I: Yeah. Sure. And did she do anything else to …

R: No, she had to go then.

I: She had to go.

R: Yeah.

I: Okay. And was there anyone else around?

R: The woman she was with in the street took her dog home, and then she came and let herself in as, before the paramedic lady came. They couldn't (inaudible) the whole thing.

I: Okay.

R: (inaudible 00:07:47) but then came in with me.

I: Okay.

R: But she, she had to take her dog home first.

I: So would you mind telling me as much as you can about the 999 call?

00:08:06

R: What, what do you wanna know about it?

I: So, firstly, who made the call? Was it yourself?

R: Me, yeah.

I: And did you use a landline or a mobile to speak to the call …

R: Mobile.

I: Mobile. And did you put this on speakerphone?

R: Yeah. I phoned 999, put it on speakerphone (inaudible) tried to get him on the floor myself and I couldn't. So I shut my dogs out of the room so I could open the front door and shout to somebody. Yeah, and then got him on the floor, and I just left my phone there on speakerphone on the sofa, and I just didn’t bother with it then. I could hear everything. It was on speakerphone.

I: Okay. Okay. So did you have to leave the patient to make the call …

R: No.

I: … your husband to make the call? No. So, so you left your husband when you went out in the street to get help. And …

R: Yeah.

I: … and how did that make you feel, leaving his side momentarily to, to do that?

R: Didn’t even think about it. I just knew I had to get somebody in quick to help me.

I: Yeah, sure. Understandable. And so I know it was, it must have been very chaotic and, you know, stressful, but I was just wondering though if you could remember, if you can remember what the call handler said or what help did they offer you?

R: She said to do CPR. But (inaudible) I said to her, "Shall I get," I said, "Shall I get a neighbour to help me?" and then just ran out and got a neighbour. And then she started talking me through CPR. But I already knew how to, 'cause I've taken lots of courses. So I was just already, you know. But she was saying to me, "Count out loud so I can hear you and I know you're doing it." And she was saying, like, "One, two, three four, one, two, three, four." I remember that (?). After a while she did say to stop and see if he was breathing or anything.

I: Yes.

R: And he did make a few, a little noise. She said, "Don’t confuse that (?). Just," you know, " carry on with the CPR." And my, it's all been recorded. You could listen to it if you wanted to, it’s been released.

I: Oh, thank you. But I, I wouldn't be allowed. That’s not part of our study approvals. But I appreciate you trying to help us and offering, offering that. But we're, you know, we'll listen to your experiences and, and how you found the call and, and what, you know, just wondering what the call handler said from your perspective, if you can, you know, what you can recall of it.

R: Yeah.

I: Yeah. Thank you though. So what questions did the call handler ask you?

R: "Is he breathing?"

I: Yeah. Yeah.

R: They'd said, and when she said that is when I could tell he wasn’t struggling anymore. His eyes had closed up, lips blue. And I, I thought, "He's gone." That’s when she said, "Well, you're gonna need to do CPR."

I: Yeah. Okay. And did you ask the call handler any questions?

00:11:41

R: No. Nothing (?), not at all.

I: No.

R: I just went into emergency mode.

I: Yes. Yeah. Of course. Yeah.  And what are your reflections on the interaction? So what went well, what was difficult, if anything?

R: I think everything on their behalf was brilliant. You know, the call handler, if I didn’t know how to do it, they'd talk me through it. I wasn’t really listening fully. But yeah, the call handler was great. The paramedics were great when they came. The ambulance crew, the helicopter crew, everyone, they were all wonderful.

I: Yeah.

R: The actual CPR itself was horrible. I broke his sternum. And I could feel it. And I knew, even though it was broken, I'll have to keep on straining/strangling (?) hard (?) but then his eyes were open, which was awful. But, you, you know, you've just got to do it, haven't I?

I: It was what, sorry?

R: (inaudible) get on now either that or he’s gonna die, so …

I: Yeah. Yeah. So I was going to ask you about your experiences of performing CPR. So did you do chest compressions only?

R: Yeah.

I: Yeah.

R: She, I said at one point, "I haven't done any breaths," and she said, "Don't worry about the breaths for now. Just keep doing the compressions."

I: Okay. And you say that it, it wasn't a very nice experience for you …

R: No.

I: … because … no. No.

R: I, I (inaudible). Absolutely horrible.

I: Yes. Yeah. Okay. So the, the call handler then, she, she gave you instructions in terms of telling you how many compressions to do and to count them …

R: She just said …

I: … out and that.

R: … "Just keeping doing them. One, two, three, four, one, two, three, four, one, two, three, four." Yeah. And she said to count out loud so she knew I was doing it and how fast I was doing it.

I: And did she offer you or give you any other instructions?

R: I can't remember.

I: No, that, that’s, that’s understandable. No, thank you for, for sharing that. And what are your reflections on the instructions and the advice that you were given from the call handler?

00:14:10

R: Yeah, no, good.

I: Yeah.

R: They, they are trained, isn't they? They just go through a script I think, don’t they?

I: Sorry, could you repeat that?

R: I think they just go through a script, don’t they, 'cause I’ve heard other calls are very similar.

I: Yes, they, you think that they use a script when they're giving the instructions. Okay. So you said earlier that a defibrillator was not used. So what …

R: There was one (inaudible).

I: There, there was one nearby, did you say?

R: Yeah. I didn’t use one. The [first responder] (inaudible) machine as well to do all the CPR 'cause it went on for so long.

I: Okay. So is that why … I was going to ask you what your thoughts on, what are your thoughts on why a defibrillator was not used?

R: (inaudible)

I: Sorry, I didn’t catch that.

R: (inaudible)

I: I'm not sure if it's where you are, but it's, like, breaking up, the, the sound.

R: Ah, sorry.

I: It's okay.

R: I said once the paramedics arrived …

I: Yes.

R: … the defibrillator was used. It was used six times.

I: Okay. I heard that clearly. Thank you. So I was wondering, like, what … I understand that the paramedic shocked your husband. I was just wondering why the, you or your neighbour or, why, why the defibrillator was not used in that, before.

R: Because it would have taken us a good, it would, probably would have taken ten minutes for somebody to go and get it. There's not one, you know, closer than that.

I: Okay. So it would …

R: And I was on my own most of the time anyway.

I: Yes. Yeah. So it would have been difficult for you to have gone. Where was it? How far away was the defibrillator from where you were?

R: It's not very far, but it's about a three- or four-minute walk.

I: Yes.

R: Do you want me to put you on speakerphone and see if that’s better now?

I: Yeah. Thank you.

R: Can you hear me better now?

I: Yes, I can hear you. We'll, I'll see how it, how it goes. These things, they can be a little bit capricious, can't they? Up and down, the connection. Can you hear me okay?

00:16:58

R: Yeah, I can hear you fine.

I: Okay. Had the call handler advised you that a defibrillator was nearby?

R: No, she asked me if I have access to …

I: Yeah. And you said, "No." Is that correct?

R: Yeah.

I: Yeah. Okay. And just to clarify, no one brought you a defibrillator.

R: No.

I: No. And you didn’t retrieve one. No.

R: No.

I: No. Okay. Thank you. So what are your thoughts about using a defibrillator in the future?

R: Would I use one, you mean?

I: Sorry?

R: Do you mean would I use one?

I: Would you use one? Or … yeah. And …

R: Yeah.

I: … like, why, if you would? Okay. So, so you would. So, so why?

R: Yeah.

I: Why is that then?

R: Well, to try and get them back functioning quicker. To shock the heart back into a, you know, a viable rhythm.

I: Okay. And is there something that would make it easier, then, for you to use a defibrillator in the future?

R: Well, it would probably be a lot easier if someone else was there with you.

I: Okay. Why is that, do you think?

R: Well, it's always nice to have somebody else there. And I would have to stop doing compressions (inaudible). But at that point in time, I hadn't done anything. And anything I could, I would have just done it.

I: Sorry, it broke up again and I couldn't catch, couldn't catch what you said, the last bit. I'm sorry.

R: I said whatever I was told to do, I would have done, I would have done *anything*.

I: Yes. Yes. Okay. But because the call handler didn’t tell you to use one …

R: But I couldn't have one to use.

I: Yeah, didn’t, yeah, didn’t have one to use. Yeah. Okay. And, sorry, just to clarify, the, the ambulance, did they come into your house?

R: Yeah.

00:19:35

I: Yes. Yeah. So did you have to leave the, your husband to open the door to them?

R: No.

I: They just walked in?

R: Yeah. The neighbour that had arrived by then saw them coming, and she opened the door.

I: Okay. I just wanted to see whether you'd left him to, to let them in, but your neighbour did that. That’s excellent. Thank you. So if it's okay with you, could we now discuss drones delivering defibrillators and your attitudes around that? So, firstly, is this technology, drones delivering defibrillators at the scene of a cardiac arrest, something that you've heard of?

R: No, I've never heard of it.

I: No. And have you heard of drones?

R: Yeah.

I: Okay. Have you seen drones used or used one yourself?

R: Yeah.

I: Okay. In what situation?

R: My grandson used to have one. And I've just seen them on lots of things. People (inaudible) a drone.

I: What are your thoughts about them then?

R: Well, I think there will be a lot more in the future. 'Cause, as I say, lots of (inaudible) and they have them in the rugby and everything now, don’t they? [Name of City] and …

I: Yes. Yeah, they're, the technology is becoming more prolific.

R: And the police use them. I've seen them on the police programmes. They got a drone up.

I: Okay. So, so you are aware that they exist and they can do different things, depending on the situation. So how do you imagine drove-delivered defibrillation works?

R: Well, if, if they can do it, I think it would be really good.

I: Yeah. And why do you think that?

R: Because if you're on your own, you wouldn't have to leave the person to run and get one. Or you wouldn't have to wait for the ambulance crew to arrive.

I: Yeah. Yeah. So you, you, you see some real benefits, real advantages …

R: Yeah.

I: … to drones delivering defibrillators. Yeah. Oh, that’s really interesting. Thank you. So as I'm sure you're aware, drones are small, flying aircraft that are piloted remotely. And as you were saying, they have camera capabilities, that they can navigate and see where to go. Now, they can carry defibrillators by landing and detaching the device, or they can hover above the ground and winch the device down to the ground. So can you imagine interacting with a defibrillator in the ways that I've just described?

00:22:46

R: Yeah.

I: Which way, if any, seems more advantageous to you? Or can you comment on those different methods of delivering the defibrillator to the person?

R: I don't think it would make any difference. 'Cause if (inaudible) in that situation, I think you'd just either, either (inaudible) produce (?) nothing or you would do anything and you'd just go up and get it.

I: You …

R: (inaudible) do anything.

I: Yes, you'd get it from, from the drone. Is that what you mean?

R: Yeah.

I: Okay. And what about getting, like, getting the defibrillator, making sure it gets safely to the ground? Do you think there's a particular way? Do you think detaching the device from the defibrillator is a safer way, or if you winch it down, or you, kind of, can't say?

R: No, I don't know.

I: No. Okay. And how would you feel about a drone bringing a defibrillator to you whilst helping someone having a cardiac arrest?

R: I would be grateful for any help.

I: And do you think it would make it easier or more difficult for you to use a defibrillator?

R: It'd be the same as using any defibrillator, wouldn't it?

I: Why do you think that?

R: There’re all similar.

I: Sorry?

R: They're all the same, ain’t they? Oh, no, the paramedics got the ones that zap you. The other ones are sticky pads, ain’t they, that go on your back and your chest.

I: Okay. And I was wondering as well, like, how you would feel about leaving the person to get the defibrillator from the drone if they are by themselves.

R: You would be in a rush to do it, but if that’s what you needed to do, that’s what you have to do.

I: How far would you be willing to go to get the defibrillator that had just been delivered?

R: Not far.

I: Not, no. No. Why is that then?

R: Because every minute you leave them without CPR or oxygen around the body, the more chance there is of damage or death.

I: Yes. So you'd want the defibrillator to be delivered quite close.

R: Yes.

00:25:33

I: Yeah. That’s understandable. So …

R: But that's only if you're on your own. If you (inaudible) you go and get it, one of you stay there.

I: Yes. So how, so it, how would you manage to get the defibrillator from the drone if others were around?

R: If others were around?

I: Yes.

R: I'd send somebody else.

I: So you'd stay with the patient, and the other, the second person you'd send out to get the defibrillator from the drone.

R: Yeah.

I: And if the cardiac arrest occurs at home, would you leave the patient to get the defibrillator from the front door? Or would you be prepared to cross the road to get it? How close would you want it to be delivered if it happened at home?

R: I'd be prepared to cross the road. But I wouldn't wanna have to go far.

I: Yes. And would location affect your thinking on this? So you've said what you'd do if it happened at home, but what about in a public space? Would it affect your thinking on what you've just told me about proximity?

R: (Breathes out and pause) Yeah, I'd still want it to be, you know, you could get it and back in a minute or something (?).

I: Yeah, sure. And what would you want to know from the emergency call handler about drone-delivered defibrillators? So, like, would you want them to tell you that one was coming or when it was there?

R: Yeah.

I: So, yeah, what would you want to know from them?

R: Yeah, I'd wanna know there was one coming.

I: Okay. And would you want to have information on how to identify the drone?

R: There wouldn't be other drones about, would there? I'd just be looking for anything.

I: Yes. Yeah. So you'd want updates that …

R: Yeah.

I: … yeah, that one was coming.

R: Well, the drones would have cameras anyway, wouldn't they? So whoever's flying it would be able to see if they were close to you.

I: Yes. Yeah. So, so what do you think the role of the call handler is in all this, then? If you're saying the drone has a camera and can see, what, what do you think the role of the call handler is?

R: I dunno. To tell, they can tell you when it's coming.

I: Yes. Yeah, you'd still want that information.

00:28:16

R: Yeah, there'd be, kind of, communication, wouldn't there, between the drone people and you.

I: Okay. And what concerns, if any, do you have about drone-delivered defibrillators?

R: Well, I do think perhaps a lot of people wouldn't use them. So it might be a lot of money or resources or … but even if only one person in ten uses them it’s (inaudible).

I: And what problems or issues, if any, do you foresee?

R: Dunno.

I: Yeah, that’s fine. This is new technology, you know, drone-delivered defibrillators. So …

R: As long as the ambulance service are still on their way and are getting (inaudible).

I: Yes.

R: You know, they shouldn't be sent instead.

I: So you wouldn't just want the drone to deliver the defibrillator and then the emergency services don’t turn up.

R: Yeah.

I: Yeah. Yeah, you want the emergency services to, to turn up too. Would that be a concern, then, that you might have, that they wouldn't turn up? Is that what you mean, that …

R: No, but I'm just saying that it, it wouldn't be a substitute, would it? It wouldn't make them not get there any quicker. They still need to get there as quick as they can.

I: Yeah. Yeah, absolutely. Okay. Well, if that’s everything, I'll just move on to the last part of the interview. So thank you for your responses. And I just have some final, brief questions that may be useful for our research. So if I may ask, what is your age and gender?

R: I'm 56 and female.

I: Okay. Thank you. And, and I, I think you mentioned this previously, have you had training in CPR and/or defibrillator use?

R: Yes.

I: Okay. So when was this?

R: I've worked [describes work] for 14 years. And you have one at least every two years.

I: Okay. So you went on a course that was 14 years ago and one that was a couple of years ago, is that correct?

R: I wasn’t, I did my last one last year.

I: Last year. Okay.

R: Yeah.

I: So quite recently then.

R: Yeah.

I: And were the courses CPR only or CPR and defibrillation?

R: (inaudible) 14 years ago I think it was just CPR. But I think for the last, about six years, it has included defib and the practice ones where you get, you know, use the pads and everything.

I: Yes. Yeah. So it's gone through how to use an AED.

00:31:43

R: Yeah, it just talks, yeah, it tells you what to do, doesn’t it?

I: Are these online course, do you mean?

R: No.

I: No. No, in-person courses.

R: Yeah. But the defibrillator tells you where to, you know, what to do. It tells you whether to carry on or not, so …

I: Yeah. Yes. And so I suppose the first round of training you went on 14 years ago, that happened before the incident that we've discussed. What about the more recent training, did that happen before or after the incident being discussed?

R: Before. I haven't been to any courses since.

I: Okay. Okay. Thank you. So is there something else you would like to add to your answers today?

R: No.

I: Okay. So I'm just going to turn off the audio recorders and go through what will happen next.

**END OF INTERVIEW**

Participant 6

I:  Okay. So, the main interview audio recording has begun and it will continue until the interview questions end.

R: Great.

I: So, to start, can you tell me as much as you can about when you provided assistance in a lay capacity to the person who had a cardiac arrest?

R: I've had two relatively recent ones. One, the last one was about five years ago during [outdoor event] when I saw a group of people attending to a runner who'd just collapsed, probably about a minute, 30 seconds to a minute beforehand. And one of them flagged me down 'cause he knew that I, I'd been a doctor and asked me if I would assist, although obviously I was retired, I think, at that stage, or nearly retired, so I was in a, sort of, lay capacity. And there was already one doctor there, who I vaguely knew, who'd, sort of, taken control and was telling everyone what to do. So, we had started CPR and continued CPR for quite some time. The striking thing, I suppose, when I arrived was that I thought that it was potentially a bit of a futile situation 'cause we were [remote location]. But I, sort of, asked, I thought the only thing that would make a difference to him would be a defibrillator really. And I checked that they'd phoned [local care team] and then I said, "Have you asked anyone if, in the [location] if they can bring up [the location’s] defibrillator?" And in fact they phoned down with a mobile and someone, while we continued with CPR, drove [to the location] as far as they could get in the car, and then ran all the way up to the casualty with a defibrillator. So, we did actually manage to use it unsuccessfully. And then eventually the [local care team] arrived and concluded that the situation was, was futile and abandoned resuscitation attempts at that stage.

00:02:34

I: (Quiet) Right, okay. So, the person passed away?

R: He did.

I: Yeah. Okay. And this person was a stranger to you?

R: Yes.

I: Yeah.

R: Didn't know him at all.

I: No, okay. So, you were walking [at location] and you came across him, is that correct?

R: Yeah. Well, it was, it was actually a, a race and I was one of the competitors, as was he, and as were nearly all the helpers, I think, that had amassed at that stage. Probably four or five people had stopped. One was another GP and I think one was definitely a nurse of some sort, and myself.

I: Sure. Yeah. So, did you realise what was happening at first?

R: Eh?

I: Did you ...?

R: Well, yes, in as much as, I suppose, the balance of probabilities was if he collapsed where he'd collapsed he basically just collapsed. And I asked a few more questions as to what had happened and he'd basically been running along and just suddenly collapsed, gone face down, out, out unconscious, lost consciousness suddenly, falling pretty much on his face and just really gone out. At which point the runners around him had stopped and he'd fortunately been near another runner who was a, was a medic who'd started the CPR and was controlling it really, telling everyone what their roles were and what to do.

I: And, and how did you realise it was a cardiac arrest?

R: I, I suppose, strictly speaking, I suppose I didn't, but it was a balance of probability. So, he had no cardiac output, we'd started CPR, he wasn't breathing, he had no consciousness. So, I, I suppose that's an assumption, but it was an assumption that we made.

I: And how soon afterwards did you make this assumption about it being a cardiac arrest?

R: I suppose, I made it within, within seconds, but that was probably because they were already performing CPR. And I knew, knew that there was a medic at the, at the head end. So, I suppose, I, I made, just made an assumption there.

00:05:03

I: And you say the medic performed CPR, did you also perform CPR?

R: I, I did, the, the other medic wasn't actually doing the CPR, one of the nurses was doing CPR, but when she was, became fatigued, 'cause we were there quite a long time, I, I also took over and did CPR.

I: Okay. Okay. So, there were other people there with you. There was this medic, the, the doctor, the nurse. Were there any other people? I guess the runners.

R: At least three or four, I couldn't remember exactly.

I: Three or four. And how did that make you feel that there were these other people around?

R: Fairly comfortable really, particularly as the, the medic in question was doing a, a very organised job, was timing and, and keeping everyone, you know, organised and stopping and starting as people became fatigued. So, so, I, I didn't, I didn't have the normal panic of responsibility that you, you, you have if you're on your own, or you're the more responsible person, or you assume you're the more responsible person.

I: So, just to clarify then, the, the doctor and the nurse, they were taking it in terms to perform CPR as were you?

R: Just the nurse.

I: Oh, just the ...

R: The, the, the other doctor didn't do any, any CPR. He, he, he was just, he was keeping us on timing and, and ...

I: Okay.

R: ... counting out timings for, for, for the CPR, but he wasn't actually doing any CPR.

I: Okay. And what did the other people do? 'Cause you said there was about four, three or four. So ...

R: Mostly standing around, checking on where the person was with the defibrillator that was coming up, guiding other runners away from the casualty so that there wasn't a crowd of people there and just, sort of, standing round doing what they could. One person was happy to do breathing for, for the casualty. So, there was one person giving him, giving him breaths, mouth to mouth.

I: Okay. This wasn't the nurse, this was a member of the team?

R: No. That, that was another member of the, the, the assembled group who had no medical training, as I, as I understand.

I: Yeah. Yeah, okay. Can you tell me as much as you can about the 999 call, please?

R: I think someone with a mobile phoned, basically phoned 999 and asked for [local care team] and explained what had happened. And then that, that's, that, that, that probably occurred just before I arrived. When, when I arrived I checked that they had done that 'cause I have seen circumstances where, where, where no one's, no one's phoned, or no one's started to, started CPR and no one's thought to phone, phone for assistance.

I: So, do you know if this person, well, they presumably used their own mobile phone, do you know or ...?

R: Yes. I'm pretty, pretty sure they used their own mobile phone, I, I wouldn't, I don't know for sure, but, yeah, I can't think how else they would have done it.

00:08:22

I: Do you know if they put it on speakerphone?

R: No, I don't.

I: No. So, you weren't there at all when the call was being made?

R: No. No.

I: So, you don't know what the call handler said or what they said to the …?

R: No, I don't know that.

I: No. Okay, that's fine. Did they relay any, the person who made the call, did they relay any information to you about the call?

R: My recollection is that they said they phoned [local care team] and they had a rough guide for timing of [local care team], I think, which was, you know, within the next 30 minutes or 40 minutes or so.

I: Yeah. Did they seem content with the call or did they raise any issues, do you know?

R: No, they didn't raise any issues at all.

I: No. No. And do you know if they left the patient to make the call?

R: I don't know that.

I: No. No. Okay, that's fine. So, you said that you were involved in performing CPR on the patient.

R: Yeah.

I: So, can you tell me as much as you can about that, please?

R: I was, for, for quite a, quite a while an observer 'cause there were quite a lot of keen characters who were happy to do CPR, but as time passed and they fatigued then I, I took over for a, a period of time. It was probably ten to 15 minutes after, after I arrived.

I: So, did you do chest compressions only?

R: Chest compressions only.

I: Okay. So, not the, you didn't do the rescue breaths?

R: I didn't do rescue breaths. No.

I: That was someone else?

R: That was someone else.

I: Yeah. Yeah. Okay. So, what are your reflections on how that went?

R: I, I felt at the time that he'd had everything that you could possibly do, done in that he'd been spotted straight away, a call had been made for help straight away, CPR had been started straight away and it had been started probably by someone who knew what they were doing, or someone who knew what they were doing was coordinating the, the people who stopped. I felt like a bit of a spare part, I think, for the first ten minutes, 'cause there was plenty people there. I felt the only useful thing I did that probably what could have made a difference was to ask them if they'd thought to get the defibrillator brought up 'cause I really didn't feel that there was anything that was going to help other than the potential of a defibrillator. I was, I, I was a bit pessimistic about the situation anyway, but felt that that, that was the best we could do. And indeed he was probably quite lucky to get a defibrillator within, within 30 minutes. So, I don't think he could have had anything else done to help him.

00:11:30

I: Can you, sorry, were you going to say something else?

R: No, no. That's, that was, kind of, that's, kind of, as much as I thought really.

I: Yeah. So, how did you perform the CPR then, how many, like, do you remember how any chest compressions you gave in a period of time, do you remember ...?

R: No. I, all I, I remember that [Name], who was the other medic, was counting the timing so that the person giving CPR didn't have to really worry about the timing, just had to follow his lead of counting to, to, to give compressions at the appropriate rate. And I probably gave CPR for about five minutes before someone else took over. (inaudible) they took over, he was, he was wearying of counting and I took over the counting and the counting and, and timing the, the person who was giving rescue breaths. I was following his lead really (laughter).

I: It was out of his league, sorry, did you say?

R: So, I said I was following his lead really ...

I: Oh.

R: ... 'cause I think in those circumstances you, you often, no matter how often you've done your training, you, you forget your timings, forget your, the frequency of rescue breaths if you're going to do rescue breaths. Yeah, so, no, I didn't have to do, do much, other than follow what was already being done.

I: So, did you receive or did anybody doing the CPR receive any instructions from the call handler or from the medic there?

R: Not clearly, but I think the people who were giving CPR were, didn't need any further instruction. So, there was no, there was no, no real need to tell the nurse who was giving CPR how to, how to give CPR, or how, how many compressions, or indeed how, how firm the movements, confirmation that it was, that it was appropriate, but nothing else. And the call handler, we never spoke to the call handler again really, that, that I can recall, during the, the episode. There may have been someone telling us where [local care team] were, were up to.

I: Do you know if the lay member of the public who didn't have medical training, if they'd been given any instructions on how to deliver the rescue breaths?

R: I don't know. They appeared to have a fair idea what they were doing and the rescue breaths looked effective in there were, there was chest movement. And I assumed, but, but I didn't check, that, that he had some, sort of, first aid training.

I: And presumably you didn't need instructions because of your medical background, you knew what to do, is that ...?

R: That, that's correct, yes.

I: Yeah.  Okay. So, did you personally use the defibrillator?

00:14:37

R: Well, yes, in as much as we put the pads on and switched it all on. And I think there was no, sort of, my recollection now is that there was no rhythm that could be, that could be shocked.

I: So, you put the pads on and did anybody else interact with the defibrillator?

R: I think the nurse was, was helping as well.

I: Okay. And how easy was it to use and understand?

R: Very straightforward.

I: Why, why, why, why was it straightforward?

R: It, it, it, it told us what to do. (chuckle) It was one of the, the relatively modern ones that gives you instructions. So, if you've, if you've had your training relatively recently and it's given you instruction, you're, you're fairly, fairly adept.

I: And ...

R: (inaudible) cold, wet skin and not very sticky pads are perhaps the only issue. But there didn't seem to be any, any problem with them.

I: You could stick it on his skin fine?

R: We did, yeah.

I: Yeah. So, it was easy to follow the defibrillator voice instructions?

R: Yes. It was very straightforward, more, more than you might, more than I had perhaps imagined having had plenty of training but relatively few, few, fewer events where I've needed to use a defibrillator myself in, in recent years anyway.

I: So, you felt comfortable using the defibrillator?

R: Yes.

I: Okay. And I, I guess you don't, wouldn't know this, if the call handler offered any instructions over the phone about using the defibrillator?

R: Definitely not. I, I don't think there was any, any call handler in contact with us at that stage.

I: Yeah. So, you were all doing it without, you know, putting the pads on ...

R: Yeah.

I: ... and shocking him ...

R: Yeah.

I: ... without advice from any ... What about from [local care team], did they give you any ...?

R: They weren't, they hadn't arrived at that point. We, we got another runner from the race organisation to run up with the defibrillator (inaudible) and then run up the last couple of miles.

I: Okay. So, the runner got, got the defibrillator?

R: Yeah. One of the, one of the, one of the, one of the organisers or another runner.

I: Okay. And who told them to run down to [local care team] to, to get it?

00:17:15

R: Well, I, I didn't, they didn't, they didn't run down to [local care team]. That was, when I'd first arrived at the casualty, that was the point at which I thought the only thing that was gonna help us get anywhere would be a defibrillator. And that's when I thought there'll be, there's a defibrillator in [location].

I: Oh, yes.

R: So, I phoned, I asked someone else to phone the race organisation and asked them to go to the [location] and get [the location’s] defibrillator. So, we actually had the [location’s] defibrillator from the [location] that, that we brought up ourselves. The race organisation brought it up. So, it wasn't [local care team]’s.

I: No, no. Okay. And [the location] was relatively close to your location at the time?

R: The ... No. [The location] was about two miles away at the [area] where the race had started. And the race happened to start very close to the [location] 'cause the registration was just outside it. I didn't know for sure there was a defibrillator there, but I was fairly certain there, there would be one, when I asked to phone down and see if they could, could get one and bring it up, which they did.

I: Yes. Yeah. So, the person ran all the way to the [location] and then ran all the way back, or ...?

R: No, no.

I: No, no.

R: Someone rang up the ...

I: Rang.

R: ... race organiser, 'cause we had the mobile number for the race organiser and asked the race organiser to get someone to bring us the defibrillator. And one of the race organisers who was on the registration desk went and got the defibrillator, got in a car, drove up a track and then got out when the track ended and ran up [to us].

I: And while all this was going on, you, the nurse and the medic, coordinating your efforts, were performing CPR?

R: That's correct, yes.

I: Yeah. Okay, excellent. You've set the scene really nicely there for me so thank you. So, again I guess you don't know whether the call handler mentioned whether a defibrillator was nearby because you weren't privy to that conversation, were you?

R: I, I wasn't privy to that and I assume not as they hadn't, hadn't thought about it.

I: No, no. Okay. And I know you said, like, you were doing the CPR and involved in that, but how, did you have any feelings or thoughts when the, when the defibrillator was being retrieved, you know, as it was coming to you and you were trying to get it, were you feeling or thinking something in particular before it came to you?

R: I suppose, my only thought at the time was that we were probably engaged in a futile exercise. But then you're always quite hopeful that if you've got a defibrillator you might have a successful outcome, so you're, sort of, crossing your fingers that there might be a, a cardiac rhythm that you, you could use a defibrillator with that, that was, that it might have some effect. So, sort of, pessimistic optimism, (inaudible) that sounds a bit contradictory but that, that was my thoughts.

00:20:41

I: Yeah, yeah. Yeah.

R: (inaudible) hopeful but pessimistic.

I: Yes. Yeah, yeah. Mixed, kind of, feelings while it was being ...

R: Yeah.

I: ... brought to you. Understandable, it was difficult situation.

R: Yeah. Well, most people in my training have said if you come across a casualty [in a remote location such as in this case], don't even start CPR because it's going to be a fruitless exercise. But it was a slightly different circumstance, I suppose, in that we weren't completely remote, we were only two miles from civilisation.

I: And may I ask why that advice is given, that you shouldn't provide CPR if [in such a remote location]?

R: It's not, it's not advice as such, it's a commentary that you, it's, it's not gonna be successful. You're, you're gonna have very little chance in the middle of a mountain on your own, which is not where we were at the time, but in, in most circumstances of, of mountain people and runners, if you come across a casualty, you're on your own, there's no way you're gonna get a defibrillator or anyone to you within about 45 minutes, in which case you're, you're going to be very lucky if you can do anything for someone who's arrested.

I: Yeah, yeah. Sure. Okay. And what are your thoughts about using a defibrillator in the future?

R: I think positive largely. I think, I think CPR is relatively rarely effective, effective outside of a hospital environment. But if it's going to be effective then early defibrillation is probably the best chance of, of success so ... And based on that experience, they, they seem to be pretty simple to use in the, in the heat of the moment. It's, it's all very well doing your training and practising and being shown how to use them, but when, when the actual reality hits, it's still slightly, makes you slightly anxious, but it was quite straightforward. I have no, no qualms about using one in the future.

I: No, no. And just something you said about CPR not being particularly effective outside of a hospital setting, do you mean any, well, if a cardiac arrest happens in any location, so at a person's home, or outside, or just if it happens [in remote location], or, or is it ...?

R: (inaudible) very few successful resuscitations, I've often felt that the public feel that CPR is far more effective than it actually is. Perhaps that's a slightly negative view, but, but, yeah, effective CPR and 'til, 'til defibrillation can, can be effective, but you've got to have all, everything lined up perfectly and, and an incident that was, is amenable to that being effective.

I: And you're speaking generally here, you mean?

R: Yeah.

I: Yeah.

R: With, with that said, I've, I've seen it be, be, be effective, surprisingly.

I: Yes, yeah. Yeah. No, I, I just wondered if you meant in a particular location CPR is less effective or whether you just mean generally in any location that a cardiac arrest occurs CPR won't be that effective unless all the other things are lined up. And ...

00:24:26

R: Yeah. I think everything else has to be lined up is what I mean.

I: Yeah, yeah. Sure.

R: Yeah.

I: Okay. And, well, you said that you found using the defibrillator quite straightforward, but is there something that would make it even more straightforward for you to use a defibrillator in the future?

R: I can't think of anything ... No, I don't think there was anything that could make it easier. I think the, the thing that makes it easier is having seen one and practised with it, which fortunately, you know, in, in my position I, I have done. But for someone who's never, never used one, I suspect it might be quite intimidating and, and I'm not sure how, how easy it would be to use if, if you haven't some idea what you're about to do. So, I'm not really the person to ask that because I, I have practised with them. If I've never seen one before, I'm not sure that I'd, how well I'd be able to follow the instructions. I would think quite, quite well given it was pretty obvious how to use it, it told me. But I had the advantage of having used, used one before and practised with them.

I: Yes. So, you were ...

R: (inaudible)

I: ... familiar with the, with the instructions?

R: Yes. I, I think that, that must make a huge difference, 'cause even someone who's familiar with the instructions is feeling some trepidation as they haven’t used one in anger (?)

I: Yeah, sure. Okay, thank you for that. So, if it's okay with you, I'd like to move on to the remaining part of the interview that's looking at your attitudes around drones delivering defibrillators to the scene of a cardiac arrest. So, firstly is this something that you've heard of?

R: I have, yes.

I: Okay. And have you heard of drones?

R: Yes.

I: Have you seen drones used or used one yourself?

R: I haven't used one myself, I have seen drones being used, mostly for filming purposes though.

I: That you've been involved in or watching ...

R: No, just watching.

I: ... yeah, TV and movies and ...

R: Yes.

I: Yeah.

R: Yeah.

I: And how have you come across drone delivery of defibrillators? You said you've heard about this, so where have you heard about it from?

R: I've heard [local care team] talking about using them.

I: Interesting. Okay. So, how do you imagine drone delivered defibrillation works?

00:27:10

R: I would imagine that someone has to pilot the drone and A) know where it's going and B) be able to see where it's going and be somewhere where they can operate it. And I cannot possibly imagine how you can (chuckles) land the drone with the defibrillator. That's some thing of a puzzle to me 'cause I haven't thought about it before.

I: Have [local care team] spoken about, about how it might work or you're not sure?

R: For the most part they've been ... Not really, no. No. They, they, they've been quite keen to show their jetpacks which means (inaudible) of the drone but whether that's a, a reality that's gonna happen I don't know.

I: Okay. A, a jetpack, you say?

R: Yeah. Yeah.

I: Yeah.

R: The [local care team] has experimented with a, like, a, a personal jetpack so, so they can fly a [local care team] individual [to a remote location].

I: Ah, oh, interesting, okay.

R: And a, and a couple of reports of them experimenting and trialling that, sort of, technology. It's in it’s infancy, I'm sure.

I: Yeah, this technology, Drone delivered defibrillators in particular is, is new and, and that's why we're wondering what ...

R: Yeah.

I: ... you know, bystanders think about it. So, yes, as you've insinuated there, drones can carry defibrillators by landing and detaching the defibrillator, but they can also, the drone can hover above the ground and then winch the defibrillator down to the ground if you, kind of, imagine the winch as a rope like device.

R: Yeah. Yes.

I: So, wondering if you could imagine interacting with a defibrillator in the ways that I've just described?

R: I, I don't see why not.

I: Yeah. What do you think about the winching it down to the ground?

R: It sounds, well, yeah, very interesting. The, the only thing I think about when, when you're telling me about that is most, most of the time we imagine drones to be quite small machines, but a drone that's big enough to carry a defibrillator is probably a fairly big machine with hazardous whirling propellers. And I'm thinking I'm not sure it will just sit underneath the drone. But that's just off the top of my head thought.

I: Yes. No that's fine. That's, that's really interesting. You're perhaps slightly concerned whether the drone would be large enough to accommodate a defibrillator, so making sure that it does would be important for you?

R: Well, well, yes. I mean, they, they, we think of drones often as small machines 'cause we see people flying little camera drones around, but drones can be quite enormous mini helicopters, can't they?

I: Yeah, okay. And how would you feel about a drone bringing a defibrillator to you whilst helping someone having a cardiac arrest?

00:30:31

R: It would, I mean, it would be a good way of getting a, a defibrillator there as quickly as possible and that's a positive really 'cause in a, in a, in a cardiac arrest I do feel that CPR is just buying time until you can see whether you can use a defibrillator to get a, an effective heart rhythm. And, and that's, that's perhaps the, the biggest factor in a successful outcome.

I: Yeah. Quick response rate and getting the defibrillator quickly put on the person.

R: Yeah. Yeah. I mean the, the, the incident I was involved in, you, you wonder if you had a defibrillator immediately whether you might have had a, a much, much higher chance of success than, than after 30 minutes when the chances of success were, are slim.

I: Is that how long you had to wait for a defibrillator or is that how long it took from the casualty falling, collapsing to being shocked, 30 minutes?

R: I would think it was at least 30 minutes.

I: Yeah. Okay. So, do you think it would make it easier or more difficult for you to use a defibrillator?

R: No difference really. The, the defibrillator arrives, it doesn't really make any difference how it arrives.

I: Yeah. Yeah. And how would you feel about leaving the person if you were by yourself with them?

R: Well, it's, it's, it's the balance of odds really. If you don't leave them and get the defibrillator, they're probably not going to survive because all you're doing is, in terms of CPR, is, is keeping is keeping things going until you can attempt defibrillation. So, as long as you didn't have to leave for particularly long I guess, I guess you'd, you'd have to leave if you were on your own. Yeah, you're balancing, balancing odds against time really, aren't you, balancing, balancing risk.

I: Yeah, sure. And how far would you be willing to go then to get the defibrillator that had just been delivered by the drone?

R: Well, well, not very far because if, again if I stop CPR for more than, more than a minute or two the casualty's going to be damaged irretrievably anyway.

I: So, if say a cardiac arrest happened at home, would you be prepared to leave the patient's side to get the defibrillator say from the doorstep or would you be prepared to get it over the road?

R: Oh, that's, that's a quite difficult question because if you are on your own and you stop CPR for a prolonged period of time or a period of time the individual is not going to survive. But if you don't get the defibrillator and there's no other way of getting it to you, the casualty's not gonna survive either. So, I guess you're making a, a judgement in a difficult circumstance then. At my doorstep probably you could get it, yeah. Across the road, probably too far ...

I: Yeah.

R: ... off the top of my head.

I: Yeah. Yes. It's difficult.

R: Yeah, making me think a bit now. (Laughter)

I: (Laughter) Oh, sorry. You don't have to think about it in any depth, you know, if it's, if that's too difficult.

R: (inaudible) (laughs)

00:34:20

I: (laughs) So, if there was other people there, so say there was, you're with another person, how would you get the defibrillator from the drone, how, how would you manage that?

R: Well, you'd send the other person. You'd, well, you need, you need someone, all you need is someone giving effective CPR and someone who can effectively bring you a defibrillator. So, instruct them to go and get it or instruct them, if they're giving effective CPR, and get it yourself.

I: Yeah, yeah. Sure. Yeah, okay. So, what do you think the role of the call handler is in all, in all this?

R: I, I know that call handlers can give very effective advice about resuscitation, CPR and timing and what to do. So if you've got access to a call handler who's got that experience, think that, I think that's very, very, very useful.

I: And what would you want to know from the call handler about drone delivered defibrillation, if anything?

R: How to get the drone, how to get the defibrillator from the drone, where the defibrillator's going to be and whether it's released from the drone or safe to proceed under the drone to, to grab it, how long it's going to take to get it to me and where any other services are that are going to assist me as well at the same time which is not entirely relevant to the drone.

I: Would you want to, like, know how to identify it as well or -?

R: I suppose so, although if this were a helicopter getting a defibrillator outside your door, it'll be obvious. (Laughter)

I: Yeah, yeah, yeah, yeah. Okay. So, what concerns, if any, do you have about this technology? I know you said about the size of the drone needing to be large enough to accommodate the, the defibrillator. Any other concerns or issues?

R: No, not beyond the, the safety of the person giving CPR and where the drone is flying relative to them. And where the drone is after it's dropped the defibrillator off. I haven't got any other particular concerns ...

I: No. No.

R: ... that I can think of.

I: So, you mentioned safety there, so, sorry, could you just elaborate on that?

R: Well, drones are quite big machines. No one wants to get hit by a drone or a descending defibrillator.

I: Yeah. So, it would need to drop or detach the defibrillator in a safe way and land not too close to the person.

R: Yes, that, that seems like a reasonable, a reasonable concern to me.

I: So, do you think it's a balance between the defibrillator being delivered close to the scene, so like you say, you're not leaving the person for too long, as you've mentioned, but then not too close that the drone risks injuring the bystander?

00:37:55

R: Yes. Yes, particularly depending on how many people are at the scene and who can retrieve the defibrillator from, from the drone or from the drop point.

I: So, would you say it's a consideration then?

R: Yes.

I: Yeah. Okay, that's fine. So thank you for those contributions, really interesting insights there. So, I just have some final brief questions that may be useful for our research. So, if I may ask, what is your age and gender?

R: My age is 61 and I'm male.

I: Thank you. So, you, have, you've had training in CPR and defibrillator use?

R: I have, yes.

I: Okay. So, when was this training?

R: I probably haven't had training since I retired and had to do my mandatory training once a year. And I retired about eight years ago now. And prior to that I have done ATLS and other training which also involves defibrillator use.

I: So, you would say over five years ago?

R: Definitely over five years, yes.

I: Yeah. Over, over five years. Okay. So, nearly ten years, would you say?

R: That would be about right, yes. I, as soon as I retired I haven't done any mandatory training since.

I: Yeah, okay. And it was both CPR and defibrillation?

R: Yes, I have done both.

I: And was the training before or after the incident being discussed?

R: I will have done training before and I don't think I'll have done any training since. No, I'm pretty sure I haven't done any since, 'cause I'm pretty sure I was already retired at the time of that incident.

I: Yeah, sure. Okay.

R: In fact, I was definitely retired 'cause there was another incident and I was definitely retired at that one.

I: Okay. Oh, dear. Okay, is there something else you would like to add to your answers today?

R: No. No, that's everything really.

I: Okay. So, I'm just going to stop the recording, the audio recorders, and go through what will happen next.

R: Okay.

**END OF INTERVIEW**

Participant 7

I:  Okay. So, sorry about that, it's a bit fiddly. (chuckles)

R: That's alright. (chuckles)

I: The main interview audio recording has begun, and ...

R: Fabulous.

I: ... it will continue until the interview questions end.

R: Yeah.

I: So, to start then, please could you tell me as much as you can about when you provided assistance, in a lay capacity, to the person who had a cardiac arrest.

R: Okay. So, I'm a volunteer community first responder for the [Name of NHS Ambulance Trust]. So, I've been to approximately 18 cardiac arrests in my time. Did, did you want me to focus on a specific one, or ...?

I: Yes, and as long as ...

00:00:45

R: Okay.

I: ... it was when you participated as a, a lay community ...

R: Yeah. Yeah.

I: ... first responder ...

R: Yeah. Yeah.

I: ... not as a medical ...

R: Okay.

I: ... professional.

R: No. Yeah. Okay. Yeah. So, so, I, I attended a, a cardiac arrest in the area in which I live. I attended, the patient was on the floor in cardiac arrest, having taken overdoses of several types of medication. When I attended, the partner was doing CPR on my arrival. I took over CPR, and basic, kind of, support. And within about 20 minutes, or 25 minutes, there was a backup resource available to attend. And we had several cars, ambulance vehicles, and [another organisation] came. So ...

I: Okay. Thank you. So, just to clarify, where did the cardiac arrest happen?

R: In the, in, in, in the middle of a local authority housing estate.

I: Okay. So, it was in, in their house ...

R: Yeah.

I: ... the person's house.

R: Yeah, yeah.

I: Okay. And what was the, who was the person who had a cardiac arrest? What was their relationship to you?

R: It was a patient. So, it was a, yeah, it was a lady in her early 40s. And her, her partner had found her unresponsive in bed after some time. So, he'd called, he called for an emergency ambulance.

I: Yeah. Sure. And so, when you arrived at the scene, did you realise what was happening at first? Did you know that this was a cardiac arrest?

R: Yeah. Yeah. Yeah. So, so the partner was doing CPR on my arrival, and so I, I, I, I instantly took over the CPR and BLS as, as we do as a volunteer CFR. He was doing a cracking job, bless him, at the CPR. So, he kept going. I applied the defib pads, and the defib wasn't able to give the patient a shock.

I: Okay. And we'll talk about your experiences of that in a moment. Do you know what the outcome was?

R: She, we, we had several ROSCs, and she was conveyed to hospital.

I: Okay. And did you perform CPR?

R: Yeah.

I: Yeah. Okay. And again, we'll talk about that in a moment.

R: Sure.

00:03:23

I: So, how did it make you feel that there were other people there? So, presumably just the partner, that ...?

R: Yeah. Yeah, just the, the partner who, who, who, in fairness, was doing really good CPR at, at the time. Yeah. I, I instructed him to, sort of, keep on going up, while I did, did other bits and bobs. And then I took over the stuff from him, because he, he got very emotional at one point. I think when, when other people arrive on scene, the adrenaline stops. So, bless him. And then, and then, when he stood up, it was apparent the fact that he, he had his own health conditions. So, I instructed him to, kind of, go and, go and take a seat in a different room. So ...

I: Yeah. Yeah. 'Cause it can be quite tiring, can't it performing CPR?

R: Oh yeah, bless him. He'd, he, you know, I mean, I, I think I was on scene within about 12 minutes. But, you know, he was, he'd, he'd, he'd found her unresponsive, and then gone to a neighbour's house to call, to call an ambulance. Because they, they didn't have a phone. And he only had one leg, bless him, so, by the time he got back to his partner, and then started CPR, and then ... And it's pretty decent CPR, to be fair to him. I then turned up, and I think all the adrenaline stopped. So ...

I: How did it feel to have other people there with you? At the scene.

R: I've been to a good few now, so, I mean, in fairness, you know, if, my initial action is, "Oh, great, I've got somebody else to cope with but obviously, if there is more than one person on scene at an arrest, then, then we can all be doing different things. I've been to a different cardiac arrest where a person had, had arrived after my arrival, having gone to fetch a public access defib as well, you know. And I think they found it difficult, because they, they, they were asked to leave the patient to fetch it. Which, which isn't, isn't a [Name of NHS Ambulance Trust] policy. So, yeah.

I: Okay. So, you said this person, he was performing CPR. Was he doing, the partner, was he doing anything else?

R: No.

I: No.

R: No. He was, he was performing CPR, and waiting for us to arrive. The, the problem I, I had on scene, was actually locating the property. It was in the middle of a large, sort of, LA housing estate. All the numbers were jumbled up, because several of them have been knocked down. It was [late] at night. Nobody was standing outside waiting for us, and I literally spent five minutes wandering through the housing estate, you know, sort of, calling out. And then, this voice from above somewhere went, "Love, at the fire escape." So, I turned around, and there was a fire escape. So, I legged it up the fire escape to find the person in cardiac arrest. In not very pleasant living conditions. So ...

I: Yeah, it must have been difficult. So, can you tell me as much as you can about the 999 call?

R: I was responding on behalf of the Trust.

I: Okay.

R: So, so, so, the partner called us. The, the, the partner called us from the neighbour's house, and wasn't with the patient at the time. So, he, so, he wasn't able to kinda answer many of the call handlers questions, because he wasn't with the patient.

00:07:04

I: Okay. So, you didn't call another call handler, you, to, for the ...

R: No.

I: ... ambulance crew, the paramedics to arrive.

R: Yeah. Yeah. So ...

I: Yeah.

R: ... so, it, it came through as a cardiac arrest. So, I respond first, first on scene, as a volunteer CFR. And then, because I'm based in the community, I'm often first on scene, and closest. So, then I, I then get backed up by, by some staff. So ...

I: Okay. So, how did you get him to hospital? Because presumably, I'm just trying to understand how the patient got to hospital.

R: So, so, I'm the volunteer community first responder. I'm first on scene. I then get backed up automatically, by, by, by ambulances, cars, and helicopters.

I: Oh, okay. So, once they know ...

R: Yeah.

I: ... that you're at the scene, they then come automatically. You don't need to call them again.

R: No. Yeah, they come. If I go to patient who, who isn't in cardiac arrest, who, who then arrest if front of me, yes, I would have to, kind of, call, you know, call control. But, but as it came through as a cardiac arrest, everyone is, is dispatched. So, and then, and then, on arrival on scene, the emergency ambulance crew can then upgrade it again, to get helicopters, [Name of organisation], and, and SPs. And, you know, other, kind of, resources. It was in one of those areas where the ambulance crew, sort of, turned up, and gave me a frown, because, because I attended by myself, at [late] at night, in a fairly dodgy housing estate. So, bless them.

I: They're concerned about your ...

R: Yeah, they were.

I: ... welfare.

R: So, yeah.

I: Yeah. Have you ever made a 999 call?

R: Yeah. So, so, I was, I, I was in a governor's meeting in a school, and I had a call from a member, member of our staff, the fact that there was a person who was unconscious on the floor outside the school. I, I, I often have these texts and phone calls. So, I turned up on scene. I looked at the patient. I, I made a triple nine call, explained who I was. The call handlers all have to go through their script, you know, in fairness, I am, I am calling in as, as a member of the public. They have to, kind of, ask me questions. They asked me if he's breathing, I said no. They then started instructing me on how to check for breathing, and how to check, and how to apply a defib. Which, which I'd done already, because I am a CFR. And we then, painfully, had to go through all of the call handler’s script questions. So ...

I: So, how did that interaction go, would you say?

00:09:57

R: Yeah. To be fair, our, our call handlers are great. They have their script. They always explain the fact that the questions they have to ask doesn't delay the arrival of, of an emergency ambulance. Which, which I know other people who have called triple nine always find difficult, because, because there are many, there are many questions to ask which help us before our arrival on scene. And I think it's getting, it's getting, kind of, MOPs to understand the fact that, you know, yes there is an, there is an ambulance coming. Because I think there is some confusion over what the call handler does, and what other parts of the team in control do. So, I think people think it's the call handler who is the one pressing the buttons, and, and sending ambulances, whereas actually, it's the call handler who takes all the information, and it's the allocators, and the dispatchers who give us truck (?). So ...

I: And did you ask any questions to the call handler, in this scenario?

R: No, no. Oh yeah, I, I, I asked them if, if there were any resources there about, because, because I wanted an, an indication of how long I would be with my unconscious guy on, on floor. And obviously, we can't give an ETA, but they advised the fact that there was a car coming down from (inaudible), so ….

I: Yeah. And they asked you questions. As you say, they go through a script.

R: Yeah. Yeah, you know, "Is the patient breathing? Please tell me every time every time he takes a breath?" I was, like, "I promise you, he's breathing, otherwise I wouldn't be having this conversation." But they asked me if there was a defib around, I said, "Well, I've got one in the car. It's currently on, on the patient." The police then turned up, and, and intervened, and tried to take over my patient, and tried to stick him on his side. And, and, and I had to stop speaking to the call handler, and tell the police who I was, and what I was doing. I think the call handler then, I think the call handler then, sort of, got confused as, as to who I was talking to. And the police obviously have a different way of handling patients than the ambulance service does. So, there was some confusion as to what was best for the patient. But, you know, all, all the while, we had the call handler on the phone. So, you know. So, yeah, in fairness, the scene management at a, at an outside, kind of, you know, unconscious (inaudible) cardiac arrest is more difficult, you know. Where, where it's in the patient's house, it's normally slightly more controlled. So, well-meaning passers-by stop and ask questions. So, you know.

I: Yeah. Yeah. Which can add to the confusion, potentially.

R: Oh, it does. Yeah.

I: Yeah.

R: Yeah. And then people always sprint up, and go, "I'm a first aider." I'm like, "Well, well, well I'm ambulance service, and so I win." You know, so ...

I: Yeah. Did you leave the patient to make the call?

R: The, the patient was unconscious at the time, and so I stayed next to them. We always ask the, the, the caller to go back to the patient if they aren't with them, or to stay with them. So, yeah, so, yeah, I was, I was kneeling down next to him at the time, with pads on him. So ...

I: Yeah. Yeah. Okay. So, well, just perhaps going back to the initial story ...

R: Yeah.

I: ... or you can ...

R: Yeah.

00:13:29

I: ... talk about the second experience as well, it's up to you.

R: Yeah.

I: Or both, if you, if you want to, it's up to you. Can you tell me as much as you can about performing CPR?

R: Yeah, so, I'm, I'm, I've performed CPR for a long time. It comes very naturally to me. My, so, with the first story, the, in the house, the gentleman had been performing very, very good CPR for a while. So, in fairness, you know, he, he'd, he'd, he'd done a good job of that. I, I put everything down, and then I took over CPR. You know, I, it comes very naturally to me. If you're on the phone to a call handler, they, they will instruct you in depth and rate, because people often do CPR way too fast. Thinking, thinking it will help the patient, which, which it won't. So, yeah, in fairness, you know, I mean, that was probably my 16^th^ cardiac arrest I've been to, so ...

I: So, you're experienced.

R: You know, I ... Yeah. So, yeah.

I: Did you do chest compressions only? Or ...

R: So, I, so, as a lay person, I, I'm able to do (?) chest compressions. As, as a, as a volunteer CFR, I also have oxygen, and, and, and airway adjuncts, and a bag-valve mask. So, we, so, we were giving breaths and compressions at the time.

I: Yes. Yeah. Okay. And what were your reflections on how that went?

R: Yeah, to be fair, you know, I mean, have ... While I was doing the questions, I was asking the partner the history of what happened, and the, the mechanism of injury. So, I, I was getting a lot of information. I'd been advised the fact the patient had taken two boxes of heart problem medication, which, which didn't give me a lot of confidence as to, as to the possible outcome. But, to be fair, we did what we all did as a team, and she survived into the back of an ambulance. So, yeah, you know, I don't think there was anything else I could have done any differently, or better. I'm, you know, the environment wasn't the best environment. They were sharps on the floor. Patient had, you know, had been living in quite a difficult environment, but if the person on the floor isn't breathing, then, then CPR is what is required. Yeah.

I: Sure. And apologies for coughing.

R: That's alright. Gosh, yeah.

I: Haven't been very well.

R: Oh, bless you.

I: Yeah. So, sorry about that. Did the call handler offer, or ... Oh, but ... Sorry. Not for this, not for the first story, because you didn't ...

R: Yeah.

I: ... call the call handler. But for the second experience, did they offer, or give you instructions on how to perform CPR? Did you need it? How did you feel about having ...

R: They, despite not requiring it myself, they, they did follow the script, yeah. So, so, in fairness, you know, they were clear and concise instructions, as to what to do, how fast to do it. Where to put your hands, you know. Clothes off, you know, pads, defib. (cough) Excuse me. I always find it difficult, because I've got a call handler on the phone. I know what I'm doing, and I've got a defib talking to me. So, and I've got people walking past, you know, you know, taking pictures, and videos, and putting it, and putting it, you know, on Facebook.

00:17:18

I: Oh.

R: Yeah. So ... Oh yeah. Yeah. We, we, we live in, in a day and age where if you end up doing CPR in the middle of the street, somebody will always stick it on Facebook.

I: Oh. So, it just adds to the pressure that you're under.

R: Well, yeah. I mean, I am okay, because I do this for, you know, you know. I've been in this for seven years, but yeah. When I walk past a person who is already doing CPR, and, and people are filming, I tend to lose my patience a little bit.

I: Yeah.

R: So ...

I: Okay. So, you said that you used a defibrillator. Can you tell me as much as you can about this experience?

R: Yeah. So, defibs are really easy to use. Open the box, turn them on. Listen to the instructions. Clear, clear pictures on, on the pads. Plenty of awareness given to our local communities, as to how to use them. So, I've, I've helped install 31 public access defibs in the, in the area I respond and live. We lock all of ours, because it makes people call us first. There was an experience, some years ago, in the area I respond, where a person went to get an unlocked defib, and did CPR and defibrillation for about 45 minutes, and hasn't actually called an ambulance. So, so, I always make sure the fact that the defibs I look after, and guard in, on the circuit, are locked, and registered obviously. So, yeah, I mean, in fairness, we, we carry a defib with, with us. The second gentleman, the, was outside the, the school where, where I'm a governor. We bought a defib for the school many years ago. So, had, had we have required a defib, there was one there. So, yeah, in fairness, defibs are, are easy to use. People always think they are big paddles, like we get on, on American television, which have to be charged up, and, sort of, you know, shaken first. Part of what we do, both, as volunteering staff, is making people aware of the fact that it's just pads. Like, like a seatbelt. The defib gives them very clear instructions. Press this button. I think most people think that, that a defib is going to always give the patient a shock, which it isn't. And it often throws them when it doesn't shock. So, with the first patient, we put the pads on, because the partner was screaming at the time. You know, "Please help, please help, please help." And I, and I put the pads on, and it didn't give the patient a shock, because the patient wasn't in a shockable rhythm at the time. And I think the partner thought the defib was broken. So, I had to promptly explain that, I had to promptly explain that this happens at times. And I kept doing CPR, back, back up, and the cavalry arrived. And then eventually, after some adrenaline, and some Narcan, bless her, we, we got her into a shockable rhythm. I had a, I had a very similar cardiac arrest a few weeks before that, and we gave the patient 17 shocks in the end. And, and after the first two, two hours of treatment, even the family were then standing around, going, "Are you gonna, like, take him somewhere?" So, I think it's, sort of, making the, the public aware of the fact that the trust policies, these days, are we stabilise patient first. You know, I think in, in, in the chaos of an arrest, people think we're gonna turn up, pick the patient up, and run. We, we, we don't do that anymore. We, we treat on scene and we do CPR, and defibs. So, the, the patient in the house, with partner doing CPR, we had our (inaudible) turn up, and, and [Name of organisation]. And we had a LUCAS device on the patient. So, our LUCAS was doing compressions on our behalf. And, and, yeah, we were giving patient, sort of, you know, medication every two minutes. And eventually, the patient was stable enough to, kind of, take a, down the (?) steps, through a very big, waiting, angry crowd, in, in the back of the ambulance. But that was after two helicopters had come and help, helped us out.

00:21:51

I: And what's this LUCAS thing you, you mentioned?

R: LUCAS is, is our automatic chest compression device. So, it, so, it, so, it straps, so, it straps around the patient, and it pumps the chest. So, if we put a LUCAS on, it gives us, it gives us more hands on deck, to, kind of, do everything else. And, in fairness, you know, the LUCAS isn't any more effective than a person who has been well, sort of, experienced, you know, in compressions. However, like, the patient, number three I mentioned, who'd had a stroke, bless him, at home. You know, I mean, we were working on this patient for well over three hours. And while there were nine of us, the LUCAS takes, takes a bit of the pressure off us doing compressions, because everyone gets tired. So, we swap around every couple of minutes anyway. But, I mean, I've done, I've done CPR in the past for 40 minutes before backups arrived. So ...

I: And did the, well, how easy was it to follow, first of all, the defibrillator voice instructions?

R: Yeah. So, you know, everyone's always surprised when we demonstrate a defib. They're really easy, you know. It always tells us to, kind of, call triple nine you know. Call triple nine, you know, attach the pads to patient's bare chest, as per pictures on the pads. Stand clear. I find in the middle of cardiac arrest, stand clear isn't always a strong, a strong enough response. Because, because every time people stand clear, somebody else jumps on top of the patient, you know. And goes, "Why are you *stopping*?" The, the number of times I've had to push people off a patient is, is, is uncomfortable. Because, like, I go in, and we start CPR. And then, all of a sudden, we, we stand clear, because the defib wants to analyse. And bystanders don't, don't understand. Even bystanders who have nothing to, kind of, do with the patient. So, I know I've been in the middle of a large, busy shopping street with a defib, and a passer-by has, has jumped up on the patient's chest while the defibs are analysing. So, I, I then have to push, push him off, and explain the fact that, you know, we can't be touching the patient. So, you know, the, the, the stand clear instruction is the only bit of the, of our defibs I think ought to be strengthened.

I: Yeah. Yeah. And you said that the call handler offered instructions over the phone ...

R: Yeah.

I: ... about using the ...

R: Yeah.

I: ... defib for the second patient.

R: Yeah.

I: How did you find following the defibrillator, and call handler instructions at the same time?

R: Yeah. It's very difficult. So, the, it was on the side of a, a busy street. I had, I had police talking as well, and police radios going. I had the call handler on speaker phone on my phone. I had people stopping to ask if they could help. And I had a defib. In fairness, most of the defibs will increase their volume in a busy environment. But equally, it's very easy to, kind of, miss an instruction from a defib, because I've got a call hander talking to me, I, I've got a defib going, police talking, police radios going. And general (inaudible) around. But I'm, I, I'm experienced at, you know, scene management, and I turn around, like, you know, and, kind of, go, "Can everyone stop a second please." You know. The, the call handler obviously has to follow a script, and isn't necessarily clinical at that stage. So, sometimes the instructions can conflict with the timing of the defib, but it's just a case of telling the call handler the fact that, you know, I'm going to stop talking to them for a second, because I'm going to have to double check everyone, everyone is standing clear before I press the shock button. So ...

00:25:56

I: So, lots of things are going through your mind, and you're having to organise ...

R: Oh, they are.

I: ... and manage other ...

R: Yeah.

I: ... people. Yeah.

R: Yeah. You know, what's nice is that the call handler will normally count how many shocks the patient has had, which, which does save, save me a job. Because, as I mentioned on patient three, you know, when it gets to 17 shocks, it's like, "I think it's 17." So, yeah. So ...

I: Yeah, you can lose count a bit, can't you, I suppose.

R: Oh, 100%, you know. Especially when people are trying to do compressions, and two breaths. And so, after COVID, we, we don't endorse, sort of, layperson breaths, at the moment. So, we currently, kind of, endorse continuous compressions, which makes it easier. But, but if you are doing, sort of, compressions and breaths, it's, it's three compressions, two breaths, and we're counting how many times the defibrillator shocked, and paying attention to everyone else who, who's around us. And looking out for an ambulance, and watching the patient. So ...

I: There's, it's a lot, there's a lot going on. So, you said for the second, and the first patient, actually, that the defib was already there, 'cause you have it with you, you bring it ...

R: Yeah.

I: ... to scene.

R: Yeah.

I: And so, you used the defibrillator, in both scenarios, that you had. Is that correct?

R: Yeah. Yeah. The, the first person I was there as a responder, and so I brought it. And the second person, I had one in the back of the car.

I: Okay. Okay. And the call handler mentioned that there was a defib, as, to you ...

R: Yeah. Yeah.

I: ... (inaudible) the second patient.

R: So, the second patient, yeah, second patient collapsed outside our school, and the, and the call handler said, "There, there is, there's defib in the school, if, if anyone's available to, kind of, go and, go and retrieve it." I said, "Well, thanks, but I've got one."

I: Yeah. Yeah.

R: So, you know. Yeah.

I: Yeah. Okay. Did anyone go and get the defib, just out of interest?

00:27:52

R: No, no, I, I'd, I'd, I told them ...

I: Yeah, no need.

R: ... not, not to, it's fine. Yeah.

I: Yeah. Yeah.

R: So ...

I: Okay. And what are your thoughts about using a defibrillator in the future?

R: Yeah, I've, I've used one plenty of times. I'm more than happy to use one. I mean, it's pretty much every street in, in the area I respond, it's covered by, by a defib. Currently, currently, we only dispatch to about 500 metres. Give me one second. (speaks to colleague briefly) Yeah. Yeah, so, yeah, so, so, I promote pads/PADs (?) in the area, I promote defibs. I do free community awareness of defibs because I feel it's people not understanding defibs, and people think they will shock when they haven't got to. And also, as I mentioned earlier, people think they will always shock. So, so, when it doesn't give them a shock, people are like, "Oh my god, it, it, it, it doesn't work, and the person's dead." So, I think it, I think it, it's about promoting the fact that, if you keep doing CPR, we may well get the patient's heart back into a shockable rhythm then. Yeah. So ...

I: Yeah. Yeah. Okay. So, it's about correcting public misperceptions of (inaudible)

R: Yeah, it is. It is. And, and I mean, you know, people watch Casualty, and people watch Bondi Beach ...

I: Yeah.

R: ... and things, and the CPR in Casualty is rubbish. You know, they turn up with these big paddles, and rub them ...

I: Yeah. Yeah.

R: ... together, and shock the person. And, in fairness, you know, before I, sort of, became, sort of, first aid, you know, and defib aware, I thought that was what happened, too. People think you can get a shock from defibs, for, what we can, if you are touching, touch the patient, which is where the stand clear option ought to, kind of, be slightly more forceful, you know. "Stand clear, because ..." Yeah? So ...

I: And is there something that would make it easier for you to use a defibrillator in the future? You've said that you would want the stand clear instruction to be louder, and explain a little bit ...

R: Yeah.

I: ... more about why. Is, is there anything else you think could change, or be improved?

R: I would, I would imagine, for a non-English speaker, the instructions are fairly clear. However, you know, the, the longer sentences, you know, you know, "Attach pads to patient's bare chest, duh, duh, duh." I, I'm, I'm a linguist, and I speak other language too, and if, if those instructions had been in a language I don't speak, or a language I, I don't speak awfully well, I think, I think I, would have, I, would be, be very difficult. People also don't know what to do with the defib after they've used it. So, I mean, I spend a lot of time finding defibs, and fetching defibs, and taking defibs back to, kind of, where they live. Even our crews, called me the other day in our locality, because they, they had a defib which, which had been accessed, and have been used by bystander in a cardiac arrest. And people are like, "Well, what then happens to the defib?" You know. So, I, I tend to get a black Sharpie, and Sharpie all over mine, "Please, please take back to the ambulance station," or, "Take it back to where it came from." In fairness, the defib itself, you know, is very clear in instructions. You, you open the box, and you turn it on, and press a button, you know, and it works. So ...

00:31:44

I: Yeah. And can I just clarify if you had to leave the patient at all, to let the ambulance crew into the house, or to the school?

R: So, the ambulance crew, the ambulance crew on the first patient, I'd left the front door open. By, force, force of habit, mainly for my own safety. Second patient was in a public place, and third patient was in a house, and there were *so* many people there every door was open. So ...

I: You didn't have to leave the, the patients, then.

R: No.

I: No.

R: We, we, we don't tend to leave patients. So, you know, I mean if, if ambulance crew has to break down the door, they, they can break down the door. Equally I've been to cardiac arrests where the, the husband was doing CPR for 20 minutes, and had to run down the stairs, and open the front door for me to, kind of, come in, and then sprint back to the patient, you know. And when that happens, I was first (?), so, you know (chuckles). Bless him, he'd been doing CPR for a good 25 minutes at the time, and he's absolutely exhausted. He couldn't even speak to me when I got it.

I: Yeah.

R: So, so, I took over. I sent him in the next room to, kind of, take a breath. But yeah, I mean, you know, we never instruct people to leave a patient to go get a defib. But obviously, if you can't get in the property, people often, they have a cardiac arrest, and the front door's locked, so ...

I: Yeah. Okay. And if you don't mind, just, the, to, the remaining part of the interview is looking at your attitudes around drones delivering defibrillators, to ...

R: Yeah.

I: ... a scene of a cardiac arrest. So, is this something that you've heard of, first of all?

R: Yeah. Yeah. So, because I live in quite a remote area, which is why I've got 31 pads/PADs (?), people, people do a, do a lot of walking, hiking. We have many mountains around us. I, I always joke the fact that I don't go mountains, and I don't go into water. Equally, you know, if my partner was on the top of one of our local mountains having a cardiac arrest, I would like to think that we could get a defib to him. Especially if there was somebody with him, obviously. Yeah. In fairness, you know, the drone project is talked about quite a lot, you know, down the beaches, down [Name of area] in particular, which is very remote. I worked at, at an event a few months ago, up, up in the [Name of area]. And even though we dispatch pads/PADs (?) within 500 metres of a patient, 500 metres varies depending on whether you're in, sort of, [Name of area], or [Name of area], or, or in [Name of area], you know. If you collapse in the middle of [Name of area], you've a damn good chance of getting a defib pretty fast. If you collapse in [Name of area], best, best of luck to you. So ...

I: And have you heard of drones?

R: Yes.

I: Yeah.

R: Yeah.

00:34:56

I: Have you seen them used, or used one yourself?

R: Yeah, both.

I: Okay. And what situation have you used one yourself?

R: We, we, we have a camera drone at home.

I: Okay. For your own personal use, you mean?

R: Yes.

I: Yeah.

R: Yeah. Yeah. Yes, I have teenage kids, so it was quite an, an easy Christmas present idea, so ...

I: Oh, interesting. And how do you imagine drone delivered defibrillation works?

R: I would, I, I assume the fact that it comes from a central location, the drone is then guided to back gardens, doors, streets, locations where the arrest is, is happening, I would question whether there are always enough people around an, an arrest at first, before we get there, to, to go and retrieve a drone. Because, because, because a drone is not a big thing to find. And, I mean, our target is to be at arrest within 8 minutes. I would I'd be interested as to whether a drone could get there any faster than we can, because we will always turn up with defib. Equally, you know, as, as I mentioned, you know, I've been at arrest waiting 40 minutes on backup. But then, I was there. So, you know. I mean, if you are in the middle of a [Name of area] farm somewhere, then I would imagine it probably get there, get there faster. And then I would imagine it's a case of just taking a drone off a defib, and just turning it on. Obviously, they will have a call handler, you know, at the same time. The call handler, I, wouldn't have access to where the drone is. So, I don't know plans for giving the patient's family instructions for the drone. But I'd imagine that would have to come from, from a different caller.

I: Okay. Interesting. 'Cause drones can carry defibrillators by landing, and detaching the defibrillator, or they can hover above the ground, and winch the device down to the ground. So, a winch is, kind of, like a rope like ...

R: Yeah.

I: ... object.

R: Yeah. Yeah.

I: So, could you imagine interacting with the defibrillator in the ways that I've just described?

R: Yeah. Yeah. I mean, I think if, for example, if a family member arrested, and they were telling me the fact that a drone was, was dropping a defib in, in the garden, my instinct would be to, to go and wait for the drone, rather than keep doing CPR.

I: Okay.

R: So, that would be my, sort of, clinical concern about it, you know, you know, if I have one. And I, and, and as a layperson, I would find it hard, sort of, leaving, leaving my dad to, kind of, go and find this random drone which is dropping out of the sky, with the defib on it. Equally, you know, as far as location goes, how would the drone be aware as to whether there was a back garden, a front garden, you know, a local authority housing estate, or driveway. So, I think, I think a drone would work, as long as there was a third person on scene.

00:38:15

I: Okay. And ...

R: Because, you know, yeah. I, I wouldn't be wanting any, anyone to be instructed to leave a patient to, kind of, go and wait for the drone that's outside, to go and get a defib.

I: Why is that, then?

R: Because it's time, it's, it's time off chest. And, and time off chest doesn't contribute to, kind of, positive patient outcomes.

I: And why would you feel that you would have to leave the patient to, to go outside, then, to ...?

R: Because the drone couldn't get in the locked door. So, so, you would need to be guiding the drone through a, assumedly open door. If you even, you know, if you, if you want to be dropping a defib into a house, then, then that requires an open door, or open window. Alternatively, if you're dropping it, you know, in the garden, then, depending on where you are in the world, the garden’s quite far. So, you know, if, if I was doing CPR in my parents’ lounge, there is no way a defib could be dropped in by a drone. It would have to be in the driveway, or in the garden. And by then, they, there will be, there will be blues outside.

I: There would what, sorry, they would ...?

R: There would be an ambulance out.

I: Right.

R: You know, outside.

I: Yes.

R: Yeah. Equally, you know, if I'm up in the, in the middle of a mountain, up [Name of mountain], by all means bring us, us a defib, so ... Because by the time, by the time we give what, what3words to the call handler, and the ambulance works out how to, how to get there, you know. Issue is ... Yeah, and this, one of the misconceptions about defibs is, is that a defib doesn't save a patient. A, a defib is part of a long chain, you know. Which includes prompt backup, and hospital access. So ...

I: Yeah. Yeah. You feel that a lot of bystanders, they put a lot of faith in a defib.

R: Oh, they do. I, I've, I, you know, I've been to so many arrests where, where, where/when (?) a defib has shocked, they, they all, sort of, relax, and look at the patient, or ... well, the patient is still dead, mind. You know? So, like, you know, you know, "Yes, it was (?) a shock, however the patient is still very unwell, and I'm gonna keep doing CPR." And they all go, "Oh, but we've given him, him a shock now." And in all these films we've watched, in Casualty, you give them a shock, and they, and they sprint up, and ...

I: Yeah.

R: ... you know, and start, and start eating toast, and drinking tea. You know. Where, actually, even, even if it, you know, it does its job, it ... Sometimes our patient will, will start talking to us, and that's great. But, you know, you know, I've been to many arrests, and very, very few have gotten out of hospital.

I: So, how would you feel about a drone bringing a defibrillator to you? I think you've touched upon it, when, this question when you ...

R: Yeah ...

00:41:22

I: ... talked about your parents.

R: ...  I think, I think if, if it got there before the arrival of an ambulance resource, yeah, that's fine. I think, my concern is that I'd be expecting the drone, the ambulance would turn up, and then I say, "Oh, one second, I've been told to, kind of, go, go and get a defib off a drone." And I think, as an ambulance crew, they'd be like, "Well, that doesn't matter anymore, because we have one. But the, the bystander, the, the layperson who hasn't been to an arrest before, has been told by the ambulance control call handler the fact that there is a defib coming. And that's what they're thinking about, you know. I, I think they'd probably run past the ambulance crew coming into the house, to, kind of, go, go, and get a defib. So, I think, so, I think if this isn't gonna get there before the ambulance crew does, there isn't any point, you know.

I: Interesting. Thank you. Would you think it'd make it easier, or more difficult for you to use a defibrillator if one was delivered to you by drone?

R: For me, personally, it wouldn't affect me. For, for the people in the area I, I respond with, it would *blow* their minds. You know, the defib itself is a very odd concept anyway, and the fact that it's being brought in by some flying thing ... You know, I live in, in an area where people are not as, as educated at times (?), are in poverty, they are elderly. I, if I told them the fact that there is defib coming via a drone, they'd probably think they stepped in, into Star Trek.

I: Yeah. Yeah. So, again, it would be important to get the public on side with this, and tell them about ...

R: I think ...

I: ... it publicly.

R: Yeah. Yeah. I think it would be a hindrance, because, because we haven't yet got the education about, about a defib, let alone the fact that it's gonna magically appear by drone. And then, I think even if it was (?) my parents, and my parents have, you know, have, have master’s degrees, etcetera, you know. If, if my mum was doing CPR on my dad, and she told, there was a drone coming, she, she wouldn't want to leave dad to, kind of, go and fetch it. She wouldn't be opening doors, you know, in, in, in case, in case the drone came. She'd probably be panicking about what to do with the drone when it came, and what to do with, with the defib when it came. And, and by the time that all happens, she's stopped doing compressions, because she's stressing about, about a drone. Dad's had 10 minutes without compressions, and the outcome isn't gonna be great when we turn up, in seven minutes' time.

I: And how would you feel about leaving the person if, if you're by yourself with them? How would you ... You said that other ...

R: I ...

I: ... people wouldn't like it.

R: Yeah. I wouldn't, I wouldn't be leaving a patient. So, whether, whether as bystander, or not. I mean, a friend of mine who is also a volunteer CFR, basically did CPR on his father-in, in-law. He wasn't stopping CPR for, for anything. So, you know. And, you know, as, as volunteers CFRs, we, we are taught, you know, we, we don't go and get a defib if, if we're by ourselves. Call handlers are taught, don't send anybody for a defib if they're by themselves. Because, because, you know, you could go and get a defib that's fine, but, but you've left your patient who, in the meantime, has, has choked, potentially. Hasn't had compressions going on. Heart isn't pumping. Blood not get, getting around. And, and therefore, outcomes are going to be probably worse than had they just stayed and done adequate compressions. (sneeze) Excuse me.

00:45:10

I: So, how far, if at all, would you be willing to get the defibrillator that had just been delivered by drone? I'm guessing ...

R: I mean, if we're talking about my parents' house, and they're, and they're collapsed in the lounge, you're probably talking 30 yards away, you know. If it, you know, if you dropped it outside my parents' front door ...

I: Yeah.

R: ... and dad had collapsed in his chair in, in the lounge, then chances are, you know ... Normally, normally if patients can see things, they are more inclined to, kind of, go and get them, you know. Even, even as (?) kit. I mean, I have left bags in the corridor before going in. I've tried to turn to a, you know, I've turned to a relative, and I've said, "Do me a favour, and, and, and pass me the bag," you know. If they can see the bag, they, they are happy to fetch it. Equally, you know, as I mentioned, if, if, if I'm on a beach, or in the middle of a mountain, I can imagine the defib would, would be handed, you know, on a plate, because, because there isn't anything stopping it. You know, if you tried to deliver a defib drone to the first patient on the local, local authority housing estate, it probably would've been stolen first, so ...

I: Okay. So, there's that issue as well, criminal activity ...

R: Oh, yeah.

I: ... around the drone, and people stealing ...

R: Oh, yeah.

I: ... it. Theft, and ...

R: Yeah.

I: Okay. So, would you get the drone from the doorstep then, if it happened in a house? You know ...

R: I'm trying to think of all the houses I've been to with an arrest, and the doorstep is too far away.

I: So, no. So, you wouldn't leave ...

R: No.

I: ... at all.

R: Nah, I would, I would not be leaving a patient when, I'm there as a bystander, or as, [Name of NHS Ambulance Trust] ... I mean, there, there is an emergency resource coming, with a defib. So, you know, if, you know, I, I think it is back to, kind of, geography, and locality. If you're, if you're up [Name of mountain], by all means, send them a defib, you know. There's one in the visitor's centre, and there's one halfway down on, on the pub, you know. That's, that's where they can be very useful, you know. If you know that the nearest (inaudible) resource is coming from [Name of area], and I'm in [Name of area], then yes, you know, they (?) will get there first. Most arrests I've been to, I mean, we went back, you know, I've been on scene within 10 minutes, and the, and the patient's either been on their own, and we had to, sort of, you know, make entry, or the, or I've been the first, first on scene, and I bring a defib. Personally, I don't think encouraging patients, encouraging relatives to leave the patient to, kind of, go and find a drone which they aren't familiar with, works.

I: What ...

00:48:03

R: And also, if the drone is dropping the defib, patients don't know what a defib looks like. So, you know, up in [Name of area], where I respond, we have seven different types. I think, I think people always go, "Is it orange?" Like, "Well, it might be orange. Or it might be yellow. Or it might be blue. And it may be purple. If you're really lucky, it's, it's gonna be black." So ...

I: So, that's another problem, potentially. It could drop ...

R: Yeah.

I: ... the drone could drop the device, but then bystanders just aren't aware that that's happened ...

R: No.

I: ... because they don't know what to ...

R: No.

I: ... look out for, in the first ...

R: No.

I: ... place.

R: Yeah.

I: Yeah. Okay. Would you go and get the defibrillator though, if it was outdoors? Would you leave the patient in that scenario?

R: So, I wouldn't expect to be leaving the patient. So, if it was outdoors, I would expect it to be dropped next to where I was ...

I: Yeah.

R: ... standing.

I: Yeah.

R: And I would imagine, you know, I imagine our navigation systems, or our defib are, are capable of doing that. You know, whether it's a tourist location, or, you know ... I mean, I mean, if you are in the middle of nowhere ... I used to be a white, white, white water paddling instructor, so, you know, I've dragged people out of boats down [Name of area]. I had no idea where I was at the time, you know. Now we have what3words in theory, you know, a drone could drop a, drop a defib to a location there. But again, you know, it's, if you asked people in the area to respond as to what a drone is, they wouldn't know. They have some idea about defibs, because god knows I've been working my socks off for the last seven, eight years, you know, in the area. But then, equally, people, people are afraid of things they, they haven't met yet. And if you drop it in the middle of a field, it's gotta be a bright orange box, about this big, flashing at them. And then the question's gonna be, "Well, how do I open the box? How do I get in? What do I do with the box? What do I do, what do I do with the defib?" I mean ambulance crews aren't keen to take anything with them, which doesn't belong to the Trust. So, even defibs, you know, I've trained all of our guys, when they turn up on scene in my locality, and there's a defib there, "Please pick it up, you know, you know, and call me." Who is going to walk back up [Name of mountain] to, kind of, fetch a defib? So ...

I: If the drone ... Well, yeah, I, I guess it ...

R: Yeah.

00:50:32

I: ... depends where the drone delivers it. If it's halfway up the mountain, or, or ...

R: Well, yeah.

I: ... wherever it would ...

R: You know, can, can a drone go back and rescue a, a defib? So ... potentially yes. It depends how it's strapped on. But, but yeah. And, and even if it's in a garden, you know, in the LA housing estate, I was at with the first patient, good luck dropping anything there, because there was so much stuff round. I probably wouldn't, wouldn't have identified it, you know. Bags of rubbish, rats everywhere. Sharps on the floor, you know. What'd you do if someone is, gets a, a needle stick injury picking up a, a defib off the floor, you know what I mean? So, I think, you know, there are definitely cases where a defib drone's a very, very good idea. My concern is, is leaving patients, getting access, identifying defibs, because people think it, it's a massive scary clinical machine. So ...

I: So, in circumstances, and in ...

R: Yeah.

I: ... certain situations, and places, it could be beneficial, such as outdoors ...

R: Yeah. Yeah.

I: ... but if you're indoors, then you think that it would have limited use, or limited ...

R: Yeah.

I: ... value. Is that, kind of ...

R: Yeah. Well, you know, by the time you leave the patient, you push past the dog who's been locked in the kitchen, you open the baby gate, you open the back door. The, the drones dropped, dropped the defib by the front door. So, close all that, and go back again. And, and then go to the front door to find it, and then open the door, and don't identify it, because it's, it's, it's, presumably, in a cosy box. You then get the box, and bring it back, and don't realise you've gotta open the box to, kind of, get the defib, to, kind of, open the defib. You know, this, while I'm very, very defib proactive, you know, I think you, I think we've gotta remember we're dealing with the general public here, who are, you know, bless them, not accustomed to, kind of, such things. Equally, as said, if I'm, if I'm on a beach, up a mountain, you know, in a field ... I mean, farmers are notoriously bad about calling us, bless them, because farmers just get, get on with it. I've been to plenty of farms, and remote locations. A defib is going to arrive first, but, but I, I'm also going. So ...

I: And what do you think the role of the call handler is in all this?

R: The call handler has one job, and that's to follow the script and take information. It's the allocators, and the dispatchers who will be dispatching our trucks. I don't know who will be dispatch, dispatching a drone, but the, the patient expects, the, the patient or relative expects the call handler to tell them who's coming, when they're coming. They'll always stay on the phone with them, until, until I arrive, for example. And the patient, patient's family is normally standing in the doorway, going, "Yes, yes." And the crew are walking up the drive. I don't think there's capacity there, personally, you know, having been, been in control, I don't think there's capacity there to, kind of, also talk about a defib, and, and update them on the fact that, the fact that I'm coming. You're gonna

00:54:01

have to have extra resourcing control, just handling defibs. Plus, you're gonna have to have a defib operator in a, a location, you know. I don't know where the locations are, intend to be, but I think the, the back end comms don't affect the patient. So, you know, so much happens on the back end, while the call handler's going through the script, they would have to have a script line which would say, "By the way, we are sending you a drone." And they then have to cope with the fact that most of their callers won't understand what a drone is, what to look for. Is it a black thing? What do I do with it when I'm finished with it? You know, I think, personally I think if you've got a call handler doing that, it would detract from the other advice the call handler's giving. Because people are very big on, on asking Siri for advice on CPR. However, the call handler does give clinical advice on what to do with a patient. So, personally, I don't think the call handler has, has any capacity to, kind of, be coping with instructions, as well.

I: No. No. So, you don't ...

R: So ...

I: ... think the call handler should tell the person that the drone was coming, or how to identify it? You don't think that that should ...

R: I, I think that would only be appropriate if they weren't giving out the clinical advice at the time. Because they will tell them a resource is coming, and they'll go through the script questions. But I think, you know, if they're giving CPR advice as well, they can't stop that to, kind of, talk, talk about drones.

I: So, you think it would be a, it could be a distraction, the drones.

R: Oh, *100*%. Yeah. Yeah. However, you know, you know, I'm mainly, sort of, focusing on people in houses who, who have ongoing CPR, because, you know, they, they will have to be doing CPR anyway, to, kind of, activate a drone. Because, because if not, it isn't a cardiac arrest. And then, why, why is defib being dispatched to them? You know. In best case scenario, you're on a farm, there are seven people around, they're all having a party. Somebody arrests, and there are plenty of people to, kind of, go and find, find a drone. Happy days. And they're all of a certain age, and they know exactly as to what a drone is, and they're more than happy to, kind of, give it a go, you know. And job done. But unfortunately, this, this isn't a perfect world, and most of our people who arrest are, well, 80, 80% of arrests in this country happen at home. You know. You know. Survival rate, rate out of hospital is 8%, if, if you happen to get a defib straight on with, with, with good, good compressions. So, so, if you stop your compressions to, to go and track down a drone, plus dogs outside, and the dogs attacked it first, you know. It's just, you know. It's, it's a really good idea, but in certain, certain circumstances. And where there and where there's a person available to go and fetch it, because we will never stop a person doing compressions to go, to go and fetch, fetch a defib.

I: No, no. So, the second person should get the defib from the drone ...

R: Yeah.

I: ... while the first person is ...

R: Yeah.

I: ... performing the CPR.

R: Doing compressions, yeah. And, you know, I've, I've been to arrests where people have gone and got a public access defib, because, because we dispatch, we're in a very small area. And if there are plenty of people around, yes, yes, it can happen. And so, perhaps the question the call handler would have to ask is, “how many people are there?” Which would then activate a press, press button of a, “can, can I please have access to a, to a drone button?” How does, I mean, you know, even postcode, if you tap in the postcode of, of the school I'm involved in, that's a big area. You're gonna have to have a decent location. You're gonna have to have a GPS, or a what3words. And I'm not stopping compressions just to give a call handler, you know, a, you know, a location, so, you know. I can, I can have a call handler on speakerphone, but, you know, by the time you tell them to stop, download an, an app which, which they haven't got yet, most of the time. Open what3words. They haven't used before. You've been off the chest for, like, a couple of minutes at this point, so ...

00:58:26

I: When you talk to the call handler, do you usually have them on speakerphone, or ...?

R: Yeah.

I: Yeah. Yeah.

R: Yeah.

I: Yeah. Yeah.

R: Because I'm doing compressions ...

I: Yeah.

R: ... you know. And, and in fairness, that's part of the call, call handler script, is, is, you know, stick it on speakerphone, if we aren't already, because we could be giving instructions. So, you know, and then the patient is sick, and I have to turn them on their side, and then they start making noises, and so, stop compressions. And then they arrest again, so they go back on their back, and we start CPR again. And then, we turn up with, with bells and whistles, and the defib, you know. All police cars in this area have a defib now, as well. So, so, as do the boys on the fire trucks. So, if they're gonna have an emergency responder, they will have a defib with them.

I: Yeah. Okay. Thank you for that, that's really interesting. I just have some final brief questions that may ...

R: Yeah.

I: ... be useful for our research. So, if I may ask you, what is your age and gender?

R: I am 42. Hang on a second, and I'm female.

I: Thank you. And you've had training in CPR and defibrillator use?

R: Yeah. Yeah.

I: Yeah. In both?

R: Yes. Yeah.

I: Yeah. And when was this?

R: I think my first training, you're talking when I was about 12. And regular first day of work sessions, and then, and then I became a volunteer community first responder in [Name of NHS Ambulance Trust] in [year]. So, regular sessions, and all refreshers I teach it myself. We do free community awareness sessions. I trained a whole pub watch the other day, in the area I, I respond in. As, as a career, I also train our Trust volunteers as well, so ...

I: And when you became a volunteer first community responder, was that before or after the incidents that we've discussed?

01:00:33

R: One, one incident was before. Yeah.

I: And the training ...

R: So yeah. Yeah. So, yeah, I mean, you know, we, we are trained to a very, sort of, you know, high standard of first response. I've also done private, you know, medical qualifications as well. I also, I also responded at events, you know, sort of, boxing, horse racing, community (?) fairs, you know." So, in fairness, I've done a lot of CPR, I've done a lot of defibs and I do it automatically at the moment, but I train people who haven't, and it's really interesting to, kind of, watch their reaction. I was at a, I, I spent nine hours on [day of week] at an event, just, just interacting with the general public. And all the kids had a go at a CPR and a defib quite happily, and the parents weren't, weren't happy to, because it was scary, and new. So, you know, I mean, I had, I had tiny kids coming up who were like two, having a go at CPR and defibs. I didn't stop all day, apart from one ice cream over lunch. And I was literally teaching CPR for eight hours, on the trot.

I: Wow. Wow.

R: So, yeah. And, you know, while the family were watching, I pointed out to everyone, that it's the family who's gonna be doing this. Because kids know exactly what to do, but they haven't got the strength to, kind of ...

I: Yeah.

R: ... to do it properly. So, yeah. Yeah. We, we, we do a lot of awareness, in the area I, I respond.

I: Okay. And is there something else you'd like to add to your answers?

R: I don't think so, you know, I mean I'll be really interested to, kind of, get the outcomes of the, of the research. I'll be really interested to find out where the defib locations are going to be, and where the drones are coming from, you know. Obviously, they can fly. And, and I know the, I know the [Name of organisation] guys and the ambulance charity has had a, has had a lot of bad press recently, because they have moved stations of their, their helicopters. I mean, the fact is, if the drones are close enough, they could come pretty quickly. But I, but I wouldn't like the drone to arrive at the same time as the, as a emergency responder. Because there would just be absolute carnage, you know. I would have people pushing past me to, kind of, go and get a drone with a defib on, because that's what someone's told them to *do*, while I'm explaining the fact that I've got a defib with me, and trying to find the patient. Like, you know, it would be a perfect storm. So, I think, you know, it's a really good idea in certain locations, with certain time responses. If defib is going to get there first, and there's capacity to go and get it, and clear instructions to give them, fine.

I: So, gotta be carefully implemented, and ...

R: Yeah.

I: ... yeah. Yeah.

R: Oh yeah.

I: Yeah. And be aware (?) ...

R: And tested first.

I: Sorry? Yeah.

01:03:26

R: And tested and trialled first.

I: Yeah. Yeah. Yeah. Of course. Yeah. Yeah Okay. Well, thank you. I'm just going to turn off the audio recorders, and I know I've ...

R: Yeah.

I: ... taken up too much of your time ...

R: Oh, no. No, that's fine.

I: So, I'll just turn this off.

**END OF INTERVIEW**

Participant 8

I:  So the main interview audio recording has begun, and it will continue until the interview questions end. Okay. So let's start, then, with the first question. Can you tell me as much as you can about when you provided assistance to the person who had a cardiac arrest?

R: Yes, okay. It was in our neighbourhood. So not like a public space though. So it is actually came out, it was, it was late in the evening when I came back from work. So, I mean, it was (inaudible) [time]. So the day was too bright. So it's not like it was dark. So it is happen in the neighbourhood. And I just, me coming back, I just saw people crowded and an individual was like, "He's on the floor." So, "What's happening? Can they rush him to the hospital?" So, and the hospital is not close by. So I just have to walk toward them, in their direction, to look what's happening. So I got (inaudible) that he is always struggling with breathe and he's in a critical stage. And everyone is (inaudible), although this, the CPR is not something I've done before. That was my first time actually doing something of such. So at this, a, a lot of people got into my head. So, and we didn’t want this, this young man here die or I wouldn't have got into this, no, take an approach, take a step, just to make something happen before the ambulance service, like, the 911 (sic) arrive. So everyone was just scared. Yeah. I, ok, I was really scared because *what* if something goes wrong? What if something goes wrong? What is in the process of doing that, something goes wrong and the person dies? The, what if the blame is not put on you? Maybe you did something wrong in the process or maybe at the process of you doing the heart beating, you over, you do it over and, you know, the person happen to give up at the, at that time when you were there? So that was a lot of thing that went through my head. But I, kind of, (inaudible). My dad, my dad is a, is a, worked …

00:02:24

R: … in the healthcare sector. So he told us about the CPR, although he, he didn’t actually advise us to engage in such. But to say, you know what, in the right cases we can give it a try, just to save someone life, just to be (inaudible). I have to pick up my phone and call my dad and … but this was before my dad arrived. It was a longer period of time. So he thought, "My son, you have to do that." So, "So *what*, what, actually what do you want me to do?" Be something that I haven't done my entire life, since my, I'm younger to now. I'm in my late 20s. So how can I do, do something as such? But he said, "Okay, do you wanna watch him die or do you prefer doing something?" I was like, "This, this young man is *lifeless*, so there's nothing I can do." He said, "No, this, although your chance of survival is, is just, is just 10% out of 100, or maybe 20% out of 100, but you can just do something before the ambulance service have arrived." I said, "Okay. Okay. Okay. I will do so." Everyone was shock (inaudible) step back. So I, "Okay, can everyone just shift back a little bit? Can you just go back? Okay, everyone just shift back." So, "*What* are you doing?" So I, I, I, I pull out my phone, I call 911(inaudible). I say, "Okay, just be on this call. Hold this phone for me so they can tell me the directive on what to do, because I might not be a lot more perfect." So I, I, I told him, brought up closer to me. "Okay, put my phone on speaker, so tell me the direction and how for me to proceed." "Okay." I said, "Okay, yes, I will do this." Although at first my hand, I felt a shaking in my hand, like my hand just vibrating on its own. So, so I have to hold my hand tight. I knew (inaudible) "No, you have to stop this." I had to take a deep breath first because (breathes out) I just need to breathe well to do this. "So, okay, I need to check his pulse now." The, the person on the call, "Okay, yeah, you know what to do now?" I said, "Just tell me what to do." "Okay, you have to lay him flat. Turn his body so it just be flat." "*What*? *How*?" "Just turn him so he can turn (?) flat for you to be able to perform this (inaudible). Just let him be flat, right on the floor." I said, "Yeah, okay, yeah, the person is laid flat." "Put the person on the back (inaudible) flat surface." So tipping my hand back (?), I'm tipping, you know, there's a way I, I cupped (?) my hand to do that (inaudible). So, but there was something funny that we've been taught to do that. I think, to some extent, it gave me, you know, some kind of firm, firmness. He told me to just think in my head, "Stay alive, please stay alive." So he said I should just keep, I will just, "Keep repeating that, that sound in your head. "Stay alive, please."" So even if he's, he's not a family member, "But just say, say something to help, just some stuff to just move you to, you know, keep tipping that nonstop." Then I was like, "Okay, please stay alive. Please stay alive." I was saying it in my head while doing the tipping. I was saying this continuously while doing the tipping. And I was being told about some defibrillator that was gonna be delivered to me. I, I, I, I haven't made use of that. I don't know what, I don't know what, how to make use of that. So I said, "No, you, you just need to arrive here before you do that." So I, my dad told them he was coming. So I said, "Okay." So I have to do that continuously until my dad comes(inaudible) and he, he helped me a lot more process. Even before the ambulance arrived, when they brought the defibrillator to, down (?), he was the one that, you know, assisted me throughout all of the process. But he was the one actually doing it, so I just assisted the process up to that. So that is how it went. It's something that I wouldn't say I would love to do it again, but it is something scary that I, I don't wanna find myself in a situation whereby I would be the only one to be able to cough up courage to do that kind of a thing. Yeah.

I: Yeah. Yeah, well that's understandable. And, and thank you for sharing those experiences. That does sound like, you know, it was, it was a very, you know, difficult time that you were experiencing. Are you okay to continue with the interview?

R: Yeah, sure.

I: Yeah. 'Cause I, I know these things can, can be upsetting, talking about, so I just want to make sure that, that you're okay to talk about it. Okay. So just going back a little bit, are you saying that the cardiac arrest happened in a public space in your neighbourhood?

00:07:22

R: Yes.

I: Yeah. Okay. And the person was a stranger. The person who had a cardiac arrest, he was a, he was a stranger to you?

R: Yes.

I: Okay. And did you realise what was happening at first? When you saw this person become unwell, what was going through your mind? You know, did you realise that he was having a cardiac arrest?

R: Sorry, can you come again?  (inaudible).

I: Yeah. So did you realise what was happening at first? Did you know it was a cardiac arrest when you saw the man become unwell?

R: (no audible response)

I: Sorry, did you …

R: Yes, I …

I: … hear me? Did you hear?

R: … I, I, thing is, there was some, some (inaudible) some kind of (inaudible). Like, I, I told people, "What, so what's, what's going on for us?" So they said, "Just sudden loss of conscience." So it was more like a heart attack or, or so. I, I'm familiar with that sign. I've been told about the sign. Because I've been told that the two signs of this cardiac arrest, it's when, it, it, it's, it's, it's either (inaudible) sudden loss of conscience, a lack of pulse, and not breathing suddenly without any, you know, no *accident* or, you know, it's not like an accident occurred that made you, that make something (inaudible) happen. It just happened suddenly. I, I was just asking people close by. They say, you know, they were feeling dizziness and saying chest pain. So I was trying to, you know, compare all of these signs which they told me that he (inaudible) according to him before the, before now. So, so that was when I have to (inaudible) that maybe it's just the cardiac arrest (?). And I, I now just know, yeah, it is.

I: Yeah. And, and how soon afterwards did you realise it was a cardiac arrest?

00:09:31

R: Oh. A lot of things go through my mind. So maybe he, he, it might be as a result of something he did that caused it. Or I was thinking, "What if he was unwell already or he had some kind of stress disease or sickness? So how sure am I?" You know, one thing I was saying, one thing is it's not like I was being given some confirmation that it’s a cardiac arrest… I just have to determine this myself. So I just have to do that after, you know, find out the symptoms. And this is, you have to do that in a hurry. It's not something that they give you a lot of time to analyse, to know actually what the  'cause. So this thing just happen suddenly and you have to make some quick decisions. So although I wasn't a lot more confident that it may be a cardiac arrest, but because (inaudible) told me about, I just thought, "Okay, yeah, yeah, yeah, I think, yeah, it's cardiac, so something needs to be done." I said, "(inaudible) this just needs to be done quickly, immediately. So who can do that? Who can assist with that?" So everyone just give me some kind of face, like. And I, I, I look at that kind of face, that kind of face means here, "No, I don't wanna do that." So, but, you know, "This is to save a life. So someone need to just (inaudible)." But everyone just keep doing that face, like, "No, no, I don't want to be the, I don't wanna be, be a, be a victim here." So I say, "Okay, but something need to be done because this collapse suddenly isn't normal. This lost consciousness and just pass out like that isn't normal. I think we need to do it." So that was when I, I have to cover that and (inaudible) happen. And, yeah, this is definitely the (inaudible) and the ambulance service did arrive and they've been taken out. Even after he, I feel that it was normal, I said, "No, you still need to go to the hospital, you just need to go run some tests. We can't let you go like this," even after he pick up and, you know, collapsed (?), having caught his breath back. (noise – breathing in and out rapidly) So, "Oh, okay. Are you okay?" "Yes, I'm fine." "But you, you just need to go to the hospital, you just need to do some check-up." "I'm fine." "No, you are not fine, even if you just got some, some breath back. But I don't know, maybe you're having some blockage in your, your … I don't know. I'm not a doctor. Maybe your (inaudible) or something or (inaudible). So, but you just, listen, you just need to go now to the hospital." But the ambulance service have arrive and (inaudible). So yeah, that is how it is.

I: Yes. And, and did he go to the hospital then?

R: Yes, he has to, because we can just let him go home at that stage. What if … we, we didn't plan for such. But what if it happened again, where no one have to take the step? That means that's the end of your *life*. So even if you feel that you're normal or you have catched your breath, you just still need to go to the hospital. I think it's something important. Yeah.

I: Yeah. Yeah. And was he okay when he came? Do you know what happened to him when, after he went to the hospital?

R: I don't really know the rest of that process is because I, I didn't actually accompany them to the, to that. So I just did the part which I needed, needed, needed to do and I left the rest for them.

I: Yeah, sure, sure.

R: But the next, next day or so, I still, you know, go towards that, the place to ask, "So how was it? How," (inaudible) "Yeah, thank you so much for doing that. Thank you so much. You saved a life." And I was like, "Wow, I really saved a *life*." So it's something that makes me feel like I did something good, so nice. But that was just how the whole thing went.

I: Yes. Yeah. That's, that's good to hear, that he was okay. I, I'm just wondering if you had immediate access to a defibrillator?

R: Actually, I didn't make use of that in (inaudible), but it was being delivered to a van (?). So the fastest way was being delivered to a drone. It's, I, I don't know how to describe it. It's an object, it has four, two front, back and …  should I say an small helicopter. (inaudible) but everyone saw this coming over. "What, what this? What's, what's flying? What's coming towards us?" So, but I was, "Okay, everyone just stay back. Everyone just go, go back, go back, go back. Just need to make it (inaudible)." And all of these processes, we were still on the call. Yeah, the person was still calling on the loudspeaker, so (inaudible) to aid us. But the, the aid, it wasn't something difficult. I thought it, aid, why I was scared initially, I thought it's something that you need to be a professional to make use of that. It wasn't. It was even, you know, having some voice sound for direction. Like, "Wow, this is," it was very good. So it's, like, it's, it's, it's another sound for you to, you know, directive on how to (inaudible). So that was how it went.

00:15:18

I: Okay. So you used an AED?

R: (no audible response)

I: Sorry, you've gone on mute.

R: Oh, sorry. Can you come again?

I: Sorry, so did you say that you used an AED?

R: Yeah.

I: Yeah. Okay. And I'll ask you about your experiences of, of doing that in a minute. So you said that there were other people there at the scene. Is that correct?

R: Uh huh. Yeah.

I: So how did that make you feel?

R: It makes you feel like, "Okay, if something bad were to occur, there are evidence." And that was, it have, makes me have a, a (inaudible) a groups of (?) fear, because if I were alone, I, you know … but to some extent I was just, I was just thankful that there were people around. Because to, to face alone, I, I, you know, I just (inaudible). The emotion alone was, like, something that drives me to, you know, to take a step. And I, the assistance which I got from the third brother which helps me, and the whole holding of the phone, doing the call, actually putting on loudspeaker (inaudible) was something, yeah, it's, it quite … I know only me cannot do all of that. I have just two hands. So I, I can't use, I, I, I, I can't, I don't have three hands that I would say I'd make use of. So for the compression, I'll make use of one to go my phone. So I just have to have … so if there are no one around, that would have been one of my worries too, to just be reluctant. But because I got assistance so, yeah, I just need to do it and someone is here to assist me. So …

I: Okay. So what, what did they do then? You said that they provided assistance to you. So they held the phone, then, or is that what you mean? What, what did they do, these other people?

R: Yeah. The, the third brother helps me with the phone, during the calling. So putting in the loudspeaker and just, you know, give me some equipment, what, yeah, yeah, just, just trying, I, and, "Please, just, just, just help him. Just," you know, there was some stuff like, "Please just help him." I'm like, "I'm not a *doctor*. I'm not, I, I'm, I'm not even a health professional. I haven't worked in that sectors/settings (?) my whole life. So how  …" He just, "I, I know that you can just do something. Please just help him." I said, "Okay, yeah, sure, I'll try my best." So, but some, some (inaudible) was coming out. And even after raising my head up, I saw the faces of the people. It, it, it drew some compassion in, in me. I've become so, "I, I, I really must do this successfully. I really must do this, even if not for myself, but for the, for the fact of these people looking at me. They, they are, they are, they are looking at me to do something, so I really must do this." Even after having groups of fear but fear but I still must (?) do it. So that was how, that was the feeling I was having when, you know, these people were, I saw these people faces.

I: Yes, they, they reassured you. And how did they organise themselves?

R: (Pause) Sorry, how specifically?

I: How did they organise themselves around you? So did some go off to get the defibrillator? Did some stay? Did some move back? Yeah, how, what did, how did they organise …

R: Yeah. Yeah, I …

I: .., them? Who did what? And how many of them were there?

00:19:32

R: Yes, I, I, I told them actually, "Okay," I told them, "Okay, please can you just go, can you just move further away, some, some, some metres?" so I can just, you know, feel, being, I, I don’t really know what to do but to just make me feel a little bit confident or so. So I just let people move back a bit. But, you know, (inaudible) in order to come closer. So, so, "Please just move back a little. Please just move back." So the, the only one that was beside me was the one with, making, the, the third brother, who was calling and helping me, putting the loudspeaker on, yeah, so would, I would hear clearly. He, he repeats it after them. So they said something, but I can't just hear. "Can you say that to me? You have to repeat what he said for me." And even when my, my, my, my dad came (inaudible) fan in terms of support. So he helps even in … and when the, the defibrillator came (inaudible) so he helped me the whole process of going (inaudible). Because everyone was like, "What's, what's this coming towards us?" So he told, "Please stay back," because I think it was  (inaudible) a little of breeze now (inaudible). So we have to move further away for it to land before you have to retrieve it. And so, so it was one they have to (inaudible) after it reached the ground. So even after all of these processes, we were still calling.

I: Okay. And could you tell me, I know we've mentioned it already, but could you go into some more detail about the 999 call?

R: (no audible response)

I: So you said that you made the call initially on your mobile?

R: Yes.

I: And you put …

R: Yes.

I: … and you put that on speakerphone.

R: Yes.

I: Okay. And did you say that you spoke to the emergency call handler, or what, were the other people around you doing that?

R: No, it was the one speaking. But my third brother just helps me, repeats a word if I'm having some, you know, some difficulty, yeah, with it. So I was like, "Okay, what's the next turn (?)?" "Okay, do compressions or we're going to lose circulation." Or, "Okay, position the patient on the back on a firm, flat surface. Okay, tipping, tipping your head back (inaudible) the airway. Okay, just listen for the breath sound and feel." "I'm not, I can't listen. I think my ear isn't open enough to listen to it. But how, how am I able to listen to breath?" So I, I, I'm not some kind of health professional so that I'll be able to, you know, use my ear to, to hear, to hear sound of breath or something. "No, just place your ear, your ear down. There will be some kind of tipping." (inaudible). "There will be some kind of breaths (?) you will hear. So if you have that, just …" Say, Okay, okay, okay." And they say, "After every 30, after every 30 compression, give two breaths or so." Okay, I was being told. I'm trying to remember. It been quite some time now. "Okay, please press the nose (?)," (inaudible) and give one breath through the mouth, lasting about a second. So that was things that I was being told. It was through the phone. It was through the phone to do this. So after I did, "Okay, now just continue tipping, doing constantly the compressing (inaudible)." I was, so that was how I continue, I continuously doing it. And, and I will keep doing the tipping and so.

00:23:24

I: Okay. Okay. So did the call handler ask you any questions?

R: Not, not really. (inaudible) I was the one like asking me what did they say tell you to repeat that "Okay, okay, okay have, listen the breathe." How I'm listening breathe?". So it was more like something collective. So the assistant really helps me a lot. So, so yeah.

I: Yeah. And apart from asking the call handler to repeat themselves, did you ask any other questions to the call handler?

R: (inaudible) not question specifically(?).

I: Okay. And did you have to leave the, the man, the patient to make the call?

R: Sorry?

I: Did you leave the patient's side to make the call or did you stay …

R: Oh, no, no.

I: … with the patient?

R: Oh, no, no, I didn't. I, it was still same range.

I: Sorry, could you repeat that?

R: Yeah, it was still in the same range.

I: Okay. And what are your reflections on the interaction between you and the call handler?

R: I'm sorry, can you explain that a little?

I: So what do you think went well and what did you find, if anything, difficult, talking to the call handler?

R: Okay. Actually, there wasn't anything that was difficult or that, that caused, seriously for, you know, for me to (inaudible). So I, I just feel that there, there, there were these, kind of, connectivities. So it was, it was not like the person was (inaudible) for us to do it. I, it's not in the person mind to do that. So he doesn't do that because he doesn’t wanna do it (?). So everyone needed to serve this person in question. So we all was working in (inaudible), we all was working hand to hand. So there was no form of reluctancy. I didn't actually feel any form of reluctancy in that. So it's not something really that caused me to have any, any negative energy or any bad energy from anyone. Everyone was just giving me that positive vibes and that collaboration just to make this work well. So for me, there, there weren't.

I: And does that include the call handler then? You felt that they were encouraging too?

R: Mmm hmm.

I: Okay. Were there any technical issues while you were talking to the call handler on the phone?

R: Actually, there, there wasn't any types of issue that caused for concern because any time, any of which I didn't actually have. I just okay (inaudible) just (inaudible) I heard that wrong, heard that clearly, repeat, repeat it to me. So there weren't issues during the whole call. So, so it was just when the, the paramedics(?) arrived that, like, "Okay, so, I am a doctor. I am just on time to help (inaudible)." So it's, there would have been, if there was no one, (inaudible) would make me, you know, feel some form of reluctancy to make sure that (inaudible) and maybe I just keep doing my compressing and checking my breath till the ambulance arrive (inaudible). Yeah.

00:27:12

I: Okay. So you mentioned that you did chest compressions when performing CPR. Did you do them on their own or did you do them with ventilations and rescue breaths? I think you said that you were trying to do it with rescue breaths but you were finding it difficult. If you could just clarify whether you did chest compressions only or whether you did these with breaths.

R: Yeah, I, and for me I don't already try chest compression for the process (?). So it just, when the defibrillator arrives, that, when, it, it, it was more, like, a, a shock. So it, it has some (inaudible) some pad. It, you just put it and it, like, just shock to the, to, to the heart's pumping. (noise) (inaudible) and you just have to bounce off. And I was, I was, "*What's* this?" Actually that was my first time seeing such. So the whole process, it was something, I, not me, not me doing that, not me doing that. So my dad asked me do that (inaudible) and he took part in his breaths, other side of the breath and yeah make use of that. But for me I only do just the chest compression.

I: So you only did chest compressions.

R: Yeah.

I: Okay. And what are your reflections on how that went? So what were your experiences of that?

R: Okay (pause) yeah, it's just, no one want to be, wouldn't really advise being in such a case. But a lot of things was going through me, a lot of change (?) was going through me. Like I said, I just have to say in my mind, "Please still alive. Please stay alive." So, but I heard some noises(?) I just think that, that (inaudible) was something that, you know, it has, it has given me that, that courage that, "I know it's my time yet (?). Something will happen (inaudible) it will. Staying alive."

I: Yeah.

R: "Stay alive." So …

I: Yeah.

R: … (inaudible).

I: Yeah.

R: (inaudible) I actually didn't say it out, but I just keep repeating this in my mind, in my head, just to have this, kind of, fate that something will just happen and, you know, he will (inaudible) breath. So, yeah.

I: Okay. And you said that the, before you said that the call handler was telling you to listen for breathing, for his breathing. Did they actually offer you or give you any instructions specifically on how to perform CPR?

R: I just, no, actually I just feel that this thing was given to me, you know, the way, like, this wasn’t (inaudible) really understood. So actually it just rush. Actually it just happened, it just, like, everything was happen in a rush time. So I don't really have, or they don’t really give me some precise way or, you know, break this, word it down for me to have (inaudible) or to really understand that. So some of it, I just have to, okay, just (inaudible 00:30:59).

I: Sorry, could you just repeat the last bit?

R: (Pause) Sorry?

I: Sorry, could you just repeat the last bit, the last bit of that, please?

R: Yeah, I, I, yeah, I just saying after everything just happened so, you know, it, it was just happening so suddenly that I even almost got confused with the process. 'Cause (inaudible) at the time and I was expected to do all of this you know and to go, go to see something I haven’t even done before.

I: Yeah, it was a lot for you, yeah. Overwhelming.

00:31:47

R: (Pause) Yes.

I: Yeah. Okay. And you, you used a defibrillator, didn't you? Is that what you said before you, that you used a defibrillator?

R: Actually, not me. My dad make use of that. So, yeah, but I can see that he doing that (inaudible).

I: Okay. So your dad used the defibrillator on the patient and you were witness to that.

R: Mmm hmm.

I: Okay. That's great. Thank you. So how easy do you think was it for your dad to use and understand?

R: Yeah, okay. I, actually, I, I, I thought it was something, you know, that you really need to be a professional to make use of. So I didn't actually know it might be, it not that now, that hard for … even some untrained, some untrained people can make use, can make use of that. So, because I do it, I saw the whole process, you know. So, so the, well, it makes some sound, so, and it just, more, like, it giving you feedback. I, is it, is it that giving you feedback what’d you say? You need some kind of (inaudible), you need to (inaudible) understand the, the language for, in terms of, you know, learning how to operate it. So, but I, I really (inaudible) the case, this sounds alright (inaudible) to my extent though, yeah.

I: Okay. So you felt that it was easy to follow the defibrillator voice instructions?

R: Mmm hmm.

I: Yeah. Okay. And, like, I know you, did you use it at all or was it just your dad using it?

R: It just, it just him using it. I, I didn’t actually use it at all.

I: And so what were your reasons for not you using it? 'Cause you performed the CPR. So then I'm just wondering why, why didn't you use the defibrillator then? What were your reasons for, for not using it?

R: Okay. I, I, I just feel that maybe … I'm, I'm not trained or it didn't actually came with a manual. So it's a different thing maybe … let's just imagine my, my dad wasn't present and he just came and I'm the only one that, I'm the one that need to make it (inaudible). So it would be a lot more helpful if it arrived with some sort of manual, something you can just get through in, in just a, a minute or two, that will give you some idea what you, what you're holding or what you're about to do. Because there might be some people that might, that might do CPR but haven't, didn't actually want to make use of the, the defibrillator 'cause they feel like, "*I* haven't done this. I even found it difficult for me to perform the CPR, and now, and now you're telling me to, you know, make use of the defibrillator. How is that possible? No, I can't. So I'll just keep doing the chest compression to the ambulance service arrive." So he just keep doing the pumping (?) and checking his breath, if there's, if there's some kind of hope. And you just keep hoping that maybe some, some kind of miracle will happen before the ambulance staffs arrive. So that's why I say it's only be good enough if maybe the defibrillator, defibrillator will arrive with some kind of manuals that will make you (inaudible) if you’re unsure about what you're holding or what you're about to do and … even if it's, even if someone have to call you to tell you. But you just need something to see that's arrived together with the drone, just to have this kind of idea of what you're about to do or what you're holding. So that will give you some information, although you have some limited time, but you just have to run through it the way (inaudible) or, or as fast as you can just to run through it before proceeding. So that's how (inaudible) I feel.

00:36:09

I: Okay. Okay. Did the call handler, though, offer any instructions to your dad over the phone about how to use a defibrillator?

R: Actually, they didn't actually give him some sort of instruction. 'Cause I, I (inaudible) that he, he might be familiar with that. But yeah, that was his own case, not, not the case of others. So the, another person might not be.

I: So he felt confident using it because he's a healthcare professional.

R: Yes.

I: Yeah. Okay. How did you feel, though, like, listening to the call handler and the voice instructions from the defibrillator at the same time? Did you find that confusing or were you, or not, or … do you see what I mean? 'Cause the defibrillator would have given instructions. And if the call handler is talking to you and your dad at the same time, how did you find that?

R: I just think everything just need to happen. We shouldn't just be in a rush. So that alone might make you be, you know, in a high jump (?) or make your mind to be unrest. So can you just, can one, can just something happen one after the other, not at a go? We are trying to, you know, achieve everything once. Let's just do one before the next one have to come through, please. So there shouldn't be any rush.

I: Okay.

R: The person speaking, the call handler is speaking, but I, I'm trying to treat him and make, you know, (inaudible). Me, I'm trying to look for what to do and I'm hearing different voices. The person, the, the emergency service on the phone is saying something, and the, the, my call handler is trying to (inaudible) at the same time. (louder) "Are you kidding me? Can someone just stop and say something once so we can know what we're doing?"

I: Right.

R: Not because the person on the face of death and you are just too eager to do something that you become a lot more confused.

I: Right. Okay. So you …

R: So yeah, that should be one. Okay, okay, just, okay, you're on the phone. Just pause. Just hold on. Someone is just here making (inaudible). But everybody just wants everything to happen on the rush. Everything should happen on the go for the person to be saved. Yeah, that's fine, that's good, everyone wants to save the person, but that shouldn't mean you should use all this, kind of, loudness or … even the people, the people who are just standby to trying to poke him, to see what's happening, what you're doing, they were surprised and that (inaudible) (louder) "Please, you guys, just shift back a little bit. Let’s do this." So it shouldn't just happen all on the go, I, I just don’t know but (inaudible), just for some minutes I, or for some second I wanted to feel frustrated. But I just have to pull myself up, just to, you know, let, let things happen well.

I: Yeah, you found …

R: I don’t really understand how I feel.

I: Yes. Yeah. You found it all chaotic, which is understandable given the situation. So the defibrillator that your dad used, was it already there at the scene or had someone else brought it over to you and your dad?

R: (Pause) It had been brought over.

00:39:51

I: Okay. So who brought it over to you?

R: (Pause) The drone.

I: Sorry?

R: There will be a drone.

I: Sorry, I didn't catch that. Was it someone in the crowd or …

R: I just seen someone around. But I didn’t actually just picture of that but I, I think someone, someone around. I, I, I, I can't just picture that.

I: You can't quite remember. That's fine. And was the fact that there was a defibrillator nearby mentioned by the call handler? Were you told? Did the call handler tell you that there's a defibrillator nearby?

R: Actually, I wasn't told that. Even if I (inaudible) I, I don't know. I don't know. (inaudible). I, I, I, I wasn’t told actually.

I: Okay. And what are your thoughts about using a defibrillator in the future?

R: Okay. There's some things I'll first need to see in place before making use of that. So you wouldn't just expect me to make use of some that I haven’t done such or I haven’t, my whole life, and, and mostly in a life-threatening case. So yes, let's make, make use of that. I want. So I just need a case whereby somebody to direct me or this kind of direction I'm talking about, someone is willing to explain to me what's going on, how this works, or how make use of it, or if the place where it is, maybe there will be some kind of logbook, no, not logbook, some manual where directive of usage will be listed in that. And everything in(?) that manual alone will be a lot more confidence to make use of that right. So, but apart from that manual and (inaudible), you just, you just need to, you know, you need some, kind of, mind or some, put yourself together before you be able to do such.

I: And is there anything else that would make it easier for you to use a defibrillator in the future?

R: (no audible response)

I: Sorry you've gone on mute.

R: (no audible response)

I: Hello?

R: It's okay and you can hear now?

I: Yes, yes, I can. Sorry, I was just wondering if there was anything else that would make it easier for you to use a defibrillator in the future.

R: Okay. I'm just, I, I, I think, okay, I'm just thinking, I'm just thinking, I'm just thinking. I can't really just get a hold of something here and now. But if I think of anything that I will let you know, I'll communicate with you via email.

I: Yeah, sure, absolutely. Thank you. So I know we're running a little bit over at the moment. Are you okay to continue with the last part of the interview?

R: Yeah, we can just proceed and do another minute (?), five minutes.

00:43:36

I: Okay, yeah. So just looking at your experience or your attitudes around drone-delivered defibrillators, so is this something that you've heard of, defibrillators being delivered by drone?

R: Yes, I've heard about this. But I haven't really exactly seen how, you know, how that is done, how, a lot of processes, or how, you know, be able to use that. So …

I: Yeah, that's fine. It's new technology, so that's understandable. Have you heard of drones, then?

R: Yeah, I've, I've seen that in movies, so I haven’t really used one.

I: Yeah, you haven't used one yourself but you've seen it in the film, on the TV.

R: Yeah.

I: How do you imagine, then, that drone-delivered defibrillation works? Do you have any ideas about how it might work?

R: I don't really, yeah, yeah, I, but I just know it might be faster compared to if it were delivered by, you know, some vehicle or, or some sort of transportation, because it can just, it, it can't be, you know, some road traffic or, or delay or so. But, you know, if you are, if you are delivery by, delivering it to a vehicle, there might be some road traffic or some congestion in the road, and that might slow the movement. But it's not the same compared to drones. So there's no traffic in the air, so it's just a full way (?) that it's just delivered to you at the scene. But it, it depends the distance. Because the most I know, that wouldn't travel, it can't really … I don't really know the distance or the kilometres which it can travel. But if it's something within the, the capacity of delivering to a drone, it would be a lot more, you know, a lot more, a lot more good to deliver to drone.

I: Yeah.

R: Because I think, I know it, it won't, it won't, it won't have any time factor in terms of delay to arrive or so.

I: Yeah. So you think it would be quicker than if it was being delivered by road or on the ground. And so, like, the, the drone, it can detach the defibrillator. So it can hover just above the ground and the defibrillator can detach itself from the drone. Or for some drones, they can lower the defibrillator down by a winch or a rope. Can you imagine yourself interacting with, with a drone in this way?

R: For me, I wouldn't love to go through the process of using some rope to detach. So, so is, is *there* not just a way that this can be delivered to you and, okay, so I don't know how it would be (inaudible), but it just have to land without you having to go through the process of, you know, holding it or so, or using the rope to drag it down or something of such. So I just need it to, just control it at your end and let it just land on the floor, so the only thing I have to do is just for me to go detach it from the drone without me having to, you know, like you say, throw some rope to drag it down or have to go and hold it on the air. Like, some people might not be a lot more confident to that extent, you know. Some people won't want to hold it. Yeah. But sometimes, or you might say, "Okay, it's around your, it's within your area and it's within your reach, so just put your hand up and hold it," while it's still (inaudible). So, "No, I can't do that. I'm scared." So can it just be pulled down at your end? So can't it land on the floor like a helicopter land? So it lands and the fan stops rotating in motion from the fan, so I'm able to go seize. And I just have to go closer and pull it off. That might be the best method, yeah.

I: And how would you feel about a drone bringing a defibrillator to you whilst helping someone having a cardiac arrest?

00:48:09

R: Okay, it's, I won't say it's something bad, but (inaudible) you know, if you really want to save that life, so you will really need the stuff to aid you. So it's something … it's not bad though, but (inaudible) put up a lot of, a lot of mind to do that. And if you're ready for that, okay, yeah, can give it a go.

I: You'd give it a go, yeah. Do you think it would make it easier or more difficult for you to use a defibrillator if one was delivered to you by drone?

R: I think it would be easier.

I: How so?

R: (no audible response)

I: Why do you think that?

R: Well, from what I see about the whole process, I, I just, I just, I just think it's (inaudible) making these events and seeing the drone process. So the other thing, you just need to be, you just need to say, "Are you ready for that? Can you do it? Do you, do you really need to, do you really want to give it a try?" So, "If you really wanna give it a try, so, then sure, go for it. You don't have to slide back." That what I have to say on that.

I: And how would you feel if you left the person, if they're by themselves, to get the defibrillator from the drone?

R: I wouldn't want to leave the person, or I wouldn't leave the person to be out of my sight. 'Cause what if the moment I leave, this person dies or something? So I can't really make promises (?). So maybe if someone in standby might help me if it's a place that, you know, there's someone there. Or if (inaudible) helps me with the, calling my phone, putting on speaker, might help me collect, retrieve the drone. Or just, like, just imagine I'm, I'm alone. So there's no one around. There's no one to help me. I just think it should be, just please bring it closer, a lot more closer to me, so even if I have to retrieve it, I won't be out of sight of the person which I'm trying to, you know, put him back to life. So that's how I want it to be.

I: How far, how close would you want the defibrillator to be delivered then, if you were by yourself with the person?

R: A lot more close, that, even if I have to retrieve it, I won't have to lose sight of the person.

I: Yeah. So, so would you be willing to go to your doorstep, if it had happened in the house, to get the defibrillator, for example? Or would you go over the road or down a few streets? How far would you go to get the defibrillator, if at all?

R: In the house, so please just bypass my, my gate, pass through my gate and let be to my doorstep if possible, so immediately I open the door I have to pick, pick it up. So I wouldn't want to go to the stress of going outside the street, losing completely sight of the person which I'm trying to, you know, get some, some breath back to.

00:51:40

I: Yeah.

R: Or, or it's in a, if it's, if it's in the street, I needed to be delivered there to the street. So not far away. Not, not the neighbouring street also. Just waiting right there. So, yeah, that's how I want it to be.

I: And what about if it was in a public place? Would your thinking on this change, how far you would want to go to get the defibrillator? Or would you want it as close as possible?

R: In the public space, so definitely there will be someone around there to help me retrieve it, even if it's not a lot more closer to me. So someone close by should do that, even in a public space with people.

I: Okay. And what do you think the role of the emergency call handler is in all this?

R: Sorry?

I: What do you think the role of the call handler is in all this? How do you think they might be able to help with getting the drone, the defibrillator to you? What information would you want them to tell you about the drone-delivered defibrillator?

R: Okay, they just have to tell me about how, how to really make heal, that if, if, if it's the person that's using it for the first time, so how can I heal that? How can I, apart from the heal(?) itself, what's the advantage of this? Like, what's really, what's, what's really the advantage? What's really the helpfulness of this? So, okay, is my CPR (inaudible) or is my compression, my hand compression not good enough also? Or is there something extra that the defibrillator can do that I couldn't do with my hand? So I just need all those, so all of those things will now give me the thoughts that, yeah, this is something good to make use of, so I can be happy.

I: Would you want to know that the drone was coming, or only when it was there, or information on identifying it?

R: (inaudible) where it is, the position, how many kilometres it's far away from me, so I'll be a lot more prepared. "Okay, okay, it's closer, so just please hold on, please stay alive. Just hold on a little bit. It's closer." So, you know, all of these words, you know, that you might say in your mind might helps you a lot. So I, I don't know, but some confidence just arose from you after seeing all of this, where that man is closer to you and you feel like the person is not really responding. So that, that hope alone that the drone is coming or the defibrillator is close to you will give you this, kind of, courage to just keep doing the compression, chest, the, and the rest of those.

I: Okay. And what concerns or problems do you foresee with drone-delivered defibrillators, if anything?

R: Huh?

I: Do you see any problems …

R: Ah.

I: … or issues or do you have any concerns about this technology?

00:54:47

R: I, I, I can't really say so, so deeply on that, but in terms of drone delivery, I don't know the distance which is covered. I don't really know how reliable it is. I don't really know, but I think it's battery usage. So I don't really know the battery failure or the life of the battery, like, the capacity of the battery and how far it can travel. If in the wind for a certain time it lost signal, and if it got totally lost (inaudible). So I don’t really know. I don't really know also if it works in weather conditions. So the climatic change so might result in how fast or how slow it will be or if it won't deliver at all, or … also I don't know if it mixes up connection. So what if my site have poor connection and just very close to it, it happened to fail or it lost signal because of connection? So I don't really know how that is. But all these are things that should be considered. And also public acceptance. I don't know if people accept this. I don't really know if people might see this as something strange or something not welcoming. So this (inaudible) that need to be made known to the, to the public about how reliable this is, so if, if you see it you won't be scared. "What's *this*?" Or, "What's, what's really coming close to me?" Or if you hear the sound of it approaching, you won't have to run away or take cover because you're scared of something or so. So it's only someone that know a lot of things about these drone that, even after seeing it, go, "Okay, okay, that drone is trying to deliver something." So you'll be rest assured because that's (inaudible). So, so I just think the public need to be aware of this device called drone, so, so even if they see that coming, no one will be, will feel unsafe or feel scared about what's going in the air, what this person is trying to do (inaudible). So, yeah, all of this information must be put into consideration so, yeah, it's fine, it's good to go.

I: Excellent. Thank you. Really interesting. So I just have some final, brief questions that may be useful for, for our research, if that's okay. So if I may ask, and I think you mentioned your age at the start of this interview, but if you could just say it again, please, for the purpose of the tape, what is your age and gender?

R: (no audible response)

I: Sorry, you're on mute. Did you hear me?

R: (no audible response)

I: Would you like me to …

R: Oh, sorry.

I: … repeat? Hi.

R: (inaudible) can you repeat yourself again?

I: Yeah. So if I may ask, what is your age and gender, please?

R: 28-year-old male.

I: Did you say 38?

R: 28.

I: 28. Okay. Male. Excellent. Thank you. And have you had training in CPR and defibrillator use?

R: I haven't really had training, but I just, I was a bit told about it. But I haven't had training that's made me confident to make use of that. I haven't.

I: No, you haven't. Okay, nothing. No. Okay. Is there something else you would like to add to your answers today?

R: Not really. Not in my mind.

I: Okay. I'm going to turn off the audio recorders now and go through what will happen next.

**END OF INTERVIEW.**

Participant 9

I:  Okay. So, the main interview audio recording has begun, and it will continue until the interview questions end. So, to start, can you tell me as much as you can about when you provided assistance to the person who had a cardiac arrest?

R: I provided assistance to a cousin of mine, who visited me in the workplace. When he visited me, and the cardiac arrest situation occurred, I had to run around for help, and see for (?) the defibrillator, to use in assistance my family member.

I: Right. So, did you say this happened at work?

R: Yeah, my family member came to my ...

I: Oh, yeah. Carry on. Sorry (Pause) You were saying your family member came to your place of work, is that correct?

R: Correct.

00:01:25

I: Okay. And your family member collapsed at your place of work?

R: Yes, yes.

I: Okay. So, did you realise what was happening to them at first?

R: Yes, I did realise, because I was aware of the situation at first.

I: Okay. And how did you realise it was a cardiac arrest?

R: I mean, I've heard some stories. I, I've heard about the occurrence of a cardiac arrest, and how, how it could be, how it could sometimes look like a convulsion, or some sort of ... So, I realised that it was one of those incidents.

I: Okay. And …

R: Some other people, my co-workers, confirmed.

I: Okay. So, how soon afterwards did you realise it was a cardiac arrest?

R: After the confirmation I got from a co-worker that it was a cardiac arrest, I actually confirmed because I'm aware that my family member already has a cardiac arrest situation, and that has happened sometime like that, but I wasn't present at that time.

I: Okay. So, your family member had a previous cardiac arrest, to the one that you were involved in.

R: Exactly.

I: Okay. What was the outcome for your family member?

R: You mean after the cardiac arrest situation?

I: Yeah. Yeah. What, what happened to them? And how were they?

R: After the cardiac ... After the cardiac arrest situation, my family member was okay at least. Not that (inaudible) okay, but stabilised.

I: But what, sorry? But ...

R: After that situation, my family member became okay, but not (inaudible) At least stabilised.

I: Oh, they, they were stabilised. Is that what you mean?

R: Yes.

I: Yeah. Okay. And presumably they went, they were taken to hospital where they received care?

R: Yes, my family member was sent to hospital.

I: Okay. And did you perform CPR?

R: Yes, I did perform CPR.

I: Okay. And we'll go over those experiences in a moment, if, if you don't mind. But for now, did you have immediate access to a defibrillator?

R: No.

I: Okay. And the, you say that there were other people with you at the time?

R: Three, three of my co-workers were close by.

I: Okay. And how did that make you feel, that there were other people around?

00:04:57

R: Felt not alone. I felt there was assistance. I felt, you know, I felt like if anything goes wrong, at least there are other people here to witness what is happening. You know, to help me out, to bring in suggestions, opinions, ideas, physical support.

I: Yes. That, that was important to you.

R: Import (?)

I: Okay. And may I ask why that support was important for you?

R: (pause) What did you say?

I: Sorry?

R: What did you say?

I: I, I said, why was the support important for you?

R: It was important because in a cardiac arrest situation, if not properly handled, most times results to death. Or results to a more tragic situation. And so, how do I go about giving an account for what happened to my family member? So, I was happy that people were there, at least, to witness the, the situation, how we are handling it, and the, the outcome. So, it would have been a different thing entirely *if* I was left alone with no support, no witness. I probably explain a lot ... I probably have a lot to explain, and I would probably lack support, and not get immediate help or assistance. Or also, like, ideas, contributions from people.

I: Yes. Yeah. So, if you were on your own, sorry, you said that you'd have a lot to explain. A lot to explain to who? And what would you have had to explain?

R: To other family members. To the doctors. To the authorities.

I: And explain what happened to them, you mean?

R: Yes.

I: Okay. So, the three other co-workers who were with you at the time. What did they do?

R: They helped with calling the hospital.

I: They phoned, they made the call. (Pause) Is that correct, they made a call?

R: The person made the call. The other person that's (inaudible) ...

I: And ...

R: ... providing some, the other person providing some ventilation for my family member and (inaudible) but while we waiting we were just supporting each other while we're using the defibrillator.

I: Okay. So, they made the call. They offered you support. And also, they performed CPR alongside yourself. Is that correct?

R: Yes (?).

I: Okay. So, can you tell me as much as you can about that 999 call?

R: (pause) The 999 call, is a very, was faster response, responsive. It was the only support we had, you know, only contact with emergency service we had at that moment. And (inaudible) made it difficult to have to calm down(?), I expect the caller you know be(?) calm and provided clear, and accurate information during the call.

00:09:14

I: Sorry, can I say that I can hear some interference on the call, and you sound a bit muffled again. Sorry.

R: Can you hear me now?

I: Yes, yeah, it's a bit clearer now, thank you. So, one of your co-workers made the call. Did they use their mobile?

R: The telephone.

I: Telephone, okay. And did they put the telephone on speakerphone?

R: No.

I: No. Okay. So, they were having the call privately?

R: Yes.

I: And did you leave the patient, to ... Did you leave your family member, to listen in to what the co-worker was saying to the call handler, or did you stay with the patient the whole time?

R: It was an office, and there was no how I could have left the patient to ... There was no how everyone could have left the patient just for a call. I wanted to (inaudible), but my colleague assured me that I shouldn't worry. I should go ahead, that everything had been sorted out, so there was no way I could have left the patient all by herself.

I: Okay. So, presumably the person who was making the call, they left the patient's side? Or were they with the patient?

R: Yes.

I: They left their side?

R: The, the, the, the caller, who happens to be my colleague, left their side.

I: Okay. So, do you have any knowledge or recollection of what the call handler said to your colleague?

R: (Pause) Hello?

I: Hi, can you hear me? Can you hear me?

R: Hello?

I: Hi. Hello?

R: (quieter) Can you hear me?

I: Yes, I can hear you. You're still a bit quiet though. Can you hear me?

R: (inaudible)

I: It's gone ...

R: What was (?) the question, please?

I: Sorry. I was just wondering if you have any recollection or knowledge of what the call handler said?

R: Yeah. I, my colleague said that the call handler said we should perform a chest compression, at a rate of 100 to 120 compressions per minute, until the services, the emergency service arrives.

I: Okay. Did they give any other help or advice?

00:12:21

R: They also said we should not stop performing this CPR until they arrive, and take over.

I: Do you know what questions the call handler asked your colleague?

R: If (inaudible) been provided, if CPR had been provided. And also asked if this is a long term issue, or how it happened. And also asked if we've confirmed that the situation is a cardiac arrest situation.

I: And did you or your colleague ask the call handler any questions?

R: I don't know if my colleague asked, but I was not present.

I: Okay. So, you don't know if any questions were asked. Okay.

R: Also, I think I remember my colleague asked so, how long it would take for the services to arrive. And they said it might take a little or no time.

I: Okay. And do you have any thoughts on how the interaction went?

R: (Pause) So, so, there were call, call, connection issues.

I: Okay.

R: I had to end the call, and my colleague kept on trying (inaudible) emergency, (inaudible) an emergency call (?) Tried that twice, and then the last time the call became smooth.

I: Okay. And did anything go well?

R: (pause) (quieter) Everything went well (?).

I: Sorry, I didn't catch that.

R: (quieter) Everything went well.

I: Okay. You've gone very quiet again, but I think I heard you say that everything went well. Okay. So, you said earlier that you performed CPR alongside your colleague. So, can you tell me as much as you can about your experience of this?

R: I am not really an experienced person doing CPR, because I don't frequently be around people with cardiac arrest, yeah. But I was able to perform CPR there, and I wasn't exactly sure of what I was doing, but for what it's worth, I was doing it the right way because the explanation my colleague got from the call handler about the CPR instructions aligned with the exact thing I was doing.

I: So, the, your colleague was relaying the instructions to you from the call handler. Is that correct?

R: I already knew how to, how to provide CPR, but for some reasons, I don't know, I wasn't certain or sure if I was doing it the right way. So, when my colleague came with the instructions on how it was being done, I followed by that. And it was almost the exact thing I was doing previously.

I: And when your colleague came to you with the instructions, had the, had your colleague finished talking to the call handler, or were they still on the phone to them?

R: My colleague had finished the call with the call handler.

I: Okay. And then they relayed the instructions to you. But you already ...

R: (inaudible)

I: ... knew. Sorry?

R: Yes.

I: Yeah. Okay. So, did you do chest compressions only, or did you do these with ventilations and rescue breaths?

00:16:43

R: Yes.

I: Which one, sorry?

R: (inaudible) chest compressions only.

I: Okay. And you said earlier that your colleague did chest compressions with the rescue breaths, is that correct? Could you just go into a bit more detail about that?

R: Sorry, you said I said what?

I: You said earlier that your, one of your colleagues helped to perform CPR. That they performed, that they gave the rescue breaths. Is that correct?

R: I said I performed CPR.

I: Okay. So, your colleague didn't perform CPR as well?

R: My, my colleague was only trying to provide ventilation (inaudible) my family member while the other person went for a call, and I performed the CPR.

I: Okay. So, ventilation, so that's the, the breaths that they were performing on your family member? Or what do you mean by ventilations? Could you just explain what you mean by that?

R: At (inaudible) my family member kind of (inaudible) like this you know, so my colleague helped him, using something like a mini fan to fan my family member. The other person made the call, and I provided the CPR. Do you, do you get me now?

I: Yeah, I understand. They didn't provide the breaths, they were giving them some air. Yeah. I understand. Ventilation, sorry. Different ... I thought you meant ventilations in the sense of CPR, and giving rescue breaths, not ventilation providing air, and making the room well-ventilated. Apologies. But thank you for clarifying that. Okay. So, what are your reflections on how the CPR went?

R: It was, CPR was very essential at that moment, because it helped sustain, or help my family member breathing until the defibrillator arrived.

I: Okay. And can you tell me what instructions the call handler offered you? And I understand that your colleague was relaying this to you after they had spoken to the call handler. What did your colleague say to you about what the call handler had told them?

R: (Pause) Think you had already asked that. I told you about how the call handler had asked some questions about if the CPR was previously performed, or was currently ongoing. And then, if CPR had not been performed, that we should perform CPR. And then gave instructions on how to perform the CPR. And also said that that shouldn't be stopped until the emergency services arrives with (?) defibrillator.

I: Yes. Yeah. You did mention that, thank you. I just wondered if you could tell me *what* instructions they gave exactly on how to perform the CPR? So, what did they tell you to do? If you could just go into a bit more detail about that, if possible? So, how often to compress the chest, or for how long? Did they give you that, sort of, kind of, information?

R: They give instructions about how to do the chest compression, and also (inaudible) into, into the mouth of my family member.

00:20:35

I: Okay. But you chose not to do the breaths. That's correct, isn't it? You said ...

R: No, I did, I did the breaths.

I: Oh, you did the breaths as well. Okay. And what are your reflections on the instructions that were given to you?

R: (pause) So, my (?) instructions were very straightforward (?). I found it to be helpful, (inaudible) but I, I found it to, I found it to be helpful because it was communicated in a simple way, communicated in a more easy way. But the same instructions I was given, I mean, (inaudible) there are some people who, who (inaudible) if it's communicated in a more complicated way.

I: Okay. And did you use a defibrillator?

R: (pause) Yes.

I: Yes, you did. Okay. So, can you tell me as much as you can about using the defibrillator?

R: Using the defibrillator was very helpful. Because defibrillator is a machine that assists people in such an emergency situation. Personally, we made sure that my family member was lying on their back, you know, on a dry (?) surface. Then we tried to put the chest (inaudible) It was (inaudible) there for easy access. Then attached the electrical pads to my family member's chest. Then (pause) then we stood very clear at this time, to avoid touching my family member while the defibrillator analysed the, the heart beating.

I: Okay. So, how easy was it to use and understand?

R: It wasn't that easy to, it was easy to understand, but not that easy to apply. Application of a machine and easy ways (?) to understand the machine are two different things. You can easily understand something, but can hardly put it to use. What happened was that the ambulance had brought the defibrillator. The emergency service (inaudible) make (?) them go. (inaudible) stand there. And also, what also guided us were the instructions from the defibrillator.

I: And how easy was it to follow those instructions from the defibrillator?

R: It was, it was easy.

I: Okay. And did the call handler offer any instructions over the phone about how to use the defibrillator?

R: (pause) Yes, but we preferred ... Think it was, think there was connection issues that, that didn't let us, you know, quite understand what the call handler was talking about. We preferred the instructions from the, the, the defibrillator, coupled with the *little* ideas, and little experience of that of my colleague. That's how we managed to use the defibrillator.

I: Were there any points when, where you were listening to the call handler, or your colleague was, and listening to the defibrillator voice instructions? Were, was there any point where you were listening to both at the same time?

R: No.

I: No. Okay. So, you were either listening to what your colleague was telling you from the call handler, or you were listening to the voice instructions?

R: (inaudible)

00:24:45

I: Yeah. Okay. And was the defibrillator already there?

R: (pause) Where?

I: So, at your place of work, was it already there, on site?

R: No, it wasn't on site.

I: Okay. So, where was it? Can, do you remember? (pause) Do you remember?

R: Emergency services in hospital.

I: Okay. So, you used a defibrillator when the emergency, when the paramedics came?

R: (inaudible) tell us how.

I: Okay. Did the call handler mention to you where a defibrillator was?

R: Not at that point.

I: No. Okay. So, you didn't have a defibrillator up until the paramedics came, and they brought you the defibrillator. And then *you* used the defibrillator?

R: Yeah. Because firstly, the situation happened at the office. It was unexpected.

I: It was un- what, sorry?

R: Was unexpected.

I: Okay. So, what, what's that, sorry?

R: I mean, we couldn't have had a defibrillator on site, because, firstly, this incident happened at my office ...

I: Yeah.

R: ... so it was an unexpected incident. I mean, it just happened.

I: Yes.

R: But nobody goes around with the idea, or with the thought that a cardiac arrest, person would seize or go into problems, or would be, or would be needing an emergency instantly. Nobody goes around with that thought.

I: Yeah. Sure. Yeah, it was sudden and unexpected. Yeah. Okay. And did the paramedics perform the, use the defibrillator, or was it just you who used it?

R: (pause) The paramedics came, and we (inaudible) but we used the defibrillator.

I: Okay. So, what are your thoughts about using a defibrillator in the future?

R: (pause) I'll, I'll (?), I believe that I will be using a defibrillator in the future if I need it, if there is use for it. Can always recommend it for other people, because I have seen, and witnessed, and experienced a defibrillator saves people's lives in the event of sudden cardiac arrest. And delivering an electrical shock to the heart helps to restore the heart rhythm, which is very essential for someone with a cardiac arrest and…

I: Is there something that would make it easier for you to use a defibrillator in the future?

00:28:15

R: (pause) I will know some, if I'm aware of the importance of defib, defibrillators, like I am now, or how to use it, you know, it can help foster a culture of readiness, and preparedness in an emergency situation. Also, if it is being created, or maybe if it is done in a way that is easy to understand and gives it clear instructions and minimal steps that I could, I could use it. And also, if it is readily accessible, you know, not something that you need a defibrillator, and it comes at night. You need it in the afternoon, it comes at night. It is very discouraging.

I: You ...

R: Because to me on using it, can also make me use it in future (?).

I: So, do you think it would have been beneficial to have one on site then, if you say that you wouldn't wanna need, need it in, say, the afternoon and then get it at night? Do you mean, then, to have it on site, or, or what do you mean? How would that look like in practice?

R: It might be having it on site, but arriving, you know ...

I: So, being given the defibrillator as soon as you need it, or as soon as. Yeah. Is that what you mean? Being brought the defibrillator as quickly as possible?

R: Yes.

I: Okay. Thank you for your responses. I just ... Sorry, did you have something else to add?

R: No, nothing really.

I: No. Okay. So, I just wanted to move on to the next part of the interview, which is about drones delivering defibrillators to the scene of a cardiac arrest. So, is this something that you've heard of? (pause) You're on mute, so I can't hear you if you are talking.

R: Sorry. I was thinking. I didn't know (inaudible)

I: That's okay, no worries. Yeah. So, is drone delivered defibrillation something you've heard of?

R: Drone.

I: Sorry?

R: Your question again.

I: Yes, sure. So, have you heard of drones delivering defibrillators to the scene of a cardiac arrest?

R: Yes.

I: Yeah. Okay. And have you heard of drones?

R: Yes.

I: Yes. Okay. Have you seen drones used, or used one yourself?

R: No.

I: No. But you've just heard of them, generally speaking. Okay. So, you say that you've heard of drones delivering defibrillators in emergency situations. So, how would you imagine that this technology works? (pause) Do you have any ideas, or thoughts about how drones would deliver defibrillators?

R: Drone defibrillators fast enough, unlike the human services.

I: Okay. So, they deliver them, it would, the drone would deliver them quickly. And why do you think that? What is it about that technology that would enable it to deliver the defibrillator quickly, do you think?

00:32:48

R: I think it's a good technology, because sometimes humans are just humans. They have their own problems. They have their own roles/rules (?) to play for themselves. If, if other humans have an emergency situation, it means that defibrillators would never be delivered on time if they wanna pay attention to their own crucial problems. So, a drone would never have any excuse, if the technology is circulated in all (inaudible) areas.

I: Interesting, thank you. So, drones are small flying aircraft, they're piloted remotely. They have cameras on them, so they can navigate and see where to go. And as you've insinuated, they can carry defibrillators. And they can do this by landing on the ground, and then detaching the defibrillator. Or they can hover just above the ground, and winch the device down to the ground. Say, with a rope like object. So, I was just wondering if you could imagine interacting with the defibrillator in the ways that I've just described.

R: Yes, that would be nice.

I: Sorry?

R: Yes, that would be nice.

I: Which ... Do you have a preference, or you can't say, or ...? Just wondering your reflections on, on what I've said.

R: I mean, the drone detaching the defibrillator after it's arrived is a very nice thing, but ... I don't know, but I just feel like that would be a nice idea.

I: And what about safety? How would the defibrillator get safely to the ground? Do you think that if it detached, that that would be a safe way, or you can't really say?

R: If the drone would gently, gently lower the defibrillator to the ground, it would be safe. If it's used gently. Or also, the drone is operated by a remote pilot, or controlled autonomously, just to ensure the defibrillator safely ... That would also be a good idea.

I: And then, how would somebody get the defibrillator from the drone? So, if it's been winched down, or if it detaches itself, how, how would you imagine that they get the defibrillator from the drone?

R: If the drone delivers the defibrillator to the exact location where it's needed, when it gets detached and dropped to the ground, then the person can get their defibrillator.

I: Yeah. Okay. And how would you feel about a drone bringing a defibrillator to you whilst helping someone having a cardiac arrest?

R: (coughs) I'd feel okay. But for a person who has no idea about using a defibrillator, who is clueless, the instructions of the defibrillator alone is just not enough for such person. So, what happens to the drone delivering the defibrillator, the person doesn't know the next thing to do, the instruction in the defibrillator is just not enough. So, what happens? The drone can't talk, the drone can't, can't perform anything. So, I also think it is wise and essential for a caller to call the person after the drone must have delivered it, find out or ensure that there is no difficulties using the defibrillator, or the defibrillator is not faulty, or that the defibrillator is quite responding very well.

I: Okay. So, what role do you think the call handler has in all this, then? Would you say that they have quite an important role then, from what you've just said, or ...?

R: Calling to find out if the person has received the defibrillator. Also ensuring if this person can (inaudible) good use of the defibrillator. Also finding out if the defibrillator is in good condition, and if the person has responded to the activities from the defibrillator. And also, finding out if this person requires other services.

00:37:57

I: Yes. Yeah. Would you think the call handler should also give information on when a drone was coming, or if it was ... Or when it was there, or information on identifying the drone? As well as giving the support that you've just mentioned.

R: (inaudible) except for just mentioned.

I: Sorry, I didn't catch that.

R: I don't really know, except for the ones I've mentioned.

I: Yeah. Sure. And that is really interesting, what you've just said. Definitely food for thought. So, how would you feel about leaving the person if they were by themselves, to get the defibrillator from the drone?

R: (pause) Not advisable.

I: Not what, sorry? Not ...

R: If there is no other person there, it's not advisable for the person to leave.

I: Yeah. So, how close would you want the defibrillator to ... How close would you want the drone to deliver the defibrillator to you, then?

R: Quite close.

I: Okay. So, imagine if the cardiac arrest happened at your house, or the patient's house. Would you be prepared to leave the patient to get the defibrillator from, say, the front door? Or would you cross the road to get it? Or would you not do any of those things?

R: I could cross the road to get it.

I: Okay. So, you'd be willing to leave your house, cross the road, get it, and then come back. Do you think you'd go any further than that, or that would be your, your limit?

R: (inaudible)

I: No. So, over the road, that, that would be your limit. And do you think your thinking on this would change depending on whether you were outdoors, or indoors? So, do you think you'd be prepared to go further if you were in an outdoor location? Or do you think you just wouldn't leave the person at all, if you were outdoors?

R: Position might change, but I don't know how, I don’t know when.

I: You're not sure.

R: Yes.

I: Okay. And how would you manage to get the defibrillator from the drone, if other people were around? (pause) So, how would ...

R: I would send someone (inaudible) to go get it. I would still not leave the person.

I: So, you'd stay with the person, and you'd get the other person to get the drone, to get the defibrillator from the drone, is that correct?

R: Yes.

00:41:11

I: Okay. Thank you. Now, do you have any concerns about this technology?

R: (pause) No.

I: Do, can you foresee any problems, or issues with, with drones delivering defibrillators?

R: No.

I: No. Okay. And sorry, just one other question. Going back earlier, when you said that the ambulance crew came to take your family member to hospital. Did you have to leave your family member’s side to let the ambulance crew into the building?

R: No. The security did.

I: Security let them in.

R: Yes.

I: Yeah. Okay. So, so, you stayed with them until the, the ambulance crew arrived. Okay. I just wanted to check that. So, thank you very much. I just have some final brief questions that may be useful for our research. So, if I may ask, what is your age and gender?

R: I'm 26 years. Gender male.

I: 26 years, and you are male. Okay. And have you had training in CPR and defibrillator use?

R: No.

I: Okay. Thank you. So, is there something else you would like to add to your answers today?

R: No.

I: Okay. I'm just going to turn off the recording, and tell you what happens next.

END OF INTERVIEW

Participant 10

I:  Okay. So, the main interview audio recording has begun. And this recording will continue until the interview questions end.

R: Okay.

I: Right. So, can you tell me as much as you can about when you provided assistance to the person who had a cardiac arrest?

R: Yeah. So, in [date of incident], my husband who was about 34 at the time, quite young, had a cardiac arrest. So, what happened is I had got up early that morning with our daughter, who was not quite a year old. Saw my husband briefly, and, kind of, said, "Oh, you know, you go back to sleep, I’ll get up with her, and I’ll see you in, you know, a couple of hours.” It was [early morning]. And, you know, no signs that he ill or anything at that point. And then, about an hour later, [time] I heard a, a, sort of, thud, as if somebody had fallen over, or dropped something. And I kind of thought to myself, “Well that’s a bit odd.” Even though, you know, we were making noise, we live in a terrace, there’s families near us (inaudible) kind of upstairs (inaudible) and I found him laying on the floor. I didn’t realise he wasn’t breathing. I didn’t know what a cardiac arrest was at the time, but he just didn’t look right. He was, kind of, non responsive. So, grabbed phone (?), phoned 999. They walked me through CPR. So, I did that. And then the ambulance crew arrived (inaudible) maybe four or five minutes later. It was really quick. Followed by several ambulances and paramedics. They took over the CPR, used a defibrillator (inaudible) And I think (?) two weeks passed (inaudible) various tests. They never found a cause for it. But they (?) said (?) he (?) had (?) a cardiac arrest it wasn’t (?) caused by a heart attack (inaudible) five (inaudible) ICD now (inaudible) defibrillator just in case it happens again.

00:02:22

I: Okay. Thank you for sharing those experiences with me. Some of what you were saying I got, but some of, some of what you were saying, the connection dropped out ...

R: Okay.

I: ... and I didn't hear the sentences fully. Would it be possible to go somewhere where there's a better connection, do you think, or is that not possible at the moment?

R: Not at the moment. I’ve got a, kind of, last minute appointment that I need to go to, but I’m happy for you to (inaudible) ask me the thing that you only heard part of, or I can try and say it again.

I: Okay. Okay. So, you said that you heard a thump initially, like, like somebody falling ...

R: Yeah.

I: ... on the floor. Is that correct?

R: Yeah, yeah, that's right. Yes, no, I was downstairs ...

I: Yeah.

R: ... I'd left my husband in bed. And then I heard, about an hour after I got up a loud sort of thud or some, like somebody had fallen over. And (?) I found him on the floor. So, it turned out that was him falling over backwards where he'd had a cardiac arrest and collapsed. Yeah, fallen back and fell on the floor.

I: Okay. Okay. So, you said that you didn't realise what was happening at first, is that correct?

R: Yeah. So, I was like, "Oh, something's wrong. I definitely need an ambulance.” But I didn't realise that he wasn't breathing. He was doing, like, agonal breathing, so he was making, kind of, raspy sound. So, I didn't quite realise he wasn't breathing. But luckily, when I phoned 999, they could, kind of, tell that he was doing that, and they obviously sent somebody out very quickly. So they obviously, yeah, sort of, assessed it correctly. 'Cause, you know, I feel like some people might say, "Oh, yeah they’re sort of breathing.” And then it might not have been such a high priority.

I: Right. Okay. So, did you realise, so when did you realise it was a cardiac arrest then?

R: Not until hours later. So, they took him to hospital, did an angiogram, and various other tests. And then doctor, or a consultant came to see me, and said, "Right, so we've ruled out a heart attack.” I thought (?), “Oh, that's good”, but they thought it was a cardiac arrest. Well, I was like, “I don't know the difference.” And then they explained, you know, that his heart had stopped pumping (?). So, it was only at that point, a couple of hours later, that I really understood what had happened.

I: Right. Okay. And, sorry, did you realise he'd stopped breathing?

00:04:56

R: Not initially.

I: Yeah.

R: It was all such a blur.

I: Yes, yes, of course.

R: But I didn't quite (?) fully realise the extent of what happened ...

I: Yes.

R: ... until few hours later when I was in hospital, and they'd done their initial triage.

I: Yes, yes. You just thought, "Oh, I'd better call 999, 'cause something's not right.”

R: Yeah.

I: A gut feeling.

R: Yeah.

I: Yes. Yeah. Okay. That makes sense. Thank you for clarifying. And you say that he was taken to hospital, and he had an internal defibrillator fitted. Is that correct?

R: Yes.

I: Yeah. Okay. And he's doing okay now, he's managing and ...?

R: Yeah, yeah, he's fine.

I: Yeah. Okay. Excellent. Thank you. So, did you perform CPR? Cardiopulmonary resuscitation.

R: Yes, I did. Yeah, because the 999 operator told me to, and (inaudible) what to do. I have done first aid training (inaudible) teenager, but I’m in my 30s now, so that's quite a long time ago. So, yeah, but because they told me to do it, so I did. Rather than, kind of, knowing that that was what I needed to do.

I: Okay. I'll ask you a little bit more about your experiences of doing that in a moment.

R: Okay.

I: But for now, did you have immediate access to a defibrillator?

R: No, 'cause it was at home.

I: Yes.

R: And I remember the ambulance service asking if I knew where the nearest one was. But also ‘cause I had my child at home, I couldn’t have left the house any way.

I: Yes. Yes. Okay. So, to clarify, you were by yourself, your young child was with you, and your husband?

R: Yeah.

I: Yeah.

R: Yes.

I: Yes. So, it was you managing this situation before the ...

R: Yeah.

00:06:48

I: ... ambulance ... Yeah. Okay. And how did that make you feel?

R: Ah! I think during the event itself, it was, it was such a blur. I think I was just running on adrenaline. And that initial instinct to phone the ambulance. 'Cause yeah, you know, phoned the ambulance, they told me to start CPR. But you know, I did that for several minutes. They then said, "Right, it's time to go unlock the door, because somebody will be there soon.” So, I did that and then went upstairs to my husband, carried on the CPR. Then the paramedic arrived, they took over the CPR. And at that point, I then went to phone my parents to come and look after (inaudible) for my daughter. 'Cause they were, they were half an hour away. So, I phoned them. And I then went and woke up my neighbours, I woke up my neighbours to sit with our daughter, in case I had to go with my husband in the ambulance before my parents got back. So, I was, kind of, quite, I don't know, sort of, organised, so to speak. I think I just, focusing on helping my husband, making sure there's someone to look after my daughter, rather than quite (?). I, I don't think I had time to think about what was wrong, I was just (?) running on adrenaline, really.

I: Yes, yes, yeah, you were just doing what needed to be done in that moment.

R: Yeah.

I: Yeah, okay that makes sense. And how did you feel about leaving your husband's side to let the ambulance crew in, presumably ...

R: Oh, it was awful. I really didn't wanna leave him. I knew that I needed to, and even before the call handler told me to do it, I was going, “No, the door’s locked, the door’s locked. I need to go and, like, unlock the door, they won’t be able to get in.” It was really like, I was really conscious (inaudible) do it, but at the same time I really didn't wanna leave him. But yeah, but I had to because, you know, I needed to go let the, the ambulance crew in.

I: Yes, yes. So you ...

R: I ran downstairs, opened the door wide, turned the lights on, and then ran, ran back upstairs and carried on with CPR while I waited for (inaudible)

I: Yes, yes. So, you, you weren't comfortable with leaving his side, but you felt that you had to do it, in order to, to let, let them in.

R: Yeah. Yeah (inaudible)

I: Yeah.

R: Yeah, it was (inaudible)

I: And the call handler had instructed you to go downstairs, and open the door at that point?

R: Yeah, they told me when it was, sort of, the right time to do it. 'Cause I kept saying, "I need to do this.” And he said, "Don't worry now, just carry on CPR.” And then, they said, "Right, *now* go and see (?) to the door.” I suppose, and, yeah they knew, kind of, when the paramedic would be there, and just basically to make sure I'd done several minutes of CPR so that I can leave my husband for 30 seconds or whatever.

I: Yes. Did you clarify with the call handler whether it was okay to leave him, or did you just go as soon as they asked you? As soon as they told you to go.

R: (inaudible) they asked me to.

00:09:52

I: As soon as they asked you to. Yeah. Okay. Excellent. So, talking about the, the 999 call, can you tell me as much as you can about it. So, anything else that you'd like to, to add. So, you presumably made the call. Could you tell me if you used a landline or mobile?

R: Yeah, it was a mobile. My husband had his mobile in his hand. I think I’d left mine downstairs, this, my initial reaction was like, "Oh, that's strange, that's a strange noise.” But it wasn't like, "Oh, my god there's clearly an emergency.” Until I saw him. So, yeah my husband had his phone in his hand, or next to him. So, I, I grabbed that, and used that phone, 999, and put it on speaker.

I: Okay. So, you were downstairs at the time, you weren't upstairs, and you heard the, the thud, you were downstairs.

R: That's right, I was downstairs ...

I: Yeah.

R: ... and he was upstairs in our bedroom.

I: Yeah. So, you came upstairs, and you used his mobile, and you put it on speakerphone.

R: Yes.

I: Okay. And did you leave him to make the call, or were you with him, by his side?

R: No, I was with him.

I: Yes. Okay. So, you've already said that the call handler instructed you to leave him to open the door, to let the ambulance crew in. Can you remember anything else what the call handler said, or what help they offered you?

R: Yeah, they talked me through CPR. So, they told me where to place my hand, and they were like, you know, (inaudible) really push, push, push, push, push. (inaudible) the frequency that you need to do it. I remember being surprised just how quickly you have to do it as well the, kind of, speed of the, the compressions, how fast you have to do them.

I: Yes. Yeah. Okay. And what questions did the call handler ask you?

R: I mean, I’m sure they asked me if he was breathing. And I said, “Oh, like, not properly, he’s making a strange noise.” They said, “Oh, is that him I can hear on the phone?” And I said, "Yes, it's like a raspy noise.” And I can't remember what else they asked me. I don't think they asked me to, like, check, you know his pulse or anything like that. I think they, they realised straight away that he wasn’t breathing, he was having an arrest. Not say those words, but I think looking back, they obviously, kind of, got to grips with the situation very quickly.

I: Yes. I was, so, actually you read my mind. I was going to ask you about that. Because if you hadn't realised it was a cardiac arrest at the time, and hadn't realised that he wasn't breathing, how did they then know to tell you to perform CPR. I was, yeah, I was going to ask, ask you that. But, so, so, they didn't know at first that it was a cardiac arrest. Did that later become apparent during the call, when they heard his raspy breathing?

00:12:48

R: It felt like it was almost immediately. I can't remember what else they asked (inaudible) I do remember the bit about the breathing. Because, as I said to you, I, I hadn't appreciated it, or realised that he wasn't breathing, because he was making some breathing sounds. But I think the call handler must have realised straight away, because it felt very quick that they told me to do CPR. They didn't tell me to put him in the recovery position, they didn't ask me to do any of, kind of observations, or I think it was almost immediately, you know, “do CPR. So, what you need to do is (inaudible) your hands in the middle of the chest, and ...” And then they, they told me when I needed to do each individual compression.

I: Yes, sure. Okay, thank you for clarifying that. And did you ask the call handler any questions?

R: Yeah. Because it was during coronavirus, so they weren’t, they were telling people not to do rescue breaths, just to do the compressions. And I remember saying, "Oh, you know, do I need to do the breaths? Like, he's my husband, I don't care if he has COVID, you know. Like, will it help?” They says, "No, do the CPR.” 'Cause I remember from my first aid training before, that you would do so many chest compressions, and then so many breaths. So, I do remember asking about that.

I: Yes. Yes. And they still advised you not to do the rescue breaths.

R: Yeah.

I: Yeah. Okay.

R: Yeah.

I: And, and how did you feel about that advice? About not doing the rescue breaths?

R: I, sort of, felt like, "Oh, I hope I’m doing it right,” or, like, “I hope it's *enough*.”

I: Yes.

R: Like on one hand, I, I trusted them and I followed their advice. And, but on the other hand, I was, sort of, thinking, "Oh, but, like, surely I need to, like you know, (inaudible) I suppose that you, you would do it normally.” But at the same time, I just, I just did what I was told, essentially.

I: Yes, yes, sure. And what are your reflections on the interaction? So, what do you feel went well? Was there anything that was difficult? What's your overall feeling on how it went?

R: I think they did a *remarkable* job! I’ve never had to phone an ambulance before, or really any emergency services. But yeah, looking back, I mean that, you know, they told me exactly what to do. And even though I didn't say that he wasn't breathing, you know, I didn't say he'd had a cardiac arrest, they very quickly seemed to realise what had happened. So, although it was obviously difficult and traumatic for me, I think the call handler did amazingly. And, and, and they got it right, essentially, which made all the difference, you know, 'cause obviously time is of the essence.

I: Yes, sure. Sure. Was there any, were there any difficulties?

R: No, not that I remember.

I: Okay. Great. And were there any technical issues while you was talking to the call handler on the phone?

R: No, no. It was fine.

I: Okay. Excellent. Thank you. So, just to go back to when you said that you performed the CPR, can you tell me some more about this? Anything else that you can recall?

R: I just remember how exhausting it was, it was, not realising before how quickly I had to do it, and grateful that the call handler said, you know, something (?) like (?) push you know constantly to make sure I was doing it at the right rhythm. 'Cause I wouldn't have done it quite as quick. I didn't realise how tiring it would be. I was really out of breath, but, but, but, you know, it's, kind of, the least of my worries at the same time. But I remember looking back thinking, god, like, I never realised quite how physically demanding it is (inaudible) to somebody.

00:16:50

I: Yes. Yes. And you did chest compressions only.

R: Yes.

I: Yes.

R: Yes.

I: Yes. Okay. So, what are your reflections on how that went? You said it was exhausting, it was tiring. You hadn't expected to be that tiring. Was there, were there any other reflections you had on, on that?

R: No, only that I think although I’d done some first aid training many, many years before, you know, I don't really know what I was doing. But I feel that they talked me through the, the, sort of, process, and, you know, what to do so that it, sort of, didn't matter if I didn't know what to do. Because as long as I followed their instructions, they, you know, it almost worked just as well for somebody who either was or wasn't first aid trained, because they tell you exactly what to do.

I: Okay. And can you remember exactly what it was that they told you? You know, could you outline for me the process from start to finish, do you think?

R: Not entirely. I do remember they said, you know, “put one hand on top of the other.”

I: Yes.

R: “Put your hand in the centre of his chest, down hard.” They said something, you know, “push, push, push.” (inaudible) And then, I was so out of breath doing it, I wasn’t (inaudible) necessarily talking to them, or asking them questions. And they said, you know, “Are you're still with me? Are you still doing it?” And I said, "Yes, yes, I am, I (inaudible), I promise.” Or something like that.

I: Yeah.

R: And apart from me asking them, "Do I need to open the door?” And, "Do I need to do the rescue breaths?” I was, kind of, just focused on what I needed to do, and just, I think I was just fuelled by adrenaline I think.

I: I, I lost the last bit of your sentence there. Sorry, what did you say at the end?

R: I think, I said I was just focused on what I needed to do, I was just fuelled by adrenaline.

I: Yes. Yes. Yeah. You were, you were coasting on adrenaline. Yeah. I understand that, 'cause it was a very stressful situation.

R: Yeah.

I:  So, I understand that you didn't use a defibrillator, is that correct?

R: That's correct.

I: Yes. Okay. So, I think you've touched upon this already, but I'll, I'll ask again just in case you want to add anything further. But what are your thoughts on why a defibrillator was not used?

R: So, the, the paramedic obviously had one with them, and they did use it. I'd left the room by that point, because they'd taken over the CPR, and I went to phone my parents and then go to my neighbours to get some, kind of (?), practical help with my daughter.

I: Yes.

00:19:39

R: But because, you know, the nearest one was maybe half a mile away, it, you know, realistic even if you forgot about me having a child at home, it just wouldn't be feasible to walk or drive down the road and get it, and, you know. Whereas if it was by, like, a parade of shops, seeing it if someone was collapsed there, that it's available. But didn't have one obviously in my home. It happened at home, rather than in a public place. That it was more effective just to carry on CPR and wait for the ambulance.

I: Yes. Yeah. And did the call handler advise you of that as well?

R: Yeah, they did. They said, "Oh, do you know if there's a defibrillator nearby?” Oh, it’s about half a mile away. And they said, "Okay, don't worry. Just carry on with the CPR.”

I: Yes. Yeah. Did you subsequently realise that there maybe was a closer one? I’m not saying that there is, but I’m just wondering if there ...

R: No, the only one I thought of is the only one that I’ve seen.

I: Yes. Yeah. Okay. That's fine. So, what, and you, and the call handler, did they advise you, did they know that there was one half a mile away from your, from your location, or ...?

R: No, I don't think they did check. I think (inaudible) me (?), "Do you know if there's a defibrillator nearby.” And I’d have said something like, "Oh, it’s, y’know, it’s, like, half a mile away.”

I: Yeah.

R: And then, and then that was, that was that.

I: Yeah. So, they asked you where one ...

R: Yeah.

I: Yeah. Yeah. Okay. So, what are your thoughts about using a defibrillator in the future?

R: Yeah, I, I would do, if I was, you know, in that situation in a, in a public place, then yeah, I, I definitely would, I would use one.

I: Yeah. Why do you, why do you say that? Why would you use one?

R: Well, because I think now, you know, I know how essential it is. And yeah, having given somebody CPR, and then them having been defibrillated, then getting them back just shows how crucial they are.

I: Yes. Sure. And is there something that would make it *easier* for you to use a defibrillator in the future?

R: (Pause) Not necessarily. I think because, even though I haven't used one, I know that there's instructions or it tells you what to do. You know, you would usually phone the ambulance service, phone 999, and they give you, you know, an access code and tell you what to do. So, I feel like, you know, I know what to do, and I know that, you know, if you place the pads on somebody and they're not in cardiac arrest, or it, it require a shock, it deliver one, you almost can't do it wrong if you (?) follow the instructions. So, although I hope I’m never in that situation, I would use one without any concerns.

I: Yes. Okay. Excellent. Thank you. So, if it's okay with you, I'd like to just move on to the remaining part of the interview, which is looking at drone delivery of defibrillators. So, firstly, is this something that you've heard of?

R: No, no, not before this study.

00:23:07

I: No. Okay. Have you heard of drones? (Pause) Hello? Can you ...

R: Oh, hi, I’m still here.

I: Oh, sorry.

R: Oh (?), that’s alright.

I: Did you ...

R: Yeah, I think I have heard of drones.

I: You have heard of drones. Okay. So ...

R: Yeah.

I: ... what do you know about drones then?

R: So, you know, people typically maybe use them to take photo, or video aerially. I guess you can use them to deliver (?) images, (inaudible) you really (?) see. Or, you know, on a much larger scale, things like military operations, as a, you know, type of ... Well, not quite an aeroplane but vehicle ...

I: Yeah.

R: Yeah.

I: Okay.

R: But I'm aware that they would have the capacity to deliver a parcel, or an item, as well as being used for things like photos and videos as well.

I: Excellent. Okay. So, have you seen drones used, or used one yourself?

R: Yeah, I’ve seen them used for photography (audio distorts)

I: Sorry, you've seen it in the news did you say?

R: No, I’ve seen it. I work in PR, so I’ve worked with lots of business (?). Some of them will have a drone, and take aerial photos or video for the projects that I am asking them to cap- (audio cuts out)

I: Oh, okay. So, you've worked closely with others who have used drones.

R: Yes.

I: Yes. Yeah. Okay. And have you ever operated one yourself, or ...?

R: No, I haven't.

I: No. No. Just worked, yeah, worked with others. Oh, brilliant.

R: Yeah.

I: Excellent. Lovely. So, in that case, how do you imagine drone delivered defibrillation works? Do you have any ideas on, on that?

R: Not really, because, you know, when I’ve, the experience I’ve had is there is somebody who is operating it from, something that’s about the size of a mobile phone. A, sort of, hand held controller, and that they have to be able to see the drone at all times. For, kind of, safety. And I know that when I’ve used photographers before, they have to check that they're like permitted to fly there, that it's not in some, sort of, of, you know, no fly zone. If it was, say, near a military base, or an airport, or somewhere that had restrictions that they have to check that they're allowed to fly it there. So, it's quite different, to the idea of being able to, sort of, fly one remotely to, say, a (inaudible)

00:25:47

I: To a, to a what, sorry? To a ...

R: Like, a house. Like, to a building, is it, to deliver a ...

I: Yeah.

R: ... defibrillator to somebody, that's obviously quite different, so I don't know exactly how that would work.

I: Yes. Yes. 'Cause you're saying in your line of work, there's a, there are a lot of rules and regulations to make sure ...

R: Yeah.

I: ... yeah, so you're not quite sure how that would apply in a situation where the drone could, effectively, go anywhere, to somebody's house. Yeah.

R: Yeah, yeah, yeah. That's right.

I: Okay. Excellent. And I'll ask you about whether you have any concerns or issues with that, the technology in a moment. But, but, as you touched upon, drones are small flying aircraft that are piloted remotely, and they have camera capabilities so they can navigate and see where to go. And they can carry defibrillators by landing and detaching the device, or they can hover above the ground and winch the device down to the ground. So, can you imagine interacting with a, with a defibrillator in the ways that I’ve just described?

R: Yeah, I think I can, like you said, yeah, I can see how one could be used.

I: Yes. Yes. Do you have any preference on how to use it? On how to get the drone, get the defibrillator from the drone?

R: (inaudible) one where it could detach itself ...

I: Yeah.

R: ... without requiring somebody to, kind of, physically take the defibrillator off, I think that's one less barrier, really. Because, you know, although on one hand I said I was running on adrenaline, you know, having to do anything that might have been slightly fiddly, I, you know, I probably shaking. So, you know, the thought of, having to, kind of, detach defibrillator from a drone. I’m not saying I wouldn't be able to do it, but I think I might find it more difficult during that (inaudible audio distorts) when time’s of the essence as well.

I: Yes. Yes. Absolutely, yes. So, it would give you one less thing to think about, one less thing ...

R: Yeah.

I: Yeah.

R: Yeah.

I: Okay. And do you think that would be a, a safer way as well, for the defibrillator to get to the ground, or ... What, what do you think about that?

00:28:15

R: Yeah, I think, I, I don't know if I feel like there's a difference in, sort of, safety per se, but I think it, yeah, I think it just would give people one less thing to do, if it could deliver it, and detach the defibrillator. Just thinking about, you know, a time perspective. Yeah. You know, how able that person is in the moment to do the steps to detach, especially (audio cuts out inaudible) sort of, stressed, shaking (audio cuts)

I: Yes. Yeah, if you're stressed and shaking, then it can be a lot more difficult, you're saying, to detach the defibrillator from the drone than if the drone just detached itself ...

R: Yeah.

I: ... and you just picked it up from the ground, and took it to the patient.

R: Yes.

I: Okay. So, how would you feel about a drone bringing a defibrillator to you whilst helping someone having a cardiac arrest?

R: I think, in an ideal world, my preference would be for the paramedic to get there, sort of, just as quickly as the, the drone would. You know, if I could wave a magic wand. But I was really fortunate that it was quite (inaudible) we’re near a major hospital, an ambulance hub. So, it only took, I don't know exactly, maybe four or five minutes for the medic to, to get to us. So, I think, in my situation, I was fortunate that they got there quickly, and I can't imagine the drone would have been able to get there that much quicker. But at the same time, if you were more rural, or there was traffic (inaudible) could take a *lot* longer to get from the hospital, then I can see that it would be of benefit. I think really, whatever the quickest way, (inaudible) a defibrillator is the best way.

I: Yes, yes, yes. And why would you prefer the ambulance to use the defibrillator, rather than yourself?

R: I think there's probably two reasons. I think, one, I'd imagine it would add to the trauma of the person who's giving CPR. It's another thing for them to do. Whereas, you know, 'cause in my experience, the paramedic turned up, took over the chest compressions, unpack the defibrillator, and I think by that point the second crew had turned up, and (inaudible). But it meant I didn't have to witness it, or, or, kind of, do it myself. I think I'd have more confidence that the ambulance service, or whoever, the paramedic, would know, you know, where to put the pads, and be quicker at doing it. And I think as well, you know, when my husband regained consciousness, he couldn't see, I think because of the hypoxia, and he was in a lot of pain, and he was really distressed. They, I think gave him morphine or something like that, that they carry. And that eventually calmed him down enough. But they did talk about getting the air ambulance, so that he could be, like, anesthetised, like, put under, I think, and intubated. So (?) they didn’t think they could get him down the stairs and out of our house when he was agitated. So, I think it's not just the needing somebody to restart their heart, or that kind of immediate medical care that they need straight afterwards.

I: Yes. Yes. That is very, very interesting, thank you. Because it, like you say, if, if the bystander is shocking the patient's heart, they can get it, might be able to get it restarted. But like you say, they won't have all the other stuff they need to look after the patient after the heart has been restarted, like morphine, and, and things. And ...

R: Yeah.

00:32:33

I: And, and you think that that, that could create a difficult situation, another difficult situation for the bystander? Or how, how do you think the bystander might, might ...

R: Yeah, I think it would be more traumatic for the bystander. I think for me, you know, the priority is always going to be the patient. I now know from experience with a cardiac arrest every second really matters. (inaudible) CPR, and then use a defibrillator quickly as you possibly can. But I think the, kind of, gold standard, if you like, would be that a paramedic arrived within a few minutes to every patient which didn’t always happen. And that they, they do it, but I think drone could be really useful in situations, and maybe just buy a bit of time. It might be that the extra few minutes it takes somebody to do that means that then by the time the person regains consciousness, the ambulance will arrive then. And they've just been defibrillated. And then the crew take over.

I: Yes. Yes

R: You know, and it’s a, it's clearly not a *replacement*. Maybe, kind of, buy people time.

I: Yes, yes, yes. So, by the time that the patient has been defibrillated by the bystander, the paramedics are on scene, and they can deliver that aftercare that you've just mentioned.

R: Yes.

I: Yes, yes. Okay. So, do you think it would make it easier or more difficult for you to use a defibrillator if one was delivered to you by drone?

R: (Pause) I think it would be the same level as difficulty, as if ... It's not maybe easier than using a access defibrillator, just if one is delivered to you. I suppose there is one less barrier compared to finding one, if it's not immediately in front of you, and then getting the, the code, and entering that, and opening the, the door, or whatever. And, you know, get (?) the (?) defibrillator out. And I suppose if one is just effectively given to you, and dropped off at your doorstep, or something, and I think that would be easier. But I, I suppose it’s probably more useful in places (audio cuts outs) publicly accessible one. Does that make sense?

I: Yes. So, you're saying that if it happened in a public place where there's more likely to be defibrillators around, then you might not need to use it as much as if you're in a remote area, or if you're at home. Is that, is that what you ...

R: Yeah. Yeah. Yeah. Yeah so, I think it's, so, if it'd make it easier or harder to use drone delivered one versus a public access one. Because for me, the only difference is that you wouldn't have to, and, you know, get the code for the public one, or indeed find it, and, you know, put the code in open it, etcetera.

I: I see. Yes, sorry, because it was dropping out a little bit, the connection there, I see what you mean, yeah.

R: Oh, I have stopped driving now, but I’m in a car park.

I: Oh, okay. Okay, I see what you mean. So, when you're outside and you're getting a public access defibrillator, you're going to have to get the code, get the code from the call handler, put that in, get the defibrillator out. Whereas if a drone delivers one to, to your doorstep, you don't have to, to do all of that stuff before getting it. You can just get it from the drone ...

R: Yeah.

I: ... the drone. Yeah. I see what you mean. So, there, there could be, kind of a, there could be some time that's saved ...

00:35:57

R: Yes.

I: ... from the drone delivering the defibrillator to the, to the bystander.

R: Yeah.

I: Okay. So, that could be a potential benefit of this technology. Okay. Excellent, thank you. So, how would you feel about leaving the person, if you're by yourself with the patient?

R: I wouldn't want to, but I think if I was instructed by the call handler, and they explained that, "We're gonna dispatch a defibrillator to you. Drone will drop it on your doorstep. Right, you need to leave now (inaudible) and tell me when you've got it.” You know, I think I would, because I think you're, kind of, inclined in those situations to (audio distorted) do what you're told, essentially, and, kind of, follow the instructions that you're given. So, although I wouldn't want to, because I didn't want to leave my husband to open the door, I knew that I had to. And so, I did it when the, when the operator told me to. And (inaudible) did it ...

I: As quickly as you could. Yes. So, you would want this advice from the call handler, then? You'd want the call handler ...

R: Yeah, I think you would definitely need a set of instructions (audio distorted) really clear what's gonna happen (? audio distorted), what you need to do.

I: Yes. Yes. You wouldn't just want to be left to your own devices as to make the decision whether to leave the patient to get the defibrillator from the drone. You'd want to be told the exact moment when you need to go to get it.

R: Yes.

I: Yes.

R: Yes, (audio cuts out, inaudible)

I: Excellent. Thank you. That’s, that’s brilliant, really, really informative, really interesting. So, how far would you be willing to go to get the defibrillator that had just been delivered?

R: Oh, probably no further than my doorstep, or, you know, the parking area that (inaudible) up the house (?) don't think I'd feel comfortable leaving the patient any longer than that. You know, it's hard to say exactly. And of course, (inaudible) it depends on, you know, each individual person's house, or wherever you're delivering it to. But for me, there's, like, a parking area outside the house, there would be room for the drone to come down. But, you know, you might be somewhere where there isn't. So, it's hard to (inaudible). But really, you want it as (audio cuts out) to the doorstep, I think, as possible.

I: Okay.

R: Because (audio cuts out) away from, the longer you're, you stop the CPR for, then obviously there's, like, a ... You have to balance how quickly the person (inaudible) defibrillator, versus how long they have (?) to (?) (audio distorts) miss CPR for.

I: Yeah. Yeah.

R: And I don't know what that equation is, but yeah, kind of, balance the, the risks and benefits, I guess, of each.

I: Yes. Yes. And do you think that's why it would be very helpful for the call handler to say, "Right, now you can stop the CPR, just to go and get it.” Because ...

00:38:57

R: Yeah.

I: Yeah. Take ...

R: Yeah. ‘Cause I suppose they probably want you to do it so, you know, (inaudible) many minutes, or as many that is to restore some (inaudible) and, you know, get the heart pumping, at least temporarily, before they then say (?) it’s (?) safe or safe enough to leave the person for a *very* short period, 30 seconds, one minute, to open the door to the ambulance crew, or get the defibrillator. Whereas, I'd imagine they, you know, want you to do it for a certain length of time perhaps, or at least wait until it's definitely on your doorstep, it's been detached, it's (inaudible) that you're not wasting any time.

I: Yes. Absolutely. Yeah. And would location affect your thinking on how far you'd be willing to go to get the defibrillator from the drone?

R: I think as well it depends, on the day, how long it would take me to get the defibrillator, or, or really just how long it would take one to arrive, whether that is by drone or by ambulance. Because for me, knowing that on a good day it would only take them five minutes for an ambulance to get to me, the drone would need to deliver it really close by, because otherwise (audio cuts out) it quicker for a medic to me. But if there were a reason it was gonna take longer, then it's potentially better (audio cuts) walk for one minute, two minutes (audio cuts out) to, to get it.

I: Yes. Sorry, it just dropped out at the end there. You said one or two minutes, and then I didn't hear.

R: Yeah. I said, you know, I can see that it might be worth walking, or going one or two minutes away ...

I: Yes.

R: ... to get the defibrillator. But again, I’d be concerned about stopping CPR, and feel that it might be more beneficial to carry that on ...

I: Yes. Yes.

R: ... rather than stop that. Whereas, if it was say on your doorstep, and it arrived quicker than a paramedic (audio cuts out), then that would be more beneficial, 'cause you don't need to stop the CPR for very long, and it gets the, the help needed, the (audio cuts out) to the person more quickly.

I: Yes. Yes. What about if the drone delivered it to over the road? Would you be willing to get it, if you're in your house?

R: Yeah, I think if it's somewhere, perhaps in my line of sight, then I'd probably be happy to run and get it.

I: Yeah.

R: I suppose my only concern would be, sort of, wasting any time trying to find it. And also, it was so, like, exhausting doing CPR, if I’m young and of, like, average fitness, I’m not, for example, elderly, or in ill health, or something (inaudible) gonna be really hard (inaudible) like running up and down the stairs to open the door. But again, I think it's just, like, (inaudible) effective CPR versus getting the defibrillator quickly. Just, sort of, it's really hard to work out ...

I: Yeah.

00:42:18

R: ... how far I would go to get one.

I: Yeah.

R: But really, I think if it, I think if I could see it, even if I could see it was coming at the end of my road, then I think I'd be happy to run and get it, bit more than if I didn't know where it was, or I couldn't see it (inaudible) left my house.

I: Yes. Then you would, you wouldn't want to do that.

R: I think perhaps psychologically, if you're able to see your house, go and pick it up, you might feel, like, safer, and not leaving that person on their own as much.

I: Yes.

R: You see (?)

I: Yes. I see what you mean. Yeah. Okay. And what do you think the role of the call handler is in all this? You've said to give instructions about when to leave the patient’s side to get the device from the drone. Anything else that you can think of?

R: Explaining exactly how you (audio cuts out) 'cause I think even if it came with some certain (?) instructions, or something (?) in it, I think it's probably more reassuring to have human voice tell you what to do and so that you can (audio cuts out) any questions.

I: Okay. Okay.

R: You know, explain more about how, you know, if need be, how to open it, and how to prepare it and anything you need to do. Where to stick the pad, you know, what button to press. Just to make it really obvious to people ...

I: Yeah.

R: ... what to do.

I: Yes, yes. That makes sense. And what would you want to know from the call handler about drone delivered defibrillators?

R: I think probably just keep it very simple, that, you know, "We're gonna send you a defibrillator. It's gonna come via a drone. You know, I'll tell you when it's ready. Carry on CPR for now. It will be delivered to, say, your doorstep, or within so many metres of your house. And that we’ll tell you exactly where it is ...”

I: Yeah.

R: “... when to get it.” Almost like to, sort of, warn you that it's coming, but to be clear that you should carry on CPR until they tell you (inaudible)

I: Yes. Yes, that makes perfect sense. And would you want information on identifying the drone?

R: Yeah, I expect so. Although I’d have thought that most people, maybe it's a generational thing, you’d expect most people would know what a drone is. Or if there was one outside, and (audio cuts out) call handler said, "Can you see the drone?” You think, "Yes, that's the drone.” But maybe for everyone that's not obvious. I don't know.

I: Yes. Yeah. Because it's still a, a ...

R: (inaudible) some people might need a brief description.

00:44:54

I: Yes. Yes, if they're not too familiar with the technology.

R: (inaudible) my grandparents would know what a drone is or not, for example.

I: Yes. Yes. Okay. And just going back to what you said earlier about the rules and regulations around flying drones. What concerns, if any, do you have about this, or problems, or issues do you foresee, if anything?

R: I think (pause) I think, really, I would just trust that, you know, for this study that you would work out how to do it safely, and that it would comply with regulations. I think if I was in a moment of need, I don't think it would concern me.

I: Yeah.

R: I think (inaudible), you know, any risk of, kind of, technical issues, or, you know, should it, I don't know, crash into a tree, (inaudible) from where I’m sitting, you know, something like that. I think, in terms of the study, I would trust that is obviously covered.

I: Okay.

R: And if I needed one in the moment, I don't think it would really cross my mind.

I: No. No. So, you can't see any particular issues or, or problems with, with it.

R: No, I can't.

I: No. Okay. Excellent. Well, thank you for your insights today, they've been really interesting. So, I have some final brief questions that may be useful for our research. So, if I may ask, what is your age and gender?

R: Yeah, I’m female, and I’m 33.

I: Okay. And have you had training in CPR and defibrillator use?

R: I have in CPR when I was a teenager, but not, not a defibrillator.

I: No. You haven't had CPR training since you were a teenager, is that correct?

R: Yes, that's right. It was just part of, like, general first aid training.

I: Yes. And that training occurred before the incident being discussed.

R: Yes.

I: Yeah. Okay. Excellent. So, is there something else that you would like to add to your answers today?

R: No, just that it's not something I’d have ever considered. I never asked myself at any time, "Oh, could you use a drone to deliver a defibrillator?” You know, you hear that, “Oh, Amazon are gonna use drones,” or this that and the other.

I: Yes.

R: So, it makes sense that, you know, if the technology exists, you might be able to use it. But no, I, I think it's good that you're looking at it. I think whatever gives people a defibrillator quickest is, is the main thing, really. So, no, I think it's really interesting, and worth looking into.

I: Oh, excellent. Thank you. Thank you for that. Okay then, I’m just going to turn off the recorders, and then just go through what will happen next.

R:  Okay.

**END OF INTERVIEW**

Participant 11

I:  Okay. So, the main interview audio recording has begun ...

R: Yeah.

I: ... and it will continue until the interview questions end. Okay. So, to start then, can you tell me as much as you can about when you provided assistance to the person who had a cardiac arrest?

R: Right. So, the circumstances were, [date of incident], at [time] in the morning. We had, my husband had made the cup of tea that we have in bed in the morning. We’d just finished our cup of tea, and the local news starts at [time], that's why I know the time exactly. And all of a sudden, I was aware of this gagging noise. Turned to look at my husband, who was beside me in bed, and tried, there was no response. Tried shaking him, no response. And I straight away realised there was a major issue. We have a phone beside the bed. I immediately rang the 999 number, and got through to the emergency services, and explained what had happened. They then asked me to check to see if he was breathing, and I said I didn't, didn't think ... I was certain he wasn't at that point, there was definitely no response. And because he was in bed, they said I

00:01:50

had to get him out of bed onto the floor. Which is a bit, it was a major challenge, and to this day I really don't know how I did it. Because there's only about two foot at the side of the bed to get him off the bed onto the floor. But I did, I managed to get him onto the floor. And the whole time, the ambulance, the operator was talking to me. I put it onto a speaker, she was talking me through, and telling me what I had to do. And I managed to get him flat, and then ... I had done CPR training when I was at work, but, I mean, I'd retired in [year], so it was a long time ago. But obviously it's still there somewhere in your memory. And the operator said, right, what I had to do was put my hands on his chest, and do the CPR, and I had to count with her. And I had to say that aloud. And if I stopped, I was still counting, but if I stopped speaking, she would tell me I had to keep speaking. Because she said she would know I was then focused on what I was doing. That, and obviously they were dispatching an ambulance off to him, which ... So, I kept doing the CPR for about, I would say it was probably about 15 minutes. Ha what you have to bear in mind here, is that I wear hearing aids. And at that time in the morning I don't have them in. So, that was my (chuckles slightly)first issue. And our house, you come to the back door, and the doorbell is in our lounge which is at the front. So, we had a combination of various things, and I kept saying, you know, shouting, "When's the ambulance getting here?” And she said I had to stay with my husband doing the CPR the whole time until I heard the doorbell. Which, I had to then explain, "You will have to tell me when they're at the back door, because I will *not* hear it.” So, I do know it would be after quarter to nine that I answered the back door. They went straight upstairs. I was told to move into the other room, and they would just deal with my husband. And I know from my phone I rang my son just before ten to nine, that's how I know the timings. From that point, there, there was another ambulance driver, looked, well, a paramedic looked after *me*. And my son got here very quickly, fortunately. I gather they shocked my husband, I think twice at home, but I’m not sure about that. I wasn't allowed in the room at all. I wasn't allowed to be anywhere when they brought him downstairs, took him into the ambulance. The paramedic that was with me, he took me in a car to the hospital. And when we got to the hospital, obviously my husband had arrived there, and he was in the, the place that you arrive, when you ... [husband interjects to remind participant where he had been taken] the triage place. I couldn't see him. All I saw was the curtain being moved around him. And I was then put into a family room, which by then my son arrived, and obviously then my daughter and son in law arrived, and we were there. I don't think we got much news at that point for over an hour, 'cause obviously they were working on [husband’s Name]. He then got transferred up to the ICU, and we were there all day. We were, I was allowed to go in, but he wasn't conscious. He didn't, you know, he was, had tubes breathing. He was breathing but he wasn't conscious at all. He didn't know we're there. But it's [late] at night, the consultant got the family together, and we were told at that point that (pause) it, they'd done as much (sighs, breathes out) as they could. And the only thing we could hope for that night was a miracle and that's how it was left on that [day of week] night. So, I came home. Well, we all left, after ... We all went in to say goodnight to him, at that point thinking, "This is it. It’s, it’s over.” And then, the next morning my daughter who had stayed with me, she rang the hospital. And they said, "You'll have to ring back later after 10 o'clock.” Which we did. And the first new, thing they said to her was that my husband had opened his eyes and spoken (pause) so, we, she put the phone down, told me, and then we rang back again, because we actually thought they had the wrong person at that point, I'll be honest. *But* it did happen. We had a miracle. He was kept under sedation, that was from, that would be the [day of week]. They, like, because he's breathing, and his brain obviously had been starved of oxygen for a while, they kept him under sedation. And they started lifting it by [day of week +1] And by then, he did become slightly conscious, but very confused. Didn't always know where he was and what was happening. Fought terrible to get the tubes out of his mouth and his nose. By the [day of week +2], he was then a lot

00:08:55

more conscious. They'd managed to get him out of the chair, out of the bed, onto a chair. And they managed to get him to walk around just in a circle. And then, on the [day of week +3], they moved him from the ICU down to the ward. Which he was definitely more with it, but not 100%. [Husband’s Name] doesn't remember any of this, or vague memories. He was on that ward until the following [day of week, following week] ... Yeah. Where they transferred him to the [Name of hospital], which is our major heart hospital [references region] here. And he's had a, an internal defibrillator fitted. And since then, he was discharged, I think the next day after getting the defibrillator fitted, and just told to take life quietly, and easy. And, you know, he was very tired, very confused. He didn't know, we had to keep telling him what had happened. And then, of course COVID hit. So, we didn't have any further check ups for quite a while. But he has had, you know, he has regular check ups. His defibrillator ... So, this all happened in [month of incident]. (Chuckles slightly)in the [month], virtually the same time in the morning, in the same circumstances, but [husband’s Name] was aware that he didn't feel right when I heard the gagging noise. But by the time, and he jumped, and that was his defibrillator giving him a shock. Which the whole bed shocked, I got a shock as well. I, he then came back round, and I’m screaming at him because I was panicking. He thought something had happened to *me*, but we then realised what had happened. Rang the hospital. They then, 'cause we've got a monitor, they then told us what to do. And they said he’d basically had another cardiac arrest in the same circumstances. So, when we saw the consultant, the medication that he was taking, and I can't remember the name of it, he was only taking first thing in the morning. The consultant decided the best thing would be to split the dose, so he takes part of it in the morning, and now he takes part at night, to keep the balance(e in his body for the whole day and overnight. So, everything was going fine. We, he does cycle, and at that point he had just an ordinary bike, going out cycling. And that would be [year]. He did start cycling again, and walking, was doing quite well. And the year that he was 70, was it 70? Yes, or 75. [Husband clarifies how long ago this was] [number of years] ago. So, it was, when he was coming up to his 75^th^ birthday, he decided he was gonna cycle 75 kilometres. [Husband clarifies it was miles] Not miles, kilometres. [Husband clarifies again it was miles] And so, he then went out, he did the bike ride, he had his lunch, and then went out to do the second half of the bike ride. And when he was out, he thought he'd been hit by a bird. He came back, and he said, "I think my defibrillator might have gone off,” when he was going up a hill. And I said, "Well, it can't of, ‘cause we would get a phone call from the hospital.” Well, they had tried ringing us, and they tried ringing [husband’s Name] on his mobile, but he'd missed his, missed the phone call. And we, as it happened, we were going for his check up the week later. And the consultant, as soon as we got in, said, "*What* were you up to on such and such a time, on such and such a date?” And of course, he had to confess what had happened. So, at that point he was told that he had to moderate how he cycled. And from then, we got him an e-bike after that. And he's fine. He gets out of breath now, we've noticed that. But apparently that's one of the side effects that could happen. But in the main, the consultant is pleased with him. And as we say, at the time, it *was* a miracle.

00:14:34

I: Okay.

R: Literally. Yeah.

I: Thank you for sharing those experiences. Are you okay to continue with the interview? 'Cause I know that what you’ve told me ...

R: Yes ...

I: ... is difficult.

R: I’m okay.

I: If you want to pause, or ...

R: Yes. It, it does bring, it does, I don't realise how much it does affect me.

I: Of course, yeah.

R: But we went to a, a conference last year, down [Name of area] for survivors and people with defibrillators. And I have to say, that was the first time I'd probably talk more about it, 'cause they took the supporters to one side.

I: Yes.

R: And I was more upset then. I mean, I’m, you know, reliving it now, it's, it's bad, but it's not as bad as it was then.

I: Well, would you like to pause or stop the interview ...

R: No, no, that's fine.

I: ... 'cause you, you know,

R:  No, I’m fine.

I:  … you don't have to continue.

R: No, I’m quite happy to continue, thank you.

I: Okay. So, if I may ...

R: Yeah.

I: ... let's go back to the first incident in [date].

R: Yeah.

I: How did you, if you did at all, realise it was a cardiac arrest?

R: Well, I didn't know it was a cardiac arrest. I just thought it was a heart attack, right. He wasn't breathing, a heart attack, right. Because at that point, I didn't know there was a difference.

I: Yes. Okay. So, how soon afterwards, if at all, did you realise it was a cardiac arrest?

R: Possibly (pause) probably not until a long time after. It would have been explained at the time, the difference, but I’m fairly confident at that time I would *not* have taken it in. It would have just been he's had a heart attack in my mind, right. It would be when we probably had the first check up at the hospital afterwards, and it would have been explained again then. And that's probably when it registered with me, you know ...

I: Yes.

00:17:06

R: ... there’s a difference.

I: Yes. But not during the event itself.

R: Ah! No, definitely not.

I: No.

R: No.

I: Okay. Understandable. A lot was going on. So, did you have immediate access to a defibrillator?

R: No, no.

I: Okay. And were you by yourself with your husband?

R: Yes.

I: Yeah.

R: Yes.

I: So, how did that make you feel?

R: Very stressed. Very anxious. Determined to keep (chuckles)thumping him on his chest and counting. And the girl, the operator, she was brilliant. You know, she kept me updated, I do remember that she kept updating me, saying, "The ambulance isn't going to be long, the ambulance isn't going to be long.” 'Cause I think it felt like a lifetime, to be honest.

I: Yeah. Sure. Understandable.

R: Yeah.

I: And that would have added to the anxiety that you were already feeling.

R: Yeah.

I: So, how did you feel about leaving your, about leaving your husband to let the ambulance crew in?

R: I have *never* come down our staircase as *quick* in my *life*. And I mean, there I am, in my nightie. That's all I had on. Hair sticking up, you know, your night time hair (chuckles) no glasses, no hearing aids. I just *literally*, as soon as she said they're there, I got ... Well, I can't kneel, there wasn't enough room, so I manage, got myself up. As I say, I came down those stairs, I’ve never come down as quick in my life. Back door open, and literally as soon as I turned the key, they were in, and past me. They *literally* just came straight past me, and they, "Where is he?” “Upstairs on the right.” And they were there. The bed that I couldn't move got moved, the lamp got ... Went all over the place, the chest, the little chest of drawers (chuckles slightly)was tipped. They just moved everything to get in at him, you know. And obviously they then said, no, I had to, I couldn't be in there. And they said, "Go in the other ...” Well, the other bedroom, I went in, and I picked up my mobile phone to ring my son, you know.

I: Yeah. Okay.

R: And in panic mode, absolute panic mode.

I: Yeah. Yeah. Okay. That, that’s understandable, because of the chaos, and yeah ...

R: Yeah.

00:20:01

I: ... going around you. So, can you tell me as much as you can about the, the 999 call? And I know you've touched upon this already ...

R: Yeah.

I: ... but you made the call, presume?

R: Yes.

I: Yes, yes, you, yeah, 'cause you were on your own. Did you use a mobile, or landline, or ...?

R: No, we have a landline phone beside our bed. So, my mobile was in the bedroom, 'cause I ... Well, no, actually I don't know whether it was that day. 'Cause it's since then I always take my mobile upstairs now. So, it, I don't think my mobile was upstairs. So, it would be, it was the landline which is beside the bed. So, I’m in bed, my husband's beside me slumped, and I rang the 999. Obviously in a, quite a panicked voice, I would guess. I, I don't think I would have been shouting, but I would have been quite hyper at that point.

I: Did you put the phone on speakerphone?

R: Yes. Yes. I had to. Well, for two reasons. I couldn't hear with it just beside my ear. And also she kept asking me to do things, which was check to see if he had a pulse in his neck, and like this, and shake him, I had to shake him. No response whatsoever, you know.

I: Yes. Yes. And you stayed with your husband while you were making the call.

R: Oh, yes. Yes. I was, at that point, I was actually still in bed ...

I: Yes.

R: ... beside him. And ...

I: Okay. Sorry.

R: Yeah.

I: Yeah.

R: Go on. Sorry.

I: So, can you remember what the call handler said, and what help she or he offered. I think you've again touched upon this already, but did you want to add anything else about what they said or offered?

R: No, it was purely to establish if he was breathing.

I: Okay. Yes.

R: That was the, that was the main thing. Was he breathing? Was he responsive? That was, I do remember those were the questions that I was asked.

I: Okay. Any other questions, or those were the main ones?

R: Not that I can remember.

I: No, no. Sure.

00:22:25

R: Those are the two things that stick in my mind. And then, the next thing was, well, obviously she said, as I must have said, he's in bed, slumped, sitting up. And that is when I was told I had to get him onto the floor (pause) so, obviously at that point, I had to get out of the bed on my side, go round the bottom to get to where he was. Pulled the covers back, and then, as I say, I *really* don't know how I did it. Because he’s, he’s, he’s a bigger person than me. I, sort of, have a vague memory trying to pull his legs. I have a funny feeling I might have got back beside him, and pushed my feet against him a bit, to try (chuckles slightly) he's [husband] laughing, because when he did land, he actually landed with his head against the chest of drawers that was there (chuckles)and somewhere along the line later, he must have, I think he possibly might have broken his nose. And I, I do remember the consultant saying words to him like, "Well, if that's the only thing that's happened, that's not an issue.”

I: Yeah.

R: But, you know, he, I think I possibly damaged his back as well, the way he landed. And across ... Oh, well, I think I may have broken some ribs when I was doing the CPR. But again, the consultant said, "They are all, they don't matter.”

I: Yes. Yes.

R: Yeah.

I: Okay. And are you sure you're okay to continue? Yeah.

R: Yes.

I: Okay.

R: Fine, yeah.

I: Just let me know if you want to stop at any (inaudible).

R: Yeah, I will do. Yeah.

I: Okay. So, did you ask the call handler any questions?

R: I don't believe so, no. I think, I mean, apart from saying, "When's,” shouting, "When's the ambulance man getting here?” That is probably the only question I asked.

I: Yes. Yes. And what's your reflections on the interaction? Do you think it, what went well, was there any difficulties?

R: Well, I mean, I have to say, even bringing it back now, the memory of that call handler on the other end of the line was just amazing. I did try to find out who it was to say thank you, but I didn't manage, you know. She was absolutely brilliant. She, her voice was, her voice was very calm, and that's what she kept, you know, she just kept saying, "We're gonna do CPR. This is what you have to do.” Explained, and I had to count. One, and two, and three, and four. And I had to keep counting aloud, 'cause obviously, after a little while I would stop. I was still doing it, but stop counting aloud. And she said, "No, I need to hear that you're doing it.” But I think that's possibly to keep you focused, you know.

I: Yes. Yes.

R: That, retrospectively, that's what I think that was all about, you know.

I: Yes.

R: And obviously, when I kept saying, "When are they getting here?” She said, "They won't be long.” And then, we had that bit of interaction about she'll have to tell me. Apparently the neighbours heard them banging on the door, but I didn't. (Chuckles slightly)**.**

00:26:12

I: Yes. Yeah. And were there any technical issues while you were talking to the call handler on the phone?

R: What do you mean technical?

I: For example, did the connection drop out, or was there interference?

R: No, no, she was there the whole time.

I: Yeah.

R: Yeah.

I: Okay. So, no issues ...

R: Yeah.

I: ... with that. Okay.

R: Yeah.

I: So, you've said that you've performed CPR.

R: I did.

I: Yes. So, can you tell me as much as you can about that? Any other insights, or thoughts, or ...?

R: (Chuckles slightly)[husband reminds participant that she couldn’t kneel] I, I can't kneel, because I’ve got artificial knees, right. So, I am, I managed to make enough room at the side of the bed to have my feet on the floor, so I’m *leaning* forward. So, you've got, he's got the full weight of my body, not just from a kneeling position. I am *leaning*,heavy, well, obviously heavily on him. I had to manipulate him so that he was flat. As I say, he ended up, sort of, sideways. Manage, got him flat. Managed to get my feet as near as I could, enough room to then have my hands in the right position to do it. And obviously the girl, the phone was right beside me on the bed, and she was talking me through it, you know.

I: Yes. Yes.

R: And as I say, I had had training years ago, and obviously that came back. So, I did know where to do it, you know, and she, she probably did tell me, but I can't remember.

I: No, that's fine. Did you do chest compressions only, or did you do those with rescue breaths?

R: No, just chest compression.

I: Okay. And what are your reflections on how that went?

R: (Chuckles) well, after doing it on a dummy not so long ago at the support group, I don't know how I did it for so long. 'Cause it was a lot harder than I imagined it was. But I suppose at the time, you've got the adrenaline, and I’m just trying to save me husband's life, basically. That's what it was.

I: Yes. Yeah. So, you've gotta do what you need to do.

R: Yeah. And you just do it, you know. You just, I just kept doing it, because the girl on the other end kept saying, "Keep going. Keep going. Keep going.” Which I did, you know, until the ambulance men got there. And as I say, it would be 15 minutes probably ...

I: Yeah. Before they came, the ambulance came. Yes.

00:29:12

R: Yeah.

I: Okay. So, you've said a little bit about what instructions the call handler gave you.

R: Yeah.

I: She told you to count while you were doing the compressions.

R: Yeah.

I: Were, were there any other instructions she gave you about how to perform the CPR?

R: (Pause) I honestly can't remember.

I: No, that's absolutely fine.

R: Really can't.

I: Yeah.

R: The, as I say, the one thing that sticks in my mind is I had to keep counting aloud.

I: Yes. Yes. Sure. Yeah.

R: Yeah.

I: And what are your reflections on the instructions and the advice given to you by the call handler?

R: As I say, you know, straight away, you know, you know, once, once we knew [husband’s Name] was okay, and I’m not saying that week or anything ... But the call handler was amazing.

I: Yeah.

R: Just absolutely amazing. So, were the ambulance men, you know, but the person who was at the other end of the phone was amazing.

I: Okay. So, and you feel that she was amazing 'cause she was calm, but also her instructions were clear, or ...

R: Yes.

I: ... what do you think ...

R: Exactly.

I: Yeah.

R: She was calm, and giving, whatever instructions there were, were clear.

I: Yes. And that helped ...

R: Yes.

I: ... clearly.

R: Yes.

I: Yeah.

R: Yeah.

I: Okay. So, you didn't use a defibrillator, is that ...?

R: No.

00:30:43

I: No.

R: No.

I: Okay. So, what are your thoughts on why a defibrillator was not used until the paramedics arrived?

R: Well, one, because we didn't have one.

I: Yes.

R: Right.

I: Yes. Yes.

R: And at that point, you know, you hear of defibrillators, but you think, "Oh, I don't even, you know, I’m not aware what they are,” or ... To be honest, you, you vaguely know, but you're not, you don't know enough about them, you know. Since then, I know an awful lot about them, and I know how to use them. I know, you know, we've had instructions and things. Obviously, my husband doesn't need one, 'cause he's got his *own*!

I: Yes. Yes. But when the incident first occurred, had you heard of defibrillators? Had you ...

R: Oh, yes.

I: Yes. You'd heard of them, but you weren't quite sure how they worked, or how you should use them.

R: Yeah.

I: Yes.

R: I mean, we knew, you know, I knew as much as that defibrillators were available, you know, they were in boxes, and you got a key ... I think at that point, I probably didn't know that you would need, you needed a key code, but I did know that the defibrillator gave you instructions. I’ve always known that.

I: Yes. Yes.

R: Yeah.

I: Okay. So, just to clarify then, there was no defibrillator there, nobody had brought you one, you hadn't retrieved one.

R: No.

I: No.

R: I was on my own. Literally.

I: Yes. Yes. Okay. Was there a defibrillator that you were aware of at the time, that you didn't go and get?

R: I wouldn't even *know* where our nearest one is even *now*.

I: Right. Okay.

R: But I’m being told there's one at the school ‘round the corner. (Chuckles)**.**

I: Okay. So, you've *just* been told that now. Yeah.

00:32:39

R: Yes. (Chuckles)**.**

I: (Chuckles)**.**Yeah. Okay. Had the call handler advised you of a nearby defibrillator?

R: No. Well, not that I remember, no.

I: No. Okay. What are your thoughts about using a defibrillator in the future? Well, I suppose you don't need to ... Or, or just in general, not, not for your husband but just in general.

R: If, if I was in, if I came across an occasion, you know, you're out and about ...

I: Yes.

R: ... and somebody took a heart attack, right, and there was a defibrillator available, I would have no qualms in using it at all. Because we've been shown, on more than one occasion since this, at various support things, how to use them, and that they're totally safe. And that in my husband's case, or anybody else who has got an internal one, if you attach another defibrillator to it, it won't shock you if the internal one has worked.

I: Yes.

R: And it also won't shock you if there's a heartbeat there.

I: Yes. Yeah, they ...

R: Yeah.

I: ... they, they, they assess whether there's a shockable rhythm, and then they, they shock ...

R: Yeah.

I: ... accordingly, don't they.

R: Yeah.

I: Yes. But generally speaking, you'd be okay to use one in the future?

R: I feel confident, in an emergency situation, if there was one around, I’d be prepared to try.

I: Yes.

R: And that's, you know.

I: Yes. Yes. And, and where do you think this confidence has come from? Because you said when the incident first occurred you hadn't really ...

R: Yeah.

I: ... heard much about, or knew about them so.

R: Because we go to a support group in the [Name of region] …

I:  Yes.

R:                … where, with the consultant that [husband Name]’s under, we go to a support group there once every three months. And we've had a lot of talks about it. They've brought one in. We've had the dummy, we've done the CPR. And also at this conference we were at last year, we got a lot of information there about them, and the advantages of them. And the advantages of getting somebody shocked as quickly as possible.

I: Yes. Absolutely. Yes.

R: Yeah.

I: Okay. So, you've been given information about it since the incident.

00:35:14

R: Yes.

I: Excellent. Lovely. Thank you.

R: Yeah.

I: And is there something that would make it easier for you to use a defibrillator in the future?

R: (Pause) well, just that I know they talk you through it, you know.

I: Yes.

R: They, you can't ... Using a defibrillator, if I’ve picked it up right, you can't do somebody harm. You can only help.

I: Yes, yeah. Okay.

R: Yeah.

I: Yeah. And you know that it speaks, that's what you're saying, that ...

R: Yes.

I: Yeah.

R: Yeah.

I: Okay. So, if it's okay with you, I just want to move on to the remaining part of the interview, which is looking at your attitudes around drones delivering defibrillators. So, is drone delivery of defibrillators something that you've heard of?

R: No! (Chuckles)**.**

I: (Chuckles)**.**Understandable, it's novel.

R: I can’t quite imagine it!

I: No, okay, lovely! That, that, well, I'll ask you about that in, in a moment, actually. Have you heard of drones?

R: Oh, yes.

I: Yes. Yes. So, have you seen drones used, or used one yourself?

R: Never used one myself. I’ve seen people on a campsite, or out and about using a drone.

I: Yes.

R: But that's about it. You know, not ... Seen it on the news in the [Name of region], 'cause they're talking about moving blood samples, and blood via drone to get it to the right place. So, I have seen that on the news, yeah.

I: Yes. Yes. But in terms of drone delivered defibrillation, and how that would work ...

R: No! (Chuckles)**.**

I: No idea. Yeah.

R: I can't ... Well, just thinking about the size of a defibrillator, and the size of a drone, it just doesn't go together, you know.

00:37:12

I: Yeah.

R: But obviously drones must be able to carry, carry things from the, you know, the weight and things, so yes. Ah ha.

I: Yeah. Okay. So, drones, as you're probably aware, are small flying aircraft that are piloted remotely, and they have camera capabilities so they can navigate and see where to go. And they can ...

R: Yeah.

I: ... carry defibrillators …

R:  Right!

I:                    … despite that sounding a little bit odd. But they can ...

R: Yeah.

I: ... by landing and detaching the defibrillator, or they can ...

R: Right.

I: ... hover above the ground, and winch the device down to the ground. So, like, with a rope like object.

R: Oh, right. Okay. Yeah.

I: So, can you imagine interacting with a drone in either of the ways that I’ve just described?

R: (Pause) (chuckles). At this precise second, no (chuckles). But I suppose if I saw one, and realised, you know, if I, I’m assuming I’m talking to somebody on the phone at the same time, if they were explaining what was happening, yes, I can understand that a, a drone would appear, as you say, with a little winch cable, and you have to detach it. So, yes, I suppose I could do it in an emergency.

I: Yes. Yeah. Okay. So, you would want to get the defibrillator, you would want to get the defibrillator from the drone if it's winched it down via ...

R: Yeah.

I: ... a cable or rope, you'd be able to get the defibrillator that way.

R: Yeah.

I: Yeah. Okay. That's great. Thank you. And how would you feel about a drone bringing a defibrillator to you whilst helping someone having a cardiac arrest?

R: (Pause) I suppose if that's the only way that a defibrillator can get there, that is good. You know, if that's the quickest way, and the easiest way, yes. You know, 'cause you can't keep doing CPR yourself forever and a, you know, for a long, long time. There's a limit. So, you know, obviously if they could get you a defibrillator, that would help. Yeah.

I: Yes. Do you think it make it easier, or more difficult for you to use a defibrillator?

R: (Pause) I suppose if you're on your own, it could be difficult. Because you're obviously in quite a stressful situation in any case, so to then leave whoever you are doing the CPR to, to then get the defibrillator, get it set up, and do it, that could be quite stressful if you were on your *own*.

I: Yes.

R: But, at the end of the day, if that's the only help you've got, I’m confident (chuckles)that you could do it.

I: Yes. Yes. Because I was going to ask you, how would you feel about leaving the patient if they are by themselves, but ...

00:40:41

R: Yeah. Well, I’m assuming that, you know, you're gonna have to. If you've got to get this defibrillator, you've got to. So, it's, you've got to work quickly, haven't you. That’s, that’s the situation. So, you know, as long as you're fit and well enough, you know, the person who's doing it, to be able to do that ... I’m sure, I’m certain I probably could.

I: Yes. Yes (participant chuckles). Okay. And ...

R: She says! (Chuckles)**.**

I: (Chuckles) how far would you be willing to go to get the defibrillator that had just been delivered by drone?

R: Oh, well, working on the theory that I can't walk quickly, I can't *run*, I would like it to be delivered very close to me (chuckles) again, if you're on your *own*.

I: Yes. Yes. Yeah.

R: Yeah.

I: Okay. So, if the cardiac arrest happens at home, would you leave the patient to get the defibrillator from the front door? Or would you maybe cross the road to get it? How far do you think you'd go?

R: You, you would have to. I mean, if I take [husband’s Name], you know, the circumstances that we had, I would have to ... Say you could deliver a defibrillator to the house, I’m not saying you could put it in the garden, but you could put it on the road outside, you would have to leave your patient. You would have to leave them, you know, if that's the only way, if *you're* on your own, you know.

I: Yeah.

R: I don't know how quickly a drone would get here.

I: Yes. Yes.

R: You know, that would be the next thing, is, you know, we had an ambulance here in just over 15 minutes, right. How quickly would a drone get here with a defibrillator?

I: Yes. Yes. So, that would be a concern that you, you have. That maybe ...

R: Well ...

I: ... might take longer?

R: ... is it, would it be an either or situation? You know, or is it a case of what would get to the person quickest?

I: Yes. Yes. The, the, defibrillator, the drone, or the ambulance crew.

R: Yes. Ah ha.

I: Yeah.

R: I mean, I can see people, you know, and maybe, you know, [Name of region], you hear about ambulances taking so long to get places. Or, like, here, you know, the rural places, then yeah, defibrillator on a drone might be the quickest thing.

I: Yes. Yes.

R: You know.

I: So, depends on location ...

00:43:37

R: Yeah.

I: ... really.

R: Exactly. You know, the circumstances. I’m assuming they're not just gonna dispatch drones off to everybody who rings 999 with a possible heart attack.

I: Yes, yes, you think it'll be more targeted, depending on where the cardiac arrest has occurred?

R: Yes. Ah ha.

I: So, do you think, then, that drones would be more beneficial going to remote places? Is that what you're saying?

R: I would think, yes, possibly. Yes.

I: Yeah. Or, and what about to people's houses? Do you think that's, could be beneficial, or not so much? What's your ...

R: It's, well, it's difficult, isn't it? It's the circumstances, and where the houses are. You, if it's a really, for an ambulance to get there with trained medical people, if, say, it's a really, and they're in a congested place, in a city centre, where the ambulance can't get there quickly, a drone might be the answer ...

I: Yes.

R: ... to get there quickly.

I: Yes.

R: But I don't know who would decide which was the, the best option.

I: Yes. Yeah.

R: Yeah.

I: Okay. Okay. So, if you were outside in a ...

R: Yes.

I: ... a public space, how far would you be willing to leave the patient to get the defibrillator from the drone in that situation?

R: (Pause) [husband says he doesn’t think it would be very far] Not very far. From here, what would you, how far from here to the other side of the road, or across there, [husband’s Name], how far was that? [Husband says he thinks it could be 50 yards]. 50 yards.

I: Yeah.

R: 50 yards or more. So, you could probably still see the patient when you were going to get the defibrillator.

I: Yes. Yes. So ...

R: Yeah.

I: ... having the patient in sight would, would be important for you.

00:45:42

R: Yeah.

I: It would be a ...

R: Yes.

I: ... determining factor.

R: So that you are still physically able to see the patient. This is all assuming you're on your own.

I: Yes. Yes. So, how would you manage it if you were with another person, then? Another bystander ...

R: I’d send somebody else. Or if they could do CPR, swap over.

I: Yes. Yes. Okay. Okay. And do you think that would make it a little bit more easier to handle the situation, or ...?

R: Yes, because you've got support.

I: Yes. Yeah. Okay. And what do you think the role of the call handler is in all this?

R: It's very important that they keep a calm voice, give very clear instructions, and keep talking to whoever's on the other end of the phone, obviously. You know, to make sure they are still focusing on what they're asking you to do.

I: Yes. Yes. And when you say instructions, instructions regarding what exactly?

R: Well, like the first, you know, the first instructions were, can I check to see if there was a pulse.

I: Yes.

R: Then it was, "Can you shake him to see if he's become responsive?” They, I do know they were the first two instructions.

I: Yes.

R: And then, obviously following that, it was then, "You need to get him on the floor. And do that the best way you can, whatever.” You know. And then flat, and then CPR. And I think she possibly said where to put your hands, but I can't remember, because I'd already vaguely knew where I had to do it.

I: Yes. Yeah. And that was because you've been trained. Yeah.

R: Yeah.

I: Yeah.

R: Ah ha. Yeah.

I: Okay. And what would you want to know from the call handler about drone delivered defibrillation?

R: Well, I think if, if the call handler then said, "Right, an ambulance with a crew is going to take ..”. I'll say half an hour to get here. “We can get you a defibrillator delivered by a drone in 10 minutes.” That would be, you know, that would be precise ... I’m assuming if a drone delivered a defibrillator, paramedics would still arrive at some point.

I: Yes. Yes.

00:48:13

R: Yeah.

I: Yeah.

R: Ah ha. So, you know, if the defib … Now that I know so much about them, if the defibrillator was gonna get there quicker to be able to shock the, the person, knowing what I know now, I would say, "Yes, can we have one.”

I: Yeah.

R: Even if it meant leaving [husband’s Name] on the floor upstairs, coming down the stairs, going out to the back, the road to pick it up, you know. And by then, I’m sure some of the neighbours would have been out in any case (chuckles)**.**

I: Yes. Yeah. And would you want to know that a drone was coming, or only when it was there? Would you want to know how to identify it?

R: Well, yes, you would have to know, you know, that it was on its way. They would have to tell you exactly where it was, you know. If, like in my, if I was in the house, and my circumstances, they would have to say, "Right, the drone will be ...” If they’d said at the front of the house, right, I would have to go out the front door, to the grass that's out the front. There's no road there, right, it's not accessible. If that's where it was gonna, I would have to know, you would have to be told exactly where this drone was gonna be, you know.

I: Yes.

R: 'Cause I think in the state of mind that you would be in, you wouldn't be going, "Where is it? Where is it?” They’d need to be quite precise as to where it was gonna be.

I: Yes. Okay. Excellent. Thank you. And do you have any concerns, or issues, or problems with this technology that you can foresee?

R: I don't. Not if it's used in the right way (chuckles)**.**

I: Yeah.

R: If it's used for the good, I haven't got a problem with technology. As I say, you know, we've heard in the [Name of region] that they're transporting blood by drone to get it to the person who needs it quicker, because of the time it takes to get it here, you know. So, I haven't got a problem with that technology. But I suppose people might, you know.

I: Yeah. Why? Why do you think they might?

R: Well, because I think people are a bit scared of technology, that it's taking over the world. And it's taking the personal touch out.

I: Yes.

R: I would say. But something like delivering a defibrillator, I think could be a, a plus, shall we say, for some occasions.

I: Yes. Excellent. Thank you.

R: Yeah.

I: I’ve taken up too much of your time.

R: No.

00:51:05

I: If I could just ask some final brief questions that may be useful for our research, if that's okay?

R: Yeah.

I: So, may I ask what is your age and gender?

R: My age. I’m gonna be 76 on [day], and I’m female.

I: Okay. Happy birthday for [day]! (Chuckles).

R: (Laughs) thank you!

I: It's okay. And you said you had training in CPR some years ago.

R: When I was at work.

I: Yeah.

R: So, I retired in [year] (pause) I probably has the first lot of CPR during [year range], and then I had a second lot during, sometime during [year range]. I can't narrow it down any further than that. I have had two lots, because I was a, I was a first, I worked in [profession], and I was a first aider for *a lot* of the years that I was there.

I: Yes. Yes. And this training occurred well like before the incident that we've discussed?

R:	Oh! Yes.

I:	Yeah. Yes.

R:	Well before yes (chuckles)**.**

I:	And no, no defibrillation training. No.

R:	No.

I:	No.

R:	No, I mean I’ve never seen a defibrillator until we did, saw one at the support groups.

I:	Yes. Yeah.

R:	Yeah.

I:	Okay. Thank you for clarifying. Is there something else you would like to add to your answers today?

R:	No, I think I’ve probably talked too much (chuckles)**.**

I:	No, it's been wonderful listening to you share your experiences, and I know that I’ve taken up too much of your time. So, I’m going to end the recording, and then just briefly chat through what will happen next, if that's okay.

R:	Yes, that's fine. Yeah.

**END OF INTERVIEW**

Participant 12

I: 	Okay. So the main interview audio recording has begun and it will continue until the interview questions end.

R:	Yes (?)

I:	Okay. So, can you tell me, please, as much as you can about when you provided assistance to the person who had a cardiac arrest?

R:	Yeah. So I was working on shifts and in one of the halls there was a, a [sports] class. I was grabbed by one of the ladies who just said they need some first aid. So I went into the main hall, didn't grab anything initially, so I was just going to assess. I went into the main hall and there was a gentleman sat among the chairs making this wheezing sound. I could see that he was going very very purple and obviously struggling to breathe, so I immediately dialled 999 for an ambulance. And, whilst I was on the phone, me and another gentleman laid the ill man down and got him into the recovery position. We then realised he had stopped breathing, so we rolled him onto his back and the gentleman I was with started with chest compressions, whilst I went back out to the reception area to grab the on-site defibrillator. I went back into the, the room, the main hall and, whilst the other gentleman was given chest compressions, I opened the de, defib and stuck the pads on his chest, where it indicated, and turned it on. The defibrillator took a reading and said that a, a shock was necessary, so we all stood clear, I pressed the shock button. It gave him a shock, it then said to us to continue chest compressions, so we continued the chest compressions. It then read his heart rate again and said another shock was needed, so I shocked him again. And then we continued with chest compressions again. And on this sort of third round, if you like, of chest compressions the man's colour started to return and we realised he was shallow breaths again with a, a faint pulse. We carried on with the chest compressions until he was aware enough to try to hit us off with his hand, and stopped then, and got him back into a recovery position, where he regained consciousness. And, by the time the ambulance arrived, he was conscious and, and speaking again.

00:03:16

I:	Right.

R:	And then they took him out, obviously out into the ambulance and took him off to the hospital.

I:	Yeah, sure. Oh, well, thank you for providing those details. It's very interesting, your experience. So, the cardiac arrest happened in a public place ...

R:	Yes.

I:	... and at your place of work, that's correct?

R:	Yeah.

I:	Yeah. Okay. And the person who had a cardiac arrest was a stranger to you, presumably?

R:	Yeah, it was his second time attending the, the badminton club that, that goes on there.

I:	Okay. And that's where you work, in that club?

R:	Yes.

I:	Yeah.

R:	I, I work in the building that the club hire.

I:	Okay. Okay. And so you realised what was happening at first because he stopped breathing, is that correct?

R:	Yeah, I kind of knew it was serious when I just saw the colour going, ‘cause obviously I realised he, he wasn't getting oxygen in. And then, yeah, as soon as we, as soon as we couldn't feel breath or a pulse, that's as soon as we started the chest compressions.

I:	Okay. So that's when you realised it was a cardiac arrest, when you couldn't feel ...

R:	Yeah.

I:	... the ... right, a pulse. Okay. And how soon afterwards did you realise it was a cardiac arrest?

R:	As soon as we started on, on the chest compressions, I knew I needed to get the, the defib, so pretty much it was quite quickly, thankfully.

I: 	And you say he was taken to hospital?

R:	Yeah, he was taken to the hospital. They done a bypass surgery on him and installed a defib into him. And, yeah, as far as I’m aware, he's, he's still alive. I did see him a few weeks after the incident when he came in to say thank you.

00:05:13

I:	Oh, oh, that must ...

R:	But, yeah.

I:	... have been ...

R:	(Chuckles).

I:	Yeah, yeah, that must have been nice for you. So, you said that the other bystander who was with you performed CPR.

R:	Yeah.

I:	Did you also perform CPR?

R:	I done a round of chest compressions between the two defibrillator shocks (inaudible) to continue ...

I:	Okay.

R:	... with the chest compressions.

I:	Okay. And I'll ask you to go into that in a bit more detail in a moment.

R:	Yeah.

I:	But, for now, you said that there was this other bystander there with you ...

R:	Yeah.

I:	... how did that make you feel?

R:	Yeah, it made me feel supported. It was, it was nice to have, (inaudible) wouldn’t say nice but it was nice to obviously have someone who was, who had been trained as well, obviously, to, to deal with, like, a real life situation. So, yeah, I think we both kind of were able to support each other. We both took the lead, ‘cause obviously we had an audience, while all this was going on, the other badminton players, so, yeah, it was, it was good to kind of have a key team, if you know what I mean, to focus with.

I:	Yes, yes. So there was other people there as well, onlookers, would you say?

R:	Yeah, so (inaudible), ‘cause it's a, it’s a hall with four courts, obviously other [sports] players kind of realised something was wrong quite quickly. So some of them, sort of, like, took themselves out of the, the hall, others were just watching.

I:	Just watching. Okay. Did they intervene at all at any point?

R:	A couple of them might have asked if we needed anything. I think one of them was out looking for the ambulance. And obviously getting a cushion or, or something, sort of, I, I think they wanted to help, but obviously whilst me and this other gentleman were actually performing the CPR, they kind of didn't want to interrupt.

I:	Sure. How did you find all these onlookers around? What …

R:	… To be honest, I wasn't focused on them. (chuckles) so, as much as I was aware they were there, they were there, I also, they weren’t the, the focal point of my attention.

I:	Yeah, sure, sure. And this other bystander who was helping you, did they ...

R:	Yeah.

00:07:42

I:	... use the defibrillator, or was that just you …

R:	No, that was just me. I, I administered the shocks and put the pads on.

I:	Okay, okay. So the other person was primarily responsible for doing the chest ...

R:	He done the chest, yeah.

I:	Okay, excellent, thank you. So can you tell me as much as you can about the 999 call, please?

R:	Yes, I dialled 999, obviously asked for the ambulance. I said, “There's a man who stopped breathing,” and I, I think, I can't remember fully, I used the word ‘cardiac arrest’, ‘cause I’ve had a history in sport myself and obviously I’m aware of sort of, like, the dangers that can happen. So I used ‘cardiac arrest’ I said we was administrating CPR. And then it, it was a bit (inhales loudly) it wasn't panicky, but it was kind of difficult, ‘cause I didn't hand the phone I had the phone on loudspeaker whilst trying to set up the defib and talk, so I mean for the operator it was probably a bit hectic, I guess, ‘cause they would have heard other voices in the background. And basically their, their advice was to listen to what the defib was telling us to do, ‘cause obviously the defib was, sort of has a voice, it was speaking. So if it said to do CP … chest compressions, to do it, if it said to administer the shock, do it. And that was kind of the only advice that I was given over the phone. Oh! And they counted as well, that's right, as we was doing chest compressions, they were going, “one, two, three, four” you know, at the rate that we should be applying it.

I:	Mmm hmmm. And how did you find this help and advice?

R:	It got, it was slightly confusing towards the end, ‘cause obviously, like I say, the gentleman did regain some consciousness and whilst, whilst he was moving and trying to stop us giving chest compressions, he wasn't fully conscious, obviously, but he was moving, the, the machine still, the defib still says he needed a shock.

I:	Yes.

R:	Which we didn't do, ‘cause we thought, “Well, he’s, he’s coming around,” like, his colour had returned. We carried on the chest compressions obviously until he was fully conscious. But then, yeah, it was kind of difficult, ‘cause he was talking. Obviously the, the lady on the phone operator was saying, “Administer shock,” or, “Continue CPR,” ‘cause that's what the defib was saying and she could hear that in the background, but he was regaining consciousness.

I:	Right, okay. So you made the executive decision to ...

R:	Yeah.

I:	... to stop that. Yes.

R:	Yeah.

I:	Right, okay, I see. And did you tell the call handler that, or how did they respond to that?

R:	Yeah, I said to the call handler that, you know, “He's moving, he's resisting chest compressions,” but because the defibrillator still said, “Administer a shock,” or, and, “Continue CPR,” she would then encourage us to continue it. But, as I say, we didn't do that.

I:	Yeah. Yes, yeah. Okay. So did you, you presumably used a mobile to speak to the call handler?

R:	Yeah.

00:11:06

I:	Yes, yeah. Okay. And did you leave the patient to make the call?

R:	No. The only time I left the patient was when I went out and the (?) chest compressions had started by the other gentleman, so I went to get the defibrillator.

I:	Okay. Okay. So someone was with the patient the whole time?

R:	At all times, yeah, yeah.

I:	Yeah. Okay. And what about when the ambulance crew came in, did either of you leave the patient then to get them (inaudible)?

R:	He was quite a heavy gentleman, so actually we stayed, stayed with him, ‘cause I was talking to him by the time the ambulance crew came in, and his wife, somebody at the club had called his wife, ‘cause they had an emergency contact, she had arrived too. So I was kind of telling the ambulance paramedics what had happened, I was speaking to the wife. And then, like I say, he was quite a big gentleman, there was a few of us that actually had to help lift him up onto the, the stretcher.

I:	Yes.

R:	And I carried the oxygen tank out next to, next to him as he went out to the ambulance.

I:	Yes. And who let the ambulance crew in then?

R:	So there, we have a fire door that goes into the main hall, so other, I think they were propped open anyway actually already, ‘cause it was hot. But, as I say, other, other badminton club were there waiting, so when the ambulance came they just directed them straight to the main hall through the fire exit.

I:	Yes, yeah. Okay. And can you remember what questions the call handler asked you?

R:	(Exhales and pauses) I can't, I, I, and it sounds silly, I don't actually think she had a chance to, because I, I'd already said to her that we were performing CPR, because it happened so fast, within me going into the hall and making that initial call, within 30 seconds or so we were performing CPR. So I don’t think ... I’ve called the ambulance before and they, you know, ask, “Is the patient breathing?” and whatnot, but we’d ... or I don’t ... she may have asked that, I can't remember, ‘cause obviously already, I already knew what needed to be done, so I was, I was just hoping an ambulance would get there fast (chuckles).

I:	Yeah, yeah, of course, understandable. Did you ask the call handler any questions?

R:	No.

I:	No. Okay. What about the other bystander, did, did they ask?

R:	I mean the only time we asked, asked a question is, “Do we need to continue CPR, because he's responding?” Even though the defib was telling us to. And, as I say, her response was to listen to the defib, but then we ultimately didn't do that.

I:	Okay. And what are your reflections on the interaction?

R:	With the call handler?

I:	Yes, yeah.

00:14:06

R:	(Pause) I feel with the defibrillator it, it, it wasn't, it wasn’t, like, effective, if that makes sense. The defibrillator was the thing that was telling us what to do. Obviously if we didn't have a defib it would have been a different story. With, with the operator counting it was helpful whilst everything was going on to keep them sort of chest compressions at that time that she was counting. But, yeah, that, that was about it really.

I:	Okay. And apart from when it got a bit confusing ...

R:	Yeah.

I:	... what, was there anything else that perhaps was difficult while interacting with the call handler?

R:	In hindsight, if, if it was to happen again, I'd probably give my phone to somebody else who might be there, or if there was other people. It's not obviously just being able to listen to them, I think if the other gentleman perhaps wasn't there, you know, o, obviously I would have had to start chest compressions myself, but it would have been a bit more difficult, I would have asked more questions in that instance.

I:	Yeah, yeah. And were there any technical issues while you were talking to the call handler on the phone?

R:	No, it was all good.

I:	Okay. So, can you go into some more detail about performing the CPR?

R:	Yes. So as I said (?) the, the defib read his, his rate, heart rate and we administered a shock and then continued chest compressions, so I took over. I’ve done, sort of, like, the [Name of organisation] CPR training and, and stuff like that, and first aid at work. So I put my hands on his chest, interlocked my fingers and then started pressing. And the call handler was obviously going one, two, three, four, so I was doing it with that beat, whilst others were there, sort of, like, as I say there’s others around. And then, yeah, the, the heart rate monitor, I don’t know how long it, how long it was between reading the heart rate, but it said “Reading heart rate,” so at that point I stopped. It read the heart rate and said, “Administer another shock,” so I administered another shock. And then the other bystander took over the chest compressions again.

I:	Yes. So did you do chest compressions only or did you do these with ventilations or rescue breaths?

R:	Just chest compressions.

I:	Yeah. Okay. And what about the other bystander, did ...

R:	Yeah, it was just chest compressions, we did, obviously, tilt the man's head back, open his airways ...

I:	Yeah.

R:	... but no one got any sort of mouth to mouth or anything.

I:	Yes, yeah. Okay. And what are your reflections on how the CPR went?

R:	Well, it worked (chuckles slightly) in this instance, so thankful for that. Yeah, it's always a bit, a bit strange when it's kind of, like, you feel ribs, you know, sort of cracking beneath, beneath your hands, obviously, but it was, yeah, it was successful, so it was (pause) I think it went okay.

I:	Yes, sure. And you said that the call handler was counting ...

R:	Yeah.

00:17:36

I:	... which was helpful for you.

R:	Yeah.

I:	Did they offer or give you any other instructions?

R:	No, I think they were just counting, yeah, they, they just counted until obviously the defib again said, “Reading heart rate,” to see if it needed to do another shock or not.

I:	And what are your reflections on the advice and the instructions that you received, that you, that were given to you?

R:	Apart from the counting, quite minimal, actually. Not, not through any fault of the operator, I suppose, but just because of the circumstances around it, we were already performing CPR when, when they answered and we had the defib talking to us. So, yeah, quite, quite minimal in this instance.

I:	But that wasn't ... was that problematic for you, or was it okay?

R:	No.

I:	No.

R:	It was okay, yeah.

I:	Yeah. Okay. So you mentioned that you use the defibrillator ...

R:	Yeah.

I:	... could you just tell me a bit more about your experience of using this, please?

R:	Yeah. As I say, I went, I, I grabbed it off, off the wall, we have it in a little orange bag. I took it in, un, unzipped it, got out the actual device. The pads were at the back, so I pulled the pads out. And on the pads itself, it tells you where to stick them, so, like, one and two. So lifted up the gentleman's shirt and I stuck on pads one and two. Turned the device on. And as soon as I turned it on, it started talking quite loudly, which is good (chuckles) and, yeah, it, it basically monitored his, his heart rate straight away, and it was very quick in saying that a shock was needed. So then an orange button sort of, like, flashed, which has got a, like, a lightning bolt symbol on it, so I, I assumed it meant a shock. So, yeah, pressed, pressed that, it delivered the shock, and then it say, “Continue chest compression, continue CPR.” So it continued with that until the machine then once again said, you know, “Shock advice, monitoring heart rate.” So, yeah, it, it was helpful, it was very, sort of, self-explanatory and, yeah, it was, it was a good guidance. I, I felt it offered some sort of level of reassurance to some people there at least, because it was, it was monitoring the heart rate and it was, it knew when we needed to administer a shock.

I:	Did you feel that it was reassuring to you as well?

R:	Yeah, I felt reassured. I was a bit apprehensive, obviously, of, of, of giving a shock (chuckles) which sounds silly, I know, but obviously I kind of didn't know what to expect when I pressed that, pressed the button.

I:	Yes.

R:	But I knew it needed to be done, so, you know, I just pressed it but you know, it, it gave him a shock, obviously, his body jolted with the shock, so it was obviously quite powerful. But then as soon as it said to continue, sort of, chest compressions, we cracked on and continued with that.

00:21:10

I:	Yes, yeah. So would you say it was easy to use and understand, or ...?

R:	Yeah, definitely.

I:	Yeah. How easy would you say it was?

R:	Very easy. I, I’d be confident … for a new member of staff at my work, I'd be confident that if a situation like that was to happen, that they'd be able to use it and use it effectively, just because of the instructions that the defibrillator gives itself.

I:	Yes, yeah. And how comfortable did you feel using it?

R:	Yeah, I'd say it was, it had all the details and explanations on it, so, I felt quite comfortable using it, even though, like, it's my first time using it in real time. But, yeah, it was, it was nice to know that ... I, I knew it was the right thing to do, I knew that this was, that this was reliable in, in what it was telling me.

I:	Yes, yeah. And following the defib voice instructions, how did you ...

R:	Yeah.

I:	... find that?

R:	Yeah, like I say the only bit that was confusing was towards the end, when he was reacting and the defib kept saying, you know, “Shock advised,” but we didn't feel it was necessary, ‘cause he was, he was coming back around. Obviously if he had stopped breathing again or something, then we would have given a shock. And then it was, I, I seem to remember us trying to turn it off, or turn the volume down was quite difficult, once he had come back around, ‘cause it was still obviously monitoring him when he was back around and regained consciousness.

I:	Yes, yeah, of course. Did the call handler offer any instructions about using the defib?

R:	No.

I:	No.

R:	It was just sort of, “Follow the instructions on the defib.”

I:	Yes, yeah, okay. So how did you find following the defib and the call handler saying, “Follow the defib,” at the same time? Was, was that difficult?

R:	It got a bit I'd say confusing towards the end, ‘cause defib was saying one thing and the handler was encouraging us to do what the defib said, but at that time we didn't feel it was necessary, because he was breathing again, his colour was returning, he, he was fluttering his eyelids, you know, he was coming back around, so that was a bit confusing.

I:	Yeah. So did the paramedics say anything about, about that? Did they ...

R:	No, when the paramedics arrived, ‘cause he was already, he was speaking again and talking when the paramedics arrived, so, you know, the paramedics came and done their tests, whatever they done, gave him oxygen, but he was already back and responding to them.

I:	Yes. Yeah. Okay. I just wondered if you managed to figure out why, you know, the defib was telling you to do that when he was, like you say …

00:24:18

R:	Nah, didn’t, didn't ask the question (chuckles) I didn't think to ask the question.

I:	No. No, no, no, of course not, it was very chaotic and, you know ...

R:	Yeah.

I:	... busy and, yeah, a difficult situation. So there was a defib on site that you ...

R:	Yeah.

I:	... retrieved, was the fact that there was a defib nearby mentioned by the call handler?

R:	No. Whether that, I mean I think I, whilst the phone was ringing I'd already ran down and was getting it, so I said to her, “We have a defib.”

I:	Ah, yes. Yeah. So, yeah, she didn't need to tell you then to get one.

R:	Yeah.

I:	Okay. And how far did you go? Was it in the next room or did you have to go down stairs?

R:	Yeah, roughly about five, six metre corridor basically.

I:	Yeah.

R:	Just from the reception into the main hall.

I:	Okay. And how did you feel about this, about having to go to get the defibrillator?

R:	(Exhales and inhales) I, I think excite … excites the wrong word, I had an adrenaline rush, obviously. I, I was like, “Oh, this is something serious happening.” I also thought, “Why me?” (Chuckles) “Why on my shift?”

I:	Yeah.

R:	But, yeah, I had that little adrenaline boost, which I was able to just funnel that, that boost into actually doing what I had to do. I think, like, my profession for quite a few years was a, was a funeral director, so I was kind of being used to situations around death and obviously deceased, so that part didn't put me into a panic of any kind. I just kind of knew that this is something serious and that, that it needs me to funnel and focus my attention and use whatever adrenaline’s going through me to, to focus on, on the, the man.

I:	Yeah. May I ask, like, why you thought, “Why me?” or how that made you feel when you thought, “Why me?”

R:	I just thought it was, it was almost like one of them typical sort of things, I was like, “Oh, why on, on my shift?” I also thought, ‘cause obviously people here know about my past profession, [describes this] like, yeah I just kind of thought, “Oh, you know, he’s, he's had a card … he’s had a cardiac arrest.” You know, if, if he wasn't successful in our CPR, you know, on my shift, you know what I mean, you could put two and two together or people would be like ... yeah, think it's coincidental.

I:	Yeah, yeah. Okay. And what are your thoughts about using a defibrillator in the future?

R:	Yeah, definitely. I actually had a case on [recent date], in, in a [sport] session again, this time in the morning, where a gentleman collapsed and his breathing went funny and he was, he was almost hyperventilating while he was unconscious. So I thought, once again, I, I got the defibrillator on this [recent date], but, thankfully, by the time I'd returned, he was already coming back around, so I only had to use it as a pillow whilst using the recovery position. But, yeah, so I, I … it's one of the things where if that situation was to happen, one of the first thing that crosses my mind is locate a defibrillator.

00:28:03

I:	Yes, yeah. And is there something that would make it easier for you to use a defibrillator again?

R:	Possibly the volume button (chuckles) just across towards the end, ‘cause they are loud and, you, you know, loud and that's good, but then also, like I say, if there's an audience or if some people are unaware of what's going on, they might suddenly become aware, just because the loudness of the, of the defibrillator.

I:	You know you say like you had an audience, were, would they, were they difficult at any time? Did you have to manage them? Sorry.

R:	No, they were all respectful, ‘cause they were all obviously quite, quite middle aged, older, they, they, they kept a sensible distance away, they only kind of got involved if they knew they could help in, in some way. Other than that they, they just sort of mingled if you like.

I:	Yes, yeah, so they weren't a bother to you.

R:	No, nah, they weren’t getting in the way at all.

I:	Okay. Excellent, thank you for sharing your views this afternoon. So I’m just going to move on to the remaining part of the interview, which is looking ...

R:	Yeah.

I:	... at drones and delivering defibrillators to the scene of a cardiac arrest.

R:	Yeah.

I:	So is this technology something that you've heard of?

R:	(Inhales). No. No, obviously I know about drones, but not directly about delivering defibrillators.

I:	Okay. So have you seen drones used or used one yourself?

R:	Yeah. I haven't used one myself, but I’ve seen them used.

I:	Okay.

R:	Mostly it's sort of, like, videography (pauses) uses.

I:	Geography uses?

R:	Videography, sorry, video.

I:	Videogeography (sic), okay. So is this, like, on TV or is that what you mean?

R:	Yeah, like, I, I, I work within a football club, too so obviously our, our media team use drones to get film footage and that.

I:	Ah, okay, so you work with people who use drones.

R:	Yeah.

I:	Yeah, but you haven't used them yourself.

R:	I haven't used one myself, I don’t trust myself to be honest.

I:	(Chuckles). Okay. So, in that case then, how do you imagine drone delivered defibrillation works?

00:30:33

R:	Perhaps, I know that you have defibs located in different places, I dunno how, how expensive it would be, but if there's a drone that would leave or be there, or maybe an ambulance centre or station and it goes to its nearest, if there's a defib there and it's in range, it could fly there? I’m, I’m not too sure.

I:	So you're aware drones are small flying aircraft that are ...

R:	Yeah.

I:	... piloted remotely and they have cameras, so they can ...

R:	Yeah.

I:	... navigate and see where to go. So ...

R:	Yeah.

I:	... they can carry defibrillators by landing and detaching the defibrillator, or they can hover above the ground and winch the defibrillator down to the ground.

R:	Okay, yeah.

I:	So could you imagine interacting with a defibrillator in the ways that I’ve just described?

R:	Yeah, 100%, yeah.

I:	Okay. Does any way sound more feasible, or do you think they're both …

R:	Obviously winching it down, I think that would work more so, ‘cause it's, it's one less thing I guess for a drone to consider if you’re not then finding somewhere to land. I’m just trying to think in the instance that I *had*, are the drones mobile enough to, like, fly through a doorway or would it deliver it outside the building?

I:	(Pause) Are you asking me? Sorry.

R:	Yeah! (Chuckles).

I:	(Chuckles). So they'd probably deliver it at the doorstep ...

R:	Okay, yeah.

I:	... particularly if it's at a house.

R:	Yeah.

I:	And I was going to ask you some questions about location and proximity in a moment and how you would feel ...

R:	Yeah.

I:	... about leaving the patient to get the device ...

R:	To get it, yeah.

I:	... in, in a moment, if, if that's okay.

R:	Absolutely.

I:	So, so, yeah, how would the defibrillator get safely to the ground? Do you think the way, one of the ways that I’ve, or both ways that I’ve described, what ...

00:32:41

R:	I think, I think the, the winch would perhaps be more effective, just because obviously the drone is always at a safe distance from anything, ‘cause it's winching it down ...

I:	Yes, yeah.

R:	... rather than landing and something, a third party or something somehow circumstantially affecting the drone itself.

I:	Yeah, yeah. So then how would you go about getting the defib? So you're saying winch it down and then how would ...

R:	And then I'd probably, if it was myself, if, if I was with somebody I'd get someone else to fetch it. If I was on my own, I would probably feel, depending on the distance, comfortable enough to, to leave the patient for five, ten seconds to run out and grab it.

I:	Okay. Okay, interesting, thank you. So how would you feel about a drone bringing a defibrillator to you whilst helping someone having a cardiac arrest?

R:	If, if, if I was in that situation, I probably, I probably wouldn't actually acknowledge the drone in, in itself, I would just be thankful that a, a defibrillator was being dropped off ...

I:	Yeah.

R:	... and was being delivered to my location.

I:	Yes. Do you think it would make it easier or more difficult for you to use a defibrillator?

R:	I don't think it would have much effect to be honest. Would, would the defibrillator be, it would be dropped off by the drone, wouldn't it? So the, it, the defib would still be a defib as normal, so I think yeah it would still be absolutely fine.

I:	Yes. Yeah. Okay. Yeah, it would still, it would be intact, it would probably be ...

R:	Yeah.

I:	... in a box and it would be dropped by the drone and, yeah, you would retrieve it and bring it to the patient.

R:	Yeah.

I:	So you don't think that would have any bearing on how easy it would be for you to use it, or ...?

R:	Absolutely not, no. As long as the, as long as the, as soon as, as soon as I turn the defib on and as long as it does what it's meant to do, yeah, I'd be comfortable using it still.

I:	Okay. So I think you said that you would feel okay about leaving the patient?

R:	Yeah, I would, you know, I wouldn't feel 100% about it, but if I knew that actually getting this defib, being away for ten seconds, is gonna improve the chances of restarting a heartbeat, then, you know, them three seconds, as much as they are precious, I would, if I was on my own, go get the defib. I'd rather ... you know, if I, if I could continue doing chest compressions and shout and somebody else grab it for me, that would obviously be ideal. But on the scenario that I was, if I was on my own and it, I would, I would leave the patient for, like I say, ten, ten seconds so as just to go grab it.

I:	So how far would you be willing to go to get the defibrillator?

R:	If I could see it or if I knew directly where it was, if ten metres even, like I wouldn’t, I wouldn't run 100 metres, let's say, if it was all that far away, I would definitely want it to be closer than that. I wouldn't, I wouldn’t judge it by distance as such, I'd judge it by the amount of time that I think it's gonna take me to get it.

00:36:04

I:	And would location affect your thinking on this?

R:	Yes, probably.

I:	Oh.

R:	The, the, the reason why I say like I’m trying to think about a lot to do with like working so if it was upstairs, for example, and the drone could only drop it off, then that's gonna take, even though it might drop it off just outside, that's gonna take longer to go and get it. Whereas we have, like, a slanted roof, so if it's winched down and we have bay windows open, if it was winched somebody could grab it through the window, or I could grab it through the window, which is obviously gonna cut that time of running downstairs to get it off the floor.

I:	Yes, yeah, so if you were at your place of work and it delivered the device at the doorstep, you wouldn't, you wouldn’t go down the stairs, it would be too, take too long …

R:	No, that, that would be, I'd judge that as too long to leave the patient alone.

I:	Yes, okay. What about if you had to manage a cardiac arrest at home, would you leave ...

R:	Yeah.

I:	... the patient to get the defibrillator from the front door? Or would you cross ...

R:	Yes.

I:	... a road and get it.

R:	Yeah.

I:	Yeah, from the front door. Okay. And what about if it was delivered across the road from you?

R:	I would probably try and get somebody else to go get it. How I'd do that, I don't know, so actually, thinking about it (pause) oh, it’s a tough one, I’m not sure, to be honest (pause) if it’s ‘cause where, where I live, the road’s quite far away, ‘cause I live in a courtyard.

I:	Yeah.

R:	So if it was dropped off in the courtyard, this is, we've got this green space, and, yeah, I would go out and get it.

I:	Yeah.

R:	If it was delivered at, at the roadside of where I, I live, just ‘cause of the circumstances in which where I live, then no, I probably wouldn't go get it.

I:	Okay. Okay. But you would if it was dropped off at your doorstep?

R:	Yeah.

I:	Yeah.

R:	Or, or just, like, five metres of the courtyard that's outside the house.

I:	Yes, yeah. Okay. And what do you think the role of the 999 call handler is in all of this?

R:	To give advice, basically, to, to obviously in their job to grade the call that they feel necessary to get the response that, that is necessary, but they’re there to give advice to make sure that, that me, if I’m the person on the phone, I’m giving the best, sort of, care that I can at that time.

I:	Okay.

00:38:43

R:	So, yeah, sort of, as an advisor.

I:	Okay. What advice in particular?

R:	Like, is the patient breathing and how to check for their breathing. When to start CPR, if necessary. You know, how to, how to do that, how to lock my hands together, where to do the chest compressions, and then the rate of what to do and that. And, and then if I was to give, sort of, breath ... mouth to mouth, whatever, when to deliver that.

I:	Okay. And what would you want to know from the call handler about drone delivered defibrillation?

R:	If it was an option. If they could say, you know, “We can get a defib off, delivered to you by drone,” and, kind of, the time response on that, then, yeah, I’d, I'd 100% be giving them that information and answering their questions to, to get that.

I:	Would you want to know that one was coming, or only when it was there?

R:	Yeah, I'd like to know if one was coming. Obviously, if I knew it was gonna, I'd want to know whatever's gonna be the fastest response. So if the drone was coming and it was gonna be faster than, than an ambulance, which I imagine it would be, then, yeah, I'd want to know when it's coming. And, being a drone, having an ETA (chuckles slightly) of when it's gonna get there.

I:	Yes, yes. Would you want any information on identifying the drone?

R:	Yes, please. I'd like to see (chuckles) like, what it's gonna look like, ‘cause, you know, I know what drone looks like, but it would be, it would be my first time seeing it, so, yeah, I'd definitely wanna know what to look out for. Or even a sound, you know, would you hear the, the hum of a drone?

I:	Yes, yeah, okay. And what concerns, if any, do you have about drone delivered defibrillators?

R:	Nothing ... well, I suppose, you know, if, if, if it's a winch, if the mechanics of the drone were to go wrong, that would be my main concern. Obviously, the same as any machinery, things can sometimes just go wrong. It might, might go to the wrong location, it might, you know, be, be affected by weather, it, if it hits a tree or something, or, you know, stuff like that, basically.

I:	Yes. And any problems, any other problems or issues do you foresee?

R:	If it was one that landed, you know, safe places to land, if there was children or animals about, you know, if it's in a public place, like a park. If it detached the defibrillator correctly. Yes, also, also, like, just mechanical mechanisms that could go wrong would be my primary concern, if I could, if I just couldn’t get that defib when I was expecting it.

I:	Yes, yeah, if you couldn't get it from the drone you mean.

R:	Yeah.

I:	Yeah. Yeah. Okay. Excellent, thank you. So I just have some final brief questions that may be useful for our research ...

R:	Mmm hmm.

I: 	... if that’s okay. So, if I may ask, what is your age and gender, please?

00:42:17

R:	So I’m 27 and I’m a male.

I:	Okay. And have you had training in CPR and defibrillator use?

R:	Yes.

I:	Okay. So when was this?

R:	Last year, [Name of Organisation], and then online course from the [Name of Organisation]

I:	Okay. And these training events both were completed last year?

R:	Yeah.

I:	Yeah. Okay.

R:	So we, we, we do have another first aid and defib training at the end of this month.

I:	Okay. Okay. So you can refresh your learning.

R:	Yeah.

I:	Yeah. Okay. So when you did it last year, was it CPR only, or CPR and defibrillation?

R:	CPR and defibrillation.

I:	Okay. And did it occur before or after the incident that we've discussed?

R:	After.

I:	After. Okay.

R:	Yeah.

I:	So you hadn't been trained in ...

R:	I hadn't been trained when I first used the defibrillator.

I:	Okay. And had, and I suppose you hadn't been trained in CPR either?

R:	I’ve been trained in CPR from obviously, like I say, when I was involved in sports, so we've had to do, like, first aid courses in CPR, but not specifically for defibs.

I:	Okay. Okay. Thank you. So, before we finish, is there something else you would like to add to your answers today?

R:	Nah, not really, not, nothing, nothing helpful anyway (chuckles).

I:	Okay. So I’m just going to turn off the audio recorders and just go through ...

R:	Yeah.

I:	... what will happen next.

R:	Okay.

END OF INTERVIEW

Participant 13

I: 	Okay. So, the main interview audio recording has begun, and it will continue until the interview questions end. Okay. So, can you tell me as much as you can about when you provided assistance to the person who had a cardiac arrest? And please use your experiences from when you acted in a lay capacity, so off, in an off duty capacity.

R:	Yeah. So, this was whilst I was a first responder actually, but I was on holiday, so it's purely a lay person account. I believe it was around [two year range]. I was on holiday in a south [Name of UK nation], southeast [Name of UK nation] [holiday park] with my family for [Name of holiday], which was also my birthday. We'd sat down for a late lunch in a grill owned within the [holiday site] by the sea, and we just started our, started to eat. And I was with my two small children, and my ex-wife, my wife at the time. And this was early after, mid afternoon-ish. And around the same time, well there, there was a sudden crash and a bang. And one of the waitresses that had served us had fallen to the floor, and there were screams from the other waitress, who ran the other way. And of course, I stopped eating and immediately ran over, and my wife took care of the kids to try and ensure they weren't frightened. Moved the table and all the cutlery that had been spilled on her.

00:01:51

Obviously she'd hurt herself on, whilst falling. Other members of the public moved out the way. There was no one assisting me at the time. To be fair, I think that other waitress had gone to call for help without assessing the situation. The lady was in a seizure, from memory. I did document this as an anonymised case study for my [masters degree], on direction from my academic supervisor at the time that it was an example of poor public access defib ownership at the time. Back those 10 years plus ago. And I also sought help from my [Name of ambulance service] supervisor, my [Name of ambulance service] ambulance supervisor post incident. The lady appeared to be frothing at the mouth, and she was having a seizure, and she was blue. I opened and assessed the airway and breathing, and she was not breathing effectively. So, I screamed out for the receptionist, to, the receptionist, the other waitress to get help. I believe she called it on a walkie talkie for the security to come. And I asked her also for the defibrillator, which I'd seen in the, we'd been there to this [holiday site] many times, and I proceeded to ask for the defibrillator which was right opposite the restaurant, bar, grill, at, on the wall outside the swimming pool, I believe. No, it was inside the swimming pool, in a cabinet at reception of the leisure pool. So, I asked for that defibrillator, knowing where it was, 'cause we'd been, I think we'd been swimming that morning even. And I just continued CPR. She started to pink up, she showed signs of ROSC. So, I believe, without going back through my records, which I also documented as is standard practice as a qualified nurse, to protect myself with the [Name of professional body], with regard to any, sort of, litigation out of work or anything. So, I would have done this statement anyway, even if I’d kept it to myself. And then I achieved ROSC at some stage. I believe it was before security arrived. I continued to ask for the defibrillator, and they demanded to know who I was. I said, "I’m a qualified nurse. Obviously, I’ve responded to this incident. This is my findings.” They said, "Well, she looks alright now.” I believe I'd put her in recovery position by that stage. They didn't try and take over or anything, but they, they, they were more worried about demanding to know my professional status, because of the way I look. Long hair, I had an [Name of band] t-shirt on. It was before I had a beard. They obviously were j-judging me as a person, and they didn't, they basically inferred they didn't believe I was a nurse. They then, I remembered, at the time we used to have a, a, [Name of regulator] card, or [Name of regulator] card, because it was before, it was, like, before online registrations and things, or round about it coming in. And I had an out of date one in my ... Yeah, it would have been out of date, so it was probably around the time they brought it in. So, I had an out of date one and I showed them. And it didn't have photo ID on, but obviously they could see all my other photo ID, and credit cards and things. So, I showed that to them. I continued to maintain the lady’s airway. Prior to, whilst I was doing CPR, before the security arrived ... Sorry, I’m doing this from memory. I remember the waitress, when she did come back and say help was coming, and I said, "Look, has an ambulance been called?” And she said, "Security are doing it, I think.” And they’ll meet her, one of them will meet them at the front gate of the [holiday site]. She said, "All we know ...” I said, "Is there any medical history?” And she said, "All we know is, all I know is that she's recently been told to stop drinking.” As in alcohol. Her GP said that her results were very poor, and she was, sort of, joking with her colleagues that, you know, she was gonna struggle, and she, she'd stop drinking suddenly. So, immediately, obviously as an experienced clinician, I thought of alcohol withdrawal, very acute alcohol withdrawal which shouldn't have happened. And that she may have had a seizure from sudden detox syndrome, and that's probably why. And I definitely couldn't feel a pulse, you know, she, she was ... As a clinician, obviously, I also felt for a pulse, and she didn't have a pulse, and she was unconscious after a seizure, and frothing at the mouth, changing colour, and not breathing normally. So, I had every right to start CPR. When, not only were the security denying me the defib, wanting to see ID, they

00:07:05

also were disputing that she'd been in cardiac arrest. So, it was a very challenging, sort of, 10, 15 minutes with her on the floor. I think they did eventually bring the defibrillator, but then, for the poor lady, I think she was starting to come round then. But I still had her in recovery, 'cause she wasn't quite around. And then, from memory, the crew arrived, and it was quite a young paramedic crew that had been met at the gates by another security officer who arrived with them, from memory. And I, unfortunately, I had an equally difficult time with them. I don't know what they'd been briefed. So, they, they went on to continue to question my clinical decision making, my (pause) my, my clinical decision making and my professional standing. To the point where the one clinician, ambulance clinician, asked me to the back of the ambulance. No, I believe I helped them get the lady into the ambulance, and I was handing over as we went. And it was quite a difficult conversation. And I showed her the card again, and she said, "How do we know you're trained? How do we know you, you can use a defibrillator? Not everyone can just grab one and use one.” I said, "Well, actually, that's not true.” And I’ve been part of national group, a campaigner myself, to make public access defibs available for all. And we've succeeded in [Name of UK nation] doing that, through [Name of database listing locations of public defibrillators], and the [Name of organisation], and, you know, my research which continues with [Name of government programme]. But this was, this was why it was included in my research at the time about public access defib for my masters, because it was very challenging. And the attitudes of the staff were that they shouldn't, that they were only, sort of, for the lifeguards, or the security guards that had training. She said, "Well, I know nurses, and they aren't defib trained. So, what ...” And I, I even went as far, and this is, this is definitely very accurate from memory. I even went as far as to say, "I’m an instructor for advanced life support in adults and in paediatric life support. I am qualified to use a defib. I’m an acute critical nurse, and my background is coronary care.” You know, and, and she just wouldn't have it. In the end, this paramedic asked for all my details, my personal details, and wrote it in her notebook. And said, "Your details are going to be passed to the consultant in the emergency department where we take the patient.” Obviously I was quite scared at that point that I was gonna have some, sort of, investigation about me. My family had already gone elsewhere. They were quite distressed, as you can imagine, young children. I was quite shaken up by it, and it obviously ruined the rest of the day. They took the patient off. I remember contacting the emergency department myself that evening, to get some sort of closure, and trying to at least have a fairly decent evening. We had some social events lined up with my ex-wife's family. And to try and get some, sort of, peace, I contacted to see how the patient was, and I explained all my details. The nurse was brilliant. She, I said, "Look I work in [Name of hospital/health board], blah, blah, blah.” And she said, "I can't tell you too much, as you know, but the patient is doing well. She's come round. She is under alcohol ...” Y’know, she didn't say anything about the alcohol detox, but they said, you know, "She is gonna go for further investigations, but don't worry. She’s, she’s made a good recovery.” And I said, "Has the consultant, sort of, said anything about her pre-hospital care?” And they said, "No, that hasn't been discussed. It's just, the crew just brought her in, and that was that.” So, that was the incident over with. Obviously I had some, sort of, closure. The [day of week], this was a [day of week], [Name of holiday], and then on the [next day] whilst we were packing up, my ex-wife got quite cross at me, 'cause I reported into my [Name of ambulance service], sort of, first responder supervisor at the time, and went over it with him. He was quite concerned that my personal details had been obtained in such a manner, and I'd been treated in such a way. Not just by the security staff, who I guess have a right, you know, especially if they thought I'd been drinking. But also the ambulance staff, with regards to quizzing me, and quizzing me, and taking my personal details. Obviously, now, that would fall under GDPR and things like that, but I said I wanted some, sort of, closure about what was being done with my details. And if [Name of ambulance service] were gonna make a formal complaint. or anything. And he said, "I doubt it'll come to that.” He did feedback to me some weeks later, I had to chase him, 'cause he was just always so busy. He did feedback to me when I chased some weeks later that the station officer in the [location] where it had occurred had undertake, made the crew, specifically that paramedic, undertake a reflection of, you know, public access defibrillators, and ... Not behaviours, but, you know, how members of the public who've tried to help should really be treated, and things like that. And that was the incident over with.

00:12:53

I:	Okay. Well, I’m sorry to hear you had such a, a difficult time, which was, you know, already challenging having to respond to a cardiac arrest. Do you want to continue with this interview? 'Cause I know talking about these things can make you relive it and ...

R:	Yeah, no, that's fine.

I:	... are you okay ...

R:	Yeah.

I:	... to continue?

R:	Yeah, I’ve had worse since. Hmm! Mostly in work. I’m trying to rack my brains if there's been any out of work. That's the most notable one out of work time ...

I:	Okay.

R:	... but the others have all been in work, so, you know, feel free to ask any other questions really.

I:	Okay. Thank you. So, the person who had a cardiac arrest, she was a stranger to you ...

R:	Yes.

I:	... wasn’t she?

R:	Yeah. Absolutely.

I:	Yes. And did you realise what was happening to her at first?

R:	Immediately.

I:	Yes. Yeah.

R:	And once I had that medical history, I drew a working diagnosis of why she had suddenly collapsed like that. And I was able to achieve ROSC quite quickly, following a seizure. This was before the guidelines changed about seizures, actually, that seizures are, could be a very high likely sign of cardiac arrest impending. Especially, well, with regard to epilepsy or not. But as soon as the alcohol withdrawal, you know, I said, "Is there anything she, you know, is she diabetic, is she any, you know, is she got heart problems?” And no, it was just this thing about she suddenly had to stop drinking about four, five days ago.

I:	Okay. Okay. So, how did you ...

R:	'Cause she freely shared it with the team, and they were ...

I:	Yeah.

00:14:27

R:	... the, the, the nurse, the waitress that ran away, sort of, said, "That’s, that’s all I know about her, really.”

I:	Yeah. Sure. And how did you realise it was a cardiac arrest, then? I know you've got this information from them, but how, was there anything else ...

R:	Well, she fell to the floor suddenly. There was a loud crash. She was immediately in a seizure that lasted, I don't know, I think ... I’ve got it recorded. It was less than 30 seconds. And then she changed colour, she was not breathing normally, I couldn't palpate a pulse in her neck, and she was unconscious and floppy.

I:	So, those were pretty good signs …

R:	I started chest compressions.

I:	Yes.

R:	Yeah.

I:	Yeah. Sure. So, you're in a restaurant. There were other customers, presuma ...

R:	All around.

I: 	All around …

R: 	It was [Name of holiday].

I:,	Yes. Yes. But they didn't intervene in any way? Those ...

R:	No.

I:	No. No.

R:	Oh, I lie. One, one chap did come up to defend me, and he turned out to be an off duty firefighter.

I:	Oh, okay.

R:	And he said to the security, in fact, I think, from memory, he ... Gotta remember how long ago this was. He said, "Look,” to the security, before the crew arrived. He said, "Look, we all are being trained to use defibrillators now. You should go and get the defibrillator if this gentleman is asking for it, in case she goes again.” So, yes. And it wasn't full, full, because it was, as I say, mid afternoon, so it was their lull, sort of, from lunchtime. Which is why we booked that time from the lunchtime through to the evening, [Name of holiday] lunch, meal thing, so ...

I:	Okay. Okay.

R:	Yeah.

I:	But none of the other customers ...

R:	There was lots of public there, including my own family, yeah.

I:	But they, they just looked on at this scene, I’m (inaudible)

R:	Yeah, my, my ex-wife took my children away, yeah.

I:	Right. Yeah. Yeah. Okay. So, I’m wondering, when this conflict was going on between you and the security guard, when you asked them to get you the defibrillator, 'cause you said, you know, you know how to use it. Were you doing chest compressions while, you know, dealing with this conflict ...

R:	I believe I was. I'd stopped at that time, and she was in ROSC and I’d had her on her side. And that's why they was disputing it was even a cardiac arrest.

00:16:36

I:	Right. Okay. Okay. So, you'd, you know ...

R:	Which is not uncommon, in my experience.

I:	Okay. And ROSC, sorry, I have come across it before, but I’m not medical ...

R:	Oh, I apologise.

I:	No, it’s fine.

R:	It's a return of spontaneous circulation.

I:	Okay. Yeah.

R:	So, her heart was beating again ...

I:	Heart was, yeah ...

R:	... for itself.

I:	... yeah.

R:	Oh, I remember now, as well, I, I, bear in mind it was a swimming pool, I also asked for any other resus equipment. I don't think she had any injuries. No, she didn't. I didn't bother for first ... *That* was right! I asked for any other equipment like, "Can you bring oxygen?” Or stuff like that, and they refused to bring me the oxygen. And I suppose she might need some oxygen. They refused to bring the oxygen. I’m pretty sure they refused to bring oxygen, or it came very late and the crew were already there, or something. But I remember they did slap a first aid kit in front of me (chuckles slightly). That was it.

I:	Okay. Okay. So, you were experiencing a lot of barriers to getting the equipment ...

R:	Oh, absolutely.

I:	... that you needed.

R:	Couldn't have been worse, really.

I:	Yes, which must have added to ...

R:	Apart from pushing me away, and, sort of, taking over, and, you know.

I:	Yeah.

R:	Yeah, it could, it could have gone a lot, lot better from their point of view.

I:	Yes. Yes. So, you're dealing ...

R:	And I, I believe I did complain to the managers, or the owners of the [holiday location] as well, for them to reflect and for further training. And I never had a response.

I:	No. No. Okay. Okay.

R:	Obviously, things have changed greatly with defibs since then.

I:	Yes. Yeah. It sounds very difficult, very challenging, on top of what already was a challenging and unexpected situation. So, can you tell me as much as you can about the 999 call?

R:	I don't think, I, I think ... I’m quite happy to look up my notes, I’ve got it online somewhere, but ... I, I should have refreshed my memory, I’m sorry.

00:18:33

I:	It’s okay.

R:	I’m pretty, I think they made me speak to the control, actually.

I:	Who?

R:	'Cause they, sort of, said, "Well, you talked to the security,” I think they said. While we were waiting for the ambulance, I said, "Well, can I speak to them? I wanna update them.” And I handed everything over. From memory, I’m pretty sure I spoke to control.

I:	Okay. So, so, who initiated the 999 call? Was it security ...

R:	They did, to start with.

I:	The security team?

R:	Yeah. Or, or the waitress ...

I:	The waitress.

R:	... or whatever, but I think it was, I, I think the waitress panicked, and she just called security. Which was probably their standard operating procedure for a medical emergency, I guess.

I:	Okay. So, you didn't tell them, they'd done that of their own volition, they, they’d made that call.

R:	Yeah. I, obviously I said, I shouted out ...

I:	Yeah.

R:	“Could someone call an ambulance?”

I:	But they'd already ...

R:	Yeah.

I:	... they’d already done it.

R:	I think she'd ran off and called security ...

I:	Yeah.

R:	... and I think they did it, as they were ...

I:	Yeah.

R:	... sort of, yeah.

I:	Okay. And do you know if they used a landline or a mobile?

R:	I have no idea.

I:	No.

R:	Mobiles would have been around then. I have no idea.

I:	Yes. Yeah. And do you remember what the call handler said to the security?

R:	It was mostly me updating them about my findings and my actions.

I:	Over the phone?

00:19:45

R:	And they ... Yes, yeah. And they said that they, they were no problem at all, from memory. They were, like supportive, and, like, "Well, the crew will be with you shortly. Do you want us to stay on the line?” “No, I’m okay, thanks. I think she's in ROSC, she’s in ROSC, I’ve got her on her side.” And, and of course, there was this to and fro from the staff, so I was trying to deal with them still. So, it, it wasn't a prolonged call, or supported call from [Name of ambulance service]. It was a quick update for the crew ...

I:	Okay.

R:	... via, via the call handler.

I:	And how did you get the phone, then it must have been a, well, it, someone must have given you the phone? To speak, or ...

R:	Yes ...

I:	Yeah.

R:	... I think it was the, I think it was one, it wasn't my mobile, it was the phone they were using for the call from security.

I:	Yes, okay.

R:	It was a mobile, yeah.

I:	It was a mobile, okay.

R:	Yeah.

I:	And can you remember what help the call handler offered you? Or, or any ...

R:	None at all. It was more an update of what I'd done, and, sort of, a live update.

I:	Why do you think they didn't give you any help, or ...?

R:	I, probably 'cause I identified myself as a nurse again, and it was sorted. The patient was in recovery. I needed to stay with her. I think we put a coat under her head, or I think they brought a blanket, something useless to put under it. Not useless, but not what I needed. I wanted oxygen and the defib in case she re-arrested, really. But there was no issue for me with, you know, them not staying on the line. They probably offered, and I said, "No, it's, I’m good thanks, I wanna get on with the patient.”

I:	Yeah. Sure. Okay. And can you remember if the call handler asked you any questions?

R:	Yeah. Obviously, just the standard, you know, "Is she conscious, breathing?” That sort of thing. And I explained what had happened. 'Cause they already had the call in remember, so ...

I:	Yes. Yeah. Okay. And did you ...

R:	Yeah.

I:	... ask the call handler any questions?

R:	No, I didn't need to.

I:	Because of your medical training?

R:	Oh, I did obviously say, "Do you know how long they're gonna be?”

I:	Yes.

00:21:53

R:	Yeah.

I:	Yeah. Okay.

R:	Yeah.

I:	But you didn't need any medical assistance with how to do the chest compressions ...

R:	No.

I:	No. Nothing ...

R:	No, not at all. I’m competent.

I:	Okay. And what are your reflections on that interaction with the call handler?

R:	Oh, with the call handler, you mean? …

I:	... Yeah, with the call handler. Yeah.

R:	None, really. It was supportive. I, I obviously ... I think at the time they weren't offering ETAs, so they said, "They'll be with you as quick as they can. Can you make sure, sure someone’s out at the front.” And I said, "I think there's already another security guard out there.” So, I think it was a team of three, obviously it’s a massive [holiday site]  so I know, I know the two security guards that were present with me, but I, I believe there must have been a third out the front. Because he was, he, she was the one that brought the crew to the site of the incident, in the middle of the restaurant. So, yeah, no, I’m, I’m not, nothing to say really about them. I didn't make the initial 999, but the one I did to update, there was no problems at all.

I:	Okay. And while you're updating the call handle on the phone, did you remain with the patient, or did you ...

R:	Absolutely.

I:	The, the whole time, you were ...

R:	Yeah. Yeah. Oh, yeah.

I:	Yes. Yeah. But when the call was initiated, presumably the security team had, were, were doing that away from the patient? Or were they with the pa ...

R:	Yes, yeah. Yeah, that's one of the things I think I fed back to the area, to the national CFR officer, who was gonna feed it back to the area ambulance officer. I think, I can't remember if they said that [Name of ambulance service] had done the training with the camp locally, or someone had ... But I said, you know, "A part of the training thing, you know, the security, if they're gonna respond with medical equipment, they should at least respond to the patient to see what's going on before they make any, sort of, 999. 'Cause it could be a simple faint, or syncope. And their patient is back round, and we've got a blue light ambulance coming, thinking it's a cardiac arrest.” You know, they didn't, they didn't make, they'd already made the call, and then responded to me.

I:	Right. Okay. And then you confirmed to the call handler that it was a cardiac arrest?

R:	Yes.

I:	Yeah. Yeah. Okay. And was ...

R:	(inaudible)

I:	... were, was there anything that was difficult when you were talking on the phone to the call handler?

00:24:12

R:	Nothing at all.

I:	No. No.

R:	From memory, nothing at all.

I:	No. Okay. That's fine, and, and you ...

R:	I think I did, I did relay that it had been a ... Obviously, they were stood in front of me, but I, I did say it'd been a difficult situation with regards to response. But obviously, in my head, I knew that they were lay people, and that even some of this trained security probably might never have seen such an incident before, despite their training.

I:	Yeah. Sure. And, and did you have any technical issues while talking on the phone?

R:	Oh, the one thing I did remember, and I think this is in my reflection when I wrote it up, was that I remember trying to whisper very quietly to the call handler about the history with the alcohol. 'Cause I wanted to hand that over, about why ROSC might have occurred suddenly with an opening airway, and then CPR, and, you know, immediate action of clearing the airway. And obviously immediate action brought ROSC back. I mean, she might, obviously I’ve reflected she might not have been in a cardiac arrest, she might have just dropped her heart rate down very, very low. But as far as I was concerned, and I remain concerned, and will defend myself, is that I followed the guidance for community CPR. And then, on top of that, as a clinician I checked her pulse as well, and I couldn't feel one in 10 seconds. So, I’ve got, not got guilty conscience. But yeah, with the call handler, I do remember trying to whisper to, you know ...

I:	Yes.

R:	... to maintain some, sort of, confidentiality for the poor lady, who was coming around a bit, you know, by then. So ...

I:	And do you think the call handler heard you, or were, were you (inaudible)

R:	Oh, yes.

I:	Yes.

R:	Yeah.

I:	They did.

R:	Yeah. Definitely. Yeah.

I:	They did. Yeah.

R:	Yeah.

I:	Okay.

R:	Yeah.

I:	And did you leave the patient to let the ambulance crew into the [holiday site], did you ...

R:	No.

I:	Oh, that was the security.

R:	I stayed with the patient.

I:	You stayed with the patient, and the security team let them in, and, or showed them where to go, is that ...?

00:26:11

R:	Yeah, and the ambulance was brought up to the, through ... Obviously, 'cause it's a pedestrianised area, so the ambulance crew was brought up to the front between the swimming pool and the pedestrianised area, and the grill. Yeah.

I:	Yes. Yeah. And you were with her ...

R:	We loaded her up there and then, yeah.

I:	Yeah. Yeah. Okay. I just wanted to check whether you left ...

R:	So, that, that part was all fine.

I:	Yes. Okay. And you've touched upon this already, how you performed the CPR. Can you tell me anything else about how you delivered CPR on that day, please?

R:	I weren’t really keen on mouth to mouth, (chuckles slightly) because of the frothing and, and everything. And I didn't know her, and I think if the arrest had continued, I would have attempted. But that's why I was calling out for all the equipment. I presumed, I, I understand the guidance has changed with swimming pools, I don't know if they're allowed oxygen anymore. But I knew they had oxygen at the time, and I wanted at least a bag valve mask, or a pocket mask, or something with the, with the defib, that would have been kept with the defib, so ...

I:	Right. Okay. So, you did chest ...

R:	... in case she went again, I could at least have carried on chest compressions, and put the pads on. And, you know, cut her clothing, put the pads on her, at least had something to start proper CPR ventilations and compressions.

I:	Yeah. Sure.

R:	But then, my, 'cause she was still a terrible colour. And I remember saying she was quite jaundiced actually, in my reflection. That I remember thinking that, "She's gonna go again, I need to be putting this lady on oxygen.” 'Cause obviously there's no sats probe, so I had her on her side, and was just concerned of her colour. And that perhaps she did need some post resus oxygen at least.

I:	Right. Okay. Okay. So, so you did chest compressions only?

R:	Chest compressions only.

I:	Yeah.

R:	After checking airway breathing, yeah.

I:	And what are your reflections on how that went?

R:	Fine. I got her back.

Interviewer and responder chuckle.

I:	Yes.

R:	I think she was back within the 30. I didn't need ... That, that was the other reason I think, obviously, the guidelines ... Yeah, she was back within the 30, 'cause I think I was gonna go for it with regards to ... I wasn't keen, but if the equipment hadn’t arrived, I probably would have done 30 to two.

I:	30 to two ... What do you mean ...?

00:28:27

R:	30 times, sorry, 30 compressions to two breaths.

I:	Okay. Okay.

R:	If, if she hadn't ROSC’d. Yeah.

I:	Okay. So, I was wondering, like, the call handler, did they give you any instructions? I know you’ve said they ...

R:	No, I wasn’t on the phone to them.

I:	You, when you were doing the chest ...

R:	Remember, I didn’t make the call.

I:	You didn’t, no, but when you were giving the update, you weren't doing the chest compressions?

R:	Oh, no, no, no.

I:	No. No.

R:	It was all over, I had her in recovery by then.

I:	Oh, okay. Yes, and you were ...

R:	She came back very quickly.

I:	Yes. And you felt confident to do the chest compressions on your own, because, of course ... Yeah.

R:	Absolutely. (Chuckles slightly).

I:	Yeah.

R:	It’s my job. (Chuckles slightly).

I:	Yeah. Yeah. So, you didn't have anyone around telling you what to do, either.

R:	No. As I say, the firefighter ...

I:	No.

R:	... came to defend my actions. I think when he saw the crew he disappeared. Yeah. But he, he defended me, and came up, and, sort of ... He, 'cause he, he was hanging around, and he didn't interfere. But when he saw I was having a hard time from security, he came over to, sort of, put them straight.

I:	And how did that ...

R:	And ask me if I needed anything. Yeah.

I:	Yeah. How did that make you feel, going ...

R:	Brilliant.

I:	Yeah.

R:	I, I, yeah.

I:	Yeah.

00:29:32

R:	I felt vindicated. But (?) I'd already handed my out of date [Name] badge over by that time. You know, they, for what it’s worth, they didn't even notice it was out of date. It was, like, it was an old one. We didn’t, we were online, we, registering online by then, I’m sure. And yeah, I, I just don't know. I dunno who'd done their training, and I ... See, I know the crew had to reflect, but it was difficult.

I:	It was difficult for you having to experience that conflict.

R:	It was upsetting, it was upsetting *enough*. And then, on my birthday and on [Name of holiday], to do that in front of my little kids, and then to be criticised twice. And then worry all night that, you know, you're gonna be phoned by a consultant in (?) ED to be questioned further about why you've done what you've done. It was hard.

I:	Yes. Goodness. Yeah. I mean, (inaudible)

R:	I'd like to say times have changed. I would like to say that. I, I, I hope times have changed.

I:	Yes. And how did you feel when you first witnessed the cardiac arrest, before all the conflict started.

R:	I just ... How did I feel? I just went into it. I, I remember being a bit loud. The one thing that I’ve always, sort of, thought ... In fact, I’ve raised this before, and I use it on my teaching, is that your competence with regard to if you've been drinking, or for some people that might mean substances. My experience that day was, was I wasn't drunk, drunk, but I sobered up very quickly. (Chuckles slightly). And in fact, I remember actually, the bar manager [Name of bar manager] said, "Do you want any food? Do, do you and your family ...” This was one positive. They said, "Do you want food?” And I said, I think my ex-wife had gone by then, and she said the kids had, I, she'd sorted the kids out with food. And I said, "I certainly don't, I’m not feeling up to it now.” But he said, "Do you wanna drink?” And I said, "Yeah, I'll have a drink, please!”

I:	(Chuckles slightly) yeah.

R:	So, I had a drink. And yeah, the family were back with me by then, so I think they had a, you know, free drink as well. But they said, "Do you need anything?” I said, I nearly said, "Yeah, retrain your staff,” but I didn't.

I:	Okay.

R:	So, that was a positive. But what I feel is, and what needs to probably, there's never been a test case, but what I feel is it probably, I think if you sober up quickly, and you know you're doing it and it’s something very basic ... If you're completely inebriated and you can't walk, you can't help anyone, and you would be remiss to do so. And there's a bit of a difference between doing basic CPR, putting some pads on and pressing a button, and trying to put a line in someone a cannula, or a, give a, prescribe a drug, or something like that. So, I think, to me, it's an interesting ethical debate, probably for further research about responders who are health professionals, or anybody who's had training that responds on a night out, or ...

I:	Yes. Yeah.

R:	You know, what are you gonna do? Say, "No, I’m not gonna help because I’ve had a few?” Or are you gonna say, "I’m gonna try and save this life to the best of my ability, but only act within my current competence, my current capacity.” But my experience that day was there was *no* issue at all, I knew what I was doing.

I:	Yes. Yes. Okay. And did you end up using a defibrillator?

00:33:06

R:	No.

I:	No. Okay.

R:	It was all over, and they brought it to me way too late. As I say, I think they, it was, I think it was only when the fireman intervene, firefighter intervened that they (inaudible) and went to go and get it.

I:	Okay. So ...

R:	As I say, I think I was brought the blanket first, then the first aid kit, and then the defib came.

I:	Okay.

R:	That's when it was too late.

I:	So, is that why you think a defibrillator was not used, because it was brought to you too late?

R:	I think, from memory, it only arrived as the crew did. So it’s pointless. 'Cause they brought theirs.

I:	And did they use it on her, or didn't ...

R:	No.

I:	... need to at that point?

R:	Oh, well, no, they, no, they didn't. I think they monitored her (inaudible). I remember as well ... Oh, yes! Now I’m remembering. And this is in my statement. I automatically, and this, this is what showed I had mental capacity, despite a few drinks before lunch. Is, I remember going into automatic mode, and putting the BP cuff on the lady, which is something I would do as a nurse, like, anyway, and as a team leader anyway. And I remember it being snatched from me.

I:	Oh dear. Yeah. Okay. And so ...

R:	And I sort of went, "Oh, sorry, sorry.” Like that, that just, like, in automatic mode. I’m sure it's a BP. It might have been the sats, but I’m sure it was doing her blood pressure.

I:	And is that when ...

R:	And it was taken from me by the crew.

I:	And that was while she was unconscious?

R:	She was coming around ...

I:	She was coming around, okay.

R:	... by that stage, but obviously they were doing a set of obs straight away.

I:	Okay. And so, the defibrillator finally, eventually was retrieved, and brought to you ...

R:	I think it arrived, like, I dunno, 30 seconds before, or round about the same time as the crew. So, it was pointless calling for it, basically. Well, it wasn't, but do you know what I mean, it was ...

I:	Yeah. Yeah.

R:	... it was, it arrived very late.

I:	And it was brought to you by the security team?

00:34:59

R:	Yeah.

I:	Yeah.

R:	On the intervention of this firefighter, yeah, who was off duty.

I:	Okay. And the reason that it was brought to you late was because they had doubts about your professional ...

R:	Competence ...

I:	... background, and competence.

R:	... and the public shouldn't be asking for it.

I:	Right.

R:	That the lady seemed okay, that they were questioning my diagnosis of seizure followed by cardiac arrest from alcohol detox, whatever.

I:	Was there another defibrillator, maybe closer to you that you hadn't realised at the time that you subsequently remembered afterwards, or, or ...

R:	No.

I:	No. That was the only one.

R:	I think that was the only one on the main drag of the camp there.

I:	Yes. Yes. Okay. And presumably you didn't want to go and get it yourself, because you wanted to remain with the patient at all times.

R:	Absolutely.

I:	Yes.

R:	Absolutely.

I:	Yeah. Okay. And did ...

R:	Following the chain of survival.

I:	Yes, which is, you're taught to stay with the patient, is that correct?

R:	In case she went again, I didn't wanna leave her with anyone else who didn't seem to know what they were doing.

I:	Yes, absolutely. Sure. And did the call handler, while you were giving your update to them, did they advise you of a defibrillator, nearby defibrillator, or did they ...

R:	No.

I:	No, it wasn't mentioned.

R:	No, not back then. They didn't, no.

I:	Do you know if defibrillation was mentioned to the security team?

R:	No.

I:	No, you don’t …

R: 	I don't know …

I: 	… know that. Okay and what are your thoughts about using a defibrillator in the future?

R:	As a lay public, or as a, as a responder, or just as a person?

00:36:34

I:	As a lay member of the public.

R:	Oh, I have no, I’ve campaigned long and hard that they're available, so I have no qualms doing it.

I:	Yes. Yeah. And is there something that would make it easier for you to use a defibrillator in the future?

R:	Increase knowledge and understanding. Yeah, just awareness with the general public, and an awareness within the ambulance service. You know, I think anybody that handles calls that come in have to realise ... And, and responding crews, and it's, this isn't against anyone in the ambulance service, we see it in the hospitals as well. You know, you never ever go to someone and say, "Well, that wasn't an arrest.” You weren't there, you don't know. You don't know how they looked, you, you, you know, you don't know how ... This is what so upset me about my details being taken, you know, that I was gonna get some, sort of, stroppy call from an ITU consultant or something. Like, if she's got broken ribs, "What did you do that for?” And that was the big inference from the crew was about, 'cause I remember now, not only was her colour off, like, I think she was probably jaundiced, was that also she was *tiny*, you know. And obviously I’m a big bloke, so ... (Sighs heavily) this, this thing hanging over my head that, you know, "My god, I have injured her, but I injured her for a reason, in her best interests. And yet I’m being called into question.” *Nobody* should criticise anybody for trying to save someone's life, you know. Like, genuinely, like, I’m not talking about letting someone walk around an accident with a broken neck, I’m not talking about that, or moving someone unnecessarily. I’m talking about if someone is down in front of you, they've got sudden unconsciousness, and they are not breathing right, you know, and they're following the guidelines. Nobody should turn up and go, "Well, that wasn't a cardiac arrest. That was just a faint.” You weren't there.

I:	Yes. Sure.

R:	That's what I feel.

I:	Absolutely.

R:	And I feel defibrillator downloads should be used way more commonly than they are. Like, post incident defibrillator downloads from the memories. For follow ups for consultants, or coroners, or whatever. Medical examiners. To establish the facts of what put this person on the floor ...

I:	Yes.

R:	... and match that with patients’ history from responders, from the public, from the family. And then, until we've done that, I don't think we can quote to people that it wasn't a cardiac arrest.

I:	Okay.

R:	It just upsets them even more then, because they feel they've done the wrong thing.

I:	Yeah. Yeah. It makes them doubt themselves. I know ...

R:	And that was me, and that was, I’m an instructor and I felt that.

I:	Yeah.

R:	So, if you said that to a member of the public, say in a factory, who was doing it on their colleague, they might *never* wanna do CPR again ...

I:	Yeah.

R:	... just from that little comment.

00:39:33

I:	Yeah.

R:	“But I did it wrong last time ...”

I:	“So I’m not gonna ...”

R:	“... I didn't get it right.”

I:	Yeah.

R:	You know, "And he was alright, so it wasn't a cardiac arrest.”

I:	Yes. Okay. We are running a ...

R:	Yeah. No, it’s fine.

I:	... little bit behind schedule but I, I will just give you the opportunity if you did want to mention other experiences of assisting with a cardiac arrest.

R:	No. It's funny, I’ve recalled more just talking about it. It's funny.

I:	That's, that’s fine. And, 'cause, like you say, you've had lots of experience of assisting with a cardiac arrest in a lay capacity, may I just ask why you chose that particular case?

R:	'Cause it was, it was out of work.

I:	It, yeah.

R:	You, you were looking for lay capacity.

I:	Yeah. Yeah.

R:	The others, I’ve responded from my NHS role, or as a CFR for the ambulance service ...

I:	Okay.

R:	... or on medical covers, or whatever. No, this was, and I, obviously it's memorable. It was my birthday, and it was [Name of holiday] ...

I:	Yeah. Yeah.

R:	So, yeah.

I:	Okay. Excellent. Thank you for sharing those experiences with me today.

R:	That's okay. I hope it helps.

I:	Oh, yeah ...

R:	I'd be very interested to read the study, it’s very, very interesting.

I:	Yes, no, fascinating insights you've given us, so thank you. If it's okay with you, can we move on to the next part of the interview? I know I’m taking up a lot of your time.

R:	No, it's okay.

I:	Is that, are you sure ...

R:	I allocated time for this, so ...

I:	Okay, excellent. Thanks. So is drone delivery of defibrillators something that you've heard of?

00:41:00

R:	Yeah.

I:	Okay. Excellent. So, you've heard of drones, presumably.

R:	Yes!

I:	Yeah.

R:	If we're using them for bombing people, we should be using them for delivering life saving equipment.

I:	Yeah. Yeah. So, you've seen drones used, or, or used one yourself or?

R:	I’ve, I’ve never seen one. I’ve seen them, like, hovering for rugby matches and things, for the police, but I’ve never seen one deliver a defib, or ...

I:	No. No.

R:	... a haemorrhage kit, or whatever like that. Obviously, I’ve seen them for surveillance. I’ve never used one, no.

I:	No. No.

R:	I know they're simple to use.

I:	Okay. So, drone delivered defibrillation isn't currently in operation in the UK ...

R:	Yeah.

I:	... so, but it is in [Name of country], which is interesting.

R:	Yes.

I:	Which is why we ...

R:	I was aware of that ...

I:	Yeah.

R:	... research, yeah.

I:	So, you've said that you've heard of drone delivery of defibrillators. So, can I ask where you've heard about it, and how would you imagine it works?

R:	Through, through my research, through my work with [Name of ambulance service]. It was in a [Name of ambulance service] paper here in the control centre, about the work of [Name of academic]. I know that he's done a previous study on it. I’ve seen the articles and the news reports from [Name of country]. I know that, think I’ve referenced it in one of my papers about the [Name of organisation] type droid delivery robots along the road. That's also a good idea. Obviously I don't think that'll be as quick as drones, but yes, I’m, I’m, I’m aware of the work going on, yeah.

I:	And how do you think it works, or do you not have an idea?

R:	Well, I presume it … what how do I think it would work, or ...

I:	How, yeah, how ...

R:	... how do I think it works?

I:	... do you think it would work?

R:	From control, I guess, the same way as a military coordinator coordinates the drone from their base. With regard to the defibrillator, going out probably from somewhere central or regionally to the nearest arrest. And probably will reach the scene quicker than an ambulance.

00:43:05

I:	Yes. Yes. And also, well as you're aware, drones can carry defibrillators. They can land ...

R:	Yeah.

I:	... and detach the AED, or they can ...

R:	Yeah ...

I:	... hover.

R:	... I’ve seen the pictures, yeah ...

I:	Have you?

R:	... looks fantastic.

I:	Yeah.

R:	I, I, I think, the point being, I think it will probably, I think we've probably got community access defibrillators wrong. I think there's evidence from the [Name of organisation] that shows that they're not being utilised as we thought they would. A lot of time and money, especially in [Name of UK nation], the [Name of government] have supported it. Obviously, it’s set up [Name of government programme], same as [Name of government programme]. And I’m not sure ... We're doing summit wrong with it, if it’s, if only 5% of them are being used, and they're almost on every street corner in a lot of cities and towns in [Name of UK nation], or anywhere. And I think getting a defib … And it'll be a hell, I think it might be hell of a lot cheaper to run a drone scheme or something similar, along with first responder schemes from the community themselves, rather than putting defibs everywhere. I’ve changed my view on that massively in the last 10 years.

I:	So, what, why ...

R:	I think drones are probably the way to go.

I:	Why do you think people aren't accessing them, if they're on, like say, every street corner?

R:	You want my hypothesis? Because I think people are following the chain of survival now. Because the advice has changed probably since that call that I put in all those years ago, and I think the evidence shows that the majority of people that are in their own home, certainly in [Name of UK nation], they're of a certain cohort, of age group. So, they're probably with their partner, often husband and wife. Not always, or son and daughter, or whatever and I think that person is undertaking life support. There is no third person to go and get the defib up from up the shop, or from the school, or the leisure centre. They're doing CPR, or, in my experience, they're pacing the room panicking.

I:	Yes. Yes. Okay. And we'll come on to talking about leaving the patient to get the defib from the drone in a moment ...

R:	Right.

I:	But, 'cause I think that links to what you've, you've just said. But just, can I ask you what you think about interacting with the drone, in terms of it landing and detaching the AED, or hovering above the ground and winching the AED down to the ground, via a cable, or a rope, or winch like device ...

00:45:40

R:	Oh, (chuckles slightly) that’s a good one!

I:	What do you think about that?

R:	I thought they only detached on the ground, from what I’ve seen. But, yeah, it's all so high tech, innit. ‘Cause (?) [Name of public figure] doing what he does ... And I think probably on the ground, I think there might be some technological fear. We have to remember that with defibs and things, and the teaching in schools, kids are growing up with these, same way they've grown up with mobile phones. So, the future generations they will not be, in their Star Wars, sci fi heads, they, it won’t be unusual for them if it's winched down to them, or it comes on a robot, or it comes, you know, it won't matter in the future. That might save us, 'cause it's our grandchildren or children that will save us. For now, from the research, original paper I did for my masters, over a certain age group there's massive fear of technology, and I think that research needs to be repeated. It was only a small pilot, but it needs to be repeated, ‘cause some of our fear might have gone in the older generation now. And I evidenced it was generally more females fearful of technology, but ... And obviously certain age groups of older generations are using iPads and things, and they go and get their [Name of organisation] from a robot, or an [Name of organisation], or whatever, do you see what I mean. So, that might have changed, that needs further work. But I think future wise, I think ... And, and remember we'll probably have them on our phones anyway, before that.

I:	Have what, sorry, on our phones?

R:	A defibrillator on your phone.

I:	Oh, wow.

R:	You can already do your own ECG on your watch, or whatever. I don't know how I feel about that. If it saves someone's life great, and they spot they've gone into AF great. But generally, I think it does set worries into people's minds that may not be there. But if it saves one life it's worth it, innit. But I think, yeah, the technology will grow, and the public will get used to it, however you manage drones.

I:	So, do you think the public's ...

R:	It’s very exciting, obviously, I think a lot of younger generation may be very excited. And you'll find if an elderly, older generation or a community saves a life with the drone, they'll probably fundraise for them. That's how it works.

I:	But do you think people's fearfulness around the technology, around drone delivered defibrillation, could be a barrier to uptake and use? Is that what you're saying? Or ...

R:	No.

I:	No. You don't think that.

R:	I think, I think there's more, I think there's probably more fear of leaving the person.

I:	Ah! Okay.

R:	Even if the, even if they're pacing the room or just holding their hand, I think there's probably more fear of leaving their loved one. This, this is from a predominant, where the majority of cardiac arrests occur, I’m not talking about in the public or whatever, but the majority, in, in homes, I think they won't leave the person to get the defib ...

I:	Why do you think ...

00:48:37

R:	... very often.

I:	Why do you think that?

R:	'Cause they're on their own. 'Cause the majority of cases happen with couples on their own, or whatever. Where you've got a family involved, or a kindly neighbour involved, or a family member that lives over the road that can dash over, I think that's when you see defibs going to get fetched.

I:	When there's more than one person being a bystander (inaudible) …

R:	And you've got to remember that person that turns up the second time, who may be an off duty firefighter, or school teacher who's done CPR, or whatever. They then get on with CPR, 'cause the other person might either be tired, or they may not be doing it.

I:	Yes. Yeah.

R:	So, you're, you're back, you're back to that square one. Now, if that was me, I'd send the person that was tired or scared to go and get a defib, to do something useful while I did CPR. But that's the, that's the dilemma, is ... So, what I’ve always said, it's gotta be a community approach.

I:	And what do you mean? …

R:	You, you've gotta get that neighbour to go and get it. But you may not speak to that neighbour. You may not speak to that neighbour, but you're doing CPR on ‘em.

I:	And, and this might sound silly, but how are you supposed to go to get the neighbour if you're in different houses? Do you see what ...

R:	Well, exactly. You shout, and you shout, and you shout. Shout for help. It literally says it. And we need to differentiate between shout for help and call for help. We shouldn't be using call for help, we should be saying, "Shout out for help, and telephone 999.” That's what we should be saying. Two different things.

I:	And do you think this community approach would be relevant, suitable for a drone delivered defibrillation model?

R:	Obviously. Because if, if it lands near the shopping, shopping centres probably always gonna have defibs now, aren't they. Rugby clubs, whatever, they're always gonna have a defib there, like, on the wall. But for communities, if it then lands in the middle of the pavement, then that neighbour can bring it in. Or that son or daughter can go out and get it, or a child could go out and get it and bring it in for you.

I:	Yeah.

R:	So, definitely.

I:	Yes ...

R:	Rather than running up to the police station, which could be quarter of a mile away, and run back. Ha!

I:	Yes. Difficult, yeah. So, how would you feel about a drone bringing a defibrillator to you whilst helping someone having a cardiac arrest?

R:	Until they're on mobile phones and we got them in our own homes, then yes, drones have gotta happen. I, I, I’m not sure how I feel about them going along the road in a little droid, or something, like an [Name of organisation] delivery. (Breathes out) that just worries me with the risks, you know. I wouldn't, you know, we know from military experience drones are working amazing, you know what I mean. (Chuckles). Drones are doing the job that they are wanted to do, whereas roadside might be difficult.

00:51:23

I:	Okay.

R:	I think drones are the way to go until we've got them in our own possession ...

I:	Okay.

R:	... on our phones.

I:	And you think that because a drone delivered defibrillation model is more amenable to this community approach that you refer to, is that ...

R:	Yes.

I:	Yeah. Okay.

R:	Without a doubt. Un, unless it's in the middle of, say [Name of shopping centre] in [Name of area], where there’s hundreds of people around, and you can't fly the drone into the middle of the shopping centre.

I:	Safety implications.

R:	I don't know, maybe you can. I don't know drone stuff, but.

I:	Okay. Okay. And would it make it easier or more difficult for you to use a defibrillator?

R:	The drone? Oh ...

I:	Yeah.

R:	... easier.

I:	Yes. Yeah. Why …

R:	Much …

I: 	Do you think ...

R: 	 Much easier.

I:	... why do you think ...

R:	'Cause it comes to you, rather than you ‘aving to go to it in a crisis situation.

I:	Yes. Yes. And you've touched upon this a lot actually, but how would you feel about leaving the person if they are by themselves? So, imagine you haven't got this community support, you're not in the middle of a shopping centre. You are on your home, in your, yeah, on your own, say in your own house. Somebody ...

R:	Right, so ...

I:	... collapses, what do you do?

R:	... so, outside of the shopping centre scenario, where you're in an enclosed environment, right, and you've got security and everything. I think if it had happened on that day, in a different universe, I'd have been happy, 'cause I'd have been happy to leave her, say with a firefighter, or my ex-wife, or something for 30 seconds to run outside, and get the ambulance delivered drone and come back in, and then still look after her, than go through what I went through again, right. And as I say, I reiterate times have probably changed, and I hope that never happens to anyone else. With, with regards to the home, absolutely even better. Because if it's landed on my lawn, or landed on my driveway, nobody's gonna balk at stopping CPR for, like, 20 seconds to go and get a defib to link, instigate link number three in the chain.

00:53:14

I:	Okay. And how far would you go to get the defibrillator then, that had just been delivered by drone?

R:	It needs to be, if I was ... It depends if anyone's able to help with CPR, doesn't it.

I:	If they're not, say. If, if it’s just you …

R:	If it was me on my own …

I: 	Yes. Yeah.

R:               Like, it would *have* to land, like, literally outside. You, you know, you wanna be less than 30 seconds. You don't wanna be off that chest for long, do you. So ...

I:	No. No. Okay. And what about if it happened outdoors? Would your thinking on this change?

R:	What, like on a rugby pitch, or something like that ...

I:	Yeah.

R:	... or ...

I:	Public space. A, a restaurant, a rugby pitch, shopping centre ...

R:	Well, even better. Yeah, even better. 'Cause there’s bound to be people that will offer you help, then.

I:	Yes. Okay. Okay. So, and like you say, if there was another person there they could get the defib, and you could continue with the chest compressions.

R:	Yeah. So, a lot of the research on public access defibs has been done in high footfall public areas, hasn't it, you know. And then, we need to focus on where the majority of them happen, especially where we think the majority of (?) happening in [Name of UK nation], which is their own home.

I:	Yes. Yeah.

R:	And, and you, *unless* you've got that third person, second person ...

I:	It's tricky.

R: 	So, and, on a, in a rugby pitch, you know, you're gonna have someone there. So, ten, I think 10% of people in a big, sort of, space, around 10% of people are, like, at professional, at any one time. So, there’s bound to be someone. Even in that terrible situation I had, there was a firefighter who could have helped, and gone to get the drone, or whatever. He would have gone to get the defib, but we had all these barriers ...

I:	Yes.

R:	... with the security.

I:	Yes. Yeah. Sure. And how would you feel if you were on your own, and you were having to say run downstairs to get the defib?

R:	Fine.

I:	You would, you'd be …

R: 	Fine.

I: 	You'd feel comfortable ...

R:	Yeah.

I:	... with that?

R:	Yeah.

00:54:57

I:	Yeah.

R:	'Cause link number three counts.

I:	Yes. And same if you were outside, and you were dealing with this on your own? Say, in a rural …

R: 	Same …

I: 	Location ...

R:	Doesn't ...

I:	Yeah.

R:	... doesn't change.

I:	Yes. But you think it would be ...

R:	Yeah ...

I:	Sorry, go on.

R:	In fact, if you think about it, the drone will get closer to you, potentially, with a camera in the sky, like you see on the military stuff on the TV. The operator will get it nearer to you than you would be to a cabinet. (Chuckles slightly).

I:	A cabinet, you (trails off) oh yeah!

R:	A cabinet with a defib, yeah.

I:	Yes. Yeah. Yeah. So, it ...

R:	A drone can drop right to you, can't it?

I:	Potentially. Yes, well, I mean, not through the window ...

R:	Potentially.

I:	Yeah.

R:	Potentially.

I:	Potentially on your doorstep, or over the road ...

R:	Or if you were outside, right next to you, if it's got a camera on (?) it (?), you know ...

I:	Potentially, but also there could be safety implications, to not have the propellers whizzing too close to you, so ...

R:	Oh, of course, of course. You know what I mean ...

I:	This is what ...

R:	Yes. Of course.

I:	... this is why …

R:	... Yeah. Not not …

I:                    I’m asking ...

R:                   Near a wire, or a ...

I:	Yeah.

R:	... overhead. (Chuckles slightly). Yeah.

I:	Yeah. Not to get whacked round the face with a, you know, the ...

00:55:56

R:	But even if you've gotta run, like, 50 yards up the road ...

I:	Yes.

R:	... to go and get it, 'cause of safety concerns, that’s still quicker than running halfway across a shopping centre, isn't it.

I:	Yes.

R:	Do you know what I mean.

I:	Yes. Yes. But yeah, I, I just wanted to explore your thoughts about leaving the patient, because we've had, you know, mixed, mixed views on this, and it's interesting ...

R:	Well, we know how quickly VF deteriorates, so if that CPR’s not working ... Fortunately it did in this lady's case, but if that CPR’s not working, you've got to defibrillate, and you've got to defibrillate quick.

I:	Yes. Yeah, absolutely. Yeah. And ...

R:	If, if they're in VF, obviously.

I:	And this can be one way to enable early defibrillation.

R:	It comes to you rather than the other way around.

I:	Yes, yes, yes. What do you think the role of the call handler is in all this?

R:	Well, for the general public who don't know, or who need, who've had CPR training and need a refresher, it's talking them through, isn't it. And, like, "Help is on its way. We've got an ambulance crew,” or, "We got, we've got air ambulance coming with a doctor on.” And, "We've got a paramedics to come in, and, "You've got a paramedic coming in an RRV, we don't know how long they're gonna be, but they’re coming. But there's a drone on its way as well.”

I:	Yes. And do ...

R:	“Have you got someone to get the defib?”

I:	Do you think that you'd be more or less willing to leave the patient if instructed to do so by the call handler, in that situation?

R:	If they know it's gonna land right near ‘em, I think they would.

I:	You ... And would you as well?

R:	Even if they're alone, yeah.

I:	Yeah, you would be, yes. Yeah. Yeah. Okay.

R:	It’ll need a massive public campaign, obviously.

I:	For, for them to be aware of ...

R:	Education.

I:	... about …

R:	Yeah.

I: 	Drones? Yeah.

R:	Yeah.

I:	Yeah. Okay.

R:	But as I say, you turn on the news, and horribly, we see every single war that's going on, or conflict that's going on, we see them used for bombing lives out of people. Surely, I’m pretty sure the general public anywhere in the world will accept the fact that it's being used to save lives.

00:57:48

I:	Yes. Yeah.

R:	And the technology is there, and it's been there a while.

I:	And what would you want to know from the call handler, specifically about drone delivered defibrillation?

R:	That it's on its way. That it's safe. That it will tell me what to do. That it's being backed up with professionals. And then, there's, "Y- you can't go wrong with using the defib, and please don't run off up the shops if you know one's there.” That it's coming to you.

I:	Yes. Yeah. And it's interesting that you say that there might be some fear around the technology initially, but actually, if people can see drones doing ...

R:	I, I think that would be …

I: 	Harm …

R: 	Age related, and I may be ...

I:	Yeah.

R:	... Am I remiss in saying that, ethically? Am I being judgy? I, I think the younger generation, there may not be.

I:	Yeah. So, over time, yeah, it could be ...

R:	Yeah. Over time, it will ...

I:	(inaudible)

R:	... and as I say, anybody that watches the news sees drones in use. But ...

I:	Yeah.

R:	... for me, for the wrong ...

I:	Yes.

R:	For, for military, let's just say ...

I:	Yeah.

R:	... for military reasons ...

I:	Yeah.

R:	... they're not, you, you know, they'll see them hovering above rugby games, and everything. They'll see people using them at pop concerts, and whatever, so ...

I:	Yes. Yeah. Why not use them for this, yeah.

R:	I don't think they'll be that surprised *that* they're delivering life saving equipment, do you see what I mean.

I:	Yeah. Yeah, I do. Okay. And any problems, or concerns, or issues that you foresee with this?

R:	No.

I:	Nothing.

R:	Only, no.

00:59:12

I:	No. You think it's a good, good thing.

R:	Only like you say, I think with the landing of it, and the detaching of it. And then perhaps if it doesn't detach, you know, because obviously for military technology, that, it's not spy in the sky and then dropping something is it, it’s, it’s gotta come down, and it's gotta do a job on the floor. So, that would be my, you know, that's gotta be robust technology, that it will release. Whether it's from a cable or not, and it, it's gotta be safe, as you say, with overhead cables, and the blades are not gonna hit someone, or whatever. But apart from that, no.

I:	Okay.

R:	No fear at all.

I:	Excellent. And I know I’ve taken up far too much of your time, so I, I just have ...

R:	No, it’s okay.

I:	... final brief questions that may be useful for our research. So, what is your age, so if I may ask, what is your age and gender?

R:	What is my age? I’m 52.

I:	And gender?

R:	Male.

I:	Okay. And have you had training in CPR and defibrillator use?

R:	Just a bit.

I:	Okay. And so, when was this?

R:	Continuous.

I:	Yeah. You're still having it, and ...

R:	Yeah. Well, I’m an instructor, so ...

I:	Yeah.

R:	... and I, and that's one of the reasons I make sure I respond with [Name of ambulance service] to keep my clinical skills up.

I:	Yes. Yes. How long have you ...

R:	'Cause, fortunately in my role, we don't see them very often. So, I keep my clinical skills up, and that provides, put my money where my mouth is, and try and do, give something back to my community when I can.

I:	And how many years have you had this training? Has it been ...

R:	34.

I:	Yeah.

R:	Oh, 36.

I:	So, yeah, many, many years. And it's been both CPR and defibrillation?

R:	Yeah.

01:00:53

I:	Yeah. Okay. And so, the training has occurred before, and also after the incident we discussed.

R:	Yeah. Yeah.

I:	Yeah. Yeah. Okay, great. Thank you. So, is there something else you would like to add to your answers today?

R:	No. Just I really look forward to reading it, it’s fascinating.

I:	Okay, excellent. So, I’m just going to turn the audio recorders off, and just briefly go through what will happen next, if that's okay.

R:	Okay. Yeah, no worries.

**END OF INTERVIEW**

Participant 14

I: 	Okay, excellent. Thank you. So, the main interview audio recording has begun, and it will continue until the interview questions end. Okay. So, can you tell me as much as you can about when you provided assistance to the person who had a cardiac arrest, please?

R:	Yeah. It, I was asleep, and I was woken up by my husband making noises. I thought he was snoring. So, I pushed him to wake him up, to, to turn over. But when I pushed him, his skin didn't react in the way I expected it to. And when I looked at him, he was actually lying prone on his stomach, and when I put my hand on his neck, he was soaking wet. So, I checked his pulse in his carotid and couldn't find a pulse. Checked his pulse in his wrist, and couldn't find his pulse. Thought, “I can't believe this is happening.” So, I ran out of bed, turned the light on, and saw he was prone with his face flat in the pillow. I dialled 999, and I turned him onto his back on the bed, and I started CPR. (Pause) want to carry on with the whole thing, or just stop there?

I:	Yeah, if you could go through what happened. So, when you first noticed that he was not right, like you say, and you phoned the ambulance, and you performed CPR. And how did you realise what was happening? If you could just talk me through ...

00:01:36

R:	Okay. I should tell you that I’m a trained nurse and midwife, so I’ve had CPR training for 40 years, every year. And I have undertaken CPR in hospital settings. And I am a first aid at work trainer, so I train all the CPR and defibs, just for context as well. So, I knew straight away that he had had a cardiac arrest, because he wasn't grey, or, no blueness or anything, he was warm to touch. So, I knew he was very much alive, but he was devoid of a pulse. So, I phoned 999, put the phone on on loudspeaker. Turned him on his back. I started CPR. I had, I completely forgot about the breathe, the breathing. I literally did compressions. I was shouting at him whilst I was doing it. “Don't, you can't leave me, you can't leave me.” And I was shouting at the person, the call handler, the ambulance service, saying, "Where's the ambulance? Where's the ambulance? It’s been ages.” And he said, "It's been two seconds,” or something like that. He didn't talk me through *anything* at all with CPR. *Nothing*. So, when I made the call initially, I, I just, kind of, robotically said what I was trained to say, which is, "Cardiac arrest. Male, [age]. I’m on my own.” And I gave the address. So, it, kind of, you know, it's been imprinted in my head 40 years, what to say in, in a cardiac arrest. And I just carried on shouting at my husband that he's not gonna die on me, and that, you know, I was just carrying out (animated tone) *CPR* with just the compressions, no breaths. And kept saying to the ambulance, "Where is he, where are they, where are they?” And he, they were saying he, "They're on their way. Go down and open the front door.” And I said, "*No*, I’m not opening the front door, because I don't want to stop the compressions.” Also, it was [very early] in the morning, I’m in my nightdress, don't live in a great area. I did not want to leave the front door open. So there's only me in the house and my husband. Didn't want to leave the front door open. And so, I said, "I’m not gonna open the front door until they're at the front door, and then I'll come down.” 'Cause I just thought, "I don't want someone coming in and robbing me, or coming ...” I just, kind of, I just wanted to carry on with my husband. But then, at some point, they said to me, "Go down and open the door, the paramedics are here.” So, I went down, opened the front door. I can't remember who came first, whether it was a, I think it was an ambulance with two or three paramedics in there. And they ran upstairs, and they put him on the floor. And they got out the defibrillator and the adrenaline and everything. Put the machine on him to look for a heart rate. I asked what I could do, and they, they asked, they asked me to open packets for them. I told them I was a nurse and a midwife, they said I could open packets for them. So, they were doing the resus, they were carrying on with their CPR, and looking at the monitor and shocking him, giving adrenaline. And then a paramedic arrived on a motorbike, and he, he came ... Apparently CPR was his speciality, so he carried on as well. And it was, kind of, like I was having an out of body experience. It was as if I was watching my husband on the floor, watching me on the side of the bed helping the paramedics, and it was like it wasn't real. For me, it was *completely* out of body experience. Yet this was my bedroom, and I can sort of all, kind of, crazy. And I thought I had this golden thread to my husband, and I was saying to him, "Come on, come on.” Every time they shocked him, "Come on, you can do it. You can do it. You're coming back to me. Come back to me.” And eventually, I don't know after how many shocks, or after how long, I’ve no idea. But they found rhythm on the oscilloscope, and they said, the lead paramedic said to me, "Okay, we’ve got him back. We’re gonna take him to the ambulance.” And yeah, so they carried him down the stairs, put him in the ambulance. I went with them, because I wasn't (chuckles slightly) leaving him alone with anyone. I had to be there. I felt like, yeah, I couldn't let go of this golden thread. And we went to the A&E. At the A&E, I asked the registrar whether they thought he could have had a stroke, and would they do a CT scan, or an MRI, because I wondered if he'd had a stroke. And they said, "No, he's not had a stroke. He's stable now, we're gonna take him to intensive care and cool him down.” So, they did that. They took him to intensive care, they cooled him down. They let me in there, and I helped settle him down, and get the TED stockings on him, and talked to

00:06:27

him, and all the rest of it. Yeah. And then, so that was, kind of, the end of that. And he was in intensive care in a coma for a week. They tried to bring him round, but they couldn't. He kept, well, they just couldn't bring him back. And eventually, after a week, they did bring him back. He was, he had quite bad side effects. He had myoclonus, so jerking of his muscles. He couldn't hold a spoon. He couldn't eat, couldn't talk properly. He couldn't walk. Nothing. So, no one really knew what the future was going to be. But between us, I spoke to the ward sister and I was allowed to, I spent ten hours a day in there basically supporting his recovery, bringing him food, nurse, helping to nurse him. He's a very strong willed person, and he was determined that he wasn't gonna end his life in that hospital bed. He was gonna get better and, you know, walk outside. And, yeah (?), he went from a, he had an internal defibrillator fitted two weeks after the cardiac arrest. He had an MRI of his heart, he had an angiogram. They could find *absolutely* *no* reason whatsoever at all why he'd had the cardiac arrest. His heart was absolutely normal, his arteries were completely normal. They had no idea why he'd had it. It just, kind of, happened in his sleep. And I think if I hadn't been there with him, and he was somewhere else, he would have been one of those adults that had an early death at 57. But luckily, fortunately, he wasn't. Yeah. And then started to really ... Oh, he went to a, a rehab ward for people that had had strokes, which didn't really do him any good. But he was meant to get physio in there, and occupational therapy, and psychological support. And he was able to go to that ward, because a very good friend of ours was the head of medical HR, knew all the consultants, and managed to get a bed, the next free bed for him. So, it wasn't that he was on a pathway to go there, it was actually because of who we knew that we managed to get the care for him that we felt he needed. And I think he was discharged after six weeks. And then, really, he, I had three months off work, and then he started building his strength, and learning to walk, and, you know, get psychologically recovering. So, now we're 11 years later. Well, before 11 years ... When he was being discharged from the hospital, the consultant said to him, "Just go and live your life.” So we did that. He wanted to go, he's always been a [describes very specific hobby, equipment and how this is a large part of their lives now]. So, he's made an absolutely incredible recovery. And yeah. That’s, that’s in a nutshell (chuckles slightly). That's the story.

I:	Well, I’m just ... Sorry, I’m just going to turn my speakers up, 'cause I’ve realised they’re actually at 40. So, hopefully that, that will, I can hear you, but ...

R:	Okay.

I:	Yeah. But so, firstly, what, you, you know, you've described is very upsetting. So, are you okay to continue, first of all?

R:	I am okay …

I: 	… I want to make sure ...

R: 	… To continue, yeah. I must, you know, I do, it's hard to talk about it, even though it's 11 years ago. I’m still feeling, I still find it difficult to talk about, but not as difficult as I found it all those, you know, years prior to ... That's my husband just walking by me. That, I don't find it as difficult to talk about now, obviously, as I did back then. But yeah, it's quite hard to talk it through, and to relive it. But *nowhere* near what it was. And actually, I really believe in contributing to research, so I’m very happy to, to talk it through.

I:	Okay.

R:	And he's very, very supportive anyway, so yeah.

I:	Yeah.

00:10:16

R:	Thank you for asking, anyway.

I:	No, that's fine. My priority is first and foremost your, your wellbeing, above getting research ...

R:	Yeah.

I:	... data.

R:	Yeah. Yeah.

I:	So, I wanted to check that, that you were okay to continue. And like I say, if you ...

R:	Yeah.

I:	... want to stop, you, you can (chuckles slightly). Just to remind you of that. So ...

R:	Yeah.

I:	... if I may just, kind of, go back to the beginning, when you saw him lying prone on the bed, and you realised something wasn't right. You said that your medical knowledge, coupled with what you could see, meant that you realised something wasn't right. But did you know it actually was a cardiac arrest, or did you think it could be a stroke, or a heart attack, or something else?

R:	I, I’m not, I’m not allowed to diagnose. What I knew was he didn't have a pulse, and if someone doesn't have a pulse, you start cardiac arrest. So, no breathing, no pulse, start CPR. So, I didn't know what he'd had, but I just knew what I had to do.

I:	Yes. That makes sense. And was that immediately after ...

R:	Immediately. Immediately. Immediately. I’ve seen, you know, 40 years nursing and midwifery, I’ve been at I can't tell you how many cardiac arrests, how many dead people I’ve seen. I *knew*. I just *knew*. Immediately there was no pulse, I knew that it wasn't right, and I had to do CPR.

I:	Okay.

R:	Yeah.

I:	Okay. And did you have immediate access to a defibrillator?

R:	No, it only came when the paramedics came. I wasn't told by the call handler that there was one in the vicinity that they could have brought to me, nothing. I wasn't told anything. I was just told to wait for the paramedics to arrive.

I:	Okay.

R:	Well, no, I was told they were, they were on their way. That was all I was told.

I:	Okay. And I'll ask you a little bit more about the call handler’s input, or lack of input in helping you through that ...

R:	Yeah.

I:	... cardiac arrest process in a moment. But just for the time being, you said that you were on your own with your husband. So, how did, how did that make you feel? And I, I think you've touched upon this already, but ...

00:12:30

R:	I, well, do you know, my first reaction is, "I can't believe this is happening.” That's, I still remember turning the light on, looking at him going, “I *can't* believe this is actually happening.” I, I was so shocked, because your bedroom and your home is a place of calm and safety. And here he was with no pulse. We went to bed, not, not an issue. In the morning, we were getting up to fly away to go for a week's holiday playing tennis. So, he was very healthy, didn't smoke, didn't drink. Not overweight (chuckles slightly). Nothing wrong with him! So, to wake up and find him in that state, I was in utter disbelief. And then, I was really angry with him for dying on me, basically. I was like, "You can't ...” I was really, I mean, I was on the call, apparently I was swearing my head off at him, that he can't leave me, and, "We're meant to be going on holiday, and you not gonna leave me! Rah! Rah! Rah!” So, (chuckles) I didn't really, I felt, when I look back on it, first thing I felt was disbelief. The second thing was absolute anger. How *dare* he do this? Which is, sounds absolutely absurd. But I was like, "You can't do this! You can't leave me! You can't die!” So, then I just felt I had to do *everything* in my *power* to bring him back. And in fact, we met up with the paramedics sometime later to thank them, and the lead paramedic, he told us that he was thinking in his head how to tell me that he won't be able to bring back my husband. And he didn't know how to tell me, because he knew from my, the way I was, kind of, he said, "I’ve never had a ch, cheerleader at a resuscitation.” I was *literally* *urging* my husband, you, you know, "You are ...!” Every time, "Come back, come back! Come on, come on, come on!” I’m surprised he came back, the way I was swearing at him, but anyway. I was really urging him to come back, and the paramedic just said, "I didn't know how to tell you, because I know you would *not* let me stop.” He said that, "Out the corner of my eye, I just saw something on the screen, and I thought I'll give it *one* last go.” And on that *one* last go, the last shock, they got his rhythm back. So, it was *so* close. If he hadn't have seen that, if he hadn't have done that last shock, I wouldn't be here now, my husband wouldn't be here now. We wouldn't have had the last 11 years. I don't know if all the ducks just lined up that day, if it was how I was behaving, you know, made the paramedic give it one last go. They don't, they didn't have a protocol, and if they did he didn't stick to it. So, how did I, I feel? Disbelief and anger. That sound weird? That's how I felt.

I:	No, no, that's valid. You felt like that, those feelings existed.

R:	Yeah.

I:	So, they're, they're perfectly ...

R:	Yeah.

I:	... valid. Yeah.

R:	Yeah.

I:	And I also wanted to ask you about how you felt about leaving your husband to let the ambulance crew in. You said you had to go downstairs ...

R:	Yeah.

I:	... to open the door ...

R:	Downstairs. I did not want to leave him at all. I felt guilty for leaving him. I didn't want him to die when I left him, you know. Plus, I was in a skimpy little nightdress, so I didn't really wanna, with nothing underneath it, so I didn't want, wanna go down, I didn't know *who* I was opening the door to. So, I had that to consider. Yeah. I did not want to go down and open the door. I really did not want to leave him.

I:	But, but, but you did, you, you ...

R:	Well, I had no, he, the, I had to open the door to let the paramedics in, 'cause I know that they, they could save his life. So, you know, they would have had the adrenaline, and the defib. So, you know, I would’ve, if I hadn't’ve done that, obviously he would have died.

00:16:33

I:	Were you instructed to leave him by the, the call handler, then? I, I think you mentioned.

R:	I can't remember, oh (?) I just was, remember being told by the call handler that, "The paramedics are outside, can you go and let, let them in.”

I:	Yeah. Yes. And that's when you went downstairs.

R:	That's when I went down.

I:	Yeah.

R:	I would be, I wouldn't go down, and I ran down. I literally ran down the stairs, opened the door, *ran* back up the stairs, and carried on with the compressions. I did not want to leave him without the compressions.

I:	Yes. Yes. So, it was difficult ...

R:	That was, it was tough.

I:	Yes, because you were having to balance the, giving the chest compressions to your husband, but then leaving him temporarily to open ...

R:	Yeah.

I:	... the door to let the people, the paramedics in ...

R:	Yes.

I:	... so they could shock him, and ... So, it was another thing you had to weigh up in your mind, presumably.

R:	Yeah.

I:	It added to the stress of the ...

R:	I, I don't think it, it, I don't, I think I was, kind of, in robotic mood at the time. I didn't, conscious, I don't recall thinking things through. I was, I just, just remember doing everything that I’ve always been trained to do, you know. I, there was no thought in it, it was just natural. I’m so well trained in it. The thing that threw me was leaving him to open the door. But of course, if you, you don't open the door, then the help doesn't come to you. So, I didn't feel comfortable with it. I didn't want to leave him. But I knew I had to do it.

I:	Yes. Yes. That makes sense. So, can you go through a little bit more about the 999 call, which I know you've mentioned already.

R:	Yeah. Yeah.

I:	So, you used a mobile, or a landline?

R:	I used a landline. I put it on loudspeaker, I put it on the bedside table. I did 999. I said, "Cardiac arrest, male 57, I’m on my own.” And then he said, "Well, what, what, what's the ...” You know, he started asking me all these questions. And I’m just like, "Never mind the name, (animated tone) just send the bloody paramedic. This is my address.” But obviously the paramedic, the call handler had a script that they had to go through, but I didn't care about the script. I just wanted to give the address, and get them here. But I had to go through the script. And obviously, went through the script, and yeah. He didn't tell me, he didn't ask me to get him on the floor, so he was on the bed all the time until the paramedics arrived to put him on the floor. Because I was on my own, it didn't occur to me to put him on the floor, because I just wanted to give him compressions. I don't think I could have got him on the floor without, on my own, without doing any damage to him. If the call handler had said to me, "You need to put him on the floor,” I might have tried. But I probably wouldn't have tried, because I would have felt it more important to do the compressions than it was to get him on the floor and potentially cause more damage. So, no, there was no anything from the call handler. And in fact, when we met the paramedics, the call handler was no longer working for the ambulance service. So, they'd obviously listened to the call when we said we wanted to meet up with them. Whether they thought we had a complaint or not, I don't know. They, the paramedics had never met anyone from a CPR afterwards. They were really nervous to meet us, 'cause they thought we had a complaint. We just wanted to say thank you. But the call handler was not working for the organisation anymore. Didn't get any other information than that.

00:20:13

I:	Right. So, I’m getting a sense that you weren't satisfied with the interaction between you and the, the call handler. Or, what are your reflections on it?

R:	Well, my, yeah, my reflections are they're supposed ... (sighs) No, I was completely dissatisfied. Less so at the time, because I had my mind on other things like keeping my husband alive. However, on reflection, I thought, "Hang on. He didn't ...” They're meant to talk you through CPR. They're meant to count for you. That's the algorithm is, with, you know, "Have they got, have they got a pulse? Are they breathing? No, no. Okay.” And then it, they're meant to talk you through everything. Didn't talk me through anything. Though on reflection, I didn't think this at the time, but on reflection, I realised that they should have talked me through it all.

I:	Yes. Yeah. Would you say anything went well, or would you say it ...?

R:	With, with them?

I:	Yeah, with the call handler.

R:	With the call handler. Well, the only thing that went well was the ambulance with paramedics arrived, with, well, apparently within eight minutes. So, that went well. But my interaction with the call handler, if I was, if I didn't have the knowledge and the training that I'd had, I would be standing there, just look at him die.

I:	Right. Right. So ...

R:	That's how I feel.

I:	Can you remember what he said, the call handler? Or any help that he gave you?

R:	No help.

I:	Nothing.

R:	Nothing. Nothing. Nothing. No. Was quiet. Asked me the questions, was quiet. Apart from when I said, "Where are they? Where are they? It's been ages.” And then he said, "It's only been two seconds,” or two minutes, I can't remember what he said. “Keep doing the ...” ‘Cause (?) I don't even know if he said keep doing the CPR. I, I just can't remember. I, I don't remember.

I:	No, that’s ...

R:	But I, I do remember not being given any instructions, no, "Put him on the floor.” No, "Let me count you through it. Now do this, now do ...” Nothing. Nothing, nothing, nothing.

00:22:24

I:	Okay. And ...

R:	But there was a lot of me effing and blinding on the call, 'cause the paramedics were laughing that they'd listened to the call, and I was swearing at my husband a *lot*. And so, maybe the call handler felt a bit worried, or scare .... I don't know. But no nothing (chuckles) at all. Nothing.

I:	Okay. And you said that the call handler asked you lots of questions. So, can you remember what any of these were?

R:	I mean, it was 11 years ago, so I don't remember. Other than I think they asked me his name, age, and the address, and what had happened. Just the basic information, in order to assess the prioritisation, I guess, of the paramedics to come to the house. But, I mean, obviously, I knew there was no pulse. I said, "No, pulse, and he's not breathing.” So, yeah, but nothing else.

I:	And did you ask the call handler any questions?

R:	Only, "Where's the, where’s the ambulance?”

I:	Yeah.

R:	“Where are they? Where are they?”

I:	Were there any technical issues on the phone when you were talking to the call handler?

R:	No. No.

I:	No. Okay. And so, you said that you performed CPR, and you did the chest compressions only, or did you end up doing them with rescue breaths?

R:	No, only chest compressions. *Completely* forgot about rescue breaths.

I:	And ...

R:	Didn't even occur to me.

I:	Yes. Well, that's understandable given how stressful and chaotic the situation was. And what are your reflections on, on how, on how that went, the CPR?

R:	Okay. Obviously, it went well, because he's alive and living his, well, his best life for the last 11 years. So, clearly, either that went well, or CPR doesn't matter, and something else was at play. I mean, who knows. So, there's that. However, from a technical perspective, when I, when (chuckles slightly) I'll tell you, when my husband came round from everything, and we were talking about what had happened ... He was still in hospital, and I told him, and he said, "Did you put me on the floor?” And I said, "No.” He said, "What, you did CPR on the bed?” And I said, "Yeah.” And he went, "Well, you didn't do it properly.” So I said, "Well, by the fact that you are having this argument with me now, I must have done it properly, 'cause you're here and talking.” So, but he, at the time, he had all this myoclonus, and, you know, he clearly was in a really poor state and no one knew what the long term outcome would be. So, there was that. On reflection, I feel that I should have put him on a hard surface, obviously. Because from my nurse training when I’m 18 years old, you take the mattress off the bed, and you have someone on a hard surf ... So, it's all stuff I should have known, but just didn't think of. So, I don't think it went well, because ... Well, okay, it did go well, because he's alive. But however, I could have possibly done it better by taking the mattress off, which is hard on your own to get a king size mattress off a bed. I couldn't have put him on the floor on my own, without, I think, doing him some grave injury. I did my compressions too quickly. I didn't break any ribs, so I don't know if I did them properly. And I didn't do rescue breaths. So, who knows! Do you know what I mean, I don't know. Did I, did I do it well, or didn't I? I don't know. I didn't do it as I should have done it, but he survived and he's fine.

00:26:11

I:	Yes. Yeah. And you were doing all of this without any instructions from the call handler.

R:	No instructions, no.

I:	So, you were having to remember from your work training ...

R:	Well, staying alive, staying alive, staying alive. Yeah. But I didn't remember, did I, ‘cause I didn't do the rescue breaths. But he was perfused! Do you know what I mean? He was perfused. You are medical, you a paramedic, or, I don't know ...

I:	No, and I’m not allowed to give ...

R:	Oh, okay.

I:	... any, even if I was, which I’m not, I’m ...

R:	Okay. Can’t say.

I:	... I’m a health scientist, I’m a researcher at Warwick.

R:	Right. Fine. Okay. Alright. So, he was, he was still perfused. He wasn't, he didn't have pallor, he wasn't blue around his lips. There was nothing about him that made me think he's not going to survive. I knew he wasn't, although he, you know, he's dead ‘cause his absence of pulse, but he wasn't dead, because he was clearly still, his soul was still alive, his soul was still within him. He hadn't left, and he, he wasn't the bag of bones on the bed when somebody's dead. So, I didn't, I don't know. I, what was the question? I’ve forgotten the question.

I:	No, well, no, it's fine. I was, you know, I’m, I’m, I’m just wondering that you're having to, to manage this stressful situation with little to no help from the call handler, and I’m just ...

R:	Yeah.

I:	... trying to get a sense of how that made you feel, and how you worked through that without your actions being facilitated by the, the call handler. I mean, what, what ... Sorry, there's a lot of questions in here. But what was the call handler doing while you were, were doing that?

R:	Nothing.

I:	Were you interacting, were you, were you telling them ...

R:	No, silence. Radio silence. Radio silence. I, I haven't had a copy of the call. I could have asked. We could have asked for it, but I didn't want to hear it, 'cause I knew that I was, like really, really swearing a lot at my husband (animated tone and chuckles), and telling him he's gotta come back in very threatening behaviour for me. But I, I was having this out of body experience. It wasn't me doing the CPR, I was watching someone that looked like me do the CPR. So, I think I was just in automaton mode. I have, every year, since the age of 18, and I was 50 years old when this happened. So, for all those years, every year I’ve been to CPR training. And I have attended during my nurse training so many CPRs, that it's embed, I’ve never seen anyone faint, but I’ve seen a lot of cardiac arrests. So, I don't think I even thought, I just *did*. I didn't think. There was no thinking. If I'd have thought, I'd have put him on the floor (animated tone), and I'd have done rescue breaths. I think I was just on completely automatic mode. “I’m gonna press your bloody chest until your heart starts beating, and you're coming back. And you're not going anywhere.”

I:	Yes.

00:29:22

R:	There was no thought.

I:	No.

R:	Thoughts left my head. That's the thing. I think, in hindsight, and on reflection, if the call handler had said to me, "Okay, now you need to ...” Then I would have done. Would it have made any difference? I don't know, because the outcome's been good. Is the outcome good because of defibrillation within, what, 20 minutes, and adrenaline, and all the rest of it? I, I don't know. I‘ve no idea.

I:	Yes. Yeah.

R:	No one. I think no one will ever know the answers.

I:	No, no …

R:	But I think it would have been nice had the call handler talked me through what to do.

I:	Yes, yes, rather than you having to get on with it yourself effectively.

R:	Yeah. Which is what I did.

I:	Yeah. Okay. So ...

R:	(coughs) Sorry.

I:	That's okay. Talking of defibrillators, what are your thoughts on why one was not used until the paramedics arrived?

R:	Well, a) I didn't have one in the house, I wouldn't. B) obviously the call handler didn't say there are ... I mean, I don't know at that time in [year] if there were community defibrillators. There are many more now. I have no idea. Well, I didn't have access to one, so I couldn't use one.

I:	And ...

R:	But also, what else plays into it, ‘cause I’ve been thinking about ... Obviously, knowing I was coming to do this research with you, would I have opened the door for a drone, a defibrillator on a drone? And this is probably one of your future questions. But I don't think I would have done? On my, if I was on my own. I, I would have not opened the door to a drone. I wanted people there who knew what they were doing to bring him back. ‘Cause I thought I knew what I was doing, but I didn't have adrenaline, I didn't have the defibrillator, I didn't have an IV. I didn't have anything. But they do. So, although defibrillators are the first line to bring someone back, in terms rhythm. Yeah, I’m used to hospitals where everyone comes with a cart, and, you know, all the bits on the cart. So, would I open the door to a drone defib? I don't think I would have done, no. Because it's extra stress. A) you've gotta open the door. I’m not, was I trained in following a defibrillator? Not at that stage. Would I have read the instructions properly? I don't know. They’re, now that I train others to use defibrillators, I think if there's a crowd of people in an open public space and there's help and support, yes. If I hadn't of been on my own, yes. On my own, I don't think, no, I would have wanted the paramedics there, with all their kit.

I:	Oh, that's really interesting. And yes, you're right, I was going to ask you about that in a few moments. So, perhaps we can explore that again in a moment.

R:	Sure.

00:32:16

I:	But just to bring you back to the incident, if that's okay, and the, you know, the fact that you didn't have a defibrillator available at the time. Were you aware of a defibrillator at the time that you didn't go and get, or, or you, you weren't aware or?

R:	Well, I mean I knew about defibrillators, 'cause we obviously had them in hospitals. So, I knew about defibrillators. I didn't know about, never crossed my mind that there could be a community one that someone could deliver. I wouldn't have gone and got one, 'cause I wouldn't have left him.

I:	Yes.

R:	But yes I knew about defibrillators.

I:	Yes. But what about nearby though, in your vicinity where you lived?

R:	No idea.

I:	You didn't know, no?

R:	No idea.

I:	No. Did you subsequently realise that there was a nearby defibrillator? I’m not saying that there is, but …

R:	I don't know that there is.

I:	You didn’t know.

R:	Even to this day, I’ve no idea.

I:	No. And like you say, the call handler …

R:	No, it didn’t ...

I:	Didn't ...

R:	... didn’t cross my mind, 'cause the paramedics were coming with all of that.

I:	Yes, yes. And like you say, the call handler didn't advise you of one.

R:	No.

I:	No. Okay, that's, that's great. Thank you for confirming that. So, what are your thoughts about using a defibrillator in the future?

R:	I think if it, it would depend on the circumstances. Obviously, you need to use a defibrillator to get someone back into sinus rhythm. Doesn't mean everyone’s gonna get back into rhythm, but you stand a better chance of someone surviving with a defibrillator. I am now, I’ve trained people now to use defibrillators, and I, everywhere I go, I see defibrillators in boxes on walls. So, they are very, very, very commonplace. But also, do I notice them because I’ve been through what I’ve been through, and I’m part of a group where people are, you know, doing charity stuff to raise money for community defibrillators? It's like when you're pregnant, you only see pregnant women. Do I only know about defibrillators and see them now everywhere, because of what we've been through? I don't know. Back in [year], it didn't even *cross* my horizon at all, 'cause I didn't expect to have to use one in my home [very early] in the morning. However, if I was at an RTA, or someone collapsed on the street, or where I work, where I’m now, yes, I would use a defibrillator. I would send someone to get it. I would know how to use it. I'd follow the instructions. But do you know what the difference is? This was my husband. If it's somebody that

00:34:59

I am not emotionally attached to, it's a whole different ball game. So, in the hospital scenario, or if someone has an accident, and I, I’m not emotionally attached to them, I’m a very different practitioner. With my husband, yeah, of course your emotions take over, and you behave very differently. So, I wouldn't need the defibrillator now, because he's got one implanted in his chest. The interesting conversation we have is, should we have one in case something happens to *me*?  So ... I will never be in that situation again with my husband, 'cause he's got his own. If it was someone else, yes I, very, the first thing I would do in that same scenario, I would ask someone for a defibrillator. If I was on my own, and it wasn't someone I was emotionally attached to, would I leave them to get a defibrillator? I don't think I would. Because, complete, I dunno, it's hard to know. If you're on your own, it's just such a completely different scenario. I would, I think I would because I, if I weren't emotionally attached to that person, I would ask the call handler, "Can you get me a defibrillator?” And if I was with other people, I would say to someone, "Get defib, go and find ...” Or, “Go and get a defibrillator.”

I:	Would that make it easier for you to use a defibrillator in the future, or ...?

R:	Would what? If it wasn't someone I knew?

I:	Yes, and asking the call handler to get someone to get it for you. Would that, would those things make it easier, or would there be something else ...

R:	Not ...

I:	... that would make it easier?

R:	No, not being, not being on your own would make everything easier. 'Cause when you're on your own, and you feel responsible to not let that person die, then you don't want to leave that person. But I think even, no matter who it was, I think if I was on my own again, I would use my mobile phone to call someone, and get something. Although if you're on the phone to 999, you can't then use your phone to phone someone else. I would like to think that the call handler's algorithm talks you through that, and they said, "There is a defibrillator in the area. We'll get it sent to you.” But again, you know, if I was on my own, would I want to leave the person and open the door? If it was someone I was emotionally attached to, probably not. If it was someone that I wasn't, probably yes. I, it's hard to know until you're in that scenario.

I:	Yes. Yes. So, leaving the person, if it's somebody that you know and emotionally attached to, could possibly prevent you from using a defibrillator, because of concerns that you wouldn't want to leave them, in case they passed away while you were getting it. Is that correct ...?

R:	Yes. Yeah. Yeah, I think that’s, I think that's how I feel, yeah.

I:	Okay. Lovely. Thank you. Is it okay if we move on to the remaining part the interview, which is asking you about ...

R:	Of course, yeah.

I:	... drone delivery of defibrillators. So, firstly ...

R:	Yeah.

I:	... is this something that you've heard of?

R:	I had not heard of it until I read about the research.

I:	Yes. Okay. Well, it is new technology, drone delivered defibrillation, so that's, that's, that makes, that’s understandable. Have you heard of drones?

R:	Oh, yes, yes.

00:38:27

I:	Yeah.

R:	We own a drone.

I:	You own a drone, did you say?

R:	Yeah. Yeah.

I:	Oh, okay. So, you've used it yourself, presumably?

R:	Yeah. Yeah.

I:	And may I ask in what situation?

R:	Just film, filming stuff.

I:	Yes. Can you imagine how drone delivered defibrillation works?

R:	Well, I do have a wild imagination. So, in my imagination, it's got some kind of bracket underneath it that the defibrillator is attached to the top of the drone, someone's sending it, (makes a buzzing sound) and it lands on your front doorstep. I have no idea if that's how it works or not, but that's what I imagine.

I:	Yes, yes. So, the defibrillator would be in a box, and that would attached to the drone. And the drone would land and detach the defibrillator to wherever the bystander was with the patient. Or the drone can ...

R:	Yes.

I:	... hover above the ground, and winch the defibrillator down to the ground, via, like ...

R:	Oh.

I:	... a rope, or winch like device.

R:	Oh, wow.

I:	So, obviously, none of that is in operation at the moment. But notionally, that’s, that’s how it, it ...

R:	Yeah. Yeah.

I:	... could work, if it was to be rolled out nationwide.

R:	Yeah.

I:	So, can you, could you imagine interacting with, with a, a defibrillator in the ways that I’ve just described?

R:	I could, yeah. Of course, now I’m gonna just say, this of course depends on the scenario based on the previous question. If I was in a public place, someone had collapsed, needed CPR. Yes, I could absolutely imagine, I think it would be *incredible*, and yes. However, if it's the middle of the night, and you're in your own home, and you have to leave someone ... I think no, because I would want the guarantee of the paramedics with all their kit coming, and that would make me feel that my person was going to survive. And it's just based on the emotional attachment to someone you love and spend your life with, to strangers you don't know. All lives are of course equal, however when you're emotionally attached, it is, emotionally unattached, it is different. So, if there was more than, if I, if I was in a public place and on my own, yes I would use it, I think it would be brilliant. If I was in a public place and with other people, I would use it. If I was in my own home, or the home of a person I’m emotionally attached to, and it's during the day, yes, I probably would use it. If they were downstairs, and if there were people with me in the home I would use it, 'cause then they could open the front door.

00:41:15

I:	Okay. And I just wanted to ask you around safety, that the, you know, I just gave those two different ways that the AED could be delivered to the bystander via the drone.

R:	Yeah.

I:	Do you think either way is more or less safe? Or they're both safe, or you have concerns about safety with both of the methods I’ve just described?

R:	Well, I think, a) if a drone's approaching, you wanna get everyone out, if people around you wouldn’t want to get anyone out the way, so no one gets clobbered by a bloody drone delivered defibrillator. I’m presuming as well, whoever's operating the drone can see where they're landing it. I’m also making the assumption that there'll be protocols in place. So, whoever you're talking to says to you, "Can you get everyone out the way? I’m gonna land the drone on wherever.” So, I’m making a lot of assumptions. I, I think if it was a drone on a winch, 'cause you could actually, and you're upstairs in a room, you could maybe bring it to a window, and then you could reach out to the window, and get a drone on a winch. Whereas possibly you couldn't do that with a drone that's just being delivered to the ground. So, it might have different applications. Or if it's in the top of a building, or something, I dunno. Different applications. As long as somebody is guiding me on where it's gonna land, get everyone out the way, and this is how you detach it, I wouldn't matter (?). And I can't imagine it's gonna be on a crane like hook that might swing and kill someone, (chuckles slightly) but I don't know.

I:	Yes. Yeah. Okay.

R:	But it'd be nice, actually, to have a visual of what you're talking about, so you would know how to answer. But I can't see what the design is, so I, I just don't know.

I:	Yes. Yeah. We are currently testing the feasibility of it at the moment, that's the other, the other work package associated with this project.

R:	Okay.

I:	To see what it could look like if it was rolled out. But as I say, in the UK ...

R:	Yeah.

I:	... there's nothing like this at the moment. That's why we're asking bystanders what they think, whether it would be worth trialling it in, you know, additional research studies, or whether bystanders think it's a, you know, no go area, no point. Or whether they think it's got some promise and potential that we could take forward. So, that's why we’re asking ...

R:	I think it's a really, really ... I think it's a really exciting idea. And given health services as they currently stand, and waiting for the paramedics ...

I:	Yeah.

R:	... if you could get a defib delivered to you by drone in super quick time, of course. I mean, it would be an *amazing* thing. The only thing I think personally, and I don’t need to say it again is whether you're on your own, or not on your own, and what time of day it was.

I:	Yes. Yes. So, I’m getting a sense that you've, you would feel, I don't, you would see benefit in having a drone deliver a defibrillator to you whilst helping someone have a cardiac arrest, but you would have concerns ...

00:44:18

R:	Yes.

I:	... about leaving them, particularly if it's somebody who you're emotionally attached to.

R:	I, yeah, I think that, that's fair. I, I mean I would have concern ... If I were on my own, it's a different scenario to if it's in, you're in a home with other people or in a public place with other people. When you're on your own, you don't want to leave that person, that, though your duty is to help them survive. If you're running downstairs, or running to open the door to get a drone, it's gonna take two seconds, and it's not complicated, and someone's talking through, "Okay, you've got the drone. Now carry on CPR whilst you open the box, and do this, X, Y, and Z.” Then that's fine. But if you don't know what you're doing, and you're on your own, it's just different. If you're on your own or not on your own, I think that makes ... There would have to be really strict algorithms in place for someone whoever's talking through it if you're on your own, versus not on your own.

I:	So, if you weren't on your own, you'd get the second person to perhaps get the defibrillator from the drone ...

R:	Yes. Yeah.

I:	...  would that be the case ...

R:	Absolutely.

I:	... if you're in a public ...

R:	Yeah.

I:	... location as well as if you were at your home? You would tell them ...

R:	Yeah. Wherever you are.

I:	Yeah. Wh- what ...

R:	You could get one person to do the compressions, and I could do the defib, or vice versa. It doesn't matter. But if you only got one pair of hands and one patient, what do you prioritise? The compressions, or the defib? I think you have to, based on life support, you have to put, you have to put the defib first, 'cause that's the first thing that's gonna get ... Compressions is never, never gonna make someone come back to life, but the defib will. But I, I think, given the state I’m in, I would need someone to, to say ... If it were me in that scenario again, I, I’d need somebody to say to me, "Go down and get it. You need to give it to him (?). It's the only thing that will save their life.” If they hadn't of said that to me, I would have gone, "No, I’m staying with him. I’m waiting for the paramedics.” So, I think it's a lot about how it's delivered to you. Whereas if you're in a crowd of people, then you can manage with others to help you. If you're on your own, and you want, you're trying to help this person live, it's ...

I:	Yeah.

R:	... it's hard.

I:	Yeah.

R:	So, you need to be really strict with the algorithms from the call handler, or whoever it is that’s you're dealing with.

00:46:31

I:	Yes. And I was going to say to you, do you think it would help you if the call handler said, "Right. Now you need to go downstairs to get the ...”

R:	Yes.

I:	“... defibrillator from the drone.”

R:	Yeah. It needs to be absolutely clear, clear instructions, on a very easy to follow algorithm that they're trained to follow.

I:	Yes. Yes. And if they’re ...

R:	You couldn't do it without that.

I:	Yeah. So, it would be, yeah, import …crucial actually, that that's included in their script, that they tell you ...

R:	Yeah.

I:	... when to, you need to leave. Would you be willing to get the defibrillator over the road, across the road if you were at home? Or how far would you be willing, if instructed by the call handler.

R:	No, if I were ... Well, again, if I’m on my own, I wouldn't be ... A) I'd have to leave my front door open. I'd be worried that I haven't got my keys with the front door shut behind me. So, I wouldn't want to leave my house, in case I can't get back in for my loved one. But that's one thing. I would, no, if I were on ... So, it’s again two answers. If someone, if I’m on my own, no, no, no, no, no. It's delivered to me where I’m at, or it's not ... Not happening, 'cause I wouldn't want to be gone, and anything could happen in the meantime. If I was with other people, yes, they could go and get it. So, very much depends, do you on your, is the bystander on their own? Is the bystander with other people?

I:	What about if the call handler said, "You can get it over the road.” Would you? And you're on your own.

R:	No.

I:	No.

R:	If I’m on my own, no.

I:	You would override them in that scenario, yeah.

R:	Yeah, because what you do about your front door shutting behind you? Can't get back in your house, it's the middle of the night, you're in a nightdress. You're in a really crappy area. How does that all work? No. Woman on your own in the middle of the night in a skimpy nightdress coming out? No, not happening.

I:	Okay. Are you okay to continue the last few questions, 'cause I know we're running over. Do ...

R:	Yeah.

I:	... that's okay?

R:	Sure.

I:	Would it make it easier or more difficult for you to use a defibrillator if it was delivered to you by drone?

R:	(Pause) I think, I don't think it matters. It's easy to use the defibrillator. The fact is, you need to use it.

I:	Yeah.

00:48:47

R:	I think it's a *fantastic* idea to send it by drone, because if it can get there within a couple of minutes of making the 999 call, of course. Why wouldn't you want it?

I:	Yeah. Sure, yes. And we've said that the call handler would need to instruct the bystander on when to leave the patient to get the defibrillator from the drone, although you would veto, you'd veto that, if they said for you across the road, 'cause that would be too far for you. But what else do you, what other role or involvement do you think the call handler should have in this, if anything?

R:	(Pause) Do you know, it's all silly stuff, because there, there's so many nuances in these scenarios, you know, where you are, who you're with. What the time of day is. For me personally, in the scenario I was in, [very early] in the morning, in a skimpy night dress, in a not very nice area, there's no way that I would leave the house. So, I would, you know, someone could say to me, encouraged me that this is the best thing for the person that I’m with, "Make sure you put your door on a lock, or take a key with you. You might want to put a coat on.” You know, just think about that person and the situation they're in, you're not going ... Whereas, if it was [middle of] the afternoon rather than [very early] in the morning, and we're downstairs, and I can put the door on the latch 'cause I, you know, it's easier to run out, and I’m not worried about ... I can see who's out there, it's not dark, and I, the kids aren't in the park smoking pot and might come in my house, and ... Do you know what I mean? There are so many nuances, that I think it would be really helpful, but probably impossible, for the call handler to have some understanding of the scenario that the bystander is in. Because there's no point saying, "Go and get it, go and get it,” if you're in a skimpy nightie in a horrible area it's [very early] in the morning, with kids smoking pot outside your front door. Then you're not gonna do it.

I:	Yes.

R:	But if you're in that scenario and there's a 15 year old child in the house, they could go and get it. So, I think there are different scenarios based on who you are, where you live, what the time of day is. Who's in the house, how many bystanders are there? It's not just a simple algorithm for one person, you know, to say, "Go do X, Y, and Z.” I mean, some people might just do it. I wouldn't personally, for all the aforementioned reasons!

I:	Yeah. So, would you want to know that one was coming, a drone was coming? Or only when it was there, or ...

R:	Yes.

I:	... how to identify it? What ...

R:	I'd like, no, I would definitely want to know when it was coming, because time is of an essence. And you feel, when you're doing the CPR, you feel that time is going *really*, *really* slowly. Everything's slow, you having this out of body experience. It's almost like you're watching something occur in slow motion. You're completely panicked that this person's gonna die. You want all that help now. So, I would, I think it'd be really good if they could say, "The drone is on its way. The drone will be here in three minutes. Three minutes 30, three minutes, two minutes 30.” A kind of count down. “Come on, carry on doing the compressions. You're doing really well. It's nearly here. It's nearly here.” That's for me, other people, of course, might not want any of that. But I just think, for me, I would want that reassurance that help is *really* nearby. *Really* near at hand. But I would still want the paramedics, because where are the people that are gonna do the intubation? Where are the people that’re gonna put the drip in? Of course, I’ve got that knowledge. If I hadn't’ve got that knowledge, then I’d just trust the NHS to deliver me a defib, I’m gonna press it, and my loved one's gonna be fine. What happens with the paramedics? That's my next question. It's all very well having a drone, but then what? Are they gonna drone you down the giving (?) set (?), the adrenaline? What will happen (?) the the intubation kit, or does the paramedic still come?

00:52:45

I:	Yes, they would come ...

R:	But I don't think a drone is, is good on its own. So, and then what? So, you've got your things sort, chain of survival. Well, where does the drone fit into the chain of survival, and where do the paramedics fit in? Because actually, without the paramedics, he'd be dead. Even if I had a defib, he'd still be dead. You know, he had to be intubated, and bagged, and ... So, there's cardiac risk, isn't there? Also, if someone's terminally ill and they're having a, a card, had a cardiac arrest, are you're still gonna send a drone? So, there’s a whole thing about, you know, have they signed a Do Not Resuscitate? That's a whole other thing. No one asked me that on the phone. “Have they got terminal illness? Do they wanna be resus- (chuckles slightly) -citate?” It's, so many ethical issues, there's so many, there's *so* much around this. Sorry, my background’s public health, so I’m a bit of a nightmare. But yeah there, it is not just simply, "We're gonna deliver you a drone.” There's a chain of survival. How does it all link in together there? What are the algorithms? Oh yeah.

I:	Yeah.

R:	Great idea. I love the idea. However, where, how does it all work in the chain of survival, or are they adapting the chain of survival?

I:	Yes. Yes. I don't think it would replace paramedics. Like you say, it would give them ...

R:	Oh, that’d be good.

I:	... the bystander the opportunity to defibrillate on scene, while the help was coming.

R:	Yeah. I think that, I think anything that can, if they can do that, that's brilliant.

I:	But, you know, it might be useful to have somebody assist the bystander to interact with the drone, even before the paramedics.

R:	Agree.

I:	As I say, this isn’t ...

R:	Yeah. Yeah.

I:	... up and running at the moment. We're, we’re exploring what might or might not work.

R:	Yeah.

I:	And that's why we, you know ...

R:	Yeah.

I:	... speaking to you, and we find it really useful (?) …

R:	Yeah. I think, I think you really have to look at whether the bystander is on their own, somewhere where no one else is gonna come to help, or if the bystander is with other people. Because I think that would make a huge, personally, I think for me it would make a huge difference, but if you spoke to a thousand bystanders who are on their own, of course, who knows what, what the outcomes would, what people would say.

I:	Yes. Yeah. Okay. And do you have any other concerns or problems with this technology? Any other issues perhaps, or do you think you've covered ...?

00:55:10

R:	Oh no, I think it's, in term, I mean obviously Amazon are using drones to deliver parcels? (Chuckles slightly) some players are using drones to deliver bombs! So, it's got all sorts of neat, you know, drones are all over the place now. I think anything that can help somebody survive a cardiac arrest, absolutely get it to them as quickly as possible by the, by the best means. And I think it's a fantastic idea. I just think it's the application of it, and how, how you're helped with it by whoever's on the end of the phone. That would be my concern.

I:	So, call handler has a ...

R:	And I think ... Call handler, and whether you're on your own or with other people, I think, for me. In the scenario I was in, I would not have gone and done it.

I:	No. No.

R:	But, but, you know, I’m trained old school. So, train new school that the drone will come, then my attitude would be different. That's what I learn, that you call 999, and they deliver you a drone and then the paramedics arrive, hey, fantastic!

I:	Yeah. That would be okay for you? Or are you saying it would be strange?

R:	Absolutely.

I:	Yeah, that would be okay.

R:	No! I think if I was trained in that way. So, I was trained on [Name of device], you know, or the, for 40 years. This is what you do, and there's a script, and this is what you follow. But obviously the training for resuscitation, for resuscitation would need to change, that you do your looking around you try find a pulse blah, blah, blah, help, help, help. And then you phone 999, and then the drone arrives with the defib. So, they, you know, the whole training programme needs to be updated, because all of us old school people wouldn't expect to be trained (inaudible). But if you trained everyone that that's part of resus now, that’d be great.

I:	Yeah. Yes. Okay. So, it definitely will have some knock on effects is what you're saying, in terms ...

R:	Yeah.

I:	Yeah. Training, and other things that you've mentioned. Excellent. Thank you. So, I just have some final brief questions that may be useful for our research. So, if I may, what is your age and gender?

R:	I’m female, and I’m 61.

I:	Okay. And you've said that you've had training in CPR. What about defibrillator use?

R:	Yeah, I’m a train, I am trained in using a defibrillator, and I have got the certificates to train others to use defibrillators.

I:	Okay. And was the CPR and defibrillator use training within the last five years?

R:	Yes.

I:	Okay. And this training, did it occur before or after the incident that we've discussed?

R:	After.

I:	So, CPR was after as well, or ...?

00:57:51

R:	Well, no, I’ve, I’ve, CPR training, I’ve had every year since the age of 18 ...

I:	So, that's before.

R:	... because in nursing and midwifery, you get, you have to do basic life support training every year, as basic, basic training, really. Can't, can’t remember what it's called now. So, yeah, I’ve done CPR, basic life support, I’ve done every year for 40, well, 30 odd years. Learning the, I became a trainer in emergency first aid at work, and as part of that training, I also was trained, I passed all the exams to train others in defibrillator use. So, I was trained to use it, and to teach others to use them.

I:	And was that ...

R:	And then I ...

I:	Sorry.

R:	... and that was within the last five years after my husband's cardiac arrest.

I:	Okay. Thank you for confirming. And did you have something else that you wanted to add, or say? No?

R:	No.

I:	Okay. So, yes, I was gonna ask you is there something else you'd like to add to your answers today, but that's fine. Excellent. Thank you. So, I’m just going to stop the recording, and go through what will happen next, if that's okay.

**END OF INTERVIEW**
