# Supplementary material for: Bystanders’ attitudes towards drone delivered Automated External Defibrillators for out-of-hospital cardiac arrest: A qualitative interview study
Source: PLoS One. 2025 Dec 3;20(12):e0337082. doi: 10.1371/journal.pone.0337082 (PMC12674532; doi:10.1371/journal.pone.0337082)
Supplement: Attachment — Submitted filename: Response to reviewers.docx [file pone.0337082.s008.docx]

# **Response to reviewers**

Thank you for your review of the manuscript, and for the opportunity to resubmit. We hope the changes that we have made have resulted in an improved manuscript.

Comments to the Author

Have the authors made all data underlying the findings in their manuscript fully available?

Reviewer #1: No

Reviewer #2: No

**Please note that we have now made anonymised transcripts available in the Supporting information submitted with this revision. We have clarified that the transcripts have been checked to make sure they were anonymised and we have further redacted any potentially identifiable information. We have clarified that participants were made aware that anonymised interview data could be shared in future, and that they provided explicit consent for this.**

Reviewer #1:

The investigators explored public attitudes towards drone-delivered AEDs for OHCA by conducting 14 semi-structured interviews with real-life OHCA bystanders. They identified 5 themes and devised interventions.

1. The sample size is small and it is unclear whether the subjects are representative of the general population. Nevertheless, the study provides clues on the complexity of the process. I suggest that the authors acknowledge the small sample size in the limitations section.

**We have added a section to the limitations**

**“The sample size in this study is small and the participants might not be representative of the general population. It may have been that more motivated or confident bystanders were more likely to put themselves forward as participants. Certainly, a higher proportion of our participants reported CPR/AED training than the general UK population [42].”**

2. The availability of AED doesn’t guarantee it being used. I would cite the Home use of AED trial (HAT), which did not improve the OHCA outcomes (DOI: 10.1056/NEJMoa0801651). One of the reasons for failure was insufficient use of the AEDs, despite resuscitation training, including AED, provided to the caregivers in the study.

**We agree that this is an important point. We have added the following text, including the above-mentioned reference:**

**“Further, there is no guarantee that an AED will be used if available, which has been demonstrated in studies reporting the use of AEDs in both public [39] and home [40] settings.”**

3. OHCA outcomes are better when the resuscitation response is integrated. Incorporation of drone-delivered AEDs require an integrated response from multiple stakeholders, including resuscitation services, drone operators, regulators, call handlers, CPR training providers. I would cite this paper (DOI: 10.1016/j.resuscitation.2016.10.029), tying the positive outcomes to integration of resuscitation response.

**Again, we agree that this is an important point. We have added the following text, including the suggested reference:**

**“It is important that any drone-delivery system that is implemented at scale is fully integrated into the community and ambulance service response to out-of-hospital cardiac arrest, which will ensure the best chance of improved patient outcomes [45].”**

Reviewer #2:

This is a well-conducted and timely qualitative study exploring bystanders’ perspectives on drone-delivered AEDs in OHCA. It makes an important contribution to understanding public acceptance and barriers to implementation of emerging prehospital technologies.

Strengths:

Clear use of theoretical frameworks (TDF, COM-B, Behaviour Change Wheel) to guide analysis and intervention development.

Rich participant data, with well-chosen illustrative quotes.

Identification of practical intervention areas (call-handler scripts, training, co-responder models) with real-world applicability.

Transparent reporting following COREQ guidelines.

**Thank you for your feedback.**

Suggestions for minor revision:

1. Data availability statement: Clarify that anonymised transcripts are available on request because of participant confidentiality, as PLOS requires explicit justification for restrictions.

**We have now made anonymised transcripts available in the supplementary material submitted with this revision.**

2. Methods – saturation: Expand on how thematic saturation was determined (e.g., what criteria were used at 14 interviews to conclude no new insights were emerging).

**Thank you, we have expanded the relevant section in the methods so that it now reads:**

**“We conducted 14 eligible interviews, at which point we determined we had obtained a sufficiently thorough and nuanced understanding of the topics presented [36]. This was determined by the interviewer (CJB) after discussion with CMS, and was based not only on no new codes being identified but also on agreement that we had a rich understanding of the topic at hand that was unlikely to be added to by additional recruitment.”**

3. Transferability of findings: Consider discussing how perceptions of drone technology may vary internationally and whether the UK-specific context (regulation, AED availability) limits generalisability.

**We have added a section in the limitations:**

**“Further, these findings may not be fully applicable outside the UK. Whilst bystanders in simulation studies internationally generally respond positively to drones delivering AEDs [17,21-23], the delivery model in the UK may vary from those in other countries. Reasons for this may include differences in aviation regulations, existing AED availability and ambulance response times. This potentially limits the applicability of our findings to other countries.”**

4. Limitations: Expand slightly on the possible impact of self-selection bias (motivated or more confident bystanders may have been overrepresented) and the exclusion of suspected fraudulent respondents.

**We have added the following limitations:**

**“The sample size in this study is small and the participants might not be representative of the general population. It may have been that more motivated or confident bystanders were more likely to put themselves forward as participants. Certainly, a higher proportion of our participants reported CPR/AED training than the general UK population [42].”**

**and (expanding our description of potential fraudulent responses):**

**“Nonetheless, determining whether or not participants were genuine was subjective [35], and so some uncertainty must remain about around the validity of other participants whose data *has* been analysed. If we have erred in our decisions to exclude participants based on suspected fraud, this may impact the validity of our results.”**

5. Language/clarity: Minor editorial corrections (e.g., sentence flow in Background and Discussion) will improve readability.

**We have reviewed the manuscript and made improvements where possible.**

Overall, this is an important and well-executed manuscript that will interest both emergency medicine and health policy audiences. I recommend acceptance after minor revisions.

**Thank you for your feedback.**

Journal Requirements

When submitting your revision, we need you to address these additional requirements.

1. Please ensure that your manuscript meets PLOS ONE's style requirements, including those for file naming. The PLOS ONE style templates can be found at https://journals.plos.org/plosone/s/file?id=wjVg/PLOSOne_formatting_sample_main_body.pdf and <https://journals.plos.org/plosone/s/file?id=ba62/PLOSOne_formatting_sample_title_authors_affiliations.pdf>

**We have made sure that the documents are formatted appropriately, as per these instructions.**

2. We note that you have indicated that there are restrictions to data sharing for this study. PLOS only allows data to be available upon request if there are legal or ethical restrictions on sharing data publicly. For more information on unacceptable data access restrictions, please see <http://journals.plos.org/plosone/s/data-availability#loc-unacceptable-data-access-restrictions>.

Before we proceed with your manuscript, please address the following prompts:

a) If there are ethical or legal restrictions on sharing a de-identified data set, please explain them in detail (e.g., data contain potentially identifying or sensitive patient information, data are owned by a third-party organization, etc.) and who has imposed them (e.g., a Research Ethics Committee or Institutional Review Board, etc.). Please also provide contact information for a data access committee, ethics committee, or other institutional body to which data requests may be sent.
b) If there are no restrictions, please upload the minimal anonymized data set necessary to replicate your study findings to a stable, public repository and provide us with the relevant URLs, DOIs, or accession numbers. For a list of recommended repositories, please see <https://journals.plos.org/plosone/s/recommended-repositories>. You also have the option of uploading the data as Supporting Information files, but we would recommend depositing data directly to a data repository if possible.

We will update your Data Availability statement on your behalf to reflect the information you provide.

**We have attached anonymised transcripts as part of our revised submission. Our funder requires that all research outputs be open-access, so there will not be any restriction in accessing these along with the revised manuscript.**

3. In the online submission form, you indicated that the anonymised transcripts generated during the current study and our analyses of them are stored securely at the host institution and are available from the corresponding author on reasonable request. Due to the risk of participant identification we will not share interview recordings.

All PLOS journals now require all data underlying the findings described in their manuscript to be freely available to other researchers, either 1. In a public repository, 2. Within the manuscript itself, or 3. Uploaded as supplementary information.

This policy applies to all data except where public deposition would breach compliance with the protocol approved by your research ethics board. If your data cannot be made publicly available for ethical or legal reasons (e.g., public availability would compromise patient privacy), please explain your reasons on resubmission and your exemption request will be escalated for approval.

**As above, we have attached anonymised transcripts as part of our revised submission. Our funder requires that all research outputs be open-access, so there will not be any restriction in accessing these along with the revised manuscript.**

**We have clarified that the transcripts have been checked to make sure they were anonymised and we have further redacted any potentially identifiable information. We have clarified that participants were made aware that anonymised interview data could be shared in future, and that they provided explicit consent for this.**

4. Thank you for stating the following in the Competing Interests/Financial Disclosure * (delete as necessary) section:

Christopher M Smith has volunteer roles with Resuscitation Council UK, European Resuscitation Council and the International Liaison Committee on Resuscitation. Keith Couper is chair of the National Institute for Health Research (NIHR) Research for Patient Benefit funding committee for the West Midlands region in the UK. Nigel Rees receives R&D funding from Health & Care Research Wales who also provided UK NHS delivery funding for this project. He is also Assistant Director for Research & Innovation for Welsh Ambulance Services University NHS Trust.

We note that you received funding from a commercial source: [Name of Company]. Please provide an amended Competing Interests Statement that explicitly states this commercial funder, along with any other relevant declarations relating to employment, consultancy, patents, products in development, marketed products, etc.

Within this Competing Interests Statement, please confirm that this does not alter your adherence to all PLOS ONE policies on sharing data and materials by including the following statement: "This does not alter our adherence to PLOS ONE policies on sharing data and materials.” (as detailed online in our guide for authors http://journals.plos.org/plosone/s/competing-interests). If there are restrictions on sharing of data and/or materials, please state these. Please note that we cannot proceed with consideration of your article until this information has been declared.

Please include your amended Competing Interests Statement within your cover letter. We will change the online submission form on your behalf.

**Apologies, but we are confused here. You only put “[Name of Company]” above. We did not receive any funding from a commercial organisation. We received funding from the National Institute for Health and Care Research (NIHR) and Health and Care Research Wales (HCRW) only.**

5. Thank you for stating the following financial disclosure:

This study was funded by the National Institute for Health and Care Research (NIHR) under its Research for Patient Benefit programme (Grant Reference Number NIHR204382) and Health and Care Research Wales (IRAS: 318417). The views expressed are those of the author(s) and not necessarily those of the NIHR, the Department of Health and Social Care or Health and Care Research Wales.

Please state what role the funders took in the study. If the funders had no role, please state: "The funders had no role in study design, data collection and analysis, decision to publish, or preparation of the manuscript." If this statement is not correct you must amend it as needed.

Please include this amended Role of Funder statement in your cover letter; we will change the online submission form on your behalf.

**The funders had no role in study design, data collection and analysis, decision to publish, or preparation of the manuscript We have updated the cover letter with the relevant information.**

6. Please amend your authorship list in your manuscript file to include author Christopher Matthew Smith, Celia J Bernstein, Carl Powell, Mary O'Sullivan, Mark Holt, Keith Couper, Nigel Rees.

**We have updated this.**

7. Please amend the manuscript submission data (via Edit Submission) to include author Bernstein, C J, Smith, C M, Powell, C, O’Sullivan, M, Holt, M, Couper, K, Rees, N.

**We have updated this**

8. Please remove all personal information, ensure that the data shared are in accordance with participant consent, and re-upload a fully anonymized data set.

Note: spreadsheet columns with personal information must be removed and not hidden as all hidden columns will appear in the published file.

Additional guidance on preparing raw data for publication can be found in our Data Policy (https://journals.plos.org/plosone/s/data-availability#loc-human-research-participant-data-and-other-sensitive-data) and in the following article: <http://www.bmj.com/content/340/bmj.c181.long>.

**We have done this.**

9. Please include captions for your Supporting Information files at the end of your manuscript, and update any in-text citations to match accordingly. Please see our Supporting Information guidelines for more information: <http://journals.plos.org/plosone/s/supporting-information>.

**We have done this.**

10. If the reviewer comments include a recommendation to cite specific previously published works, please review and evaluate these publications to determine whether they are relevant and should be cited. There is no requirement to cite these works unless the editor has indicated otherwise.

**We have done this. The suggestions made by reviewers were entirely appropriate and we have incorporated them into our revision.**

11. Please review your reference list to ensure that it is complete and correct. If you have cited papers that have been retracted, please include the rationale for doing so in the manuscript text, or remove these references and replace them with relevant current references. Any changes to the reference list should be mentioned in the rebuttal letter that accompanies your revised manuscript. If you need to cite a retracted article, indicate the article’s retracted status in the References list and also include a citation and full reference for the retraction notice.

**We have done this.**
